# Aestuariibius violaceus sp. nov., isolated from a marine limpet Cellana toreuma

**DOI:** 10.1099/ijsem.0.006834

**Published:** 2025-07-08

**Authors:** Min Seo Lee, Mi-Jeong Park, Purena Son, Taekeun Rho, Kae Kyoung Kwon, Jin-Sook Park

**Affiliations:** 1 Marine Biotechnology Research Center, Korea Institute of Ocean Science & Technology, 385, Haeyang-ro, Yeongdo-gu, Busan, 49111, Republic of Korea; 2 Department of Biological Sciences and Biotechnology, Hannam University, Daejeon 34054, Republic of Korea; 3 Marine Environment Research Department, Korea Institute of Ocean Science & Technology, 385, Haeyang-ro, Yeongdo-gu, Busan, 49111, Republic of Korea; 4 KIOST School, University of Science and Technology, Daejeon 34113, Republic of Korea

**Keywords:** *Cellana toreuma*, genus *Aestuariibius*, genus *Aestuariibius *metabolism, Ulleung Island

## Abstract

A novel bacterium, designated strain 2305UL40-4^T^, was isolated from *Cellana toreuma* (a Korean limpet) collected from Ulleung Island, Republic of Korea. The cells were Gram-stain-negative, aerobic, rod-shaped (0.5–0.9 µm×1.5–2.2 µm) and non-motile. Growth was observed at 22–36 °C (optimum 25–30 °C), pH 6–8 (optimum pH 7) and in the presence of 0.5–4.0% (w/v, optimum 2.0%) NaCl. The 16S rRNA gene sequence analysis revealed that strain 2305UL40-4^T^ shared 97.4% similarity with *Aestuariibius insulae* DBTF-13^T^. The average nucleotide identity value between the two strains was 76.5%, and the digital DNA–DNA hybridization value was 19.0%. Both values are well below the species delineation thresholds. The DNA G+C content of strain 2305UL40-4^T^ was 63.8 mol%. The major fatty acids were C_16 : 0_, C_18 : 0_ and summed feature 8 (C_18 : 1_* ω7*c and/or C_18 : 1_* ω6*c). Major polar lipids included phosphatidylethanolamine, phosphatidylglycerol, diphosphatidylglycerol, phosphatidylcholine, five unidentified aminolipids and fifteen unidentified lipids. Ubiquinone-10 was the predominant quinone. The new strain differs from *A. insulae* in its ability to utilize d-raffinose, d-ribose and d-xylose. Based on these polyphasic taxonomic characteristics, strain 2305UL40-4^T^ is proposed as a novel species within the genus *Aestuariibius*, with the name *Aestuariibius violaceus* sp. nov. The type strain is 2305UL40-4^T^ (=KCCM 43505^T^=MCCC 1K09164^T^).

## Introduction

We describe a novel bacterial strain, 2305UL40-4^T^, isolated from *Cellana toreuma* (a Korean limpet) inhabiting marine environments. Strain 2305UL40-4^T^ belongs to the genus *Aestuariibius*, which was initially proposed by Park *et al*. [1]. As of June 2025, the only species within the genus *Aestuariibius* is *Aestuariibius insulae* DBTF-13^T^, which was isolated from a tidal flat sediment. Members of the genus *Aestuariibius* are Gram-negative, aerobic, without flagella and rod-shaped or filamentous long rod-shaped. Catalase and oxidase activities are present. Nitrate reduction is positive. The main ubiquinone is ubiquinone-10 (Q-10). l-Arabinose, d-fructose, d-galactose, d-glucose, maltose and d*-*mannose are utilized as carbon and energy sources. The main fatty acids are C_18 : 1_* ω7*c and C_16 : 0_, and the DNA G+C content is 61.6–63.8 mol%. However, genomic analysis and genome-based studies of *A. insulae* DBTF-13^T^ have not been conducted. The intertidal environment from which strain 2305UL40-4ᵀ was isolated is generally characterized as a coastal region with high sulphate (SO₄²^-^) concentration and is highly sensitive to climate change and human activities such as destruction and pollution caused by coastal infrastructure development and the discharge of industrial effluents [2,3]. The sulphur oxidation-related functions carried out by the Sox system are essential for supporting the host’s adaptation and survival in the sulphur-rich intertidal environment through contributing to energy production and detoxification processes, highlighting their significance in the symbiotic relationship [4]. In this study, we conducted a comprehensive genomic analysis of *A. insulae* DBTF-13^T^ and examined the taxonomic status and genome features of strain 2305UL40-4^T^.

## Methods

### Isolation and cultivation

On 23 May 2023, specimens of *C. toreuma* (a Korean limpet) were collected from the eastern coast of Ulleung Island, Republic of Korea (37° 30′ 35′ N 130° 54′ 56′ E). The shells were removed, and the tissues were suspended in sterile artificial seawater (20 g NaCl, 2.9 g MgSO₄, 4.53 g MgCl₂·6H₂O, 0.64 g KCl and 1.75 g CaCl₂·2H₂O per litre). The suspension was serially diluted up to 10^−^⁴ and spread onto marine agar (MA) 2216 plates (BD, Difco). Then, the plates were incubated at 25 °C. After 1 week, distinct colonies were purified by subculturing on fresh MA plates. One pure isolate, designated as 2305UL40-4^T^, was preserved at −80 °C in 20% (v/v) glycerol for long-term storage. For 16S rRNA gene sequence similarity analysis, genomic DNA was extracted using the QIAamp DNA Mini Kit (Qiagen, Germany) according to the manufacturer’s instructions. PCR amplification of the 16S rRNA gene was performed using TaKaRa Ex Taq PCR mixture (Takara Bio, Japan) and bacteria-specific primer sets 27F (5′–AGAGTTTGATCMTGGCTCAG–3′) and 1492R (5′–TACGGYTACCTTGTTACGACTT–3′) (expected amplicon size is ~1,450 bp). PCR products were purified using the QIAquick PCR Purification Kit (Qiagen, Germany), and sequencing was performed by Macrogen Co., Ltd. (Seoul, Korea). The resulting 16S rRNA gene sequences were edited using EditSeq and compiled using SeqMan (DNASTAR). Closely related strains were identified via blast against the GenBank database and the EzBioCloud server (http://www.ezbiocloud.net) [5]. Based on the 16S rRNA gene similarity, *A. insulae* KACC 19432ᵀ (purchased from the Korean Agricultural Culture Collection) was selected as the reference species. Unless otherwise stated, cells were cultured at 25 °C for 3 days.

### Phylogenetic analysis and whole-genome analysis

Since the whole-genome sequence is not available for the related species, *A. insulae* KACC 19432ᵀ, whole-genome extraction and sequencing were performed for strain KACC 19432ᵀ together with strain 2305UL40-4ᵀ. The DNA extraction was conducted according to the procedure described above. The concentration and purity of extracted DNA were assessed using the Qubit dsDNA Assay Kit (Invitrogen) with a Qubit 4.0 fluorometer. Whole-genome sequencing was conducted using the Illumina iSeq 100 platform [6]. For unbiased paired-end sequencing (2×150 bp), the extracted total DNA was prepared using the Illumina DNA Prep Kit (Illumina, Inc.) according to the manufacturer’s instructions. The sequencing library was indexed and barcoded with IDT^®^ for Illumina^®^ DNA/RNA UD Indexes (Illumina, Inc.). Library quantification was performed using the Qubit dsDNA Assay Kit (Invitrogen) on a Qubit 4.0 fluorometer. The quality of raw sequencing reads was evaluated using FastQC v0.12.1 [7] with default parameters to assess base quality scores, G+C content and adapter contamination. Quality trimming was performed using Cutadapt v4.4 [8]. Bases with a Phred quality score below 20 at either end of the paired-end reads were removed (-q 20,20), and reads shorter than 50 bp after trimming were discarded (-m 50). Processed paired-end reads were retained using the -p option. Further quality filtering of paired-end reads was performed using Trimmomatic v0.39 [9]. Bases with quality scores below 3 were trimmed from both the leading and trailing ends (LEADING:3, TRAILING:3). When the average quality score within a four-base window fell below 20, reads were trimmed using a sliding window approach (SLIDINGWINDOW:4 : 20). Reads shorter than 50 bp after trimming were discarded (MINLEN:50). Paired and unpaired reads were retained separately. Genome assembly was performed using SPAdes v3.15.5 [10] with multiple k-mer sizes (21, 33, 55, 77 and 99) to improve assembly quality. The --careful option was used to reduce mismatches and short indels by employing a built-in error correction algorithm. Coverage-based contig filtering was applied using the --cov-cutoff auto parameter. QUAST v5.2.0 [11] was employed to evaluate the genome assembly quality, focusing on total length, contiguity and potential misassemblies. In addition, genome completeness and contamination were evaluated using CheckM 1.2.3 [12] to ensure the quality of the assembled genome.

The genome assembly statistics of *Aestuariibius* spp. were analysed using the GAAS toolkit [13]. Considering the length and quality, the 16S rRNA gene sequence extracted from the whole genome of strain 2305UL40-4^T^ was used for phylogenetic analysis. Multiple sequence alignment was performed using Clustal W integrated in mega 11 [14], complemented by BioEdit [15]. The Kimura 2-parameter model was applied to construct phylogenetic trees using the neighbor-joining [16], maximum-likelihood [17] and maximum-parsimony [18] methods in mega 11. Whole-genome sequences of closely related species, selected by 16S rRNA gene-based phylogeny, were retrieved from GenBank and then compared (Table S1, available in the online Supplementary Material).

Genome annotation was conducted using Prokka [19]. A phylogenetic tree based on 400 universal protein markers extracted from whole-genome amino acid sequences was constructed using PhyloPhlAn v3.1.68 with the medium diversity setting and a customized configuration file (supermatrix_aa.cfg) [20]. The final concatenated alignment spanned 36,650 amino acid positions. Following Lee *et al*. [21], IQ-TREE2’s ModelFinder [22] selected LG+F+R4 as the best-fit substitution model. The robustness of the phylogenomic tree topology was evaluated with 1,000 ultrafast bootstrap replicates and approximate likelihood ratio tests (aLRTs).

Average nucleotide identity (ANI) values were calculated using the OrthoANI-usearch tool [23]. Digital DNA–DNA hybridization (*d*DDH) values were determined via the Genome-to-Genome Distance Calculator provided by DSMZ (Braunschweig, Germany). Amino acid identity (AAI) was computed with EzAAI version 1.2.3 [24], applying thresholds of a minimum of 20% identity and 50% query coverage. The Percentage of Conserved Proteins (POCP) was calculated by comparing the amino acid sequences of the two genomes using BLASTP as described by Qin *et al*. [25].

To predict secondary metabolite biosynthetic gene clusters (BGCs), the whole genome of strain 2305UL40-4^T^ was uploaded to the anti-SMASH 7.0 online server [26] (https://antismash.secondarymetabolites.org/). Pangenome analysis was performed using Roary 3.13.0 [27]. A comparative analysis of metabolic processes between strain 2305UL40-4^T^ and *A. insulae* KACC 19432^T^ was performed using KEGG BlastKOALA [28]. Additionally, the KofamKOALA tool [29] was employed to analyse the metabolic functions of strains in the genus *Aestuariibius*.

### Physiology and chemotaxonomy

Cell wall characteristics were determined using the method of Buck [30]. Morphological features of strain 2305UL40-4ᵀ were observed using transmission electron microscopy (TEM; Hitachi HT7800, HITACHI) after negative staining with 2% uranyl acetate. Growth of strain 2305UL40-4ᵀ was tested on MA 2216 plates at various temperatures (4, 10, 15, 20, 22, 25, 30, 35 and 45 °C) over 7 days. Growth at various initial pH values (3–10, at intervals of 1.0 pH unit) was assessed in marine broth 2216 using the following 10 mM buffers: citrate-phosphate buffer (pH 3–5), MES buffer (pH 6), tricine buffer (pH 7–8) and MEPSO buffer (pH 9–10). The pH was adjusted with 2N HCl and 2N NaOH. Salinity tolerance was evaluated in marine broth prepared with distilled water containing NaCl at concentrations of 0, 0.5 and 1–10% (w/v, at 1% intervals). Growth in pH and salinity tests was monitored for up to 5 days at 25 °C in a shaking incubator (180 r.p.m.) by measuring OD₆₀₀.

Oxidase and catalase activities were determined as previously described [31]. Anaerobic growth was tested using the GasPak EZ anaerobe pouch system (BD) for 1 week. Biochemical characterization was performed using API kits (API 20NE, API 50CH and API ZYM) according to the manufacturer’s instructions (bioMérieux, France). Results for API 20NE and API 50CH kits were observed after incubation at 25 °C for 24 and 48 h, respectively. Antibiotic susceptibility testing was conducted using the disc diffusion method described by Bauer *et al*. [32]. Dissimilatory nitrate reduction (DNRA) and denitrification were tested by inoculating strain 2305UL40-4ᵀ in 50 ml serum bottles with 20 ml of marine broth supplemented with 1% potassium nitrate, followed by purging with nitrogen gas (N_2_) for 3 min and incubation. To maintain a completely anaerobic condition by removing dissolved oxygen in the medium, 20 µl of a 100×reducing agent (Na₂S·9H₂O, 30 g l^−1^; cysteine-HCl, 30 g l^−1^) was used. Typically, this strain requires less than 3 days (72 h) to fully grow; therefore, the experiment was terminated after 3 days of incubation. Cultures were incubated at 25 °C, and cell numbers were counted every 24 h for 72 h using a QUANTOM Tx^™^ Microbial Cell Counter [33]. Dissolved nitrate and nitrite concentrations were measured using an autoanalyzer (QuAAtro, SEAL), with detection limits of 0.02 µM for both nitrate and nitrite [34].

Cells were cultivated in marine broth at 25 °C for 60 h to provide sufficient biomass for subsequent biochemical analysis. The cellular fatty acid profiles of strain 2305UL40-4ᵀ and *A. insulae* KACC 19432ᵀ were determined using the MIDI/Hewlett Packard Microbial Identification System (MIS) [35], employing Sherlock version 6.3 and the RTSBA6 database. Cellular quinones of strain 2305UL40-4ᵀ were analysed using HPLC with a reverse-phase C18 column, as described by Minnikin *et al*. [36]. Polar lipids were extracted using a chloroform/methanol system and separated by two-dimensional TLC on silica gel 60 F254 aluminium-backed plates (Merck) [36]. The first dimension was developed as a chloroform/methanol/water (65 : 24 : 4, v/v) solution, while the second dimension as a chloroform/glacial acetic acid/methanol/water (40 : 7.5 : 6 : 1.8, v/v/v/v) solution. Lipids were visualized with 10% (w/v) molybdatophosphoric acid, followed by heating at 150 °C for 10 min. Lipids containing free amino groups (e.g. phosphatidylethanolamine, aminophospholipids and aminolipids) were detected with 0.2% ninhydrin solution [37]. Phosphorus-containing lipids (e.g. phosphatidylglycerol and aminophospholipids) were identified using the Zinzadze reagent [38]. Sugar-containing lipids were detected with 0.5% (w/v) *α*-naphthol reagent, followed by heating at 100 °C for 10 min [39]. Phosphatidylcholine was identified using Dragendorff’s reagent.

## Result

### Phylogenetic analysis and genomic indices

**Fig. 1.**
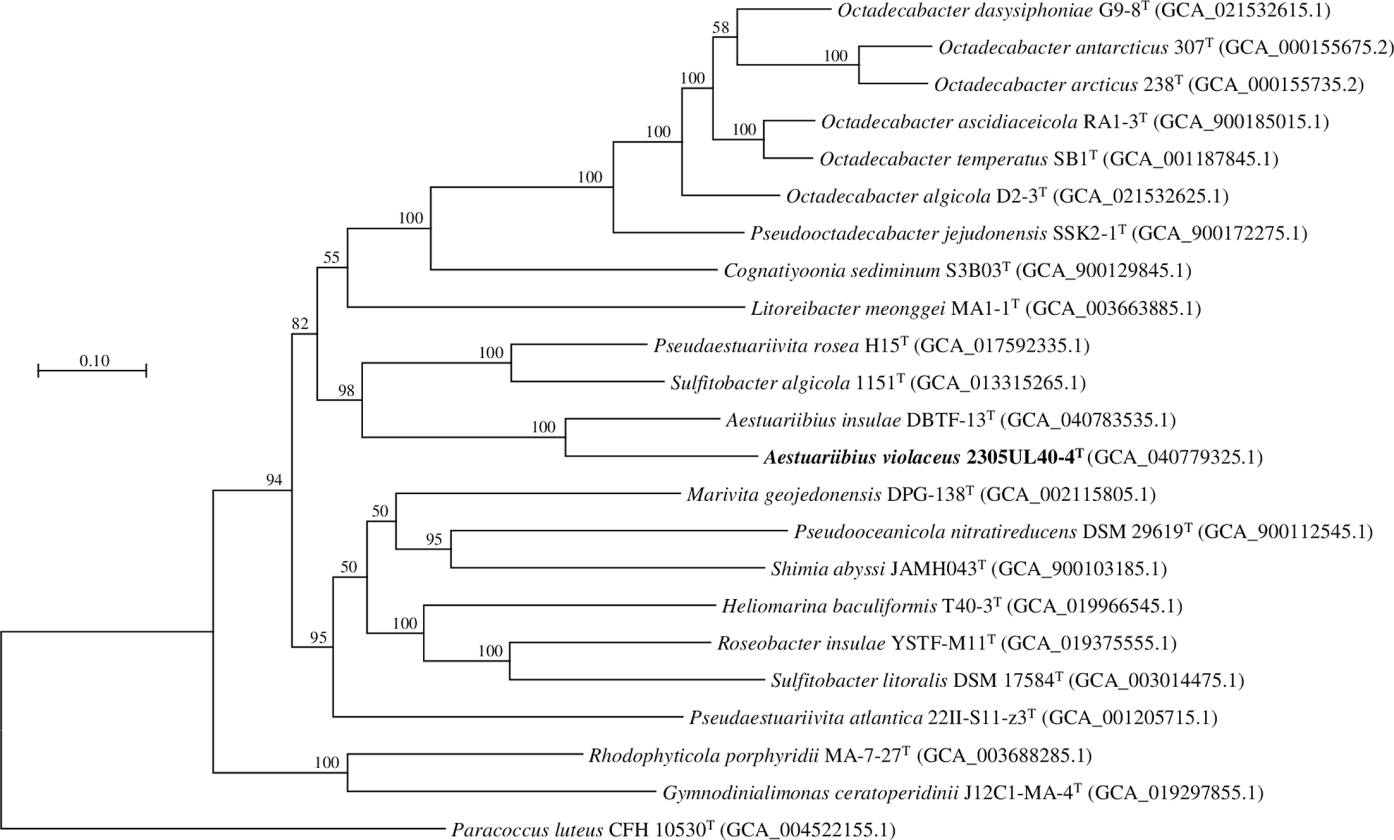
Phylogenomic tree constructed using IQ-TREE. The numbers at each node represent aLRT values obtained through bootstrap analysis with 1,000 replicates.

The obtained 16S rRNA gene sequence of strain 2305UL40-4^T^ by Sanger sequencing was 1,459 bp in length and spanning positions 55–1,444 within the *Escherichia coli* positioning system. Based on 16S rRNA gene sequence similarity, the closest relative to strain 2305UL40-4^T^ was *A. insulae* KACC 19432^T^ (97.4%), followed by *Pseudooctadecabacter jejudonensis* SSK2-1^T^ (96.72%), *Octadecabacter ponticola* HDSW-34^T^ (96.44%) and *Octadecabacter antarcticus* 307^T^ (96.25%). A comparison between 16S rRNA gene sequences obtained via Sanger sequencing and whole-genome sequencing showed 100% similarity for both *Aestuariibius* strains. The genome sequencing depths were 107.84× for strain 2305UL40-4^T^ and 81.24× for *A*. *insulae* KACC 19432^T^. The whole genome of strain 2305UL40-4^T^ was assembled into 43 contigs with a total size of 4.42 Mb, an N50 value of 350,313 bp, 4,398 CDSs, 3 rRNAs, 47 tRNAs and 1 tmRNA. For *A. insulae* KACC 19432^T^, the genome size was 3.71 Mb with 13 contigs, N50 value of 567,184 bp, 3,606 CDSs, 3 rRNAs, 45 tRNAs and 1 tmRNA (Table S1). In the phylogenetic tree constructed using the 16S rRNA gene, strains 2305UL40-4^T^ and *A. insulae* KACC 19432^T^ formed a clade with low bootstrap value (Fig. S1). In the phylogenomic tree, the two strains clustered into a single clade with high confidence, confirming that strain 2305UL40-4^T^ belongs to the genus *Aestuariibius* (Fig. 1). The ANI, *d*DDH, AAI and POCP values between strains 2305UL40-4^T^ and *A. insulae* KACC 19432^T^ were 76.3%, 19.0%, 73.9% and 70.6%, respectively. The ANI, *d*DDH and AAI values were lower than the species delineation thresholds, confirming that strain 2305UL40-4^T^ represents a novel species distinct from *A. insulae* (Table 1).

**Table 1.** Comparison of characteristics between (1) strain 2305UL40-4^T^ and (2) *A. insulae* KACC 19432ᵀ. Both strains are aerobic, Gram-reaction-negative and non-motile. Both are positive for nitrate reduction, oxidase and catalase activity and utilization of mannitol, aesculin and ferric citrate. Both are weakly positive for alkaline phosphatase, esterase activities and utilization of inulin. Both are negative for the activity of lipase, cystine arylamidase, *α*-chymotrypsin, *N*-acetyl-*β*-glucosaminidase, *α*-fucosidase, l-tryptophane and utilization of d-glucose, l-arginine, urea, gelatine, d-maltose, potassium gluconate, capric acid, adipic acid, malic acid, trisodium citrate, phenylacetic acid, glycerol, erythritol, d-arabinose, l-arabinose, l-xylose, d-galactose, d-glucose, d-fructose, d-mannose, l-sorbose, l-rhamnose, dulcitol, d-mannitol, d-sorbitol, methyl-*α* d-mannopyranoside, methyl-*α* d-glucopyranoside, *N*-acetylglucosamine, amygdalin, arbutin, salicin, d-cellobiose, d-maltose, d-lactose, d-melibiose, starch, xylitol, gentiobiose, d-turanose, d-fucose and l-fucose

Characteristic	1	2
Isolation		
Source	*C. toreuma*	Sediment
Location	South Korea, Ulleung Island	South Korea, Yellow Sea
Growth conditions (optimum in parenthesis)		
Temperature (℃)	22–36 (25–30)	10–30 (25–30)
pH	6.0–8.0 (7.0)	6.0–8.0 (7.0–8.0)
NaCl Cone. (%, w/v)	0.5–4.0 (2.0)	1.0–6.0 (2.0)
Morphological features		
Size (µm)	0.5–0.9×1.5–2.2	0.2–0.8×0.4–10.0
Shape	Rod	Rod, filamentous long rod
Colour	Magenta	Moderate orange
Cell components		
Major fatty acids (>5%)	C_16 : 0_, C_18 : 0_, summed feature 8*	C_16 : 0_, C_18 : 0_, 11-methyl C_18 : 1_ ω8c, summed feature 8*
G+C content	63.7 mol%	61.4 mol%
API 50CH and API ZYM test		
d-Ribose, d-xylose	w	−
d-Adonitol, inositol, trypsin	−	+
*β*-Glucosidase, *α*-glucosidase	w	+
d-Trehalose, d-melezitose	−	w
d-Raffinose	+	w

*Summed feature 8 (C_18:1_ *ω*7*c* and/or C_18:1_ *ω*6*c*).

w, weakly positive; −, negative.

Currently, the genus *Aestuariibius* is classified in the family *Paracoccaceae*. However, our findings suggest that it should be transferred to the family *Roseobacteraceae*. When Liang *et al*. [40] proposed the family *Roseobacteraceae*, the genus *Aestuariibius* was excluded due to the absence of genomic data. In addition, *Gymnodinialimonas ceratoperidinii* was proposed during the establishment of *Roseobacteraceae* and remains within the *Paracoccaceae*. Our results indicate that members of the genus *Gymnodinialimonas* [41,42] should also be transferred to the family *Roseobacteraceae*.

Still, phylogenetic analysis of order *Rhodobacterales* remains a challenging task. The 16S rRNA gene-based phylogenetic trees do not clearly resolve their positions, making taxonomic assignment ambiguous (Fig. S1). According to Zhang *et al*. [43], the phylotaxonomic assessment based on four core gene sets suggested the need for re-evaluation of the family-level classification within the *Paracoccaceae* and *Roseobacteraceae*. Their result of this study supports the idea that classifications based on limited markers alone may not accurately reflect evolutionary relationships. Our results are consistent with this conclusion and suggest that the genus *Aestuariivius* should be transferred to the family *Roseobacteraceae* based on genome-based phylogenetic analyses.

### Phenotypic and physiological analysis

Cell images of strain 2305UL40-4ᵀ obtained by TEM were rod-shaped (0.5–0.9 µm×1.5–2.2 µm) without flagella (Fig. S2). The strain exhibited optimal growth at 25–30 °C, at pH 7.0 and with 2.0% NaCl. When cultivated on MA over a 5-day incubation at 25 °C, the colony colour initially appeared between light purple and white and eventually turned into a distinct light purple. The predominant respiratory quinone was Q-10. The predominant fatty acids were C_16 : 0_ (13.9%), C_18 : 0_ (5.6%) and summed feature 8 (C_18 : 1_* ω7*c and/or C_18 : 1_* ω6*c) (62.4%), which were very similar to those of *A. insulae* KACC 19432ᵀ. However, the proportion of 11-methyl C_18 : 1_* ω7*c differed notably (Table S2). The major polar lipids included phosphatidylethanolamine, phosphatidylglycerol, diphosphatidylglycerol and phosphatidylcholine, along with five unidentified amino lipids and fifteen unidentified lipids (Fig. S3). API test results revealed weak positive reactions for d-ribose, d-xylose, d-sucrose, inulin, glycogen, d-lyxose, d-tagatose and d-arabitol. They are positive for enzymatic activities of alkaline phosphatase, esterase, esterase lipase, valine arylamidase, naphthol-AS-BI-phosphohydrolase, *β*-glucuronidase, *α*-glucosidase, aesculin ferric citrate hydrolysis, *β*-galactosidase and utilization of d-raffinose and nitrate reduction. All other API test reactions were negative. Antibiotic sensitivity testing revealed that strain 2305UL40-4ᵀ was sensitive to cefuroxime sodium, ampicillin, ofloxacin, sulphamethoxazole/trimethoprim, piperacillin, ampicillin/sulbactam, ciprofloxacin and chloramphenicol. It was weakly sensitive to cefoxitin, amikacin, tetracycline and meropenem, but resistant to cephalothin and amoxicillin/clavulanic acid (2 : 1). *A. insulae* KACC 19432ᵀ was susceptible to cefoxitin, cefuroxime sodium, ampicillin, ofloxacin, cephalothin, ciprofloxacin and chloramphenicol; weakly sensitive to amikacin; and resistant to sulphamethoxazole/trimethoprim, piperacillin, ampicillin/sulbactam, amoxicillin/clavulanic acid (2 : 1), tetracycline and meropenem.

To evaluate the anaerobic growth potential of strain 2305UL40-4^T^ under DNRA and denitrification conditions, dissolved metabolites and cell numbers in the medium were measured. During the 3-day incubation period, no significant changes in cell numbers and nitrate levels in the medium were observed, and only a slight increase in ammonium (NH_4_^+^) was detected (Table S3). Genome analysis revealed that genes required for both denitrification and DNRA pathways were absent, indicating that strain 2305UL40-4ᵀ is unable to use nitrate or nitrite as terminal electron acceptors for anaerobic respiration. Instead, the strain appears to reduce nitrate to ammonium through the assimilatory nitrate reduction pathway and using ammonium as the nitrogen source for biosynthesis. The slight increase in cell numbers and ammonium concentration and the decrease in nitrate and nitrite observed shortly after inoculation are likely due to metabolic activity prior to the complete depletion of residual dissolved oxygen in the medium by the reducing agent. These results also explain why the strain exhibited nitrate reduction activity in API tests but cannot use nitrate or nitrite as electron acceptors for anaerobic respiration (Fig. S4).

### Genomic characteristics

**Fig. 2.**
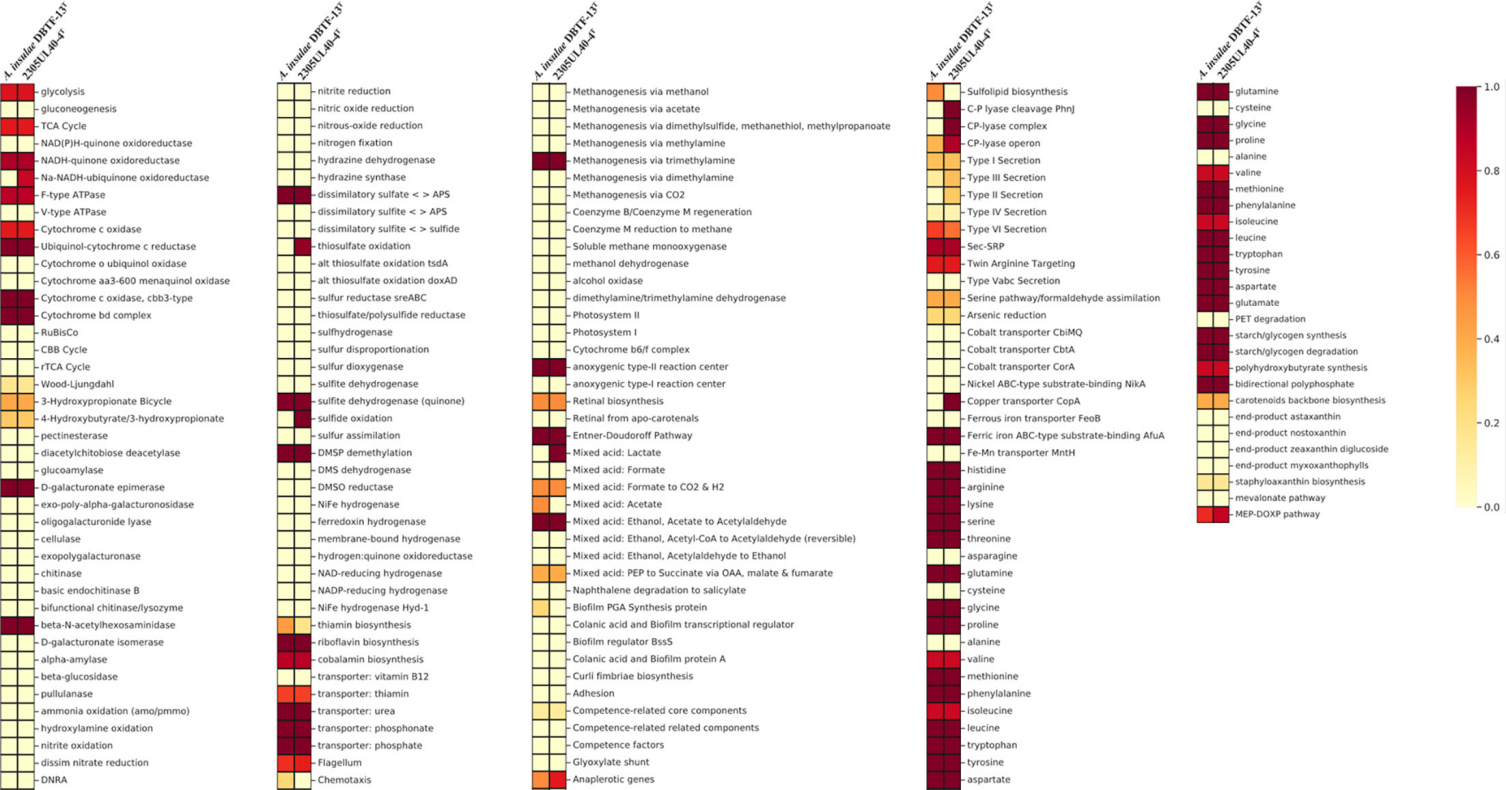
KEGG Orthology-based analysis.

The anti-SMASH analysis identified clusters of secondary metabolites associated with distinct pigmentation in both strains. Both strains contain a terpene (carotenoid) BGC comprising 1-hydroxycarotenoid 3,4-desaturase, carotenoid 1,2-hydratase, 15-cis-phytoene synthase and phytoene desaturase, which are conserved in both strains. However, *A. insulae* KACC 19432ᵀ contains an additional gene encoding spheroidene monooxygenase within the cluster, which is absent in 2305UL40-4ᵀ. The presence of spheroidene monooxygenase is thought to contribute to the more pronounced red pigmentation in *A. insulae* KACC 19432ᵀ compared to 2305UL40-4ᵀ. According to the annotation results based on the KofamKOALA database, strain 2305UL40-4ᵀ has essential biological metabolic pathways such as the citrate cycle (TCA cycle and Krebs cycle), glycolysis, pyruvate oxidation, pentose phosphate pathway, glycogen metabolism, propanoyl-CoA and both oxidative and non-oxidative phases of carbohydrate metabolism. Additionally, strain 2305UL40-4ᵀ has biosynthesis pathways for glycogen, nucleotide sugar and UDP-N-acetyl-d-glucosamine. In energy metabolism, it exhibited crassulacean acid metabolism, NADH in ATP synthesis and enzymes such as succinate dehydrogenase, cytochrome and F-type ATPase. In purine and pyrimidine metabolism, the strain could perform adenine, guanine and pyrimidine ribonucleotide biosynthesis and degradation. Fatty acid metabolism of strain 2305UL40-4ᵀ includes acyl-CoA synthesis and *β*-oxidation pathways, which were identified through genome annotation. A comparison of metabolic subsystems using the KEGG database revealed that both strains shared overall similar metabolic profiles. However, strain 2305UL40-4ᵀ possesses a higher abundance of genes associated with carbohydrate metabolism and genetic information processing (Fig. S4), whereas *A. insulae* KACC 19432ᵀ exhibited more genes involved in the metabolism of cofactors and vitamins. These differences suggest that the symbiotic strain 2305UL40-4ᵀ may efficiently acquire metabolites such as cofactors and vitamins through interactions with its host, whereas the free-living *A. insulae* KACC 19432ᵀ must synthesize these compounds independently. The higher abundance of genetic information processing genes in strain 2305UL40-4ᵀ also implies its greater potential for rapid adaptation to changing environments and diverse metabolic conditions. In addition, BLASTKOALA analysis identified the presence of the thiosulphate oxidation (SOX) pathway exclusively in strain 2305UL40-4ᵀ, further supporting the unique metabolic features of this strain. Conversely, *A. insulae* KACC 19432ᵀ uniquely possesses genes for the thiamine salvage pathway (cofactors and vitamin metabolism), glycine cleavage system (amino acid metabolism) and betaine biosynthesis. Strain 2305UL40-4ᵀ also harbours genes encoding *α*-galactosidase and enzymes involved in the metabolism of d-glucose, l-arabinose, d-mannose and d-mannitol. However, API test results indicated that these enzymes were not functionally expressed under the tested conditions. A notable difference between the two strains was observed in the glycolytic pathway. Only strain 2305UL40-4ᵀ possesses genes encoding l-lactate dehydrogenase [EC:1.1.1.27], pyruvate, water dikinase [EC:2.7.9.2], fructose-bisphosphate aldolase [EC:4.1.2.13], alcohol dehydrogenase [EC:1.1.1.1], 6-phosphofructokinase 2 [EC:2.7.1.11] and gluconolactonase [EC:3.1.1.17], highlighting its broader glycolytic capacity compared to *A. insulae* KACC 19432ᵀ. In particular, with regard to sulphur metabolism, genes of l-cysteine S-thiosulphotransferase [EC:2.8.5.2], S-sulfosulfanyl-l-cysteine sulfohydrolase [EC:3.1.6.20], sulphide dehydrogenase [flavocytochrome c] flavoprotein chain [EC:1.8.2.3], methanethiol oxidase [EC:1.8.3.4] and S-disulfanyl-l-cysteine oxidoreductase SoxD [EC:1.8.2.6] were exclusively detected in strain 2305UL40-4ᵀ and not in *A. insulae* KACC 19432ᵀ. Based on these results, strain 2305UL40-4ᵀ is expected to decompose more diverse organic sulphur compounds and contribute to the carbon cycle than strain KACC 19432ᵀ (Tables S4 and S5). Neither *Aestuariibius* species possesses genes related to the metabolism of specific amino acids such as asparagine, cysteine and alanine. However, a complete gene set for transporters of urea, phosphonate and phosphate was indicated in dark red in Fig. 2(a). This was consistent with the ability of synthesis and degradation of starch/glycogen. For a more detailed heat map, refer to Fig. 2. The proportional values can be found in Fig. S5.

**Fig. 3.**
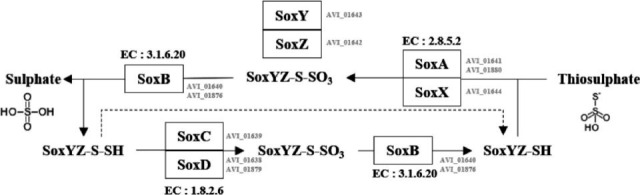
Sox system pathways and the corresponding enzyme EC numbers in strain 2305UL40-4ᵀ.

*C. toreuma*, the host of 2305UL40-4ᵀ, belongs to the gastropods and contains various minerals such as potassium, sodium, selenium, zinc, iron and sulphur [44]. Therefore, it was expected that there was a metabolic process related to sulphur provided by the host. In particular, the presence of all the genes required for the Sox system (Fig. 3) suggests that strain 2305UL40-4ᵀ may contribute to environmental detoxification by oxidizing reduced sulphur compounds such as hydrogen sulphide to sulphate, thereby providing available resources to planktonic marine organisms. Although strain 2305UL40-4ᵀ and *A. insulae* belong to the same genus, each strain exhibits ecological function shaped by its biological host and sedimentary environments, reflecting the influence of their specific isolation sources.

## Protologues

Considering the genomic indices, phylogenetic and phylogenomic analyses, strain 2305UL40-4ᵀ should be classified as a novel species in the genus *Aestuariibius* (Fig. 1). The strain exhibited phenotypic characteristics consistent with previous members of the genus *Aestuariibius*, such as respiratory quinone type and fatty acid composition (Table 1). Subsystem-based genomic analysis further supported its taxonomic distinction from other genera. However, it was clearly differentiated from previously reported species in the genus *Aestuariibius* in terms of its optimal growth conditions, detailed fatty acid profiles, multiple enzyme activities, difference in metabolic genes and carbon source utilization patterns (Table 1, Fig. 2). Based on these results, strain 2305UL40-4ᵀ (=KCCM 43505ᵀ=MCCC 1K09164ᵀ) is proposed as the type strain of the novel species *Aestuariibius violaceus* sp. nov.

## Emended description of the genus *Aestuariibius* Park *et al*. 2018

*Aestuariibius* (Aes.tu.a.ri.i′bi.us. L. neut. n. *aestuarium* -i tidal flat; N.L. masc. n. bius from *Gr. n. bios* life; N.L. masc. n. *Aestuariibius* life in tidal flat).

Description of the genus *Aestuariibius* Park *et al*. 2018 is emended as follows: DNA G+C content ranged 61.6–63.8 mol%. The major fatty acids were summed feature 8 (C_18 : 1_* ω*7*c* and/or C_18 : 1_* ω*6*c*), C_16  : 0_ and C_18 : 0_. The type species is *A. insulae*, and genome sequence analysis indicates that it is affiliated with the family *Roseobacteraceae* in the class *Alphaproteobacteria*.

## Description of *Aestuariibius violaceus* sp. nov.

*Aestuariibius violaceus* (vi.o.la’ce.us. L. masc. adj. *violaceus* violet-coloured)

Cells are Gram-reaction-negative, aerobic, non-motile and non-spore-forming. When cultured on an MA plate at 25 ℃ for 3 days, the cell size was 0.5–0.9 µm×1.5–2.2 µm, and the colonies were magenta colour, punctiform, flat, smooth and with even margins. Growth is observed at 22–36 °C (optimal 25–30 °C), pH 6.0–8.0 (optimal 7.0) and 0.5–4.0% (optimum 2.0%) NaCl. The major fatty acids and quinones are C_16 : 0_, C_18 : 0_ and summed feature 8 (C_18 : 1_* ω*7*c* and/or C_18 : 1_* ω*6*c*) and Q-10, respectively. Polar lipids mainly include phosphatidylethanolamine, phosphatidylglycerol, diphosphatidylglycerol, phosphatidylcholine, five unidentified aminolipids and fifteen unidentified lipids. The DNA G+C content is 63.8 mol%. In the API kit results, the reduction of nitrates to nitrogen is positive, indicating leucine arylamidase and *β*-galactosidase enzyme activities. Alkaline phosphatase, esterase, esterase lipase, valine arylamidase, naphtol-AS-BI-phosphohydrolase, *β*-glucuronidase and *α*-glucosidase exhibit weak activity. Aesculin ferric citrate and d-raffinose can be used as carbon sources, and d-ribose, d-xylose, d-sucrose, inulin, glycogen, d-lyxose, d-tagatose and d-arabitol can be weakly used as carbon sources.

The type strain, 2305UL40-4^T^ (=KCCM 43505^T^=MCCC 1K09164^T^), was isolated from *C. toreuma* collected at Ulleungdo, Republic of Korea. The deposition numbers of the 16S rRNA gene and whole genome registered in NCBI/EMBL/DDBJ are OR945532 and JBFONT000000000, respectively.

## Supplementary material

10.1099/ijsem.0.006834 Uncited Supplementary Material 1.

## Abbreviations

AAI: amino acid identity
ANI: average nucleotide identity
BGCs: biosynthetic gene clusters
*d*DDH: digital DNA–DNA hybridization
DNRA: dissimilatory nitrate reduction
Q-10: ubiquinone-10
TEM: transmission electron microscopy
