# Supplementary material for: Aestuariibius violaceus sp. nov., isolated from a marine limpet Cellana toreuma
Source: Int J Syst Evol Microbiol. 2025 Jul 8;75(7):006834. doi: 10.1099/ijsem.0.006834 (PMC12282053; doi:10.1099/ijsem.0.006834)
Supplement: Uncited Supplementary Material 1. [file ijsem-75-06834-s001.pdf]

Supplementary materials for : INTERNATIONAL JOURNAL OF SYSTEMATIC AND EVOLUTIONARY MICROBIOLOGY

Aestuariibius violaceus sp. nov., isolated from a marine limpet *Cellana toreuma*

Min Seo Lee [1, 2], Mi-Jeong Park[1], Purena Son[3], Taekeun Rho[3], Kae Kyoung Kwon[1,4]*, Jin-Sook Park[2]*

[1]Marine Biotechnology Research Center, Korea Institute of Ocean Science & Technology, 385, Haeyang- ro, Yeongdo-gu, Busan, 49111, Republic of Korea

[2]Department of Biological Sciences and Biotechnology, Hannam University, Daejeon 34054, Republic of Korea

[3]Marine Environment Research Department, Korea Institute of Ocean Science & Technology, 385, Haeyang- ro, Yeongdo-gu, Busan, 49111, Republic of Korea

[4]KIOST School, University of Science and Technology, Daejeon 34113, Republic of Korea

Running title; *Aestuariibius violaceus* sp. nov.,

Subject category; **Novel Taxa (*Pseudomonadota*)**

The GenBank/EMBL/DDBJ accession numbers for the 16S rRNA gene and whole genome sequences of strains 2305UL40-4^T are OR945532 and JBFONT000000000, respectively. *Aestuariibius insulae* deposition numbers of the whole genome registered in GenBank/EMBL/DDBJ is JBFONW000000000.

***Corresponding author:** Kae Kyoung Kwon Phone: +82 51 664 3371. E-mail: kkkwon@kiost.ac.kr

***Corresponding author:** Jin-Sook Park: Phone : +82 42 629 8775. E-mail: jspark@hnu.kr

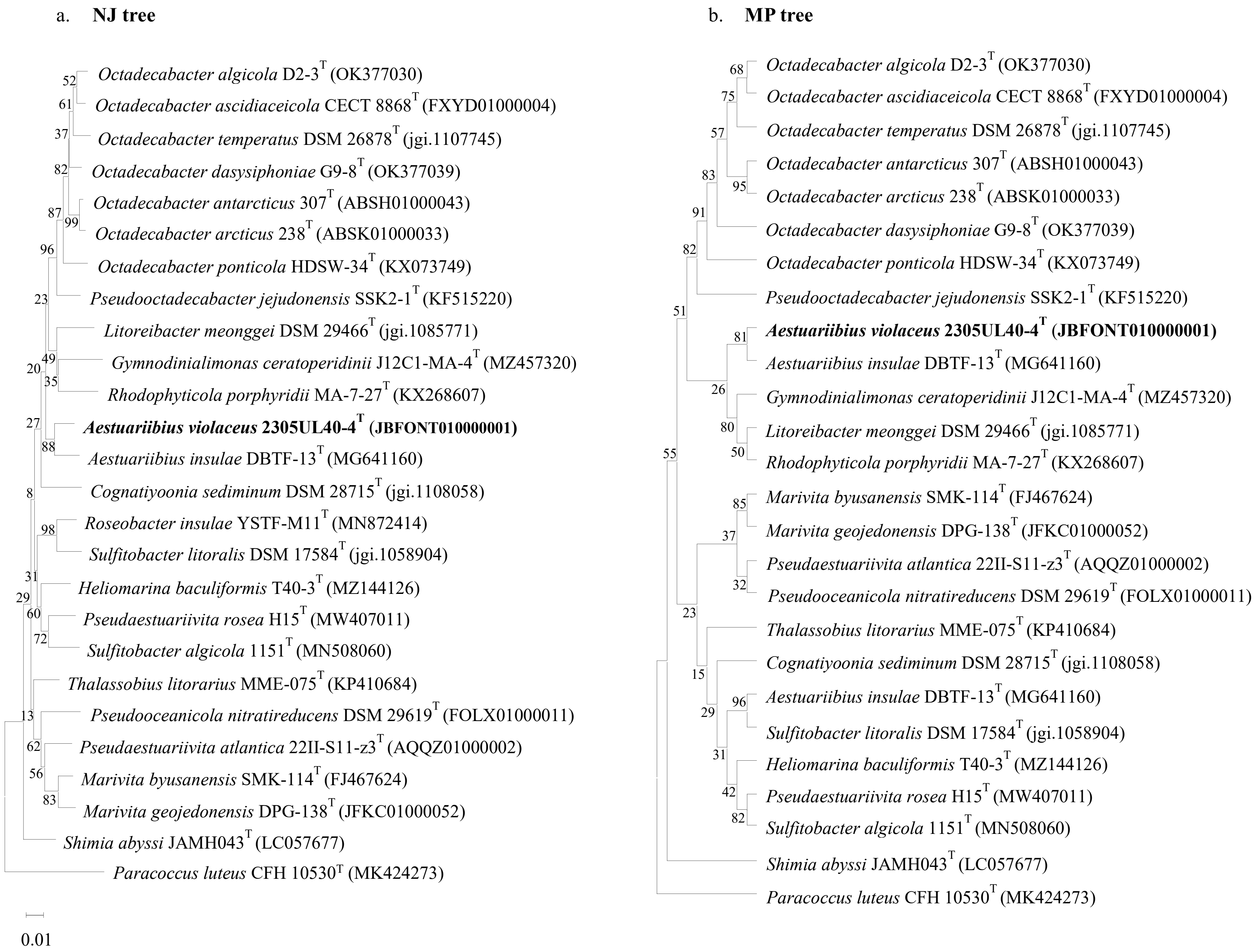

Supplementary Fig. S1. Phylogenetic tree of strain 2305UL40-4^{T} and closely related species based on nearly complete 16S rRNA gene sequence. Numbers at the nodes indicate percentages of replicate trees in which the associated taxa clustered together in toe bootstrap test. (1000 replicates) **a.** Neighbor-joining phylogenetic tree. **b.** Maximum-parsimony phylogenetic tree. **c.** Maximum-likelihood phylogenetic tree. **d.** Nodes with MP and ML values from NJ phylogeny. All nodes were recovered with 90% < (●), with 70% < (○), or one method with lower than 70% (▲) Bootstrap values by NJ, ML, and MP methods.

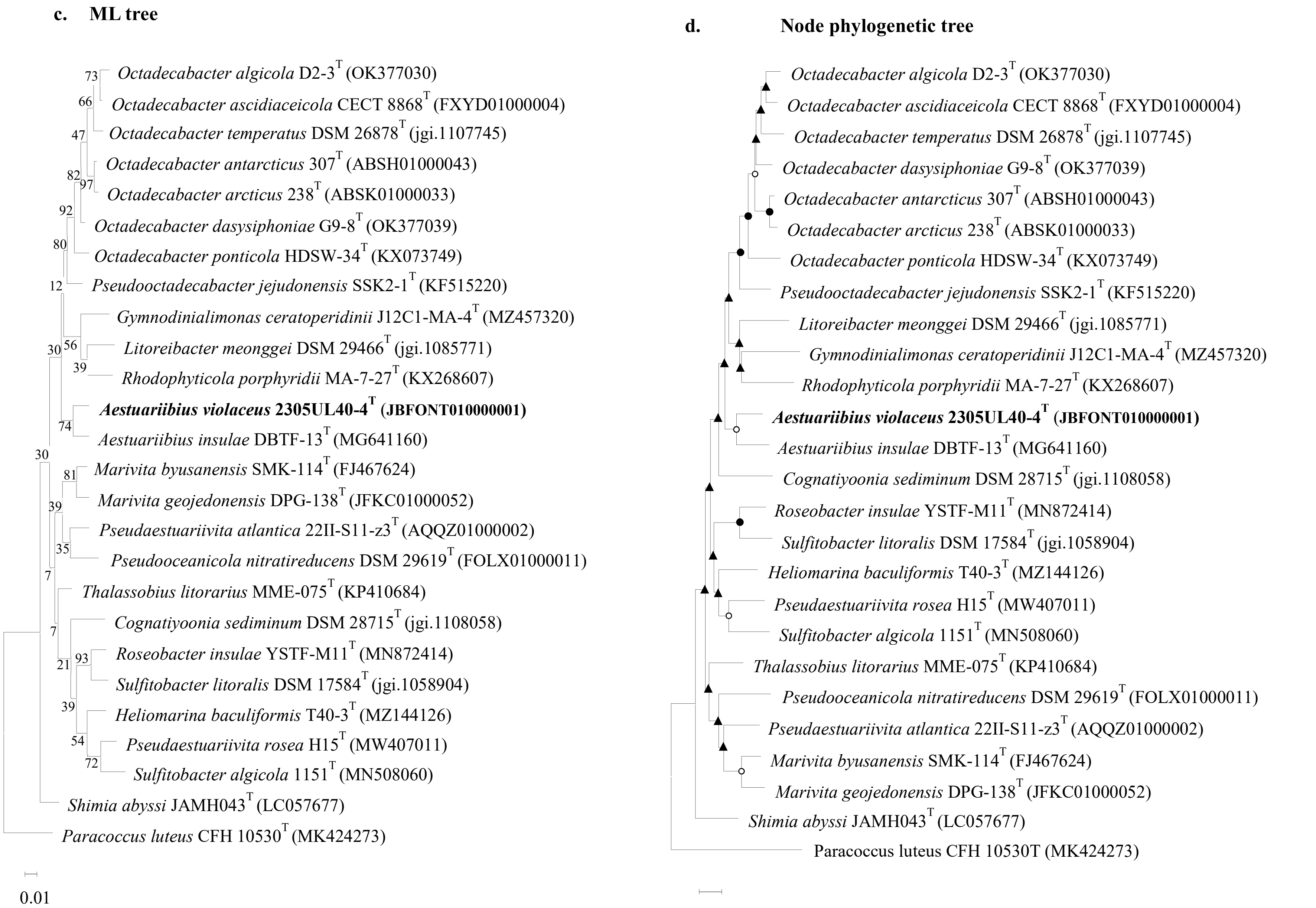

Fig S1. continued

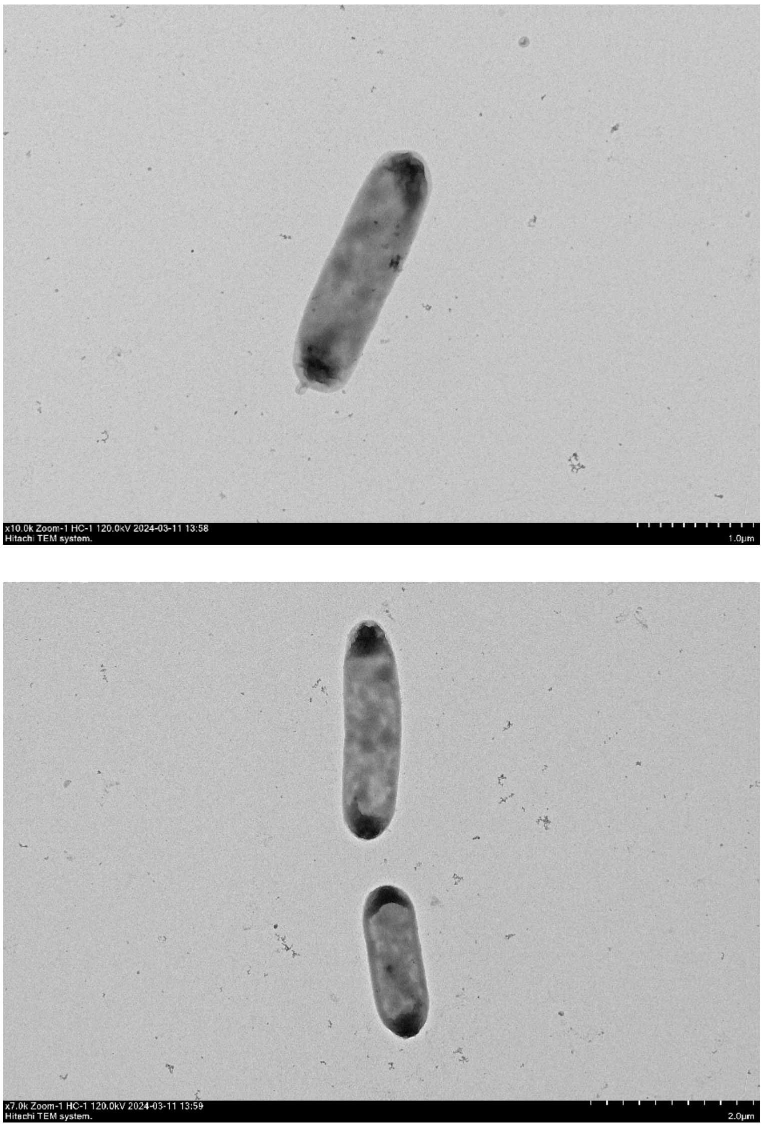

Supplementary Fig. S2. Transmission electron micrographs of negatively stained cells of *Aestuariibius violaceus* 2305UL40-4^T from cells cultivated for 3 days at 25 °C on Marine Agar 2216.

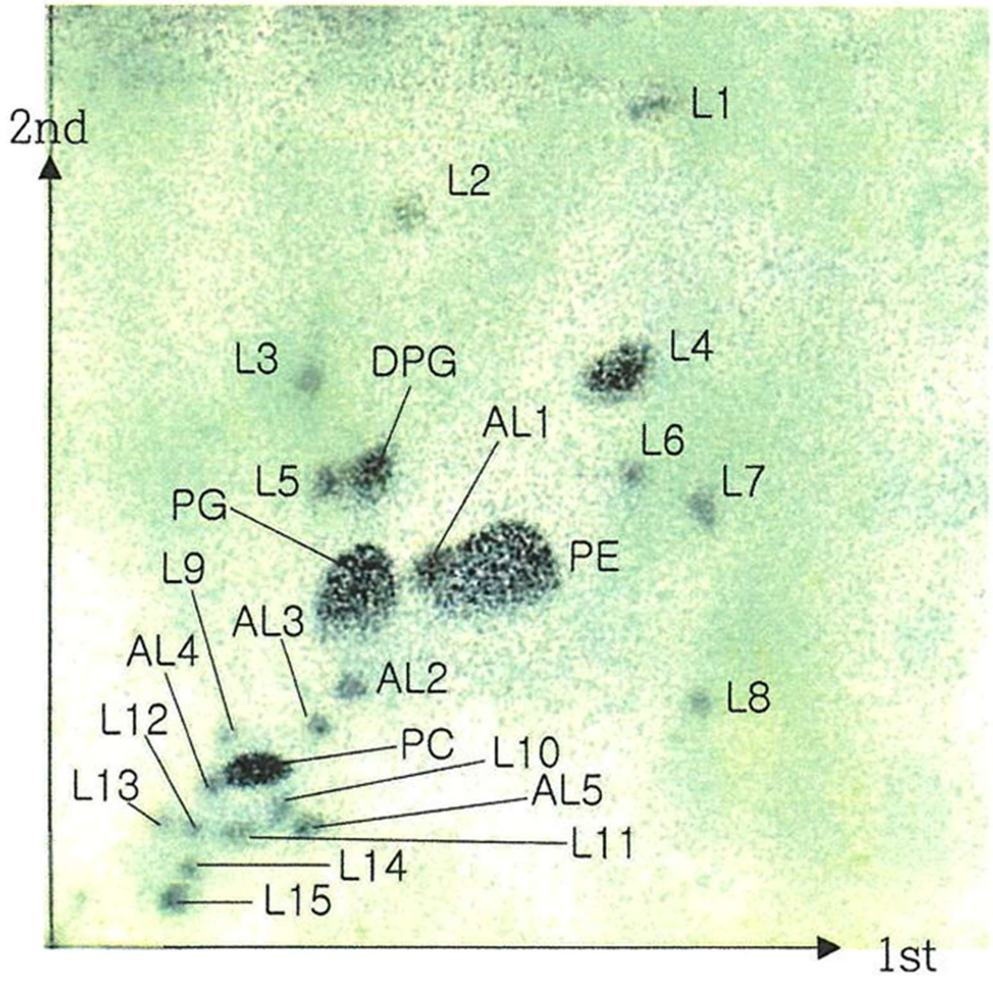

Supplementary Fig. S3. The total polar lipids of *Aestuariibius violaceus* 2305UL40-4^T confirmed through chromatography. PE; phosphatidylethanolamine, PG; phosphatidylglycerol, DPG; diphosphatidylglycerol, PC; phosphatidylcholine, AL1–5; unidentified aminolipids, L1–15; unidentified lipids

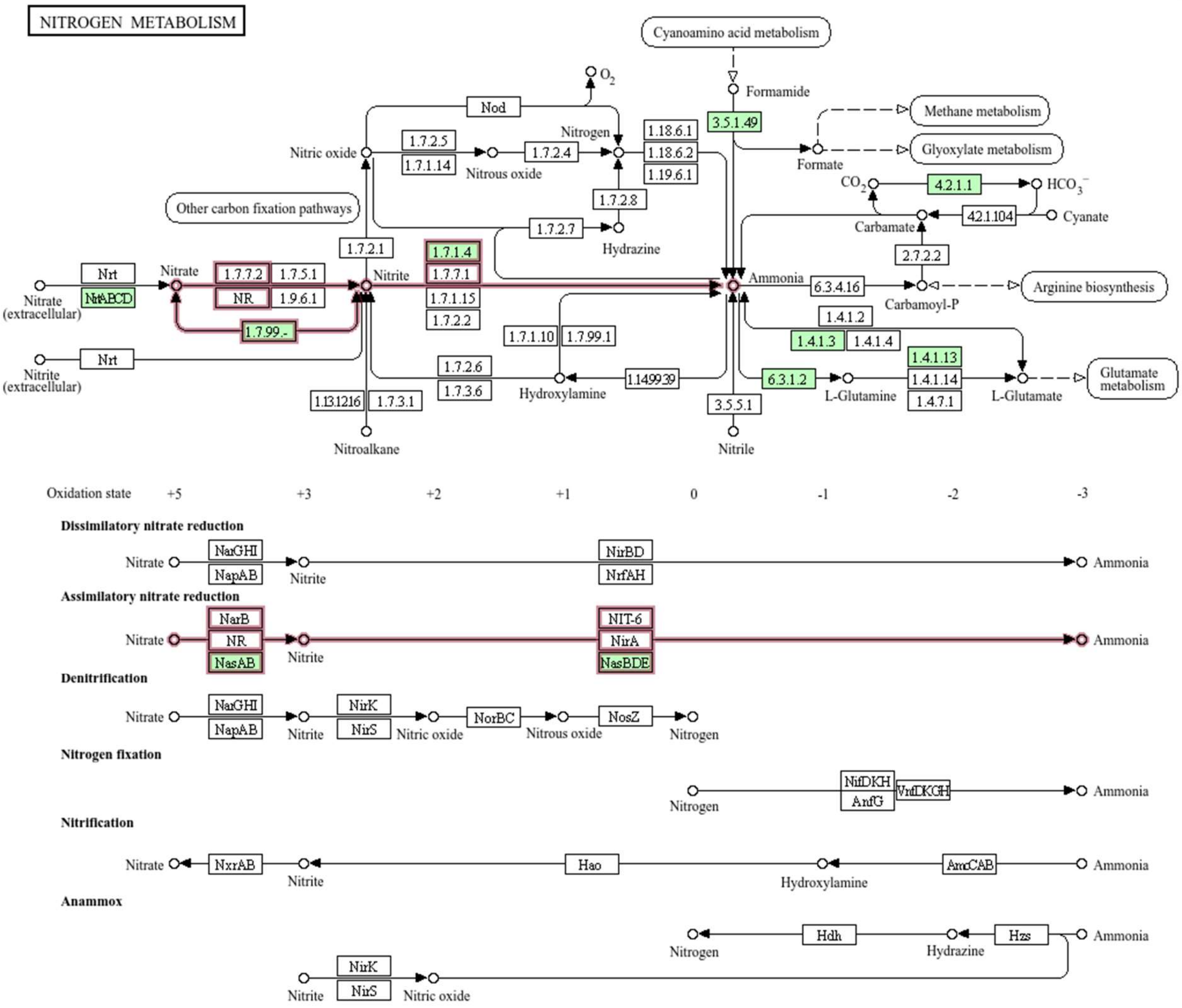

Supplementary Fig. S3. Nitrogen metabolism pathway based on KEGG pathway database. Metabolic pathways were conserved in *Aestuariibius* spp.

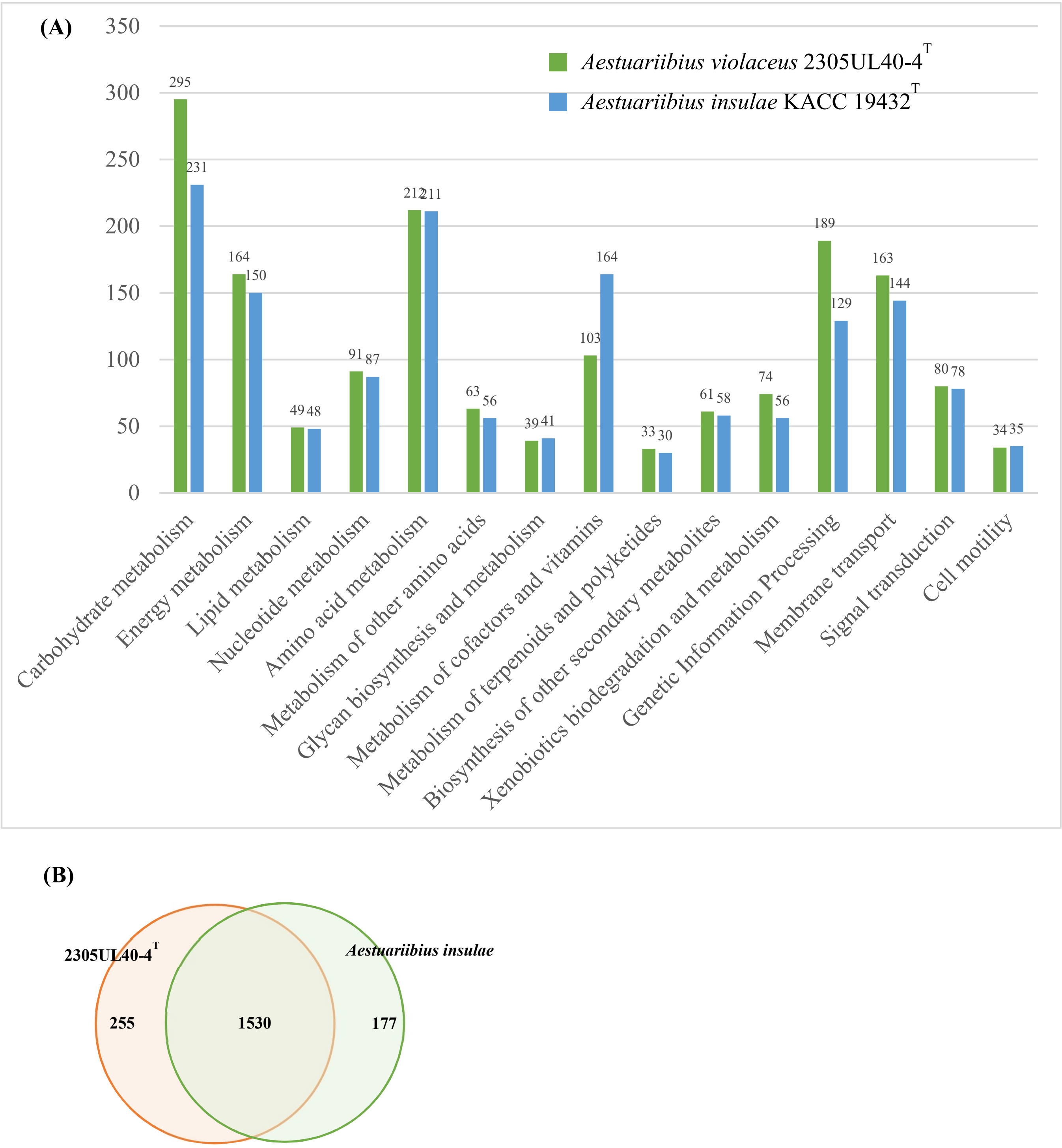

Supplementary Fig. S4. **(A)** Comparison of subsystem coverage and categories between the two strains as described by KEGG metabolism. **(B)** Venn diagram based on KEGG ID.

Table S1. Genome information of the strains used to create a whole genome-based amino acid phylogenetic tree.

Genus	Species	Strain	Isolation source	Accession number	No. contigs	N50 (kb)	CDS	rRNA	tRNA	tmRNA	CRISPR repeat region	G+C content (mol%)	Size (Mb)
Aestuariibius	*violaceus*	2305UL4-04T	Cellana toreuma	GCF_040779325.1	43	350	4398	3	52	1		63.7	4.42
Aestuariibius	*insulae*	DBTF-13T	sediment	GCF_040783535.1	13	567	3606	3	44	1		61.4	3.71
Cognatiyoonia	*sediminum*	S3B03T	sediment	GCA_900129845.1	16	1637	3279	3	39	1		54.4	3.26
Gymnodinialimonas	*ceratoperidinii*	J12C1-MA-4T	seawater	GCA_019297855.1	1	3622	3454	3	42	1		64.5	3.62
Heliomarina	*baculiformis*	T40-3T	seawater	GCA_019966545.1	36	472	5010	3	46	1		60.1	5.33
Litoreibacter	*meonggei*	MA1-1T	Halocynthia roretzi	GCA_003663885.1	16	705	3906	3	42	1		57.5	3.95
Marivita	*geojedonensis*	DPG-138T	seawater	GCA_002115805.1	73	125	4215	3	43	1		60	4.31
Octadecabacter	*algicola*	D2-3T	marine red alga	GCA_021532625.1	14	868	3419	3	45	1		54.4	3.45
Octadecabacter	*antarcticus*	307T	polar sea ice	GCA_000155675.2	2	4813	5287	6	43	1		54.6	4.88
Octadecabacter	*arcticus*	238T	sea ice and water	GCA_000155735.2	3	5200	6038	6	42	1		55.1	5.48
Octadecabacter	*ascidiaceicola*	RA1-3T	Halocynthia roretzi	GCA_900185015.1	11	677	3209	3	39	1		54.9	3.23
Octadecabacter	*dasysiphoniae*	G9-8T	marine red alga	GCA_021532615.1	39	1075	3684	3	41	1		56.9	3.76
Octadecabacter	*temperatus*	SB1T	surface water	GCA_001187845.1	2	3233	3294	3	39	1		54.7	3.26
Paracoccus	*luteus*	CFH 10530T	the intestine of grass carp	GCA_004522155.1	48	312	3132	3	53	1	3	69.6	3.33
Pseudaestuariivita	*atlantica*	22II-S11-z3T	sediment	GCA_001205715.1	56	542	4220	3	46	1		65.5	4.34
Pseudaestuariivita	*rosea*	H15T	marine mollusk	GCA_017592335.1	87	115	3925	3	41	1		56.1	3.89
Pseudaestuariivita	*nitratireducens*	DSM 29619T	seawater	GCA_900112545.1	11	3458	3834	3	44	1		64.2	4.07
Pseudooctadecabacter	*jejudonensis*	SSK2-1T	seawater	GCA_900172275.1	15	827	3345	3	43	1		59.6	3.41
Rhodophyticola	*porphyridii*	MA-7-27T	red alga	GCA_003688285.1	22	338	3738	3	42	1		63.4	3.84
Roseobacter	*insulae*	YSTF-M11T	tidal flats	GCA_019375555.1	17	574	4781	3	46	1		60.3	4.88
Shimia	*abyssi*	JAMH 043T	sediment	GCA_003014475.1	48	191	4522	3	43	1		56.2	4.73
Sulfitobacter	*algicola*	1151T	green algae	GCA_013315265.1	70	224	4014	3	38	1		51.8	3.97
Sulfitobacter	*litoralis*	Iso 3T	marine environment	GCA_900103185.1	63	141	3584	4	44	1		58.5	3.68

Table S2. Cellular fatty acid composition (%) of strain (1) 2305UL40-4^T and the type strain of (2) *Aestuariibius insulae* KACC 19432^T.

TR, Trace (<0.5 %). –, Not detected.

Fatty acid	1	2
**Saturated**		
$C_{12:0}$	0.5	–
$C_{14:0}$	TR	–
$C_{16:0}$	13.9	10
$C_{17:0}$	0.4	1.5
$C_{18:0}$	5.6	9.4
**Branched-chain**		
iso-$C_{15:0}$	1.1	–
iso-$C_{15:0}$	–	TR
anteiso-$C_{15:0}$	2	–
iso-$C_{16:0}$	0.7	–
anteiso-$C_{17:0}$	0.7	–
**Hydroxy**		
$C_{10:0}$ 3-OH	4.4	3.3
$C_{15:0}$ 2-OH	TR	–
$C_{16:0}$ 3-OH	0.6	–
cyclo $C_{19:0}$ $\omega 8c$	2.7	1.7
11-methyl $C_{18:1}$ $\omega 7c$	2	8.3
**summed feature***		
7	0.7	–
8	62.4	64.2

*Summed features represent groups of two or three fatty acids that could not be separated using the MIDI system. Summed feature 3 comprises $C_{16:1}$ $\omega 7c$ and/or $C_{16:1}$ $\omega 6c$; summed features 7 comprises unknown fatty acid 18.846 (ECL), $C_{19:1}$ $\omega 6c$ and/or cyclo $C_{19:0}$ $\omega 10c$; summed feature 8 comprises $C_{18:1}$ $\omega 7c$ and/or $C_{18:1}$ $\omega 6c$.

Table S3. Assessment of the denitrification capacity of strain 2305UL40-4^T, showing dissolved nitrate and nitrite concentrations at 24-hour intervals from 0 to 96 hours. The blank represents a medium without potassium nitrate supplementation. Measurement of bacterial cell density for comparison with denitrification capacity.

Sample	Ammonium (mM)	Nitrite (μM)	Nitrate (mM)	Cell number (cells/ml)
0 h	2.57	0.76	98.63	10^5
24 h	3.06	0.45	97.46	$(3.20 \pm 0.36) \times 10^5$
48 h	2.98	0.43	97.92	$(9.34 \pm 2.21) \times 10^5$
72 h	3.03	0.45	96.67	$(6.72 \pm 4.40) \times 10^5$

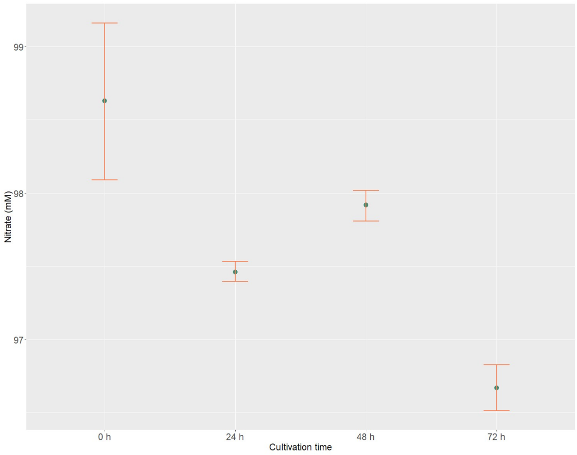

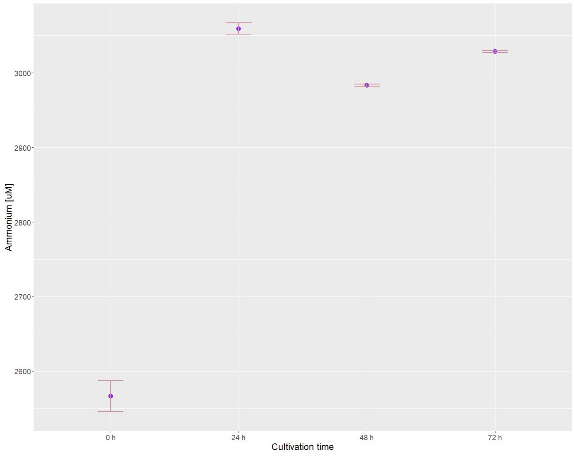

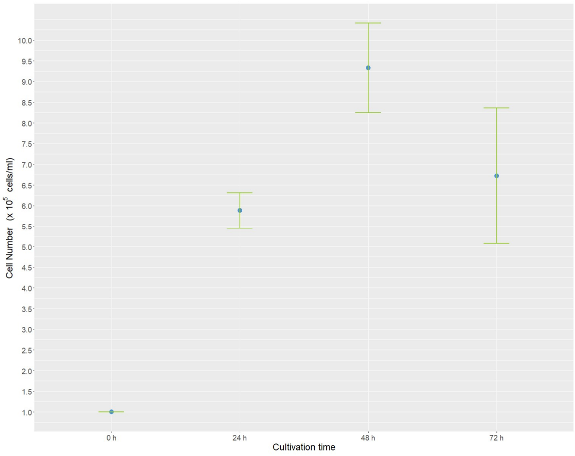

Table S4. KEGG analysis results for the genome of strain 2305UL40-4^T.

AVI_00001	K03702	uvrB; excinuclease ABC subunit B
AVI_00007	K09981	K09981; uncharacterized protein
AVI_00011	K03303	lutP, lctP; lactate permease
AVI_00014	K02030	ABC.PA.S; polar amino acid transport system substrate-binding protein
AVI_00015	K01897	ACSL, fadD; long-chain acyl-CoA synthetase [EC:6.2.1.3]
AVI_00016	K03453	TC.BASS; bile acid:Na+ symporter, BASS family
AVI_00017	K01077	E3.1.3.1, phoA, phoB; alkaline phosphatase [EC:3.1.3.1]
AVI_00021	K13623	btaB; S-adenosylmethionine-diacylgycerolhomoserine-N-methlytransferase
AVI_00022	K13622	btaA; S-adenosylmethionine-diacylglycerol 3-amino-3-carboxypropyl transferase
AVI_00025	K07001	K07001; NTE family protein
AVI_00033	K13483	yagT; xanthine dehydrogenase YagT iron-sulfur-binding subunit
AVI_00034	K11178	yagS; xanthine dehydrogenase YagS FAD-binding subunit [EC:1.17.1.4]
AVI_00035	K11177	yagR; xanthine dehydrogenase YagR molybdenum-binding subunit [EC:1.17.1.4]
AVI_00036	K00568	ubiG; 2-polyprenyl-6-hydroxyphenyl methylase / 3-demethylubiquinone-9 3-methyltransferase [EC:2.1.1.222 2.1.1.64]
AVI_00041	K01284	dcp; peptidyl-dipeptidase Dcp [EC:3.4.15.5]
AVI_00044	K03060	rpoZ; DNA-directed RNA polymerase subunit omega [EC:2.7.7.6]
AVI_00045	K01139	spoT; GTP diphosphokinase / guanosine-3',5'-bis(diphosphate) 3'-diphosphatase [EC:2.7.6.5 3.1.7.2]
AVI_00046	K09928	K09928; uncharacterized protein
AVI_00047	K03474	pdxJ; pyridoxine 5-phosphate synthase [EC:2.6.99.2]
AVI_00048	K00024	mdh; malate dehydrogenase [EC:1.1.1.37]
AVI_00051	K01903	sucC; succinyl-CoA synthetase beta subunit [EC:6.2.1.5]
AVI_00055	K01902	sucD; succinyl-CoA synthetase alpha subunit [EC:6.2.1.5]
AVI_00059	K00164	OGDH, sucA; 2-oxoglutarate dehydrogenase E1 component [EC:1.2.4.2]
AVI_00060	K00658	DLST, sucB; 2-oxoglutarate dehydrogenase E2 component (dihydrolipoamide succinyltransferase) [EC:2.3.1.61]
AVI_00062	K06893	K06893; uncharacterized protein
AVI_00066	K00382	DLD, lpd, pdhD; dihydrolipoyl dehydrogenase [EC:1.8.1.4]
AVI_00067	K02613	paaE; ring-1,2-phenylacetyl-CoA epoxidase subunit PaaE
AVI_00068	K02612	paaD; ring-1,2-phenylacetyl-CoA epoxidase subunit PaaD
AVI_00069	K02611	paaC; ring-1,2-phenylacetyl-CoA epoxidase subunit PaaC [EC:1.14.13.149]
AVI_00070	K02610	paaB; ring-1,2-phenylacetyl-CoA epoxidase subunit PaaB
AVI_00071	K02609	paaA; ring-1,2-phenylacetyl-CoA epoxidase subunit PaaA [EC:1.14.13.149]
AVI_00073	K02618	paaZ; oxepin-CoA hydrolase / 3-oxo-5,6-dehydrosuberyl-CoA semialdehyde dehydrogenase [EC:3.3.2.12 1.2.1.91]
AVI_00074	K15866	paaG; 2-(1,2-epoxy-1,2-dihydrophenyl)acetyl-CoA isomerase [EC:5.3.3.18]
AVI_00075	K02614	paaI; acyl-CoA thioesterase [EC:3.1.2.-]
AVI_00076	K01912	paaK; phenylacetate-CoA ligase [EC:6.2.1.30]
AVI_00084	K01990	ABC-2.A; ABC-2 type transport system ATP-binding protein
AVI_00085	K08738	CYC; cytochrome c
AVI_00091	K09781	K09781; uncharacterized protein
AVI_00092	K04518	pheA2; prephenate dehydratase [EC:4.2.1.51]
AVI_00093	K08738	CYC; cytochrome c
AVI_00094	K13893	yejA; microcin C transport system substrate-binding protein

AVI_00095	K13894	yejB; microcin C transport system permease protein
AVI_00096	K13895	yejE; microcin C transport system permease protein
AVI_00097	K13896	yejF; microcin C transport system ATP-binding protein
AVI_00098	K17838	oxa; beta-lactamase class D [EC:3.5.2.6]
AVI_00102	K01119	cpdB; 2',3'-cyclic-nucleotide 2'-phosphodiesterase / 3'-nucleotidase [EC:3.1.4.16 3.1.3.6]
AVI_00103	K03426	NUDT12_13, nudC; NAD+ diphosphatase [EC:3.6.1.22]
AVI_00107	K07127	uraH, pucM, hiuH; 5-hydroxyisourate hydrolase [EC:3.5.2.17]
AVI_00108	K16842	hpxB; allantoinase [EC:3.5.2.5]
AVI_00109	K14977	ylbA, UGHY; (S)-ureidoglycine aminohydrolase [EC:3.5.3.26]
AVI_00110	K01483	allA; ureidoglycolate lyase [EC:4.3.2.3]
AVI_00111	K06889	K06889; uncharacterized protein
AVI_00112	K00425	cydA; cytochrome bd ubiquinol oxidase subunit I [EC:7.1.1.7]
AVI_00113	K00426	cydB; cytochrome bd ubiquinol oxidase subunit II [EC:7.1.1.7]
AVI_00114	K14733	limB; limonene 1,2-monooxygenase [EC:1.14.13.107]
AVI_00116	K01749	hemC, HMBS; hydroxymethylbilane synthase [EC:2.5.1.61]
AVI_00117	K01599	hemE, UROD; uroporphyrinogen decarboxylase [EC:4.1.1.37]
AVI_00118	K00228	CPOX, hemF; coproporphyrinogen III oxidase [EC:1.3.3.3]
AVI_00120	K00076	hdhA; 7-alpha-hydroxysteroid dehydrogenase [EC:1.1.1.159]
AVI_00121	K00564	rsmC; 16S rRNA (guanine1207-N2)-methyltransferase [EC:2.1.1.172]
AVI_00122	K06891	clpS; ATP-dependent Clp protease adaptor protein ClpS
AVI_00123	K07025	K07025; putative hydrolase of the HAD superfamily
AVI_00124	K01286	E3.4.16.4; D-alanyl-D-alanine carboxypeptidase [EC:3.4.16.4]
AVI_00129	K02343	dnaX; DNA polymerase III subunit gamma/tau [EC:2.7.7.7]
AVI_00130	K09747	ebfC; nucleoid-associated protein EbfC
AVI_00131	K06187	recR; recombination protein RecR
AVI_00132	K02005	ABC.CD.TX; HlyD family secretion protein
AVI_00133	K02004	ABC.CD.P; putative ABC transport system permease protein
AVI_00134	K02003	ABC.CD.A; putative ABC transport system ATP-binding protein
AVI_00135	K09987	K09987; uncharacterized protein
AVI_00136	K01166	RNASET2; ribonuclease T2 [EC:4.6.1.19]
AVI_00138	K03088	rpoE; RNA polymerase sigma-70 factor, ECF subfamily
AVI_00142	K18587	COQ9; ubiquinone biosynthesis protein COQ9
AVI_00143	K02970	RP-S21, MRPS21, rpsU; small subunit ribosomal protein S21
AVI_00144	K03282	mscL; large conductance mechanosensitive channel
AVI_00145	K11209	yghU, yfcG; GSH-dependent disulfide-bond oxidoreductase [EC:1.8.4.-]
AVI_00147	K01890	FARSB, pheT; phenylalanyl-tRNA synthetase beta chain [EC:6.1.1.20]
AVI_00149	K01889	FARSA, pheS; phenylalanyl-tRNA synthetase alpha chain [EC:6.1.1.20]
AVI_00151	K02887	RP-L20, MRPL20, rplT; large subunit ribosomal protein L20
AVI_00152	K02916	RP-L35, MRPL35, rpmI; large subunit ribosomal protein L35
AVI_00154	K00873	PK, pyk; pyruvate kinase [EC:2.7.1.40]
AVI_00156	K09948	K09948; uncharacterized protein
AVI_00159	K12573	rnr, vacB; ribonuclease R [EC:3.1.13.1]

AVI_00160	K01439	dapE; succinyl-diaminopimelate desuccinylase [EC:3.5.1.18]
AVI_00164	K00674	dapD; 2,3,4,5-tetrahydropyridine-2,6-dicarboxylate N-succinyltransferase [EC:2.3.1.117]
AVI_00167	K06941	rlmN; 23S rRNA (adenine2503-C2)-methyltransferase [EC:2.1.1.192]
AVI_00168	K20035	dmdC; 3-(methylsulfanyl)propanoyl-CoA dehydrogenase [EC:1.3.99.41]
AVI_00169	K07566	tsaC, rimN, SUA5, YRDC; L-threonylcarbamoyladenylate synthase [EC:2.7.7.87]
AVI_00171	K03566	gcvA; LysR family transcriptional regulator, glycine cleavage system transcriptional activator
AVI_00175	K02523	ispB; octaprenyl-diphosphate synthase [EC:2.5.1.90]
AVI_00176	K00919	ispE; 4-diphosphocytidyl-2-C-methyl-D-erythritol kinase [EC:2.7.1.148]
AVI_00178	K00311	ETFDH; electron-transferring-flavoprotein dehydrogenase [EC:1.5.5.1]
AVI_00179	K03624	greA; transcription elongation factor GreA
AVI_00182	K03750	moeA; molybdopterin molybdotransferase [EC:2.10.1.1]
AVI_00183	K03753	mobB; molybdopterin-guanine dinucleotide biosynthesis adapter protein
AVI_00184	K03752	mobA; molybdenum cofactor guanylyltransferase [EC:2.7.7.77]
AVI_00185	K02379	fdhD; FdhD protein
AVI_00186	K26605	azlC, brnF; branched chain amino acid efflux pump
AVI_00187	K26606	azlD, brnE; branched chain amino acid efflux pump
AVI_00191	K00626	ACAT, atoB; acetyl-CoA C-acetyltransferase [EC:2.3.1.9]
AVI_00193	K01703	leuC, IPMI-L; 3-isopropylmalate/(R)-2-methylmalate dehydratase large subunit [EC:4.2.1.33 4.2.1.35]
AVI_00195	K01704	leuD, IPMI-S; 3-isopropylmalate/(R)-2-methylmalate dehydratase small subunit [EC:4.2.1.33 4.2.1.35]
AVI_00198	K00052	leuB, IMDH; 3-isopropylmalate dehydrogenase [EC:1.1.1.85]
AVI_00201	K16703	wcaL, amsK, cpsK; colanic acid/amylovoran/stewartan biosynthesis glycosyltransferase WcaL/AmsK/CpsK [EC:2.4.-.-]
AVI_00204	K03328	TC.PST; polysaccharide transporter, PST family
AVI_00206	K16564	exoU; succinoglycan biosynthesis protein ExoU [EC:2.4.-.-]
AVI_00207	K16555	exoO; succinoglycan biosynthesis protein ExoO [EC:2.4.-.-]
AVI_00209	K16552	exoF; polysaccharide biosynthesis/export protein ExoF
AVI_00210	K16554	exoP, vpsO; polysaccharide biosynthesis transport protein [EC:2.7.10.3]
AVI_00211	K16567	exoQ; exopolysaccharide production protein ExoQ
AVI_00212	K16559	exoK; endo-1,3-1,4-beta-glycanase ExoK [EC:3.2.1.-]
AVI_00215	K01179	E3.2.1.4; endoglucanase [EC:3.2.1.4]
AVI_00216	K03296	TC.HAE1; hydrophobic/amphiphilic exporter-1 (mainly G- bacteria), HAE1 family
AVI_00217	K03585	acrA, mexA, adeI, smeD, mtrC, cmeA; membrane fusion protein, multidrug efflux system
AVI_00218	K09794	K09794; uncharacterized protein
AVI_00219	K03704	cspA; cold shock protein
AVI_00221	K03704	cspA; cold shock protein
AVI_00224	K10804	tesA; acyl-CoA thioesterase I [EC:3.1.2.- 3.1.2.2 3.1.1.2 3.1.1.5]
AVI_00225	K02003	ABC.CD.A; putative ABC transport system ATP-binding protein
AVI_00226	K02004	ABC.CD.P; putative ABC transport system permease protein
AVI_00227	K01187	malZ; alpha-glucosidase [EC:3.2.1.20]
AVI_00229	K21029	moeB; molybdopterin-synthase adenylyltransferase [EC:2.7.7.80]
AVI_00234	K00220	tyrC; cyclohexadieny/prephenate dehydrogenase [EC:1.3.1.43 1.3.1.12]
AVI_00235	K00817	hisC; histidinol-phosphate aminotransferase [EC:2.6.1.9]
AVI_00236	K02986	RP-S4, NAM9, rpsD; small subunit ribosomal protein S4

AVI_00237	K00639	kbl, GCAT; glycine C-acetyltransferase [EC:2.3.1.29]
AVI_00238	K00060	tdh; threonine 3-dehydrogenase [EC:1.1.1.103]
AVI_00239	K00525	E1.17.4.1A, nrdA, nrdE; ribonucleoside-diphosphate reductase alpha chain [EC:1.17.4.1]
AVI_00241	K00765	hisG; ATP phosphoribosyltransferase [EC:2.4.2.17]
AVI_00242	K02502	hisZ; ATP phosphoribosyltransferase regulatory subunit
AVI_00243	K01892	HARS, hisS; histidyl-tRNA synthetase [EC:6.1.1.21]
AVI_00247	K00500	phhA, PAH; phenylalanine-4-hydroxylase [EC:1.14.16.1]
AVI_00248	K03745	slyX; SlyX protein
AVI_00254	K02337	dnaE; DNA polymerase III subunit alpha [EC:2.7.7.7]
AVI_00262	K13481	xdhA; xanthine dehydrogenase small subunit [EC:1.17.1.4]
AVI_00263	K13482	xdhB; xanthine dehydrogenase large subunit [EC:1.17.1.4]
AVI_00264	K07402	xdhC; xanthine dehydrogenase accessory factor
AVI_00265	K23537	nupA; general nucleoside transport system ATP-binding protein
AVI_00267	K23535	nupB; general nucleoside transport system permease protein
AVI_00269	K23536	nupC; general nucleoside transport system permease protein
AVI_00270	K07335	bmpA, bmpB, tmpC; basic membrane protein A and related proteins
AVI_00272	K19746	dauA; D-arginine dehydrogenase [EC:1.4.99.6]
AVI_00274	K00315	DMGDH; dimethylglycine dehydrogenase [EC:1.5.8.4]
AVI_00279	K01918	panC; pantoate--beta-alanine ligase [EC:6.3.2.1]
AVI_00280	K00606	panB; 3-methyl-2-oxobutanoate hydroxymethyltransferase [EC:2.1.2.11]
AVI_00285	K00015	gyaR, GOR1; glyoxylate reductase [EC:1.1.1.26]
AVI_00292	K00950	folK; 2-amino-4-hydroxy-6-hydroxymethyldihydropteridine diphosphokinase [EC:2.7.6.3]
AVI_00294	K01520	dut, DUT; dUTP diphosphatase [EC:3.6.1.23]
AVI_00295	K13038	coaBC, dfp; phosphopantothenoylcysteine decarboxylase / phosphopantothenate---cysteine ligase [EC:4.1.1.36 6.3.2.5]
AVI_00298	K01867	WARS, trpS; tryptophanyl-tRNA synthetase [EC:6.1.1.2]
AVI_00300	K03980	murJ, mviN; putative peptidoglycan lipid II flippase
AVI_00301	K00990	glnD; [protein-PII] uridylyltransferase [EC:2.7.7.59]
AVI_00303	K07056	rsmI; 16S rRNA (cytidine1402-2'-O)-methyltransferase [EC:2.1.1.198]
AVI_00304	K07460	yraN; putative endonuclease
AVI_00305	K01920	gshB; glutathione synthase [EC:6.3.2.3]
AVI_00306	K07391	comM; magnesium chelatase family protein
AVI_00307	K00799	GST, gst; glutathione S-transferase [EC:2.5.1.18]
AVI_00309	K02231	cobP, cobU; adenosylcobinamide kinase / adenosylcobinamide-phosphate guanylyltransferase [EC:2.7.1.156 2.7.7.62]
AVI_00310	K03089	rpoH; RNA polymerase sigma-32 factor
AVI_00313	K14742	tsaB; tRNA threonylcarbamoyladenosine biosynthesis protein TsaB
AVI_00314	K03789	rimI; [ribosomal protein S18]-alanine N-acetyltransferase [EC:2.3.1.266]
AVI_00315	K07335	bmpA, bmpB, tmpC; basic membrane protein A and related proteins
AVI_00316	K23537	nupA; general nucleoside transport system ATP-binding protein
AVI_00318	K23535	nupB; general nucleoside transport system permease protein
AVI_00319	K05835	rhtC; threonine efflux protein
AVI_00320	K23536	nupC; general nucleoside transport system permease protein
AVI_00321	K07090	K07090; uncharacterized protein

AVI_00323	K24291	tmpA; [2-(trimethylamino)ethyl]phosphonate dioxygenase [EC:1.14.11.72]
AVI_00325	K20249	raiI; acyl homoserine lactone synthase [EC:2.3.1.184]
AVI_00326	K25873	raiR; LuxR family transcriptional regulator, quorum-sensing system regulator RaiR
AVI_00327	K14446	ccr; crotonyl-CoA carboxylase/reductase [EC:1.3.1.85]
AVI_00330	K14447	ecm; ethylmalonyl-CoA mutase [EC:5.4.99.63]
AVI_00331	K03746	hns; DNA-binding protein H-NS
AVI_00332	K03784	deoD; purine-nucleoside phosphorylase [EC:2.4.2.1]
AVI_00334	K09967	K09967; uncharacterized protein
AVI_00335	K06143	creD; inner membrane protein
AVI_00336	K00240	sdhB, frdB; succinate dehydrogenase iron-sulfur subunit [EC:1.3.5.1]
AVI_00340	K00239	sdhA, frdA; succinate dehydrogenase flavoprotein subunit [EC:1.3.5.1]
AVI_00341	K00242	sdhD, frdD; succinate dehydrogenase membrane anchor subunit
AVI_00342	K00241	sdhC, frdC; succinate dehydrogenase cytochrome b subunit
AVI_00346	K14449	mch, mcd; 2-methylfumaryl-CoA hydratase [EC:4.2.1.148]
AVI_00349	K14451	mcl2; (3S)-malyl-CoA thioesterase [EC:3.1.2.30]
AVI_00350	K11904	vgrG; type VI secretion system secreted protein VgrG
AVI_00358	K00997	acpS; holo-[acyl-carrier protein] synthase [EC:2.7.8.7]
AVI_00359	K03100	lepB; signal peptidase I [EC:3.4.21.89]
AVI_00360	K03100	lepB; signal peptidase I [EC:3.4.21.89]
AVI_00361	K03685	rnc, DROSHA, RNT1; ribonuclease III [EC:3.1.26.3]
AVI_00363	K03595	era, ERAL1; GTPase
AVI_00368	K00383	GSR, gor; glutathione reductase (NADPH) [EC:1.8.1.7]
AVI_00370	K01915	glnA, GLUL; glutamine synthetase [EC:6.3.1.2]
AVI_00371	K11074	potI, spuH; putrescine transport system permease protein
AVI_00372	K11075	potH, spuG; putrescine transport system permease protein
AVI_00373	K11076	potG, spuF; putrescine transport system ATP-binding protein [EC:7.6.2.16]
AVI_00375	K11073	potF, spuD, spuE; putrescine transport system substrate-binding protein
AVI_00376	K12256	spuC; putrescine---pyruvate transaminase [EC:2.6.1.113]
AVI_00377	K09471	puuB, ordL; gamma-glutamylputrescine oxidase [EC:1.4.3.-]
AVI_00379	K03286	TC.OOP; OmpA-OmpF porin, OOP family
AVI_00383	K06206	sfsA; sugar fermentation stimulation protein A
AVI_00384	K01265	map; methionyl aminopeptidase [EC:3.4.11.18]
AVI_00385	K21395	yiaO; TRAP-type transport system periplasmic protein
AVI_00386	K19802	ycjG, ykfB, dgcA; L-Ala-D/L-Glu epimerase / N-acetyl-D-glutamate racemase [EC:5.1.1.20 5.1.1.25]
AVI_00387	K26272	dgcN; D-glutamate N-acetyltransferase [EC:2.3.1.312]
AVI_00388	K08691	mcl; malyl-CoA/(S)-citramalyl-CoA lyase [EC:4.1.3.24 4.1.3.25]
AVI_00389	K03932	lpqC; polyhydroxybutyrate depolymerase
AVI_00391	K00824	dat; D-alanine transaminase [EC:2.6.1.21]
AVI_00393	K09125	yhhQ; queuosine precursor transporter
AVI_00395	K07261	mepA; penicillin-insensitive murein DD-endopeptidase [EC:3.4.24.-]
AVI_00396	K06902	UMF1; MFS transporter, UMF1 family
AVI_00398	K02221	yggT; YggT family protein

AVI_00401	K01991	wza, gfcE; polysaccharide biosynthesis/export protein
AVI_00403	K01784	galE, GALE; UDP-glucose 4-epimerase [EC:5.1.3.2]
AVI_00404	K25706	tsaD; tRNA N6-adenosine threonylcarbamoyltransferase [EC:2.3.1.234]
AVI_00405	K01719	hemD, UROS; uroporphyrinogen-III synthase [EC:4.2.1.75]
AVI_00407	K02498	hemY; HemY protein
AVI_00413	K02051	ABC.SN.S; NitT/TauT family transport system substrate-binding protein
AVI_00414	K02050	ABC.SN.P; NitT/TauT family transport system permease protein
AVI_00415	K02049	ABC.SN.A; NitT/TauT family transport system ATP-binding protein
AVI_00416	K01480	speB; agmatinase [EC:3.5.3.11]
AVI_00422	K03496	parA, soj; chromosome partitioning protein
AVI_00423	K03498	trkH, trkG, ktrB, ktrD; trk/ktr system potassium uptake protein
AVI_00425	K02909	RP-L31, rpmE; large subunit ribosomal protein L31
AVI_00426	K02884	RP-L19, MRPL19, rplS; large subunit ribosomal protein L19
AVI_00427	K00554	trmD; tRNA (guanine37-N1)-methyltransferase [EC:2.1.1.228]
AVI_00429	K02860	rimM; 16S rRNA processing protein RimM
AVI_00432	K02959	RP-S16, MRPS16, rpsP; small subunit ribosomal protein S16
AVI_00433	K04092	tyrA1; chorismate mutase [EC:5.4.99.5]
AVI_00435	K07112	K07112; uncharacterized protein
AVI_00436	K07112	K07112; uncharacterized protein
AVI_00447	K03106	SRP54, ffh; signal recognition particle subunit SRP54 [EC:3.6.5.4]
AVI_00453	K14731	mlhB, chnC; epsilon-lactone hydrolase [EC:3.1.1.83]
AVI_00456	K01118	acpD, azoR; FMN-dependent NADH-azoreductase [EC:1.7.1.17]
AVI_00460	K02116	atpI; ATP synthase protein I
AVI_00461	K02108	ATPF0A, atpB; F-type H+-transporting ATPase subunit a
AVI_00462	K02110	ATPF0C, atpE; F-type H+-transporting ATPase subunit c
AVI_00463	K02109	ATPF0B, atpF; F-type H+-transporting ATPase subunit b
AVI_00464	K02109	ATPF0B, atpF; F-type H+-transporting ATPase subunit b
AVI_00466	K17285	SELENBP1; methanethiol oxidase [EC:1.8.3.4]
AVI_00467	K05799	pdhR; GntR family transcriptional regulator, transcriptional repressor for pyruvate dehydrogenase complex
AVI_00469	K10778	ada; AraC family transcriptional regulator, regulatory protein of adaptative response / methylated-DNA-[protein]-cysteine methyltransferase [EC:2.1.1.63]
AVI_00470	K10773	NTHL1, nth; endonuclease III [EC:3.2.2.- 4.2.99.18]
AVI_00474	K02503	HINT1_2, hinT, hit; histidine triad (HIT) family protein [EC:3.9.1.-]
AVI_00475	K08484	ptsP; phosphotransferase system, enzyme I, PtsP [EC:2.7.3.9]
AVI_00478	K08688	E3.5.3.3; creatinase [EC:3.5.3.3]
AVI_00479	K10823	oppF; oligopeptide transport system ATP-binding protein
AVI_00480	K15583	oppD; oligopeptide transport system ATP-binding protein
AVI_00481	K15582	oppC; oligopeptide transport system permease protein
AVI_00482	K15581	oppB; oligopeptide transport system permease protein
AVI_00483	K15580	oppA, mppA; oligopeptide transport system substrate-binding protein
AVI_00485	K01840	manB; phosphomannomutase [EC:5.4.2.8]
AVI_00486	K16011	algA, xanB, rfbA, wbpW, pslB; mannose-1-phosphate guanylyltransferase / mannose-6-phosphate isomerase [EC:2.7.7.13 5.3.1.8]
AVI_00490	K01610	pckA; phosphoenolpyruvate carboxykinase (ATP) [EC:4.1.1.49]

AVI_00491	K14981	chvI; two-component system, OmpR family, response regulator ChvI
AVI_00492	K14980	chvG; two-component system, OmpR family, sensor histidine kinase ChvG [EC:2.7.13.3]
AVI_00494	K06958	rapZ; RNase adapter protein RapZ
AVI_00495	K02793	manXa; mannose PTS system EIIA component [EC:2.7.1.191]
AVI_00496	K02784	ptsH; phosphocarrier protein HPr
AVI_00498	K00074	paaH, hbd, fadB, mmgB; 3-hydroxybutyryl-CoA dehydrogenase [EC:1.1.1.157]
AVI_00501	K03522	fixB, etfA; electron transfer flavoprotein alpha subunit
AVI_00502	K03521	fixA, etfB; electron transfer flavoprotein beta subunit
AVI_00505	K03527	ispH, lytB; 4-hydroxy-3-methylbut-2-en-1-yl diphosphate reductase [EC:1.17.7.4]
AVI_00509	K03469	rnhA, RNASEH1; ribonuclease HI [EC:3.1.26.4]
AVI_00510	K00604	MTFMT, fmt; methionyl-tRNA formyltransferase [EC:2.1.2.9]
AVI_00511	K01462	PDF, def; peptide deformylase [EC:3.5.1.88]
AVI_00512	K01462	PDF, def; peptide deformylase [EC:3.5.1.88]
AVI_00513	K01462	PDF, def; peptide deformylase [EC:3.5.1.88]
AVI_00514	K14155	patB, malY; cysteine-S-conjugate beta-lyase [EC:4.4.1.13]
AVI_00518	K05936	cobM, cbiF; precorrin-4/cobalt-precorrin-4 C11-methyltransferase [EC:2.1.1.133 2.1.1.271]
AVI_00519	K13541	cbiGH-cobJ; cobalt-precorrin 5A hydrolase / cobalt-factor III methyltransferase / precorrin-3B C17-methyltransferase [EC:3.7.1.12 2.1.1.272 2.1.1.131]
AVI_00520	K03394	cobI-cbiL; precorrin-2/cobalt-factor-2 C20-methyltransferase [EC:2.1.1.130 2.1.1.151]
AVI_00521	K00595	cobL-cbiET; precorrin-6B C5,15-methyltransferase / cobalt-precorrin-6B C5,C15-methyltransferase [EC:2.1.1.132 2.1.1.289 2.1.1.196]
AVI_00522	K06042	cobH-cbiC; precorrin-8X/cobalt-precorrin-8 methylmutase [EC:5.4.99.61 5.4.99.60]
AVI_00523	K03795	cbiX; sirohydrochlorin cobaltochelatase [EC:4.99.1.3]
AVI_00525	K07393	ECM4, yqjG; glutathionyl-hydroquinone reductase [EC:1.8.5.7]
AVI_00528	K02225	cobC1, cobC; cobalamin biosynthesis protein CobC
AVI_00529	K02227	cbiB, cobD; adenosylcobinamide-phosphate synthase [EC:6.3.1.10]
AVI_00533	K25911	pssJ; exopolysaccharide biosynthesis galactosyltransferase PssJ
AVI_00534	K03529	smc; chromosome segregation protein
AVI_00535	K03192	ureJ; urease accessory protein
AVI_00536	K02234	cobW; cobalamin biosynthesis protein CobW
AVI_00537	K03829	yedL; putative acetyltransferase [EC:2.3.1.-]
AVI_00539	K03829	yedL; putative acetyltransferase [EC:2.3.1.-]
AVI_00540	K02230	cobN; cobaltochelatase CobN [EC:6.6.1.2]
AVI_00543	K19221	cobA, btuR; cob(I)alamin adenosyltransferase [EC:2.5.1.17]
AVI_00545	K01652	E2.2.1.6L, ilvB, ilvG, ilvI; acetolactate synthase I/II/III large subunit [EC:2.2.1.6]
AVI_00546	K19784	chrR, NQR; chromate reductase, NAD(P)H dehydrogenase (quinone)
AVI_00551	K08309	slt; peptidoglycan lytic transglycosylase [EC:4.2.2.29]
AVI_00552	K01714	dapA; 4-hydroxy-tetrahydrodipicolinate synthase [EC:4.3.3.7]
AVI_00554	K03566	gcvA; LysR family transcriptional regulator, glycine cleavage system transcriptional activator
AVI_00555	K03664	smpB; SsrA-binding protein
AVI_00556	K01011	TST, MPST, sseA; thiosulfate/3-mercaptopyruvate sulfurtransferase [EC:2.8.1.1 2.8.1.2]
AVI_00557	K00832	tyrB; aromatic-amino-acid transaminase [EC:2.6.1.57]
AVI_00558	K03320	amt, AMT, MEP; ammonium transporter, Amt family
AVI_00559	K04751	glnB; nitrogen regulatory protein P-II 1

AVI_00561	K12536	hasD, prtD, aprD, rsaD, prsD, eexD; ATP-binding cassette, subfamily C, type I secretion system permease/ATPase
AVI_00562	K12537	hasE, prtE, rsaE, prsE, eexE; membrane fusion protein, type I secretion system
AVI_00565	K09919	K09919; uncharacterized protein
AVI_00566	K01724	PCBD, phhB; 4a-hydroxytetrahydrobiopterin dehydratase [EC:4.2.1.96]
AVI_00567	K03442	mscS; small conductance mechanosensitive channel
AVI_00568	K24138	prx3; glutaredoxin/glutathione-dependent peroxiredoxin [EC:1.11.1.25 1.11.1.27]
AVI_00569	K00529	hcaD; 3-phenylpropionate/trans-cinnamate dioxygenase ferredoxin reductase component [EC:1.18.1.3]
AVI_00570	K08316	rsmD; 16S rRNA (guanine966-N2)-methyltransferase [EC:2.1.1.171]
AVI_00571	K13652	K13652; AraC family transcriptional regulator
AVI_00572	K01962	accA; acetyl-CoA carboxylase carboxyl transferase subunit alpha [EC:6.4.1.2 2.1.3.15]
AVI_00573	K00655	plsC; 1-acyl-sn-glycerol-3-phosphate acyltransferase [EC:2.3.1.51]
AVI_00574	K09811	ftsX; cell division transport system permease protein
AVI_00575	K09812	ftsE; cell division transport system ATP-binding protein
AVI_00578	K01586	lysA; diaminopimelate decarboxylase [EC:4.1.1.20]
AVI_00579	K01246	tag; DNA-3-methyladenine glycosylase I [EC:3.2.2.20]
AVI_00581	K00626	ACAT, atoB; acetyl-CoA C-acetyltransferase [EC:2.3.1.9]
AVI_00584	K00023	phbB; acetoacetyl-CoA reductase [EC:1.1.1.36]
AVI_00586	K00057	gpsA; glycerol-3-phosphate dehydrogenase (NAD(P)+) [EC:1.1.1.94]
AVI_00587	K00012	UGDH, ugd; UDPglucose 6-dehydrogenase [EC:1.1.1.22]
AVI_00588	K06041	kdsD, kpsF; arabinose-5-phosphate isomerase [EC:5.3.1.13]
AVI_00589	K01627	kdsA; 2-dehydro-3-deoxyphosphooctonate aldolase (KDO 8-P synthase) [EC:2.5.1.55]
AVI_00591	K03654	recQ; ATP-dependent DNA helicase RecQ [EC:5.6.2.4]
AVI_00592	K21929	udg; uracil-DNA glycosylase [EC:3.2.2.27]
AVI_00594	K07096	K07096; uncharacterized protein
AVI_00595	K03638	moaB; molybdopterin adenylyltransferase [EC:2.7.7.75]
AVI_00596	K03585	acrA, mexA, adeI, smeD, mtrC, cmeA; membrane fusion protein, multidrug efflux system
AVI_00600	K03852	xsc; sulfoacetaldehyde acetyltransferase [EC:2.3.3.15]
AVI_00601	K06911	PIR; quercetin 2,3-dioxygenase [EC:1.13.11.24]
AVI_00603	K03851	tpa; taurine-pyruvate aminotransferase [EC:2.6.1.77]
AVI_00604	K15551	tauA; taurine transport system substrate-binding protein
AVI_00605	K10831	tauB; taurine transport system ATP-binding protein [EC:7.6.2.7]
AVI_00606	K15552	tauC; taurine transport system permease protein
AVI_00607	K15034	yaeJ; ribosome-associated protein
AVI_00609	K06177	rluA; tRNA pseudouridine32 synthase / 23S rRNA pseudouridine746 synthase [EC:5.4.99.28 5.4.99.29]
AVI_00610	K08968	msrC; L-methionine (R)-S-oxide reductase [EC:1.8.4.14]
AVI_00611	K00626	ACAT, atoB; acetyl-CoA C-acetyltransferase [EC:2.3.1.9]
AVI_00612	K00681	ggt; gamma-glutamyltranspeptidase / glutathione hydrolase [EC:2.3.2.2 3.4.19.13]
AVI_00613	K11940	hspQ; heat shock protein HspQ
AVI_00617	K03466	ftsK, spoIIIE; DNA segregation ATPase FtsK/SpoIIIE, S-DNA-T family
AVI_00619	K02433	gatA, QRSL1; aspartyl-tRNA(Asn)/glutamyl-tRNA(Gln) amidotransferase subunit A [EC:6.3.5.6 6.3.5.7]
AVI_00620	K03185	ubiH; 2-octaprenyl-6-methoxyphenol hydroxylase [EC:1.14.13.-]
AVI_00621	K09791	K09791; uncharacterized protein

AVI_00622	K07157	K07157; uncharacterized protein
AVI_00623	K05838	ybbN; putative thioredoxin
AVI_00624	K01142	E3.1.11.2, xthA; exodeoxyribonuclease III [EC:3.1.11.2]
AVI_00626	K01497	ribA, RIB1; GTP cyclohydrolase II [EC:3.5.4.25]
AVI_00628	K06997	yggS, PROSC; PLP dependent protein
AVI_00629	K08720	ompU; outer membrane protein OmpU
AVI_00631	K01869	LARS, leuS; leucyl-tRNA synthetase [EC:6.1.1.4]
AVI_00632	K03643	lptE, rlpB; LPS-assembly lipoprotein
AVI_00633	K02340	holA; DNA polymerase III subunit delta [EC:2.7.7.7]
AVI_00634	K06162	phnM; alpha-D-ribose 1-methylphosphonate 5-triphosphate diphosphatase [EC:3.6.1.63]
AVI_00636	K05774	phnN; ribose 1,5-bisphosphokinase [EC:2.7.4.23]
AVI_00637	K05780	phnL; alpha-D-ribose 1-methylphosphonate 5-triphosphate synthase subunit PhnL [EC:2.7.8.37]
AVI_00638	K05781	phnK; putative phosphonate transport system ATP-binding protein
AVI_00640	K21252	fosX; fosfomycin resistance protein FosX
AVI_00642	K06163	phnJ; alpha-D-ribose 1-methylphosphonate 5-phosphate C-P lyase [EC:4.7.1.1]
AVI_00644	K06164	phnI; alpha-D-ribose 1-methylphosphonate 5-triphosphate synthase subunit PhnI [EC:2.7.8.37]
AVI_00645	K06165	phnH; alpha-D-ribose 1-methylphosphonate 5-triphosphate synthase subunit PhnH [EC:2.7.8.37]
AVI_00646	K06166	phnG; alpha-D-ribose 1-methylphosphonate 5-triphosphate synthase subunit PhnG [EC:2.7.8.37]
AVI_00647	K02043	phnF; GntR family transcriptional regulator, phosphonate transport system regulatory protein
AVI_00652	K03592	pmbA; PmbA protein
AVI_00653	K01092	E3.1.3.25, IMPA, suhB; myo-inositol-1(or 4)-monophosphatase [EC:3.1.3.25]
AVI_00655	K02527	kdtA, waaA; 3-deoxy-D-manno-octulosonic-acid transferase [EC:2.4.99.12 2.4.99.13 2.4.99.14 2.4.99.15]
AVI_00656	K00912	lpxK; tetraacyldisaccharide 4'-kinase [EC:2.7.1.130]
AVI_00657	K06153	bacA; undecaprenyl-diphosphatase [EC:3.6.1.27]
AVI_00658	K00266	gltD; glutamate synthase (NADPH) small chain [EC:1.4.1.13]
AVI_00667	K00265	gltB; glutamate synthase (NADPH) large chain [EC:1.4.1.13]
AVI_00669	K03814	mtgA; monofunctional glycosyltransferase [EC:2.4.99.28]
AVI_00670	K00799	GST, gst; glutathione S-transferase [EC:2.5.1.18]
AVI_00671	K18979	queG; epoxyqueuosine reductase [EC:1.17.99.6]
AVI_00673	K21430	yliI; aldose sugar dehydrogenase [EC:1.1.5.-]
AVI_00682	K01079	serB, PSPH; phosphoserine phosphatase [EC:3.1.3.3]
AVI_00683	K00831	serC, PSAT1; phosphoserine aminotransferase [EC:2.6.1.52]
AVI_00684	K00058	serA, PHGDH; D-3-phosphoglycerate dehydrogenase / 2-oxoglutarate reductase [EC:1.1.1.95 1.1.1.399]
AVI_00687	K06917	selU, mnmH; tRNA 2-selenouridine synthase [EC:2.9.1.3]
AVI_00688	K01008	selD, SEPHS; selenide, water dikinase [EC:2.7.9.3]
AVI_00689	K00275	pdxH, PNPO; pyridoxamine 5'-phosphate oxidase [EC:1.4.3.5]
AVI_00691	K02004	ABC.CD.P; putative ABC transport system permease protein
AVI_00692	K02003	ABC.CD.A; putative ABC transport system ATP-binding protein
AVI_00693	K05805	creA; CreA protein
AVI_00696	K06886	glbN; hemoglobin
AVI_00701	K03470	rnhB; ribonuclease HII [EC:3.1.26.4]
AVI_00702	K13581	ccrM; modification methylase [EC:2.1.1.72]

AVI_00708	K05818	mhpR; IclR family transcriptional regulator, mhp operon transcriptional activator
AVI_00713	K02040	pstS; phosphate transport system substrate-binding protein
AVI_00714	K00496	alkB1_2, alkM; alkane 1-monooxygenase [EC:1.14.15.3]
AVI_00715	K03575	mutY; A/G-specific adenine glycosylase [EC:3.2.2.31]
AVI_00721	K02030	ABC.PA.S; polar amino acid transport system substrate-binding protein
AVI_00722	K02029	ABC.PA.P; polar amino acid transport system permease protein
AVI_00723	K02028	ABC.PA.A; polar amino acid transport system ATP-binding protein [EC:7.4.2.1]
AVI_00727	K18199	inhA; cyclohexyl-isocyanide hydratase [EC:4.2.1.103]
AVI_00729	K02013	ABC.FEV.A; iron complex transport system ATP-binding protein [EC:7.2.2.-]
AVI_00730	K02016	ABC.FEV.S; iron complex transport system substrate-binding protein
AVI_00731	K02015	ABC.FEV.P; iron complex transport system permease protein
AVI_00732	K01611	speD, AMD1; S-adenosylmethionine decarboxylase [EC:4.1.1.50]
AVI_00733	K03684	rnd; ribonuclease D [EC:3.1.13.5]
AVI_00734	K11719	lptC; lipopolysaccharide export system protein LptC
AVI_00735	K09774	lptA; lipopolysaccharide export system protein LptA
AVI_00736	K06861	lptB; lipopolysaccharide export system ATP-binding protein [EC:7.5.2.5]
AVI_00737	K05808	hpf; ribosome hibernation promoting factor
AVI_00738	K02806	ptsN; nitrogen PTS system EIIA component [EC:2.7.1.-]
AVI_00743	K01784	galE, GALE; UDP-glucose 4-epimerase [EC:5.1.3.2]
AVI_00744	K00963	UGP2, galU, galF; UTP--glucose-1-phosphate uridylyltransferase [EC:2.7.7.9]
AVI_00746	K00979	kdsB; 3-deoxy-manno-octulosonate cytidylyltransferase (CMP-KDO synthetase) [EC:2.7.7.38]
AVI_00747	K01082	cysQ, MET22, BPNT1; 3'(2'), 5'-bisphosphate nucleotidase [EC:3.1.3.7]
AVI_00750	K03919	alkB; DNA oxidative demethylase [EC:1.14.11.33]
AVI_00751	K04043	dnaK, HSPA9; molecular chaperone DnaK
AVI_00752	K03686	dnaJ; molecular chaperone DnaJ
AVI_00753	K03630	radC; DNA repair protein RadC
AVI_00754	K02040	pstS; phosphate transport system substrate-binding protein
AVI_00756	K03070	secA; preprotein translocase subunit SecA [EC:7.4.2.8]
AVI_00757	K03769	ppiC; peptidyl-prolyl cis-trans isomerase C [EC:5.2.1.8]
AVI_00758	K00620	argJ; glutamate N-acetyltransferase / amino-acid N-acetyltransferase [EC:2.3.1.35 2.3.1.1]
AVI_00759	K03574	mutT, NUDT15, MTH2; 8-oxo-dGTP diphosphatase [EC:3.6.1.55]
AVI_00761	K02519	infB, MTIF2; translation initiation factor IF-2
AVI_00762	K07742	ylxR; uncharacterized protein
AVI_00763	K02600	nusA; transcription termination/antitermination protein NusA
AVI_00764	K09748	rimP; ribosome maturation factor RimP
AVI_00766	K01259	pip; proline iminopeptidase [EC:3.4.11.5]
AVI_00767	K00568	ubiG; 2-polyprenyl-6-hydroxyphenyl methylase / 3-demethylubiquinone-9 3-methyltransferase [EC:2.1.1.222 2.1.1.64]
AVI_00769	K11206	NIT1, ybeM; deaminated glutathione amidase [EC:3.5.1.128]
AVI_00770	K03676	grxC, GLRX, GLRX2; glutaredoxin 3
AVI_00773	K01772	hemH, FECH; protoporphyrin/coproporphyrin ferrochelatase [EC:4.98.1.1 4.99.1.9]
AVI_00778	K03215	rumA; 23S rRNA (uracil1939-C5)-methyltransferase [EC:2.1.1.190]
AVI_00779	K06147	ABCB-BAC; ATP-binding cassette, subfamily B, bacterial

AVI_00783	K18893	vcaM; ATP-binding cassette, subfamily B, multidrug efflux pump
AVI_00784	K00970	pcnB; poly(A) polymerase [EC:2.7.7.19]
AVI_00788	K04083	hslO; molecular chaperone Hsp33
AVI_00789	K03574	mutT, NUDT15, MTH2; 8-oxo-dGTP diphosphatase [EC:3.6.1.55]
AVI_00790	K01754	E4.3.1.19, ilvA, tdcB; threonine dehydratase [EC:4.3.1.19]
AVI_00794	K01940	argG, ASS1; argininosuccinate synthase [EC:6.3.4.5]
AVI_00795	K21014	stf0; trehalose 2-sulfotransferase [EC:2.8.2.37]
AVI_00798	K07304	msrA; peptide-methionine (S)-S-oxide reductase [EC:1.8.4.11]
AVI_00799	K00852	rbsK, RBKS; ribokinase [EC:2.7.1.15]
AVI_00800	K03555	mutS; DNA mismatch repair protein MutS
AVI_00801	K03687	GRPE; molecular chaperone GrpE
AVI_00802	K03705	hrcA; heat-inducible transcriptional repressor
AVI_00803	K00989	rph; ribonuclease PH [EC:2.7.7.56]
AVI_00804	K01519	rdgB, ITPA; XTP/dITP diphosphohydrolase [EC:3.6.1.66]
AVI_00806	K03497	parB, spo0J; ParB family transcriptional regulator, chromosome partitioning protein
AVI_00807	K03496	parA, soj; chromosome partitioning protein
AVI_00808	K03501	gidB, rsmG; 16S rRNA (guanine527-N7)-methyltransferase [EC:2.1.1.170]
AVI_00809	K03495	gidA, mnmG, MTO1; tRNA uridine 5-carboxymethylaminomethyl modification enzyme
AVI_00810	K03650	mnmE, trmE, MSS1; tRNA modification GTPase [EC:3.6.-.-]
AVI_00811	K03628	rho; transcription termination factor Rho
AVI_00812	K08973	hemJ; protoporphyrinogen IX oxidase [EC:1.3.99.-]
AVI_00813	K06287	yhdE; nucleoside triphosphate pyrophosphatase [EC:3.6.1.-]
AVI_00814	K00014	aroE; shikimate dehydrogenase [EC:1.1.1.25]
AVI_00815	K00859	coaE; dephospho-CoA kinase [EC:2.7.1.24]
AVI_00816	K02342	dnaQ; DNA polymerase III subunit epsilon [EC:2.7.7.7]
AVI_00817	K03071	secB; preprotein translocase subunit SecB
AVI_00818	K07113	fxsA; UPF0716 protein FxsA
AVI_00820	K08304	mltA; peptidoglycan lytic transglycosylase A [EC:4.2.2.29]
AVI_00822	K02470	gyrB; DNA gyrase subunit B [EC:5.6.2.2]
AVI_00823	K03629	recF; DNA replication and repair protein RecF
AVI_00824	K02338	dnaN; DNA polymerase III subunit beta [EC:2.7.7.7]
AVI_00825	K02313	dnaA; chromosomal replication initiator protein
AVI_00826	K02968	RP-S20, rpsT; small subunit ribosomal protein S20
AVI_00827	K01715	crt; enoyl-CoA hydratase [EC:4.2.1.17]
AVI_00828	K10563	mutM, fpg; formamidopyrimidine-DNA glycosylase [EC:3.2.2.23 4.2.99.18]
AVI_00830	K03183	ubiE; demethylmenaquinone methyltransferase / 2-methoxy-6-polyprenyl-1,4-benzoquinol methylase [EC:2.1.1.163 2.1.1.201]
AVI_00831	K03688	ubiB, aarF; ubiquinone biosynthesis protein
AVI_00832	K02389	flgD; flagellar basal-body rod modification protein FlgD
AVI_00834	K02395	flgJ; peptidoglycan hydrolase FlgJ
AVI_00838	K06601	flbT; flagellar biosynthesis repressor protein FlbT
AVI_00840	K03490	chbR, celD; AraC family transcriptional regulator, dual regulator of chb operon
AVI_00843	K02035	ABC.PE.S; peptide/nickel transport system substrate-binding protein

AVI_00844	K02033	ABC.PE.P; peptide/nickel transport system permease protein
AVI_00845	K02034	ABC.PE.P1; peptide/nickel transport system permease protein
AVI_00846	K02031	ddpD; peptide/nickel transport system ATP-binding protein
AVI_00847	K02032	ddpF; peptide/nickel transport system ATP-binding protein
AVI_00848	K01191	MAN2C1; alpha-mannosidase [EC:3.2.1.24]
AVI_00849	K03453	TC.BASS; bile acid:Na+ symporter, BASS family
AVI_00850	K07077	K07077; uncharacterized protein
AVI_00851	K00077	panE, apbA; 2-dehydropantoate 2-reductase [EC:1.1.1.169]
AVI_00852	K09857	K09857; uncharacterized protein
AVI_00853	K06192	pqiB; paraquat-inducible protein B
AVI_00854	K03808	pqiA; paraquat-inducible protein A
AVI_00855	K03808	pqiA; paraquat-inducible protein A
AVI_00856	K02032	ddpF; peptide/nickel transport system ATP-binding protein
AVI_00857	K02031	ddpD; peptide/nickel transport system ATP-binding protein
AVI_00859	K02034	ABC.PE.P1; peptide/nickel transport system permease protein
AVI_00860	K02033	ABC.PE.P; peptide/nickel transport system permease protein
AVI_00862	K01821	praC, xylH; 4-oxalocrotonate tautomerase [EC:5.3.2.6]
AVI_00863	K16137	nemR; TetR/AcrR family transcriptional regulator, transcriptional repressor for nem operon
AVI_00867	K02035	ABC.PE.S; peptide/nickel transport system substrate-binding protein
AVI_00869	K09982	K09982; uncharacterized protein
AVI_00870	K01593	DDC, TDC; aromatic-L-amino-acid/L-tryptophan decarboxylase [EC:4.1.1.28 4.1.1.105]
AVI_00871	K03885	ndh; NADH:quinone reductase (non-electrogenic) [EC:1.6.5.9]
AVI_00874	K02035	ABC.PE.S; peptide/nickel transport system substrate-binding protein
AVI_00875	K02557	motB; chemotaxis protein MotB
AVI_00876	K02390	flgE; flagellar hook protein FlgE
AVI_00877	K02396	flgK; flagellar hook-associated protein 1
AVI_00878	K02397	flgL; flagellar hook-associated protein 3 FlgL
AVI_00879	K02394	flgI; flagellar P-ring protein FlgI
AVI_00880	K02419	fliP; flagellar biosynthesis protein FliP
AVI_00881	K02417	fliN; flagellar motor switch protein FliN
AVI_00883	K02409	fliF; flagellar M-ring protein FliF
AVI_00884	K02415	fliL; flagellar protein FliL
AVI_00887	K02556	motA; chemotaxis protein MotA
AVI_00890	K02400	flhA; flagellar biosynthesis protein FlhA
AVI_00891	K02421	fliR; flagellar biosynthesis protein FliR
AVI_00892	K02401	flhB; flagellar biosynthesis protein FlhB
AVI_00894	K02415	fliL; flagellar protein FliL
AVI_00895	K02393	flgH; flagellar L-ring protein FlgH
AVI_00896	K02386	flgA; flagellar basal body P-ring formation protein FlgA
AVI_00897	K02392	flgG; flagellar basal-body rod protein FlgG
AVI_00898	K02391	flgF; flagellar basal-body rod protein FlgF
AVI_00899	K02420	fliQ; flagellar biosynthesis protein FliQ

AVI_00900	K02408	fliE; flagellar hook-basal body complex protein FliE
AVI_00901	K02388	flgC; flagellar basal-body rod protein FlgC
AVI_00902	K02387	flgB; flagellar basal-body rod protein FlgB
AVI_00903	K02412	fliI; flagellum-specific ATP synthase [EC:7.4.2.8]
AVI_00904	K06145	gntR; LacI family transcriptional regulator, gluconate utilization system Gnt-I transcriptional repressor
AVI_00908	K18302	mexJ; membrane fusion protein, multidrug efflux system
AVI_00911	K00826	E2.6.1.42, ilvE; branched-chain amino acid aminotransferase [EC:2.6.1.42]
AVI_00913	K07659	ompR; two-component system, OmpR family, phosphate regulon response regulator OmpR
AVI_00915	K03602	xseB; exodeoxyribonuclease VII small subunit [EC:3.1.11.6]
AVI_00916	K00795	ispA; farnesyl diphosphate synthase [EC:2.5.1.1 2.5.1.10]
AVI_00917	K01662	dxs; 1-deoxy-D-xylulose-5-phosphate synthase [EC:2.2.1.7]
AVI_00918	K08714	VGSC; voltage-gated sodium channel
AVI_00921	K01486	ade; adenine deaminase [EC:3.5.4.2]
AVI_00922	K01241	amn; AMP nucleosidase [EC:3.2.2.4]
AVI_00923	K03530	hupB; DNA-binding protein HU-beta
AVI_00924	K01736	aroC; chorismate synthase [EC:4.2.3.5]
AVI_00925	K02064	thiB, tbpA; thiamine transport system substrate-binding protein
AVI_00926	K02063	thiP; thiamine transport system permease protein
AVI_00927	K02062	thiQ; thiamine transport system ATP-binding protein [EC:7.6.2.15]
AVI_00928	K00413	CYC1, CYT1, petC; ubiquinol-cytochrome c reductase cytochrome c1 subunit
AVI_00929	K00412	CYTB, petB; ubiquinol-cytochrome c reductase cytochrome b subunit
AVI_00930	K00411	UQCRFS1, RIP1, petA; ubiquinol-cytochrome c reductase iron-sulfur subunit [EC:7.1.1.8]
AVI_00931	K12980	lpxQ; lipid A oxidase
AVI_00933	K07734	paiB; transcriptional regulator
AVI_00934	K18707	mtaB; threonylcarbamoyladenosine tRNA methylthiotransferase MtaB [EC:2.8.4.5]
AVI_00935	K01778	dapF; diaminopimelate epimerase [EC:5.1.1.7]
AVI_00937	K01561	dehH; haloacetate dehalogenase [EC:3.8.1.3]
AVI_00938	K06162	phnM; alpha-D-ribose 1-methylphosphonate 5-triphosphate diphosphatase [EC:3.6.1.63]
AVI_00939	K02042	phnE; phosphonate transport system permease protein
AVI_00940	K02041	phnC; phosphonate transport system ATP-binding protein [EC:7.3.2.2]
AVI_00941	K02044	phnD; phosphonate transport system substrate-binding protein
AVI_00942	K02012	afuA, fbpA; iron(III) transport system substrate-binding protein
AVI_00945	K02011	afuB, fbpB; iron(III) transport system permease protein
AVI_00947	K04047	dps; starvation-inducible DNA-binding protein
AVI_00948	K06938	K06938; uncharacterized protein
AVI_00955	K02027	ABC.MS.S; multiple sugar transport system substrate-binding protein
AVI_00956	K02025	ABC.MS.P; multiple sugar transport system permease protein
AVI_00957	K02026	ABC.MS.P1; multiple sugar transport system permease protein
AVI_00959	K25649	mgh; mannosylglycerate hydrolase [EC:3.2.1.170]
AVI_00960	K10111	malK, mtlK, thuK; multiple sugar transport system ATP-binding protein [EC:7.5.2.-]
AVI_00962	K24158	prx; thioredoxin-dependent peroxiredoxin [EC:1.11.1.24]
AVI_00963	K01061	E3.1.1.45; carboxymethylenebutenolidase [EC:3.1.1.45]

AVI_00966	K04095	fic, FICD, HYPE; cell filamentation protein, protein adenylyltransferase [EC:2.7.7.108]
AVI_00967	K00962	pnp, PNPT1; polyribonucleotide nucleotidyltransferase [EC:2.7.7.8]
AVI_00969	K07086	K07086; uncharacterized protein
AVI_00972	K02956	RP-S15, MRPS15, rpsO; small subunit ribosomal protein S15
AVI_00974	K01561	dehH; haloacetate dehalogenase [EC:3.8.1.3]
AVI_00976	K03177	truB, PUS4, TRUB1; tRNA pseudouridine55 synthase [EC:5.4.99.25]
AVI_00978	K02834	rbfA; ribosome-binding factor A
AVI_00979	K00215	dapB; 4-hydroxy-tetrahydrodipicolinate reductase [EC:1.17.1.8]
AVI_00982	K03500	rsmB, sun; 16S rRNA (cytosine967-C5)-methyltransferase [EC:2.1.1.176]
AVI_00984	K00602	purH; phosphoribosylaminoimidazolecarboxamide formyltransferase / IMP cyclohydrolase [EC:2.1.2.3 3.5.4.10]
AVI_00985	K03101	lspA; signal peptidase II [EC:3.4.23.36]
AVI_00988	K07263	pqqL; zinc protease [EC:3.4.24.-]
AVI_00989	K07263	pqqL; zinc protease [EC:3.4.24.-]
AVI_00990	K03572	mutL; DNA mismatch repair protein MutL
AVI_00991	K09760	rmuC; DNA recombination protein RmuC
AVI_00994	K07167	chrR; putative transcriptional regulator
AVI_00995	K03088	rpoE; RNA polymerase sigma-70 factor, ECF subfamily
AVI_00996	K06954	K06954; uncharacterized protein
AVI_00997	K09701	K09701; uncharacterized protein
AVI_00998	K03292	TC.GPH; glycoside/pentoside/hexuronide:cation symporter, GPH family
AVI_01002	K00290	LYS1; saccharopine dehydrogenase (NAD+, L-lysine forming) [EC:1.5.1.7]
AVI_01003	K00123	fdoG, fdhF, fdwA; formate dehydrogenase major subunit [EC:1.17.1.9]
AVI_01004	K01897	ACSL, fadD; long-chain acyl-CoA synthetase [EC:6.2.1.3]
AVI_01005	K01995	livG; branched-chain amino acid transport system ATP-binding protein
AVI_01007	K01997	livH; branched-chain amino acid transport system permease protein
AVI_01009	K01998	livM; branched-chain amino acid transport system permease protein
AVI_01010	K01999	livK; branched-chain amino acid transport system substrate-binding protein
AVI_01011	K01996	livF; branched-chain amino acid transport system ATP-binding protein
AVI_01014	K07303	iorB; isoquinoline 1-oxidoreductase subunit beta [EC:1.3.99.16]
AVI_01015	K07302	iorA; isoquinoline 1-oxidoreductase subunit alpha [EC:1.3.99.16]
AVI_01016	K02919	RP-L36, MRPL36, rpmJ; large subunit ribosomal protein L36
AVI_01019	K02346	dinB; DNA polymerase IV [EC:2.7.7.7]
AVI_01020	K03574	mutT, NUDT15, MTH2; 8-oxo-dGTP diphosphatase [EC:3.6.1.55]
AVI_01027	K00285	dadA; D-amino-acid dehydrogenase [EC:1.4.5.1]
AVI_01032	K01591	pyrF; orotidine-5'-phosphate decarboxylase [EC:4.1.1.23]
AVI_01036	K03695	clpB; ATP-dependent Clp protease ATP-binding subunit ClpB
AVI_01041	K01790	rfbC, rmlC; dTDP-4-dehydrorhamnose 3,5-epimerase [EC:5.1.3.13]
AVI_01044	K07147	msrP; methionine sulfoxide reductase catalytic subunit [EC:1.8.-.-]
AVI_01045	K17247	msrQ; methionine sulfoxide reductase heme-binding subunit
AVI_01053	K10773	NTHL1, nth; endonuclease III [EC:3.2.2.- 4.2.99.18]
AVI_01055	K01990	ABC-2.A; ABC-2 type transport system ATP-binding protein
AVI_01056	K01992	ABC-2.P; ABC-2 type transport system permease protein

AVI_01057	K04774	sohB; serine protease SohB [EC:3.4.21.-]
AVI_01058	K07301	yrbG; cation:H+ antiporter
AVI_01060	K03703	uvrC; excinuclease ABC subunit C
AVI_01061	K00995	pgsA, PGS1; CDP-diacylglycerol---glycerol-3-phosphate 3-phosphatidyltransferase [EC:2.7.8.5]
AVI_01062	K03636	moaD, cysO; sulfur-carrier protein
AVI_01063	K03635	MOCS2B, moaE; molybdopterin synthase catalytic subunit [EC:2.8.1.12]
AVI_01065	K03286	TC.OOP; OmpA-OmpF porin, OOP family
AVI_01066	K03179	ubiA; 4-hydroxybenzoate polyprenyltransferase [EC:2.5.1.39]
AVI_01067	K09761	rsmE; 16S rRNA (uracil1498-N3)-methyltransferase [EC:2.1.1.193]
AVI_01069	K01919	gshA; glutamate--cysteine ligase [EC:6.3.2.2]
AVI_01072	K08591	plsY; acyl phosphate:glycerol-3-phosphate acyltransferase [EC:2.3.1.275]
AVI_01073	K01465	URA4, pyrC; dihydroorotase [EC:3.5.2.3]
AVI_01077	K00609	pyrB, PYR2; aspartate carbamoyltransferase catalytic subunit [EC:2.1.3.2]
AVI_01078	K09861	K09861; uncharacterized protein
AVI_01079	K00798	MMAB, pduO; cob(I)alamin adenosyltransferase [EC:2.5.1.17]
AVI_01084	K11336	bchF; 3-vinyl bacteriochlorophyllide hydratase [EC:4.2.1.165]
AVI_01085	K04038	chlN; light-independent protochlorophyllide reductase subunit N [EC:1.3.7.7]
AVI_01086	K04039	chlB; light-independent protochlorophyllide reductase subunit B [EC:1.3.7.7]
AVI_01087	K03403	chlH, bchH; magnesium chelatase subunit H [EC:6.6.1.1]
AVI_01089	K04037	chlL; light-independent protochlorophyllide reductase subunit L [EC:1.3.7.7]
AVI_01090	K03428	bchM, chlM; magnesium-protoporphyrin O-methyltransferase [EC:2.1.1.11]
AVI_01091	K08226	pucC; MFS transporter, BCD family, chlorophyll transporter
AVI_01092	K13991	puhA; photosynthetic reaction center H subunit
AVI_01096	K04035	E1.14.13.81, acsF, chlE; magnesium-protoporphyrin IX monomethyl ester (oxidative) cyclase [EC:1.14.13.81]
AVI_01098	K00643	E2.3.1.37, ALAS; 5-aminolevulinate synthase [EC:2.3.1.37]
AVI_01099	K08738	CYC; cytochrome c
AVI_01100	K01251	AHCY, ahcY; adenosylhomocysteinase [EC:3.13.2.1]
AVI_01103	K02040	pstS; phosphate transport system substrate-binding protein
AVI_01104	K00481	pobA; p-hydroxybenzoate 3-monooxygenase [EC:1.14.13.2]
AVI_01105	K01607	pcaC; 4-carboxymuconolactone decarboxylase [EC:4.1.1.44]
AVI_01106	K00449	pcaH; protocatechuate 3,4-dioxygenase, beta subunit [EC:1.13.11.3]
AVI_01107	K00448	pcaG; protocatechuate 3,4-dioxygenase, alpha subunit [EC:1.13.11.3]
AVI_01109	K07735	algH; putative transcriptional regulator
AVI_01111	K01273	DPEP; membrane dipeptidase [EC:3.4.13.19]
AVI_01112	K21307	soeA; sulfite dehydrogenase (quinone) subunit SoeA [EC:1.8.5.6]
AVI_01113	K20608	tet; tetrahedral aminopeptidase [EC:3.4.11.-]
AVI_01114	K21308	soeB; sulfite dehydrogenase (quinone) subunit SoeB
AVI_01115	K21309	soeC; sulfite dehydrogenase (quinone) subunit SoeC
AVI_01119	K10111	malK, mtlK, thuK; multiple sugar transport system ATP-binding protein [EC:7.5.2.-]
AVI_01121	K02027	ABC.MS.S; multiple sugar transport system substrate-binding protein
AVI_01122	K02025	ABC.MS.P; multiple sugar transport system permease protein
AVI_01125	K02026	ABC.MS.P1; multiple sugar transport system permease protein

AVI_01126	K07406	melA; alpha-galactosidase [EC:3.2.1.22]
AVI_01127	K22215	galD; galactose dehydrogenase [EC:1.1.1.48 1.1.1.120]
AVI_01128	K00883	dgoK; 2-dehydro-3-deoxygalactonokinase [EC:2.7.1.58]
AVI_01129	K01631	dgoA; 2-dehydro-3-deoxyphosphogalactonate aldolase [EC:4.1.2.21]
AVI_01131	K12308	bgaB, lacA; beta-galactosidase [EC:3.2.1.23]
AVI_01132	K01785	galM, GALM; aldose 1-epimerase [EC:5.1.3.3]
AVI_01133	K09826	irr; Fur family transcriptional regulator, iron response regulator
AVI_01134	K01716	fabA; 3-hydroxyacyl-[acyl-carrier protein] dehydratase / trans-2-decenoyl-[acyl-carrier protein] isomerase [EC:4.2.1.59 5.3.3.14]
AVI_01135	K00647	fabB; 3-oxoacyl-[acyl-carrier-protein] synthase I [EC:2.3.1.41]
AVI_01136	K00208	fabI; enoyl-[acyl-carrier protein] reductase I [EC:1.3.1.9 1.3.1.10]
AVI_01137	K00315	DMGDH; dimethylglycine dehydrogenase [EC:1.5.8.4]
AVI_01139	K26937	dinF, mepA, vmrA; MATE family, multidrug efflux pump
AVI_01140	K00820	glmS, GFPT; glutamine---fructose-6-phosphate transaminase (isomerizing) [EC:2.6.1.16]
AVI_01143	K10112	msmX, msmK, malK, sugC, ggtA, msiK; multiple sugar transport system ATP-binding protein [EC:7.5.2.-]
AVI_01144	K01755	argH, ASL; argininosuccinate lyase [EC:4.3.2.1]
AVI_01146	K02026	ABC.MS.P1; multiple sugar transport system permease protein
AVI_01147	K02025	ABC.MS.P; multiple sugar transport system permease protein
AVI_01148	K02027	ABC.MS.S; multiple sugar transport system substrate-binding protein
AVI_01160	K02433	gatA, QRSL1; aspartyl-tRNA(Asn)/glutamyl-tRNA(Gln) amidotransferase subunit A [EC:6.3.5.6 6.3.5.7]
AVI_01167	K02016	ABC.FEV.S; iron complex transport system substrate-binding protein
AVI_01169	K02016	ABC.FEV.S; iron complex transport system substrate-binding protein
AVI_01170	K02016	ABC.FEV.S; iron complex transport system substrate-binding protein
AVI_01171	K25111	sirC, fecD, cbrC; iron-siderophore transport system permease protein
AVI_01172	K25110	sirB, fecC, cbrB; iron-siderophore transport system permease protein
AVI_01173	K25130	fecE, cbrD; iron-siderophore transport system ATP-binding protein [EC:7.2.2.-]
AVI_01175	K10778	ada; AraC family transcriptional regulator, regulatory protein of adaptative response / methylated-DNA-[protein]-cysteine methyltransferase [EC:2.1.1.63]
AVI_01179	K02899	RP-L27, MRPL27, rpmA; large subunit ribosomal protein L27
AVI_01180	K14415	RTCB, rtcB; tRNA-splicing ligase RtcB (3'-phosphate/5'-hydroxy nucleic acid ligase) [EC:6.5.1.8]
AVI_01183	K03979	obgE, cgtA, MTG2; GTPase [EC:3.6.5.-]
AVI_01184	K00931	proB; glutamate 5-kinase [EC:2.7.2.11]
AVI_01185	K00147	proA; glutamate-5-semialdehyde dehydrogenase [EC:1.2.1.41]
AVI_01186	K13588	chpT; histidine phosphotransferase ChpT
AVI_01188	K22617	olsA; lyso-ornithine lipid O-acyltransferase [EC:2.3.1.270]
AVI_01189	K00788	thiE; thiamine-phosphate pyrophosphorylase [EC:2.5.1.3]
AVI_01190	K02533	lasT; tRNA/rRNA methyltransferase [EC:2.1.1.-]
AVI_01191	K02259	COX15, ctaA; heme a synthase [EC:1.17.99.9]
AVI_01192	K01299	E3.4.17.19; carboxypeptidase Taq [EC:3.4.17.19]
AVI_01196	K02871	RP-L13, MRPL13, rplM; large subunit ribosomal protein L13
AVI_01197	K02996	RP-S9, MRPS9, rpsI; small subunit ribosomal protein S9
AVI_01200	K22468	ppk2; polyphosphate kinase [EC:2.7.4.34]
AVI_01202	K00651	metA; homoserine O-succinyltransferase/O-acetyltransferase [EC:2.3.1.46 2.3.1.31]
AVI_01209	K03584	recO; DNA repair protein RecO (recombination protein O)

AVI_01210	K00847	E2.7.1.4, scrK; fructokinase [EC:2.7.1.4]
AVI_01211	K01443	nagA, AMDHD2; N-acetylglucosamine-6-phosphate deacetylase [EC:3.5.1.25]
AVI_01214	K05020	opuD, betL; glycine betaine transporter
AVI_01215	K02000	proV; glycine betaine/proline transport system ATP-binding protein [EC:7.6.2.9]
AVI_01216	K02001	proW; glycine betaine/proline transport system permease protein
AVI_01217	K02002	proX; glycine betaine/proline transport system substrate-binding protein
AVI_01218	K00108	betA, CHDH; choline dehydrogenase [EC:1.1.99.1]
AVI_01220	K03786	aroQ, qutE; 3-dehydroquinate dehydratase II [EC:4.2.1.10]
AVI_01221	K02068	STAR1, fetA; UDP-glucose/iron transport system ATP-binding protein
AVI_01222	K02069	STAR2, fetB; UDP-glucose/iron transport system permease protein
AVI_01223	K25109	sirA, fecB, cbrA; iron-siderophore transport system substrate-binding protein
AVI_01224	K25110	sirB, fecC, cbrB; iron-siderophore transport system permease protein
AVI_01225	K25111	sirC, fecD, cbrC; iron-siderophore transport system permease protein
AVI_01226	K23188	fepC, fagC, cchE, desF; iron-siderophore transport system ATP-binding protein [EC:7.2.2.17 7.2.2.-]
AVI_01229	K01118	acpD, azoR; FMN-dependent NADH-azoreductase [EC:1.7.1.17]
AVI_01234	K07107	ybgC; acyl-CoA thioester hydrolase [EC:3.1.2.-]
AVI_01236	K01810	GPI, pgi; glucose-6-phosphate isomerase [EC:5.3.1.9]
AVI_01237	K01057	PGLS, pgl, devB; 6-phosphogluconolactonase [EC:3.1.1.31]
AVI_01238	K00036	G6PD, zwf; glucose-6-phosphate 1-dehydrogenase [EC:1.1.1.49 1.1.1.363]
AVI_01241	K06999	K06999; phospholipase/carboxylesterase
AVI_01242	K01247	alkA; DNA-3-methyladenine glycosylase II [EC:3.2.2.21]
AVI_01243	K05895	cobK-cbiJ; precorrin-6A/cobalt-precorrin-6A reductase [EC:1.3.1.54 1.3.1.106]
AVI_01244	K02188	cbiD; cobalt-precorrin-5B (C1)-methyltransferase [EC:2.1.1.195]
AVI_01245	K02303	cobA; uroporphyrin-III C-methyltransferase [EC:2.1.1.107]
AVI_01246	K02224	cobB-cbiA; cobyrinic acid a,c-diamide synthase [EC:6.3.5.9 6.3.5.11]
AVI_01249	K13639	soxR; MerR family transcriptional regulator, redox-sensitive transcriptional activator SoxR
AVI_01252	K01883	CARS, cysS; cysteinyl-tRNA synthetase [EC:6.1.1.16]
AVI_01253	K01649	leuA, IMS; 2-isopropylmalate synthase [EC:2.3.3.13]
AVI_01260	K01768	E4.6.1.1; adenylate cyclase [EC:4.6.1.1]
AVI_01265	K06889	K06889; uncharacterized protein
AVI_01266	K09927	K09927; uncharacterized protein
AVI_01269	K02012	afuA, fbpA; iron(III) transport system substrate-binding protein
AVI_01270	K02011	afuB, fbpB; iron(III) transport system permease protein
AVI_01271	K02010	afuC, fbpC; iron(III) transport system ATP-binding protein [EC:7.2.2.7]
AVI_01274	K21898	orr; ornithine racemase [EC:5.1.1.12]
AVI_01283	K03704	cspA; cold shock protein
AVI_01285	K02348	elaA; ElaA protein
AVI_01286	K22186	xylD; xylonate dehydratase [EC:4.2.1.82]
AVI_01288	K00634	ptb; phosphate butyryltransferase [EC:2.3.1.19]
AVI_01289	K00925	ackA; acetate kinase [EC:2.7.2.1]
AVI_01292	K24662	rgtD; lipid A galacturonosyltransferase RgtD [EC:2.4.1.-]
AVI_01295	K06196	ccdA; cytochrome c-type biogenesis protein

AVI_01296	K04085	tusA, sirA; tRNA 2-thiouridine synthesizing protein A [EC:2.8.1.-]
AVI_01297	K08300	rne; ribonuclease E [EC:3.1.26.12]
AVI_01299	K10125	dctB; two-component system, NtrC family, C4-dicarboxylate transport sensor histidine kinase DctB [EC:2.7.13.3]
AVI_01300	K23265	purQ; phosphoribosylformylglycinamidine synthase subunit PurQ / glutaminase [EC:6.3.5.3 3.5.1.2]
AVI_01301	K00001	E1.1.1.1, adh; alcohol dehydrogenase [EC:1.1.1.1]
AVI_01302	K23264	purS; phosphoribosylformylglycinamidine synthase subunit PurS [EC:6.3.5.3]
AVI_01303	K01923	purC; phosphoribosylaminoimidazole-succinocarboxamide synthase [EC:6.3.2.6]
AVI_01305	K00548	metH, MTR; 5-methyltetrahydrofolate--homocysteine methyltransferase [EC:2.1.1.13]
AVI_01313	K18302	mexJ; membrane fusion protein, multidrug efflux system
AVI_01315	K03312	gltS; glutamate:Na+ symporter, ESS family
AVI_01318	K02426	sufE; cysteine desulfuration protein SufE
AVI_01320	K03684	rnd; ribonuclease D [EC:3.1.13.5]
AVI_01321	K11175	purN; phosphoribosylglycinamide formyltransferase 1 [EC:2.1.2.2]
AVI_01322	K01933	purM; phosphoribosylformylglycinamidine cyclo-ligase [EC:6.3.3.1]
AVI_01326	K07638	envZ; two-component system, OmpR family, osmolarity sensor histidine kinase EnvZ [EC:2.7.13.3]
AVI_01332	K00940	ndk, NME; nucleoside-diphosphate kinase [EC:2.7.4.6]
AVI_01333	K06158	ABCF3; ATP-binding cassette, subfamily F, member 3
AVI_01334	K06985	K06985; aspartyl protease family protein
AVI_01335	K02510	hpaI, hpcH; 4-hydroxy-2-oxoheptanedioate aldolase [EC:4.1.2.52]
AVI_01336	K10235	aglK; alpha-glucoside transport system ATP-binding protein
AVI_01337	K01187	malZ; alpha-glucosidase [EC:3.2.1.20]
AVI_01338	K10234	aglG, ggtD; alpha-glucoside transport system permease protein
AVI_01339	K10233	aglF, ggtC; alpha-glucoside transport system permease protein
AVI_01340	K10232	aglE, ggtB; alpha-glucoside transport system substrate-binding protein
AVI_01341	K05350	bglB; beta-glucosidase [EC:3.2.1.21]
AVI_01342	K00845	glk; glucokinase [EC:2.7.1.2]
AVI_01343	K17321	glpV; glycerol transport system substrate-binding protein
AVI_01346	K17323	glpQ; glycerol transport system permease protein
AVI_01347	K17322	glpP; glycerol transport system permease protein
AVI_01348	K17325	glpT; glycerol transport system ATP-binding protein
AVI_01349	K17324	glpS; glycerol transport system ATP-binding protein
AVI_01350	K00111	glpA, glpD; glycerol-3-phosphate dehydrogenase [EC:1.1.5.3]
AVI_01353	K07727	K07727; putative transcriptional regulator
AVI_01354	K02357	tsf, TSFM; elongation factor Ts
AVI_01355	K02967	RP-S2, MRPS2, rpsB; small subunit ribosomal protein S2
AVI_01363	K16329	psuG; pseudouridylate synthase [EC:4.2.1.70]
AVI_01365	K06895	lysE, argO; L-lysine exporter family protein LysE/ArgO
AVI_01366	K16348	ecnB; entericidin B
AVI_01367	K07001	K07001; NTE family protein
AVI_01368	K00019	BDH1, bdhA; 3-hydroxybutyrate dehydrogenase [EC:1.1.1.30]
AVI_01370	K02035	ABC.PE.S; peptide/nickel transport system substrate-binding protein
AVI_01375	K03741	arsC; arsenate reductase (thioredoxin) [EC:1.20.4.4]

AVI_01380	K18372	acmB; methyl acetate hydrolase [EC:3.1.1.114]
AVI_01385	K24160	NHA1, SOD2; sodium/hydrogen antiporter
AVI_01386	K03723	mfd; transcription-repair coupling factor (superfamily II helicase) [EC:5.6.2.4]
AVI_01388	K01698	hemB, ALAD; porphobilinogen synthase [EC:4.2.1.24]
AVI_01391	K04719	bluB; 5,6-dimethylbenzimidazole synthase [EC:1.13.11.79]
AVI_01394	K00315	DMGDH; dimethylglycine dehydrogenase [EC:1.5.8.4]
AVI_01400	K07390	grxD, GLRX5; monothiol glutaredoxin
AVI_01401	K09888	zapA; cell division protein ZapA
AVI_01403	K00615	E2.2.1.1, tktA, tktB; transketolase [EC:2.2.1.1]
AVI_01408	K00382	DLD, lpd, pdhD; dihydrolipoyl dehydrogenase [EC:1.8.1.4]
AVI_01409	K07114	yfbK; Ca-activated chloride channel homolog
AVI_01412	K03701	uvrA; excinuclease ABC subunit A
AVI_01414	K00020	HIBADH, mmsB; 3-hydroxyisobutyrate dehydrogenase [EC:1.1.1.31]
AVI_01418	K00140	mmsA, iolA, ALDH6A1; malonate-semialdehyde dehydrogenase (acetylating) / methylmalonate-semialdehyde dehydrogenase [EC:1.2.1.18 1.2.1.27]
AVI_01420	K00954	E2.7.7.3A, coaD, kdtB; pantetheine-phosphate adenylyltransferase [EC:2.7.7.3]
AVI_01424	K00134	GAPDH, gapA; glyceraldehyde 3-phosphate dehydrogenase (phosphorylating) [EC:1.2.1.12]
AVI_01426	K07568	queA; S-adenosylmethionine:tRNA ribosyltransferase-isomerase [EC:2.4.99.17]
AVI_01428	K03564	BCP, PRXQ, DOT5; thioredoxin-dependent peroxiredoxin [EC:1.11.1.24]
AVI_01434	K02836	prfB; peptide chain release factor 2
AVI_01435	K05366	mrcA; penicillin-binding protein 1A [EC:2.4.99.28 3.4.16.4]
AVI_01436	K01448	amiABC; N-acetylmuramoyl-L-alanine amidase [EC:3.5.1.28]
AVI_01441	K03526	gcpE, ispG; (E)-4-hydroxy-3-methylbut-2-enyl-diphosphate synthase [EC:1.17.7.1 1.17.7.3]
AVI_01443	K00643	E2.3.1.37, ALAS; 5-aminolevulinate synthase [EC:2.3.1.37]
AVI_01446	K06181	rluE; 23S rRNA pseudouridine2457 synthase [EC:5.4.99.20]
AVI_01447	K00325	pntB; proton-translocating NAD(P)+ transhydrogenase subunit beta [EC:7.1.1.1]
AVI_01448	K00324	pntA; proton-translocating NAD(P)+ transhydrogenase subunit alpha [EC:7.1.1.1]
AVI_01450	K01556	KYNU, kynU; kynureninase [EC:3.7.1.3]
AVI_01451	K03830	yafP; putative acetyltransferase [EC:2.3.1.-]
AVI_01452	K09988	lyxA; D-lyxose ketol-isomerase [EC:5.3.1.15]
AVI_01453	K07010	K07010; putative glutamine amidotransferase
AVI_01455	K01638	aceB, glcB; malate synthase [EC:2.3.3.9]
AVI_01457	K01118	acpD, azoR; FMN-dependent NADH-azoreductase [EC:1.7.1.17]
AVI_01459	K01406	prtC; serralysin [EC:3.4.24.40]
AVI_01464	K01256	pepN; aminopeptidase N [EC:3.4.11.2]
AVI_01466	K01265	map; methionyl aminopeptidase [EC:3.4.11.18]
AVI_01469	K01524	ppx-gppA; exopolyphosphatase / guanosine-5'-triphosphate,3'-diphosphate pyrophosphatase [EC:3.6.1.11 3.6.1.40]
AVI_01471	K00297	metF, MTHFR; methylenetetrahydrofolate reductase (NADH) [EC:1.5.1.54]
AVI_01472	K00548	metH, MTR; 5-methyltetrahydrofolate--homocysteine methyltransferase [EC:2.1.1.13]
AVI_01474	K02339	holC; DNA polymerase III subunit chi [EC:2.7.7.7]
AVI_01475	K01255	CARP, pepA; leucyl aminopeptidase [EC:3.4.11.1]
AVI_01476	K07091	lptF; lipopolysaccharide export system permease protein
AVI_01477	K11720	lptG; lipopolysaccharide export system permease protein

AVI_01478	K04744	lptD, imp, ostA; LPS-assembly protein
AVI_01479	K03771	surA; peptidyl-prolyl cis-trans isomerase SurA [EC:5.2.1.8]
AVI_01480	K00097	pdxA; 4-hydroxythreonine-4-phosphate dehydrogenase [EC:1.1.1.262]
AVI_01481	K02528	ksgA; 16S rRNA (adenine1518-N6/adenine1519-N6)-dimethyltransferase [EC:2.1.1.182]
AVI_01483	K02493	hemK, prmC, HEMK; release factor glutamine methyltransferase [EC:2.1.1.297]
AVI_01484	K02835	prfA, MTRF1, MRF1; peptide chain release factor 1
AVI_01487	K01480	speB; agmatinase [EC:3.5.3.11]
AVI_01488	K04765	mazG; nucleoside triphosphate diphosphatase [EC:3.6.1.9]
AVI_01490	K14448	mcd; (2S)-methylsuccinyl-CoA dehydrogenase [EC:1.3.8.12]
AVI_01491	K07090	K07090; uncharacterized protein
AVI_01492	K01739	metB; cystathionine gamma-synthase [EC:2.5.1.48]
AVI_01494	K00942	gmk, GUK1; guanylate kinase [EC:2.7.4.8]
AVI_01497	K01626	E2.5.1.54, aroF, aroG, aroH; 3-deoxy-7-phosphoheptulonate synthase [EC:2.5.1.54]
AVI_01498	K21826	gbdR; AraC family transcriptional regulator, glycine betaine-responsive activator
AVI_01500	K01999	livK; branched-chain amino acid transport system substrate-binding protein
AVI_01501	K01995	livG; branched-chain amino acid transport system ATP-binding protein
AVI_01502	K01996	livF; branched-chain amino acid transport system ATP-binding protein
AVI_01504	K01997	livH; branched-chain amino acid transport system permease protein
AVI_01505	K01998	livM; branched-chain amino acid transport system permease protein
AVI_01506	K00598	tam; trans-aconitate 2-methyltransferase [EC:2.1.1.144]
AVI_01507	K00020	HIBADH, mmsB; 3-hydroxyisobutyrate dehydrogenase [EC:1.1.1.31]
AVI_01508	K01434	pac; penicillin G amidase [EC:3.5.1.11]
AVI_01510	K03665	hflX; GTPase
AVI_01511	K03666	hfq; host factor-I protein
AVI_01512	K03498	trkH, trkG, ktrB, ktrD; trk/ktr system potassium uptake protein
AVI_01513	K03499	trkA, ktrA, ktrC; trk/ktr system potassium uptake protein
AVI_01515	K13599	ntrX; two-component system, NtrC family, nitrogen regulation response regulator NtrX
AVI_01516	K13598	ntrY; two-component system, NtrC family, nitrogen regulation sensor histidine kinase NtrY [EC:2.7.13.3]
AVI_01517	K07712	glnG, ntrC; two-component system, NtrC family, nitrogen regulation response regulator GlnG
AVI_01518	K07708	glnL, ntrB; two-component system, NtrC family, nitrogen regulation sensor histidine kinase GlnL [EC:2.7.13.3]
AVI_01519	K05540	dusB; tRNA-dihydrouridine synthase B [EC:1.-.-.-]
AVI_01520	K12506	ispDF; 2-C-methyl-D-erythritol 4-phosphate cytidylyltransferase / 2-C-methyl-D-erythritol 2,4-cyclodiphosphate synthase [EC:2.7.7.60 4.6.1.12]
AVI_01521	K01095	pgpA; phosphatidylglycerophosphatase A [EC:3.1.3.27]
AVI_01522	K03743	pncC; nicotinamide-nucleotide amidase [EC:3.5.1.42]
AVI_01523	K18588	COQ10; coenzyme Q-binding protein COQ10
AVI_01524	K00760	hprT, hpt, HPRT1; hypoxanthine phosphoribosyltransferase [EC:2.4.2.8]
AVI_01528	K03644	lipA, LIAS, LIP1, LIP5; lipoyl synthase [EC:2.8.1.8]
AVI_01532	K14083	mttB; trimethylamine---corrinoid protein Co-methyltransferase [EC:2.1.1.250]
AVI_01534	K01011	TST, MPST, sseA; thiosulfate/3-mercaptopyruvate sulfurtransferase [EC:2.8.1.1 2.8.1.2]
AVI_01535	K01951	guaA, GMPS; GMP synthase (glutamine-hydrolysing) [EC:6.3.5.2]
AVI_01538	K06076	fadL; long-chain fatty acid transport protein
AVI_01541	K09457	queF; 7-cyano-7-deazaguanine reductase [EC:1.7.1.13]

AVI_01544	K02434	gatB, PET112; aspartyl-tRNA(Asn)/glutamyl-tRNA(Gln) amidotransferase subunit B [EC:6.3.5.6 6.3.5.7]
AVI_01549	K05527	bolA; BolA family transcriptional regulator, general stress-responsive regulator
AVI_01554	K09882	cobS; cobaltochelatase CobS [EC:6.6.1.2]
AVI_01557	K09883	cobT; cobaltochelatase CobT [EC:6.6.1.2]
AVI_01560	K00010	iolG; myo-inositol 2-dehydrogenase / D-chiro-inositol 1-dehydrogenase [EC:1.1.1.18 1.1.1.369]
AVI_01561	K03710	K03710; GntR family transcriptional regulator
AVI_01562	K26058	smoC, squD; sulfoquinovose monooxygenase [EC:1.14.14.181]
AVI_01563	K26057	smoB, squF; 6-dehydroglucose reductase [EC:1.1.1.432]
AVI_01568	K06048	gshA, ybdK; glutamate---cysteine ligase / carboxylate-amine ligase [EC:6.3.2.2 6.3.-.-]
AVI_01572	K01805	xylA; xylose isomerase [EC:5.3.1.5]
AVI_01573	K00854	xylB, XYLB; xylulokinase [EC:2.7.1.17]
AVI_01575	K10545	xylG; D-xylose transport system ATP-binding protein [EC:7.5.2.10]
AVI_01576	K10544	xylH; D-xylose transport system permease protein
AVI_01577	K10543	xylF; D-xylose transport system substrate-binding protein
AVI_01579	K21162	sgcE4, ncsE4, kedE4; enediyne biosynthesis protein E4
AVI_01581	K07215	pigA, hemO; heme oxygenase (biliverdin-IX-beta and delta-forming) [EC:1.14.99.58]
AVI_01583	K14160	imuA; protein ImuA
AVI_01584	K14161	imuB; protein ImuB
AVI_01585	K14162	dnaE2; error-prone DNA polymerase [EC:2.7.7.7]
AVI_01589	K10111	malK, mtlK, thuK; multiple sugar transport system ATP-binding protein [EC:7.5.2.-]
AVI_01591	K10119	msmG; raffinose/stachyose/melibiose transport system permease protein
AVI_01592	K10118	msmF; raffinose/stachyose/melibiose transport system permease protein
AVI_01593	K10117	msmE; raffinose/stachyose/melibiose transport system substrate-binding protein
AVI_01594	K07106	murQ; N-acetylmuramic acid 6-phosphate etherase [EC:4.2.1.126]
AVI_01595	K18676	gspK; glucosamine kinase [EC:2.7.1.8]
AVI_01596	K24967	nagR; GntR family transcriptional regulator, N-acetylglucosamine utilization regulator
AVI_01597	K00820	glmS, GFPT; glutamine---fructose-6-phosphate transaminase (isomerizing) [EC:2.6.1.16]
AVI_01598	K01443	nagA, AMDHD2; N-acetylglucosamine-6-phosphate deacetylase [EC:3.5.1.25]
AVI_01599	K18480	linN; cholesterol transport system auxiliary component
AVI_01600	K02067	mlaD, linM; phospholipid/cholesterol/gamma-HCH transport system substrate-binding protein
AVI_01601	K02065	mlaF, linL, mkl; phospholipid/cholesterol/gamma-HCH transport system ATP-binding protein
AVI_01602	K02066	mlaE, linK; phospholipid/cholesterol/gamma-HCH transport system permease protein
AVI_01603	K01420	fnr; CRP/FNR family transcriptional regulator, anaerobic regulatory protein
AVI_01604	K22736	VIT; vacuolar iron transporter family protein
AVI_01605	K01537	ATP2C; P-type Ca2+ transporter type 2C [EC:7.2.2.10]
AVI_01607	K03821	phaC, phbC; poly[(R)-3-hydroxyalkanoate] polymerase subunit PhaC [EC:2.3.1.304]
AVI_01608	K02005	ABC.CD.TX; HlyD family secretion protein
AVI_01609	K02004	ABC.CD.P; putative ABC transport system permease protein
AVI_01610	K02003	ABC.CD.A; putative ABC transport system ATP-binding protein
AVI_01611	K13953	adhP; alcohol dehydrogenase, propanol-preferring [EC:1.1.1.1]
AVI_01612	K14986	fixL; two-component system, LuxR family, sensor kinase FixL [EC:2.7.13.3]
AVI_01613	K14987	fixJ; two-component system, LuxR family, response regulator FixJ

AVI_01615	K13993	HSP20; HSP20 family protein
AVI_01620	K23536	nupC; general nucleoside transport system permease protein
AVI_01621	K23535	nupB; general nucleoside transport system permease protein
AVI_01622	K23537	nupA; general nucleoside transport system ATP-binding protein
AVI_01623	K07335	bmpA, bmpB, tmpC; basic membrane protein A and related proteins
AVI_01624	K01895	ACSS1_2, acs; acetyl-CoA synthetase [EC:6.2.1.1]
AVI_01626	K00627	DLAT, aceF, pdhC; pyruvate dehydrogenase E2 component (dihydrolipoyllysine-residue acetyltransferase) [EC:2.3.1.12]
AVI_01627	K02078	acpP; acyl carrier protein
AVI_01634	K17229	fccB; sulfide dehydrogenase [flavocytochrome c] flavoprotein chain [EC:1.8.2.3]
AVI_01635	K08738	CYC; cytochrome c
AVI_01638	K22622	soxD; S-disulfanyl-L-cysteine oxidoreductase SoxD [EC:1.8.2.6]
AVI_01639	K17225	soxC; sulfane dehydrogenase subunit SoxC
AVI_01640	K17224	soxB; S-sulfosulfanyl-L-cysteine sulfohydrolase [EC:3.1.6.20]
AVI_01641	K17222	soxA; L-cysteine S-thiosulfotransferase [EC:2.8.5.2]
AVI_01642	K17227	soxZ; sulfur-oxidizing protein SoxZ
AVI_01643	K17226	soxY; sulfur-oxidizing protein SoxY
AVI_01644	K17223	soxX; L-cysteine S-thiosulfotransferase [EC:2.8.5.2]
AVI_01646	K06196	ccdA; cytochrome c-type biogenesis protein
AVI_01649	K07112	K07112; uncharacterized protein
AVI_01650	K04034	bchE; anaerobic magnesium-protoporphyrin IX monomethyl ester cyclase [EC:1.21.98.3]
AVI_01651	K04036	bchJ; divinyl protochlorophyllide a 8-vinyl-reductase [EC:1.-.-.-]
AVI_01654	K07050	AARSD1, ALAX; misacylated tRNA(Ala) deacylase [EC:3.1.1.-]
AVI_01655	K05802	mscK, kefA, aefA; potassium-dependent mechanosensitive channel
AVI_01656	K01738	cysK; cysteine synthase [EC:2.5.1.47]
AVI_01659	K00574	cfa; cyclopropane-fatty-acyl-phospholipid synthase [EC:2.1.1.79]
AVI_01661	K01669	phr, PHR1; deoxyribodipyrimidine photo-lyase [EC:4.1.99.3]
AVI_01662	K19745	acuI; acrylyl-CoA reductase (NADPH) [EC:1.3.1.-]
AVI_01663	K17486	dmdA; dimethylsulfoniopropionate demethylase [EC:2.1.1.269]
AVI_01670	K07240	chrA; chromate transporter
AVI_01671	K09985	K09985; uncharacterized protein
AVI_01673	K01679	E4.2.1.2B, fumC, FH; fumarate hydratase, class II [EC:4.2.1.2]
AVI_01676	K07657	phoB; two-component system, OmpR family, phosphate regulon response regulator PhoB
AVI_01677	K02039	phoU; phosphate transport system protein
AVI_01678	K02036	pstB; phosphate transport system ATP-binding protein [EC:7.3.2.1]
AVI_01679	K02038	pstA; phosphate transport system permease protein
AVI_01680	K02037	pstC; phosphate transport system permease protein
AVI_01681	K02040	pstS; phosphate transport system substrate-binding protein
AVI_01682	K07636	phoR; two-component system, OmpR family, phosphate regulon sensor histidine kinase PhoR [EC:2.7.13.3]
AVI_01687	K07793	tctA; putative tricarboxylic transport membrane protein
AVI_01691	K01153	hsdR; type I restriction enzyme, R subunit [EC:3.1.21.3]
AVI_01692	K03427	hsdM; type I restriction enzyme M protein [EC:2.1.1.72]
AVI_01696	K07154	hipA; serine/threonine-protein kinase HipA [EC:2.7.11.1]

AVI_01700	K03497	parB, spo0J; ParB family transcriptional regulator, chromosome partitioning protein
AVI_01704	K00059	fabG, OAR1; 3-oxoacyl-[acyl-carrier protein] reductase [EC:1.1.1.100]
AVI_01711	K01118	acpD, azoR; FMN-dependent NADH-azoreductase [EC:1.7.1.17]
AVI_01722	K03205	virD4, lvhD4; type IV secretion system protein VirD4 [EC:7.4.2.8]
AVI_01724	K20527	trbB; type IV secretion system protein TrbB [EC:7.4.2.8]
AVI_01725	K20528	trbC; type IV secretion system protein TrbC
AVI_01726	K20529	trbD; type IV secretion system protein TrbD
AVI_01727	K20530	trbE; type IV secretion system protein TrbE [EC:7.4.2.8]
AVI_01728	K20266	trbJ; type IV secretion system protein TrbJ
AVI_01730	K07344	trbL; type IV secretion system protein TrbL
AVI_01731	K20531	trbF; type IV secretion system protein TrbF
AVI_01732	K20532	trbG; type IV secretion system protein TrbG
AVI_01733	K20533	trbI; type IV secretion system protein TrbI
AVI_01738	K06893	K06893; uncharacterized protein
AVI_01739	K06988	fno; 8-hydroxy-5-deazaflavin:NADPH oxidoreductase [EC:1.5.1.40]
AVI_01743	K06893	K06893; uncharacterized protein
AVI_01750	K07506	K07506; AraC family transcriptional regulator
AVI_01754	K11717	sufS; cysteine desulfurase / selenocysteine lyase [EC:2.8.1.7 4.4.1.16]
AVI_01756	K09015	sufD; Fe-S cluster assembly protein SufD
AVI_01757	K09013	sufC; Fe-S cluster assembly ATP-binding protein
AVI_01764	K09014	sufB; Fe-S cluster assembly protein SufB
AVI_01765	K04487	iscS, NFS1; cysteine desulfurase [EC:2.8.1.7]
AVI_01766	K13643	iscR; Rrf2 family transcriptional regulator, iron-sulfur cluster assembly transcription factor
AVI_01767	K07018	K07018; uncharacterized protein
AVI_01768	K22479	argA; N-acetyltransferase
AVI_01770	K00031	IDH1, IDH2, icd; isocitrate dehydrogenase [EC:1.1.1.42]
AVI_01773	K01607	pcaC; 4-carboxymuconolactone decarboxylase [EC:4.1.1.44]
AVI_01777	K03534	rhaM; L-rhamnose mutarotase [EC:5.1.3.32]
AVI_01778	K10561	rhaQ; rhamnose transport system permease protein
AVI_01779	K10560	rhaP; rhamnose transport system permease protein
AVI_01780	K10562	rhaT; rhamnose transport system ATP-binding protein [EC:7.5.2.-]
AVI_01781	K10559	rhaS; rhamnose transport system substrate-binding protein
AVI_01784	K01813	rhaA; L-rhamnose isomerase [EC:5.3.1.14]
AVI_01793	K07302	iorA; isoquinoline 1-oxidoreductase subunit alpha [EC:1.3.99.16]
AVI_01794	K07303	iorB; isoquinoline 1-oxidoreductase subunit beta [EC:1.3.99.16]
AVI_01796	K07183	nasT; two-component system, response regulator / RNA-binding antiterminator
AVI_01797	K22067	nasS; two-component system, oxyanion-binding sensor
AVI_01798	K15576	nrtA, nasF, cynA; nitrate/nitrite transport system substrate-binding protein
AVI_01799	K15577	nrtB, nasE, cynB; nitrate/nitrite transport system permease protein
AVI_01802	K26139	nasD, nasB; nitrite reductase [NAD(P)H] large subunit [EC:1.7.1.4]
AVI_01803	K26138	nasE; nitrite reductase [NAD(P)H] small subunit [EC:1.7.1.4]
AVI_01804	K00372	nasC, nasA; assimilatory nitrate reductase catalytic subunit [EC:1.7.99.-]

AVI_01805	K02302	cysG; uroporphyrin-III C-methyltransferase / precorrin-2 dehydrogenase / sirohydrochlorin ferrochelatase [EC:2.1.1.107 1.3.1.76 4.99.1.4]
AVI_01808	K21395	yiaO; TRAP-type transport system periplasmic protein
AVI_01813	K00573	E2.1.1.77, pcm; protein-L-isoaspartate(D-aspartate) O-methyltransferase [EC:2.1.1.77]
AVI_01814	K12340	tolC, bepC, cyaE, raxC, sapF, rsaF, hasF; outer membrane protein
AVI_01816	K02232	cobQ, cbiP; adenosylcobyric acid synthase [EC:6.3.5.10]
AVI_01819	K02356	efp; elongation factor P
AVI_01821	K06980	ygfZ; tRNA-modifying protein YgfZ
AVI_01822	K11085	msbA; ATP-binding cassette, subfamily B, bacterial MsbA [EC:7.5.2.6]
AVI_01824	K01092	E3.1.3.25, IMPA, suhB; myo-inositol-1(or 4)-monophosphatase [EC:3.1.3.25]
AVI_01827	K07059	K07059; rhomboid family protein
AVI_01833	K01874	MARS, metG; methionyl-tRNA synthetase [EC:6.1.1.10]
AVI_01835	K03098	APOD; apolipoprotein D and lipocalin family protein
AVI_01842	K03088	rpoE; RNA polymerase sigma-70 factor, ECF subfamily
AVI_01845	K02488	pleD; two-component system, cell cycle response regulator [EC:2.7.7.65]
AVI_01847	K14083	mttB; trimethylamine---corrinoid protein Co-methyltransferase [EC:2.1.1.250]
AVI_01849	K21420	bpt; leucyl-tRNA---protein transferase [EC:2.3.2.29]
AVI_01852	K00982	glnE; [glutamine synthetase] adenylyltransferase / [glutamine synthetase]-adenylyl-L-tyrosine phosphorylase [EC:2.7.7.42 2.7.7.89]
AVI_01854	K01625	eda; 2-dehydro-3-deoxyphosphogluconate aldolase / (4S)-4-hydroxy-2-oxoglutarate aldolase [EC:4.1.2.14 4.1.3.42]
AVI_01855	K07006	K07006; uncharacterized protein
AVI_01859	K01690	edd; phosphogluconate dehydratase [EC:4.2.1.12]
AVI_01860	K01754	E4.3.1.19, ilvA, tdcB; threonine dehydratase [EC:4.3.1.19]
AVI_01863	K06969	rlmI; 23S rRNA (cytosine1962-C5)-methyltransferase [EC:2.1.1.191]
AVI_01865	K07124	K07124; uncharacterized protein
AVI_01870	K05539	dusA; tRNA-dihydrouridine synthase A [EC:1.-.-.-]
AVI_01873	K06938	K06938; uncharacterized protein
AVI_01874	K07447	ruvX; putative pre-16S rRNA nuclease [EC:3.1.-.-]
AVI_01875	K02200	ccmH; cytochrome c-type biogenesis protein CcmH
AVI_01876	K00303	soxB; sarcosine oxidase, subunit beta [EC:1.5.3.24 1.5.3.1]
AVI_01879	K00304	soxD; sarcosine oxidase, subunit delta [EC:1.5.3.24 1.5.3.1]
AVI_01880	K00302	soxA; sarcosine oxidase, subunit alpha [EC:1.5.3.24 1.5.3.1]
AVI_01881	K00305	soxG; sarcosine oxidase, subunit gamma [EC:1.5.3.24 1.5.3.1]
AVI_01884	K04564	SOD2; superoxide dismutase, Fe-Mn family [EC:1.15.1.1]
AVI_01887	K24821	atm1, pexA; ATP-binding cassette, subfamily B, heavy metal transporter
AVI_01888	K07266	kpsC, lipA; capsular polysaccharide export protein
AVI_01889	K07265	kpsS, lipB; capsular polysaccharide export protein
AVI_01890	K00793	ribE, RIB5; riboflavin synthase [EC:2.5.1.9]
AVI_01892	K14652	ribBA; 3,4-dihydroxy 2-butanone 4-phosphate synthase / GTP cyclohydrolase II [EC:4.1.99.12 3.5.4.25]
AVI_01893	K00794	ribH, RIB4; 6,7-dimethyl-8-ribityllumazine synthase [EC:2.5.1.78]
AVI_01894	K03625	nusB; transcription antitermination protein NusB
AVI_01899	K25911	pssJ; exopolysaccharide biosynthesis galactosyltransferase PssJ
AVI_01905	K06180	rluD; 23S rRNA pseudouridine1911/1915/1917 synthase [EC:5.4.99.23]
AVI_01906	K03089	rpoH; RNA polymerase sigma-32 factor

AVI_01914	K09815	znuA; zinc transport system substrate-binding protein
AVI_01915	K09823	zur; Fur family transcriptional regulator, zinc uptake regulator
AVI_01916	K09817	znuC; zinc transport system ATP-binding protein [EC:7.2.2.20]
AVI_01917	K09816	znuB; zinc transport system permease protein
AVI_01926	K10914	crp; CRP/FNR family transcriptional regulator, cyclic AMP receptor protein
AVI_01930	K10232	aglE, ggtB; alpha-glucoside transport system substrate-binding protein
AVI_01934	K03932	lpqC; polyhydroxybutyrate depolymerase
AVI_01935	K01915	glnA, GLUL; glutamine synthetase [EC:6.3.1.2]
AVI_01936	K04751	glnB; nitrogen regulatory protein P-II 1
AVI_01938	K00645	fabD, MCAT, MCT1; [acyl-carrier-protein] S-malonyltransferase [EC:2.3.1.39]
AVI_01939	K00059	fabG, OAR1; 3-oxoacyl-[acyl-carrier protein] reductase [EC:1.1.1.100]
AVI_01940	K02078	acpP; acyl carrier protein
AVI_01943	K09458	fabF, OXSM, CEM1; 3-oxoacyl-[acyl-carrier-protein] synthase II [EC:2.3.1.179]
AVI_01944	K07082	mltG; peptidoglycan lytic transglycosylase G [EC:4.2.2.29]
AVI_01948	K03593	mrp, NUBPL; ATP-binding protein involved in chromosome partitioning
AVI_01952	K01243	mtnN, mtn, pfs; adenosylhomocysteine nucleosidase [EC:3.2.2.9]
AVI_01961	K08678	UXS1, uxs; UDP-glucuronate decarboxylase [EC:4.1.1.35]
AVI_01965	K00655	plsC; 1-acyl-sn-glycerol-3-phosphate acyltransferase [EC:2.3.1.51]
AVI_01966	K02034	ABC.PE.P1; peptide/nickel transport system permease protein
AVI_01967	K02033	ABC.PE.P; peptide/nickel transport system permease protein
AVI_01968	K02035	ABC.PE.S; peptide/nickel transport system substrate-binding protein
AVI_01969	K02032	ddpF; peptide/nickel transport system ATP-binding protein
AVI_01970	K15583	oppD; oligopeptide transport system ATP-binding protein
AVI_01971	K01438	argE; acetylornithine deacetylase [EC:3.5.1.16]
AVI_01974	K26939	norM, mdtK; MATE family, multidrug efflux pump
AVI_01980	K06904	GP4; Escherichia/Staphylococcus phage prohead protease
AVI_01994	K06204	dksA; RNA polymerase-binding transcription factor
AVI_01995	K00640	cysE; serine O-acetyltransferase [EC:2.3.1.30]
AVI_01997	K00981	E2.7.7.41, CDS1, CDS2, cdsA; phosphatidate cytidylyltransferase [EC:2.7.7.41]
AVI_01998	K00995	pgsA, PGS1; CDP-diacylglycerol---glycerol-3-phosphate 3-phosphatidyltransferase [EC:2.7.8.5]
AVI_01999	K00627	DLAT, aceF, pdhC; pyruvate dehydrogenase E2 component (dihydrolipoyllysine-residue acetyltransferase) [EC:2.3.1.12]
AVI_02002	K00162	PDHB, pdhB; pyruvate dehydrogenase E1 component subunit beta [EC:1.2.4.1]
AVI_02004	K00161	PDHA, pdhA; pyruvate dehydrogenase E1 component subunit alpha [EC:1.2.4.1]
AVI_02007	K09456	aidB; putative acyl-CoA dehydrogenase
AVI_02008	K11645	fbaB; fructose-bisphosphate aldolase, class I [EC:4.1.2.13]
AVI_02009	K00927	PGK, pgk; phosphoglycerate kinase [EC:2.7.2.3]
AVI_02014	K01866	YARS, tyrS; tyrosyl-tRNA synthetase [EC:6.1.1.1]
AVI_02015	K09001	anmK; anhydro-N-acetylmuramic acid kinase [EC:2.7.1.170]
AVI_02017	K01653	E2.2.1.6S, ilvH, ilvN; acetolactate synthase I/III small subunit [EC:2.2.1.6]
AVI_02018	K01652	E2.2.1.6L, ilvB, ilvG, ilvI; acetolactate synthase I/II/III large subunit [EC:2.2.1.6]
AVI_02026	K07246	ttuC, dmlA; tartrate dehydrogenase/decarboxylase / D-malate dehydrogenase [EC:1.1.1.93 4.1.1.73 1.1.1.83]
AVI_02027	K01803	TPI, tpiA; triosephosphate isomerase (TIM) [EC:5.3.1.1]

AVI_02029	K13628	iscA; iron-sulfur cluster assembly protein
AVI_02030	K07454	K07454; putative restriction endonuclease
AVI_02031	K00661	maa; maltose O-acetyltransferase [EC:2.3.1.79]
AVI_02037	K01126	E3.1.4.46, glpQ, ugpQ; glycerophosphoryl diester phosphodiesterase [EC:3.1.4.46]
AVI_02040	K00773	tgt; queuine tRNA-ribosyltransferase [EC:2.4.2.29]
AVI_02042	K01338	lon; ATP-dependent Lon protease [EC:3.4.21.53]
AVI_02046	K02314	dnaB; replicative DNA helicase [EC:5.6.2.3]
AVI_02048	K00762	pyrE; orotate phosphoribosyltransferase [EC:2.4.2.10]
AVI_02049	K01465	URA4, pyrC; dihydroorotase [EC:3.5.2.3]
AVI_02052	K00252	GCDH, gcdH; glutaryl-CoA dehydrogenase [EC:1.3.8.6]
AVI_02055	K07161	K07161; uncharacterized protein
AVI_02057	K07402	xdhC; xanthine dehydrogenase accessory factor
AVI_02058	K07141	mocA; molybdenum cofactor cytidylyltransferase [EC:2.7.7.76]
AVI_02062	K02520	infC, MTIF3; translation initiation factor IF-3
AVI_02064	K00528	fpr; ferredoxin/flavodoxin---NADP+ reductase [EC:1.18.1.2 1.19.1.1]
AVI_02066	K00390	cysH; phosphoadenosine phosphosulfate reductase [EC:1.8.4.8 1.8.4.10]
AVI_02067	K00381	cysI; sulfite reductase (NADPH) hemoprotein beta-component [EC:1.8.1.2]
AVI_02069	K02302	cysG; uroporphyrin-III C-methyltransferase / precorrin-2 dehydrogenase / sirohydrochlorin ferrochelatase [EC:2.1.1.107 1.3.1.76 4.99.1.4]
AVI_02070	K05800	decR, cyuR; Lrp/AsnC family transcriptional regulator, cysteine-sensing transcriptional activator
AVI_02071	K07089	K07089; uncharacterized protein
AVI_02073	K02040	pstS; phosphate transport system substrate-binding protein
AVI_02076	K11927	rhlE; ATP-dependent RNA helicase RhlE [EC:5.6.2.7]
AVI_02077	K00928	lysC; aspartate kinase [EC:2.7.2.4]
AVI_02081	K04768	acuC; acetoin utilization protein AcuC
AVI_02082	K02527	kdtA, waaA; 3-deoxy-D-manno-octulosonic-acid transferase [EC:2.4.99.12 2.4.99.13 2.4.99.14 2.4.99.15]
AVI_02083	K02020	modA; molybdate transport system substrate-binding protein
AVI_02084	K02018	modB; molybdate transport system permease protein
AVI_02085	K02017	modC; molybdate transport system ATP-binding protein [EC:7.3.2.5]
AVI_02091	K23269	purL; phosphoribosylformylglycinamidine synthase subunit PurL [EC:6.3.5.3]
AVI_02094	K04090	E1.2.7.8; indolepyruvate ferredoxin oxidoreductase [EC:1.2.7.8]
AVI_02096	K01776	murI; glutamate racemase [EC:5.1.1.3]
AVI_02097	K00145	argC; N-acetyl-gamma-glutamyl-phosphate reductase [EC:1.2.1.38]
AVI_02098	K02197	ccmE; cytochrome c-type biogenesis protein CcmE
AVI_02101	K02198	ccmF; cytochrome c-type biogenesis protein CcmF
AVI_02102	K02200	ccmH; cytochrome c-type biogenesis protein CcmH
AVI_02103	K15866	paaG; 2-(1,2-epoxy-1,2-dihydrophenyl)acetyl-CoA isomerase [EC:5.3.3.18]
AVI_02106	K01647	CS, gltA; citrate synthase [EC:2.3.3.1]
AVI_02107	K01885	EARS, gltX; glutamyl-tRNA synthetase [EC:6.1.1.17]
AVI_02108	K02238	comEC; competence protein ComEC
AVI_02109	K01356	lexA; repressor LexA [EC:3.4.21.88]
AVI_02110	K03750	moeA; molybdopterin molybdotransferase [EC:2.10.1.1]
AVI_02111	K03637	moaC, CNX3; cyclic pyranopterin monophosphate synthase [EC:4.6.1.17]

AVI_02112	K01609	trpC; indole-3-glycerol phosphate synthase [EC:4.1.1.48]
AVI_02113	K00766	trpD; anthranilate phosphoribosyltransferase [EC:2.4.2.18]
AVI_02114	K01658	trpG; anthranilate synthase component II [EC:4.1.3.27]
AVI_02119	K01657	trpE; anthranilate synthase component I [EC:4.1.3.27]
AVI_02120	K03770	ppiD; peptidyl-prolyl cis-trans isomerase D [EC:5.2.1.8]
AVI_02123	K00769	gpt; xanthine phosphoribosyltransferase [EC:2.4.2.22]
AVI_02124	K05835	rhtC; threonine efflux protein
AVI_02125	K05835	rhtC; threonine efflux protein
AVI_02126	K00208	fabI; enoyl-[acyl-carrier protein] reductase I [EC:1.3.1.9 1.3.1.10]
AVI_02127	K00275	pdxH, PNPO; pyridoxamine 5'-phosphate oxidase [EC:1.4.3.5]
AVI_02128	K03704	cspA; cold shock protein
AVI_02129	K09005	K09005; uncharacterized protein
AVI_02133	K00525	E1.17.4.1A, nrdA, nrdE; ribonucleoside-diphosphate reductase alpha chain [EC:1.17.4.1]
AVI_02140	K07231	K07231; putative iron-regulated protein
AVI_02143	K07338	K07338; uncharacterized protein
AVI_02144	K09947	K09947; uncharacterized protein
AVI_02145	K07222	K07222; putative flavoprotein involved in K+ transport
AVI_02155	K05800	decR, cyuR; Lrp/AsnC family transcriptional regulator, cysteine-sensing transcriptional activator
AVI_02157	K00088	IMPDH, guaB; IMP dehydrogenase [EC:1.1.1.205]
AVI_02158	K03500	rsmB, sun; 16S rRNA (cytosine967-C5)-methyltransferase [EC:2.1.1.176]
AVI_02159	K13587	cckA; two-component system, cell cycle sensor histidine kinase and response regulator CckA [EC:2.7.13.3]
AVI_02161	K03553	recA; recombination protein RecA
AVI_02163	K01872	AARS, alaS; alanyl-tRNA synthetase [EC:6.1.1.7]
AVI_02164	K06207	typA, bipA; GTP-binding protein
AVI_02167	K03545	tig; trigger factor
AVI_02172	K01007	pps, ppsA; pyruvate, water dikinase [EC:2.7.9.2]
AVI_02173	K00016	LDH, ldh; L-lactate dehydrogenase [EC:1.1.1.27]
AVI_02175	K16370	pfkB; 6-phosphofructokinase 2 [EC:2.7.1.11]
AVI_02176	K02939	RP-L9, MRPL9, rplI; large subunit ribosomal protein L9
AVI_02177	K02963	RP-S18, MRPS18, rpsR; small subunit ribosomal protein S18
AVI_02178	K02990	RP-S6, MRPS6, rpsF; small subunit ribosomal protein S6
AVI_02183	K01455	E3.5.1.49; formamidase [EC:3.5.1.49]
AVI_02184	K10536	aguA; agmatine deiminase [EC:3.5.3.12]
AVI_02204	K09794	K09794; uncharacterized protein
AVI_02205	K07491	rayT; REP-associated tyrosine transposase
AVI_02208	K15783	doeA; ectoine hydrolase [EC:3.5.4.44]
AVI_02210	K00986	ltrA; RNA-directed DNA polymerase [EC:2.7.7.49]
AVI_02212	K00969	nadD; nicotinate-nucleotide adenylyltransferase [EC:2.7.7.18]
AVI_02213	K07259	dacB; serine-type D-Ala-D-Ala carboxypeptidase/endopeptidase (penicillin-binding protein 4) [EC:3.4.16.4 3.4.21.-]
AVI_02217	K04566	lysK; lysyl-tRNA synthetase, class I [EC:6.1.1.6]
AVI_02220	K03190	ureD, ureH; urease accessory protein
AVI_02221	K01430	ureA; urease subunit gamma [EC:3.5.1.5]

AVI_02223	K01429	ureB; urease subunit beta [EC:3.5.1.5]
AVI_02225	K01428	ureC; urease subunit alpha [EC:3.5.1.5]
AVI_02226	K03187	ureE; urease accessory protein
AVI_02227	K03188	ureF; urease accessory protein
AVI_02228	K03189	ureG; urease accessory protein
AVI_02232	K22922	vexD; Vi polysaccharide transport system permease protein
AVI_02233	K09689	kpsT; capsular polysaccharide transport system ATP-binding protein [EC:7.6.2.12]
AVI_02234	K22921	vexB; Vi polysaccharide transport system permease protein
AVI_02235	K08679	GAE, cap1J; UDP-glucuronate 4-epimerase [EC:5.1.3.6]
AVI_02238	K02474	wbpO; UDP-N-acetyl-D-glucosamine/UDP-N-acetyl-D-galactosamine dehydrogenase [EC:1.1.1.136 1.1.1.-]
AVI_02244	K01652	E2.2.1.6L, ilvB, ilvG, ilvI; acetolactate synthase I/II/III large subunit [EC:2.2.1.6]
AVI_02245	K00135	gabD; succinate-semialdehyde dehydrogenase / glutarate-semialdehyde dehydrogenase [EC:1.2.1.16 1.2.1.79 1.2.1.20]
AVI_02246	K13796	cobZ, tcuA; tricarballylate dehydrogenase
AVI_02247	K13795	citB, tcuB; citrate/tricarballylate utilization protein
AVI_02249	K07793	tctA; putative tricarboxylic transport membrane protein
AVI_02251	K07774	tctD; two-component system, OmpR family, response regulator TctD
AVI_02252	K07649	tctE; two-component system, OmpR family, sensor histidine kinase TctE [EC:2.7.13.3]
AVI_02253	K07120	K07120; uncharacterized protein
AVI_02254	K01711	gmd, GMDS; GDPmannose 4,6-dehydratase [EC:4.2.1.47]
AVI_02259	K10126	dctD; two-component system, NtrC family, C4-dicarboxylate transport response regulator DctD
AVI_02260	K10125	dctB; two-component system, NtrC family, C4-dicarboxylate transport sensor histidine kinase DctB [EC:2.7.13.3]
AVI_02262	K07080	K07080; uncharacterized protein
AVI_02266	K10107	kpsE; capsular polysaccharide transport system permease protein
AVI_02267	K09689	kpsT; capsular polysaccharide transport system ATP-binding protein [EC:7.6.2.12]
AVI_02268	K03313	nhaA; Na+:H+ antiporter, NhaA family
AVI_02273	K12972	ghrA; glyoxylate/hydroxypyruvate reductase [EC:1.1.1.79 1.1.1.81]
AVI_02274	K05837	rodA, mrdB; rod shape determining protein RodA
AVI_02275	K05515	mrdA; penicillin-binding protein 2 [EC:3.4.16.4]
AVI_02276	K03571	mreD; rod shape-determining protein MreD
AVI_02277	K03570	mreC; rod shape-determining protein MreC
AVI_02278	K03569	mreB; rod shape-determining protein MreB and related proteins
AVI_02280	K01649	leuA, IMS; 2-isopropylmalate synthase [EC:2.3.3.13]
AVI_02283	K06876	phrB; (6-4)DNA photolyase [EC:4.1.99.13]
AVI_02285	K01916	nadE; NAD+ synthase [EC:6.3.1.5]
AVI_02291	K01885	EARS, gltX; glutamyl-tRNA synthetase [EC:6.1.1.17]
AVI_02294	K07232	CHAC, chaC; glutathione-specific gamma-glutamylcyclotransferase [EC:4.3.2.7]
AVI_02296	K02557	motB; chemotaxis protein MotB
AVI_02297	K15986	ppaC; manganese-dependent inorganic pyrophosphatase [EC:3.6.1.1]
AVI_02299	K17865	croR; 3-hydroxybutyryl-CoA dehydratase [EC:4.2.1.55]
AVI_02300	K11753	ribF; riboflavin kinase / FMN adenylyltransferase [EC:2.7.1.26 2.7.7.2]
AVI_02301	K09160	K09160; uncharacterized protein
AVI_02302	K01620	ltaE; threonine aldolase [EC:4.1.2.48]

AVI_02305	K00948	PRPS, prsA; ribose-phosphate pyrophosphokinase [EC:2.7.6.1]
AVI_02307	K02114	ATPF1E, atpC; F-type H+-transporting ATPase subunit epsilon
AVI_02308	K02112	ATPF1B, atpD; F-type H+/Na+-transporting ATPase subunit beta [EC:7.1.2.2 7.2.2.1]
AVI_02309	K02115	ATPF1G, atpG; F-type H+-transporting ATPase subunit gamma
AVI_02310	K02111	ATPF1A, atpA; F-type H+/Na+-transporting ATPase subunit alpha [EC:7.1.2.2 7.2.2.1]
AVI_02311	K02113	ATPF1D, atpH; F-type H+-transporting ATPase subunit delta
AVI_02314	K01069	gloB, gloC, HAGH; hydroxyacylglutathione hydrolase [EC:3.1.2.6]
AVI_02315	K03694	clpA; ATP-dependent Clp protease ATP-binding subunit ClpA
AVI_02318	K09973	K09973; uncharacterized protein
AVI_02319	K04078	groES, HSPE1; chaperonin GroES
AVI_02320	K04077	groEL, HSPD1; chaperonin GroEL [EC:5.6.1.7]
AVI_02325	K03574	mutT, NUDT15, MTH2; 8-oxo-dGTP diphosphatase [EC:3.6.1.55]
AVI_02330	K06203	cysZ; CysZ protein
AVI_02332	K05606	MCEE, epi; methylmalonyl-CoA/ethylmalonyl-CoA epimerase [EC:5.1.99.1]
AVI_02336	K01876	DARS2, aspS; aspartyl-tRNA synthetase [EC:6.1.1.12]
AVI_02337	K00574	cfa; cyclopropane-fatty-acyl-phospholipid synthase [EC:2.1.1.79]
AVI_02339	K01955	carB, CPA2; carbamoyl-phosphate synthase large subunit [EC:6.3.5.5]
AVI_02340	K16872	E2.3.1.207; beta-ketodecanoyl-[acyl-carrier-protein] synthase [EC:2.3.1.207]
AVI_02341	K11811	arsH; arsenical resistance protein ArsH
AVI_02342	K00857	tdk, TK; thymidine kinase [EC:2.7.1.21]
AVI_02343	K01589	purK; 5-(carboxyamino)imidazole ribonucleotide synthase [EC:6.3.4.18]
AVI_02344	K01588	purE; 5-(carboxyamino)imidazole ribonucleotide mutase [EC:5.4.99.18]
AVI_02348	K00958	sat, met3; sulfate adenylyltransferase [EC:2.7.7.4]
AVI_02349	K00384	trxB, TRR; thioredoxin reductase (NADPH) [EC:1.8.1.9]
AVI_02353	K05810	LACC1, yfiH; purine-nucleoside/S-methyl-5'-thioadenosine phosphorylase / adenosine deaminase [EC:2.4.2.1 2.4.2.28 3.5.4.4]
AVI_02355	K13292	lgt, umpA; phosphatidylglycerol---prolipoprotein diacylglyceryl transferase [EC:2.5.1.145]
AVI_02358	K00286	proC; pyrroline-5-carboxylate reductase [EC:1.5.1.2]
AVI_02359	K06878	K06878; tRNA-binding protein
AVI_02361	K11068	hlyIII; hemolysin III
AVI_02362	K00616	TALDO1, talB, talA; transaldolase [EC:2.2.1.2]
AVI_02363	K04066	priA; primosomal protein N' (replication factor Y) (superfamily II helicase) [EC:5.6.2.4]
AVI_02364	K01999	livK; branched-chain amino acid transport system substrate-binding protein
AVI_02367	K07304	msrA; peptide-methionine (S)-S-oxide reductase [EC:1.8.4.11]
AVI_02368	K00661	maa; maltose O-acetyltransferase [EC:2.3.1.79]
AVI_02369	K02687	prmA; ribosomal protein L11 methyltransferase [EC:2.1.1.-]
AVI_02372	K01159	ruvC; crossover junction endodeoxyribonuclease RuvC [EC:3.1.21.10]
AVI_02373	K03550	ruvA; holliday junction DNA helicase RuvA
AVI_02374	K03551	ruvB; holliday junction DNA helicase RuvB [EC:5.6.2.4]
AVI_02376	K07107	ybgC; acyl-CoA thioester hydrolase [EC:3.1.2.-]
AVI_02377	K03562	tolQ; biopolymer transport protein TolQ
AVI_02378	K03560	tolR; biopolymer transport protein TolR
AVI_02380	K03641	tolB; TolB protein

AVI_02381	K03640	pal; peptidoglycan-associated lipoprotein
AVI_02383	K04075	tilS, mesJ; tRNA(Ile)-lysidine synthase [EC:6.3.4.19]
AVI_02384	K03798	ftsH, hflB; cell division protease FtsH [EC:3.4.24.-]
AVI_02386	K01938	fhs; formate--tetrahydrofolate ligase [EC:6.3.4.3]
AVI_02387	K01491	folD; methylenetetrahydrofolate dehydrogenase (NADP+) / methenyltetrahydrofolate cyclohydrolase [EC:1.5.1.5 3.5.4.9]
AVI_02390	K06953	K06953; uncharacterized protein
AVI_02391	K03724	lhr; ATP-dependent helicase Lhr and Lhr-like helicase [EC:5.6.2.6 5.6.2.4]
AVI_02392	K00020	HIBADH, mmsB; 3-hydroxyisobutyrate dehydrogenase [EC:1.1.1.31]
AVI_02395	K02278	cpaA, tadV; prepilin peptidase CpaA [EC:3.4.23.43]
AVI_02398	K12511	tadC; tight adherence protein C
AVI_02399	K12510	tadB; tight adherence protein B
AVI_02400	K02283	cpaF, tadA; pilus assembly protein CpaF [EC:7.4.2.8]
AVI_02401	K02282	cpaE, tadZ; pilus assembly protein CpaE
AVI_02403	K02280	cpaC, rcpA; pilus assembly protein CpaC
AVI_02404	K02279	cpaB, rcpC; pilus assembly protein CpaB
AVI_02406	K02651	flp, pilA; pilus assembly protein Flp/PilA
AVI_02410	K16653	dprE1; decaprenylphospho-beta-D-ribofuranose 2-oxidase [EC:1.1.98.3]
AVI_02412	K06975	K06975; uncharacterized protein
AVI_02413	K01029	scoB; 3-oxoacid CoA-transferase subunit B [EC:2.8.3.5]
AVI_02415	K01028	scoA; 3-oxoacid CoA-transferase subunit A [EC:2.8.3.5]
AVI_02416	K03168	topA; DNA topoisomerase I [EC:5.6.2.1]
AVI_02418	K04096	smf; DNA processing protein
AVI_02420	K01286	E3.4.16.4; D-alanyl-D-alanine carboxypeptidase [EC:3.4.16.4]
AVI_02423	K03568	tldD; TldD protein
AVI_02425	K00274	MAO, aofH; monoamine oxidase [EC:1.4.3.4]
AVI_02426	K02275	coxB, ctaC; cytochrome c oxidase subunit II [EC:7.1.1.9]
AVI_02427	K02257	COX10, ctaB, cyoE; heme o synthase [EC:2.5.1.141]
AVI_02429	K02258	COX11, ctaG; cytochrome c oxidase assembly protein subunit 11
AVI_02430	K02276	coxC, ctaE; cytochrome c oxidase subunit III [EC:7.1.1.9]
AVI_02431	K14998	SURF1, SHY1; surfeit locus 1 family protein
AVI_02432	K01733	thrC; threonine synthase [EC:4.2.3.1]
AVI_02434	K03790	rimJ; [ribosomal protein S5]-alanine N-acetyltransferase [EC:2.3.1.267]
AVI_02439	K00759	APRT, apt; adenine phosphoribosyltransferase [EC:2.4.2.7]
AVI_02440	K05834	rhtB; homoserine/homoserine lactone efflux protein
AVI_02441	K00772	mtaP, MTAP; 5'-methylthioadenosine phosphorylase [EC:2.4.2.28]
AVI_02442	K03321	TC.SULP; sulfate permease, SulP family
AVI_02444	K18234	vat; virginiamycin A acetyltransferase [EC:2.3.1.-]
AVI_02451	K00114	exaA; alcohol dehydrogenase (cytochrome c) [EC:1.1.2.8]
AVI_02453	K02051	ABC.SN.S; NitT/TauT family transport system substrate-binding protein
AVI_02454	K02049	ABC.SN.A; NitT/TauT family transport system ATP-binding protein
AVI_02456	K01992	ABC-2.P; ABC-2 type transport system permease protein
AVI_02457	K01990	ABC-2.A; ABC-2 type transport system ATP-binding protein

AVI_02463	K09386	K09386; uncharacterized protein
AVI_02464	K03518	coxS; aerobic carbon-monoxide dehydrogenase small subunit [EC:1.2.5.3]
AVI_02465	K03520	coxL, cutL; aerobic carbon-monoxide dehydrogenase large subunit [EC:1.2.5.3]
AVI_02466	K03519	coxM, cutM; aerobic carbon-monoxide dehydrogenase medium subunit [EC:1.2.5.3]
AVI_02467	K03734	apbE; FAD:protein FMN transferase [EC:2.7.1.180]
AVI_02468	K19339	nosR; NosR/NirI family transcriptional regulator, nitrous oxide reductase regulator
AVI_02469	K02050	ABC.SN.P; NitT/TauT family transport system permease protein
AVI_02471	K22515	fdwB; formate dehydrogenase beta subunit [EC:1.17.1.9]
AVI_02472	K03110	ftsY; fused signal recognition particle receptor
AVI_02475	K06190	ispZ; intracellular septation protein
AVI_02477	K11209	yghU, yfcG; GSH-dependent disulfide-bond oxidoreductase [EC:1.8.4.-]
AVI_02479	K10764	metZ; O-succinylhomoserine sulfhydrylase [EC:2.5.1.-]
AVI_02483	K09007	folE2; GTP cyclohydrolase IB [EC:3.5.4.16]
AVI_02485	K00600	glyA, SHMT; glycine hydroxymethyltransferase [EC:2.1.2.1]
AVI_02486	K00858	ppnK, NADK; NAD+ kinase [EC:2.7.1.23]
AVI_02487	K07301	yrbG; cation:H+ antiporter
AVI_02488	K00330	nuoA; NADH-quinone oxidoreductase subunit A [EC:7.1.1.2]
AVI_02491	K00331	nuoB; NADH-quinone oxidoreductase subunit B [EC:7.1.1.2]
AVI_02492	K00332	nuoC; NADH-quinone oxidoreductase subunit C [EC:7.1.1.2]
AVI_02495	K00333	nuoD; NADH-quinone oxidoreductase subunit D [EC:7.1.1.2]
AVI_02499	K00334	nuoE; NADH-quinone oxidoreductase subunit E [EC:7.1.1.2]
AVI_02502	K00335	nuoF; NADH-quinone oxidoreductase subunit F [EC:7.1.1.2]
AVI_02507	K00336	nuoG; NADH-quinone oxidoreductase subunit G [EC:7.1.1.2]
AVI_02508	K00337	nuoH; NADH-quinone oxidoreductase subunit H [EC:7.1.1.2]
AVI_02510	K00338	nuoI; NADH-quinone oxidoreductase subunit I [EC:7.1.1.2]
AVI_02511	K01607	pcaC; 4-carboxymuconolactone decarboxylase [EC:4.1.1.44]
AVI_02512	K01607	pcaC; 4-carboxymuconolactone decarboxylase [EC:4.1.1.44]
AVI_02513	K00339	nuoJ; NADH-quinone oxidoreductase subunit J [EC:7.1.1.2]
AVI_02514	K00340	nuoK; NADH-quinone oxidoreductase subunit K [EC:7.1.1.2]
AVI_02515	K00341	nuoL; NADH-quinone oxidoreductase subunit L [EC:7.1.1.2]
AVI_02516	K00342	nuoM; NADH-quinone oxidoreductase subunit M [EC:7.1.1.2]
AVI_02517	K00343	nuoN; NADH-quinone oxidoreductase subunit N [EC:7.1.1.2]
AVI_02518	K03524	birA; BirA family transcriptional regulator, biotin operon repressor / biotin---[acetyl-CoA-carboxylase] ligase [EC:6.3.4.15]
AVI_02519	K03525	coaX; type III pantothenate kinase [EC:2.7.1.33]
AVI_02520	K12574	rnj; ribonuclease J [EC:3.1.-.-]
AVI_02526	K02837	prfC; peptide chain release factor 3
AVI_02530	K00059	fabG, OAR1; 3-oxoacyl-[acyl-carrier protein] reductase [EC:1.1.1.100]
AVI_02531	K02010	afuC, fbpC; iron(III) transport system ATP-binding protein [EC:7.2.2.7]
AVI_02537	K03116	tatA; sec-independent protein translocase protein TatA
AVI_02538	K03117	tatB; sec-independent protein translocase protein TatB
AVI_02539	K03118	tatC; sec-independent protein translocase protein TatC
AVI_02540	K06923	K06923; uncharacterized protein

AVI_02549	K07516	fadN; 3-hydroxyacyl-CoA dehydrogenase [EC:1.1.1.35]
AVI_02550	K00626	ACAT, atoB; acetyl-CoA C-acetyltransferase [EC:2.3.1.9]
AVI_02554	K23948	E1.6.5.9; NADH:quinone reductase (non-electrogenic) [EC:1.6.5.9]
AVI_02564	K07506	K07506; AraC family transcriptional regulator
AVI_02568	K07080	K07080; uncharacterized protein
AVI_02569	K02035	ABC.PE.S; peptide/nickel transport system substrate-binding protein
AVI_02573	K00875	rbtK, FGGY; D-ribulokinase [EC:2.7.1.47]
AVI_02574	K18910	dpe, lre; D-psicose/D-tagatose/L-ribulose 3-epimerase [EC:5.1.3.30 5.1.3.31]
AVI_02575	K02057	ABC.SS.P; simple sugar transport system permease protein
AVI_02576	K02057	ABC.SS.P; simple sugar transport system permease protein
AVI_02577	K02056	ABC.SS.A; simple sugar transport system ATP-binding protein [EC:7.5.2.-]
AVI_02578	K02058	ABC.SS.S; simple sugar transport system substrate-binding protein
AVI_02579	K03435	fruR1, fruR; LacI family transcriptional regulator, fructose operon transcriptional repressor
AVI_02581	K05844	rimK; ribosomal protein S6--L-glutamate ligase [EC:6.3.2.-]
AVI_02582	K06987	K06987; uncharacterized protein
AVI_02583	K01481	E3.5.3.15; protein-arginine deiminase [EC:3.5.3.15]
AVI_02587	K17686	copA, ctpA, ATP7; P-type Cu+ transporter [EC:7.2.2.8]
AVI_02588	K19591	cueR; MerR family transcriptional regulator, copper efflux regulator
AVI_02589	K07152	SCO1; protein SCO1
AVI_02590	K09796	pccA; periplasmic copper chaperone A
AVI_02592	K02003	ABC.CD.A; putative ABC transport system ATP-binding protein
AVI_02593	K02004	ABC.CD.P; putative ABC transport system permease protein
AVI_02595	K12410	cobB, srtN, npdA; NAD-dependent protein deacetylase/lipoamidase [EC:2.3.1.286 2.3.1.313]
AVI_02599	K02483	K02483; two-component system, OmpR family, response regulator
AVI_02602	K02902	RP-L28, MRPL28, rpmB; large subunit ribosomal protein L28
AVI_02603	K07588	MMAA, argK; GTPase [EC:3.6.5.-]
AVI_02604	K03579	hrpB; ATP-dependent RNA helicase HrpB [EC:5.6.2.6]
AVI_02606	K00033	PGD, gnd, gntZ; 6-phosphogluconate dehydrogenase [EC:1.1.1.44 1.1.1.343]
AVI_02609	K02053	ABC.SP.P; putative spermidine/putrescine transport system permease protein
AVI_02610	K02054	ABC.SP.P1; putative spermidine/putrescine transport system permease protein
AVI_02612	K02055	ABC.SP.S; putative spermidine/putrescine transport system substrate-binding protein
AVI_02613	K02052	ABC.SP.A; putative spermidine/putrescine transport system ATP-binding protein
AVI_02614	K13075	ahlD, aiiA, attM, blcC; N-acyl homoserine lactone hydrolase [EC:3.1.1.81]
AVI_02618	K01113	phoD; alkaline phosphatase D [EC:3.1.3.1]
AVI_02619	K08602	pepF, pepB; oligoendopeptidase F [EC:3.4.24.-]
AVI_02622	K07577	K07577; putative mRNA 3-end processing factor
AVI_02625	K03801	lipB; lipoyl(octanoyl) transferase [EC:2.3.1.181]
AVI_02634	K05571	mnhG, mrpG; multicomponent Na+:H+ antiporter subunit G
AVI_02635	K05570	mnhF, mrpF; multicomponent Na+:H+ antiporter subunit F
AVI_02636	K05569	mnhE, mrpE; multicomponent Na+:H+ antiporter subunit E
AVI_02637	K05568	mnhD, mrpD; multicomponent Na+:H+ antiporter subunit D
AVI_02638	K05567	mnhC, mrpC; multicomponent Na+:H+ antiporter subunit C

AVI_02639	K05566	mnhB, mrpB; multicomponent Na+:H+ antiporter subunit B
AVI_02640	K05565	mnhA, mrpA; multicomponent Na+:H+ antiporter subunit A
AVI_02642	K04761	oxyR; LysR family transcriptional regulator, hydrogen peroxide-inducible genes activator
AVI_02644	K02051	ABC.SN.S; NitT/TauT family transport system substrate-binding protein
AVI_02645	K02049	ABC.SN.A; NitT/TauT family transport system ATP-binding protein
AVI_02646	K02050	ABC.SN.P; NitT/TauT family transport system permease protein
AVI_02678	K06919	K06919; putative DNA primase/helicase
AVI_02695	K00558	DNMT1, dcm; DNA (cytosine-5)-methyltransferase 1 [EC:2.1.1.37]
AVI_02702	K07462	recJ; single-stranded-DNA-specific exonuclease [EC:3.1.-.-]
AVI_02703	K11532	glpX-SEBP; fructose-1,6-bisphosphatase II / sedoheptulose-1,7-bisphosphatase [EC:3.1.3.11 3.1.3.37]
AVI_02704	K00003	hom; homoserine dehydrogenase [EC:1.1.1.3]
AVI_02710	K00261	GLUD1_2, gdhA; glutamate dehydrogenase (NAD(P)+) [EC:1.4.1.3]
AVI_02712	K22084	mgdA; methylglutamate dehydrogenase subunit A [EC:1.5.99.5]
AVI_02716	K22085	mgdB; methylglutamate dehydrogenase subunit B [EC:1.5.99.5]
AVI_02717	K22086	mgdC; methylglutamate dehydrogenase subunit C [EC:1.5.99.5]
AVI_02718	K22087	mgdD; methylglutamate dehydrogenase subunit D [EC:1.5.99.5]
AVI_02719	K02316	dnaG; DNA primase [EC:2.7.7.101]
AVI_02720	K03086	rpoD; RNA polymerase primary sigma factor
AVI_02724	K01524	ppx-gppA; exopolyphosphatase / guanosine-5'-triphosphate,3'-diphosphate pyrophosphatase [EC:3.6.1.11 3.6.1.40]
AVI_02725	K00937	ppk1; polyphosphate kinase [EC:2.7.4.1]
AVI_02728	K01881	PARS, proS; prolyl-tRNA synthetase [EC:6.1.1.15]
AVI_02729	K09808	lolC_E; lipoprotein-releasing system permease protein
AVI_02730	K09810	lolD; lipoprotein-releasing system ATP-binding protein [EC:7.6.2.-]
AVI_02731	K09936	TC.BAT2; bacterial/archaeal transporter family-2 protein
AVI_02733	K00799	GST, gst; glutathione S-transferase [EC:2.5.1.18]
AVI_02734	K01640	HMGCL, hmgL; hydroxymethylglutaryl-CoA lyase [EC:4.1.3.4]
AVI_02735	K13766	liuC; methylglutaconyl-CoA hydratase [EC:4.2.1.18]
AVI_02737	K26937	dinF, mepA, vmrA; MATE family, multidrug efflux pump
AVI_02742	K03704	cspA; cold shock protein
AVI_02748	K20035	dmdC; 3-(methylsulfanyl)propanoyl-CoA dehydrogenase [EC:1.3.99.41]
AVI_02749	K00799	GST, gst; glutathione S-transferase [EC:2.5.1.18]
AVI_02750	K00626	ACAT, atoB; acetyl-CoA C-acetyltransferase [EC:2.3.1.9]
AVI_02752	K01782	fadJ; 3-hydroxyacyl-CoA dehydrogenase / enoyl-CoA hydratase / 3-hydroxybutyryl-CoA epimerase [EC:1.1.1.35 4.2.1.17 5.1.2.3]
AVI_02754	K20034	dmdB; 3-(methylthio)propionyl---CoA ligase [EC:6.2.1.44]
AVI_02758	K00761	upp, UPRT; uracil phosphoribosyltransferase [EC:2.4.2.9]
AVI_02759	K01488	add, ADA; adenosine deaminase [EC:3.5.4.4]
AVI_02760	K01839	deoB; phosphopentomutase [EC:5.4.2.7]
AVI_02761	K00758	deoA, TYMP; thymidine phosphorylase [EC:2.4.2.4]
AVI_02762	K01489	cdd, CDA; cytidine deaminase [EC:3.5.4.5]
AVI_02766	K00029	maeB; malate dehydrogenase (oxaloacetate-decarboxylating)(NADP+) [EC:1.1.1.40]
AVI_02768	K05834	rhtB; homoserine/homoserine lactone efflux protein
AVI_02769	K01908	ACSS3, prpE; propionyl-CoA synthetase [EC:6.2.1.17]

AVI_02770	K00254	DHODH, pyrD; dihydroorotate dehydrogenase [EC:1.3.5.2]
AVI_02772	K11751	ushA; 5'-nucleotidase / UDP-sugar diphosphatase [EC:3.1.3.5 3.6.1.45]
AVI_02774	K06147	ABCB-BAC; ATP-binding cassette, subfamily B, bacterial
AVI_02775	K03782	katG; catalase-peroxidase [EC:1.11.1.21]
AVI_02776	K04761	oxyR; LysR family transcriptional regulator, hydrogen peroxide-inducible genes activator
AVI_02777	K00666	K00666; fatty-acyl-CoA synthase [EC:6.2.1.-]
AVI_02779	K01696	trpB; tryptophan synthase beta chain [EC:4.2.1.20]
AVI_02783	K09966	K09966; uncharacterized protein
AVI_02786	K01056	PTH1, PTRH1, pth, spoVC; peptidyl-tRNA hydrolase, PTH1 family [EC:3.1.1.29]
AVI_02790	K02897	RP-L25, rplY; large subunit ribosomal protein L25
AVI_02792	K00101	lldD; L-lactate dehydrogenase (cytochrome) [EC:1.1.2.3]
AVI_02796	K01695	trpA; tryptophan synthase alpha chain [EC:4.2.1.20]
AVI_02797	K06942	ychF; ribosome-binding ATPase
AVI_02808	K01647	CS, gltA; citrate synthase [EC:2.3.3.1]
AVI_02810	K07793	tctA; putative tricarboxylic transport membrane protein
AVI_02811	K07794	tctB; putative tricarboxylic transport membrane protein
AVI_02812	K07795	tctC; putative tricarboxylic transport membrane protein
AVI_02814	K05952	K05952; uncharacterized protein
AVI_02815	K03734	apbE; FAD:protein FMN transferase [EC:2.7.1.180]
AVI_02816	K00351	nqrF; Na+-transporting NADH:ubiquinone oxidoreductase subunit F [EC:7.2.1.1]
AVI_02817	K00350	nqrE; Na+-transporting NADH:ubiquinone oxidoreductase subunit E [EC:7.2.1.1]
AVI_02818	K00349	nqrD; Na+-transporting NADH:ubiquinone oxidoreductase subunit D [EC:7.2.1.1]
AVI_02819	K00348	nqrC; Na+-transporting NADH:ubiquinone oxidoreductase subunit C [EC:7.2.1.1]
AVI_02820	K00347	nqrB; Na+-transporting NADH:ubiquinone oxidoreductase subunit B [EC:7.2.1.1]
AVI_02821	K00346	nqrA; Na+-transporting NADH:ubiquinone oxidoreductase subunit A [EC:7.2.1.1]
AVI_02825	K08970	rcnA; nickel/cobalt transporter (NicO) family protein
AVI_02832	K02077	ABC.ZM.S; zinc/manganese transport system substrate-binding protein
AVI_02833	K02075	ABC.ZM.P; zinc/manganese transport system permease protein
AVI_02834	K02074	ABC.ZM.A; zinc/manganese transport system ATP-binding protein
AVI_02835	K09823	zur; Fur family transcriptional regulator, zinc uptake regulator
AVI_02837	K11529	gck, gckA, GLYCTK; glycerate 2-kinase [EC:2.7.1.165]
AVI_02839	K02034	ABC.PE.P1; peptide/nickel transport system permease protein
AVI_02840	K02033	ABC.PE.P; peptide/nickel transport system permease protein
AVI_02841	K02035	ABC.PE.S; peptide/nickel transport system substrate-binding protein
AVI_02842	K06999	K06999; phospholipase/carboxylesterase
AVI_02843	K15975	K15975; glyoxalase family protein
AVI_02845	K10238	thuG, sugB; trehalose/maltose transport system permease protein
AVI_02848	K10112	msmX, msmK, malK, sugC, ggtA, msiK; multiple sugar transport system ATP-binding protein [EC:7.5.2.-]
AVI_02851	K21929	udg; uracil-DNA glycosylase [EC:3.2.2.27]
AVI_02852	K09800	tamB; translocation and assembly module TamB
AVI_02853	K07278	tamA; translocation and assembly module TamA
AVI_02854	K01004	pcs; phosphatidylcholine synthase [EC:2.7.8.24]

AVI_02859	K01870	IARS, ileS; isoleucyl-tRNA synthetase [EC:6.1.1.5]
AVI_02860	K01992	ABC-2.P; ABC-2 type transport system permease protein
AVI_02861	K13926	rbbA; ribosome-dependent ATPase
AVI_02862	K01993	ABC-2.TX; HlyD family secretion protein
AVI_02864	K01075	E3.1.2.23; 4-hydroxybenzoyl-CoA thioesterase [EC:3.1.2.23]
AVI_02865	K00252	GCDH, gcdH; glutaryl-CoA dehydrogenase [EC:1.3.8.6]
AVI_02867	K09461	E1.14.13.40; anthraniloyl-CoA monooxygenase [EC:1.14.13.40]
AVI_02871	K00249	ACADM, acd; acyl-CoA dehydrogenase [EC:1.3.8.7]
AVI_02872	K00453	TDO2, kynA; tryptophan 2,3-dioxygenase [EC:1.13.11.11]
AVI_02873	K08295	abmG; 2-aminobenzoate-CoA ligase [EC:6.2.1.32]
AVI_02874	K01999	livK; branched-chain amino acid transport system substrate-binding protein
AVI_02875	K01997	livH; branched-chain amino acid transport system permease protein
AVI_02876	K01998	livM; branched-chain amino acid transport system permease protein
AVI_02877	K01995	livG; branched-chain amino acid transport system ATP-binding protein
AVI_02878	K01996	livF; branched-chain amino acid transport system ATP-binding protein
AVI_02883	K07641	creC; two-component system, OmpR family, sensor histidine kinase CreC [EC:2.7.13.3]
AVI_02884	K02483	K02483; two-component system, OmpR family, response regulator
AVI_02889	K01783	rpe, RPE; ribulose-phosphate 3-epimerase [EC:5.1.3.1]
AVI_02890	K03490	chbR, celD; AraC family transcriptional regulator, dual regulator of chb operon
AVI_02891	K01619	deoC, DERA; deoxyribose-phosphate aldolase [EC:4.1.2.4]
AVI_02892	K00128	ALDH; aldehyde dehydrogenase (NAD+) [EC:1.2.1.3]
AVI_02908	K00135	gabD; succinate-semialdehyde dehydrogenase / glutarate-semialdehyde dehydrogenase [EC:1.2.1.16 1.2.1.79 1.2.1.20]
AVI_02912	K02453	gspD; general secretion pathway protein D
AVI_02914	K02460	gspK; general secretion pathway protein K
AVI_02916	K02458	gspI; general secretion pathway protein I
AVI_02918	K02456	gspG; general secretion pathway protein G
AVI_02919	K02455	gspF; general secretion pathway protein F
AVI_02920	K02454	gspE; general secretion pathway protein E [EC:7.4.2.8]
AVI_02923	K02461	gspL; general secretion pathway protein L
AVI_02926	K16841	hpxA; allantoin racemase [EC:5.1.99.3]
AVI_02927	K02053	ABC.SP.P; putative spermidine/putrescine transport system permease protein
AVI_02928	K02054	ABC.SP.P1; putative spermidine/putrescine transport system permease protein
AVI_02929	K02055	ABC.SP.S; putative spermidine/putrescine transport system substrate-binding protein
AVI_02930	K02052	ABC.SP.A; putative spermidine/putrescine transport system ATP-binding protein
AVI_02933	K01119	cpdB; 2',3'-cyclic-nucleotide 2'-phosphodiesterase / 3'-nucleotidase [EC:3.1.4.16 3.1.3.6]
AVI_02934	K00567	ogt, MGMT; methylated-DNA-[protein]-cysteine S-methyltransferase [EC:2.1.1.63]
AVI_02937	K00763	pncB, NAPRT1; nicotinate phosphoribosyltransferase [EC:6.3.4.21]
AVI_02939	K01790	rfbC, rmlC; dTDP-4-dehydrorhamnose 3,5-epimerase [EC:5.1.3.13]
AVI_02940	K01710	rfbB, rmlB, rffG; dTDP-glucose 4,6-dehydratase [EC:4.2.1.46]
AVI_02941	K00067	rfbD, rmlD; dTDP-4-dehydrorhamnose reductase [EC:1.1.1.133]
AVI_02942	K00973	rfbA, rmlA, rffH; glucose-1-phosphate thymidylyltransferase [EC:2.7.7.24]
AVI_02944	K09689	kpsT; capsular polysaccharide transport system ATP-binding protein [EC:7.6.2.12]

AVI_02945	K10107	kpsE; capsular polysaccharide transport system permease protein
AVI_02946	K09688	kpsM; capsular polysaccharide transport system permease protein
AVI_02947	K08281	pncA; nicotinamidase/pyrazinamidase [EC:3.5.1.19 3.5.1.-]
AVI_02948	K07146	K07146; UPF0176 protein
AVI_02951	K03667	hslU; ATP-dependent HslUV protease ATP-binding subunit HslU
AVI_02955	K01419	hslV, clpQ; ATP-dependent HslUV protease, peptidase subunit HslV [EC:3.4.25.2]
AVI_02958	K25908	pssI; exopolysaccharide biosynthesis glycosyltransferase PssI
AVI_02961	K03671	TXN, trxA; thioredoxin
AVI_02962	K16898	addA; ATP-dependent helicase/nuclease subunit A [EC:5.6.2.4 3.1.-.-]
AVI_02964	K00992	murU; N-acetyl-alpha-D-muramate 1-phosphate uridylyltransferase [EC:2.7.7.99]
AVI_02965	K07102	amgK; N-acetylmuramate 1-kinase [EC:2.7.1.221]
AVI_02966	K06925	tsaE; tRNA threonylcarbamoyladenosine biosynthesis protein TsaE
AVI_02968	K15011	regB, regS, actS; two-component system, sensor histidine kinase RegB [EC:2.7.13.3]
AVI_02969	K15012	regA, regR, actR; two-component system, response regulator RegA
AVI_02972	K06952	yfdR; 5'-nucleotidase [EC:3.1.3.89]
AVI_02974	K06049	bchO; magnesium chelatase accessory protein
AVI_02975	K03404	chlD, bchD; magnesium chelatase subunit D [EC:6.6.1.1]
AVI_02977	K03405	chlI, bchI; magnesium chelatase subunit I [EC:6.6.1.1]
AVI_02978	K09847	crtA; spheroidene monooxygenase [EC:1.14.15.9]
AVI_02986	K10439	rbsB; ribose transport system substrate-binding protein
AVI_02987	K10440	rbsC; ribose transport system permease protein
AVI_02988	K10441	rbsA; ribose transport system ATP-binding protein [EC:7.5.2.7]
AVI_02989	K05499	cytR; LacI family transcriptional regulator, repressor for deo operon, udp, cdd, tsx, nupC, and nupG
AVI_02992	K01823	idi, IDI; isopentenyl-diphosphate Delta-isomerase [EC:5.3.3.2]
AVI_02993	K10960	chlP, bchP; geranylgeranyl diphosphate/geranylgeranyl-bacteriochlorophyllide a reductase [EC:1.3.1.83 1.3.1.111]
AVI_02994	K08226	pucC; MFS transporter, BCD family, chlorophyll transporter
AVI_02995	K04040	chlG, bchG; chlorophyll/bacteriochlorophyll a synthase [EC:2.5.1.62 2.5.1.133]
AVI_02998	K02621	parC; topoisomerase IV subunit A [EC:5.6.2.2]
AVI_03003	K09710	ybeB; ribosome-associated protein
AVI_03004	K00783	rlmH; 23S rRNA (pseudouridine1915-N3)-methyltransferase [EC:2.1.1.177]
AVI_03005	K15633	gpmI; 2,3-bisphosphoglycerate-independent phosphoglycerate mutase [EC:5.4.2.12]
AVI_03006	K22719	envC; murein hydrolase activator
AVI_03007	K03797	E3.4.21.102, prc, ctpA; carboxyl-terminal processing protease [EC:3.4.21.102]
AVI_03008	K08311	nudH; putative (di)nucleoside polyphosphate hydrolase [EC:3.6.1.-]
AVI_03011	K08305	mltB; peptidoglycan lytic transglycosylase B [EC:4.2.2.29]
AVI_03012	K00451	HGD, hmgA; homogentisate 1,2-dioxygenase [EC:1.13.11.5]
AVI_03014	K16171	faaH; fumarylacetoacetate (FAA) hydrolase [EC:3.7.1.2]
AVI_03017	K05712	mhpA; 3-(3-hydroxy-phenyl)propionate hydroxylase [EC:1.14.13.127]
AVI_03019	K11748	kefG; glutathione-regulated potassium-efflux system ancillary protein KefG
AVI_03020	K11747	kefB; glutathione-regulated potassium-efflux system protein KefB
AVI_03021	K11604	sitA; manganese/iron transport system substrate-binding protein
AVI_03022	K11607	sitB; manganese/iron transport system ATP-binding protein

AVI_03023	K11605	sitC; manganese/iron transport system permease protein
AVI_03024	K11606	sitD; manganese/iron transport system permease protein
AVI_03027	K02335	polA; DNA polymerase I [EC:2.7.7.7]
AVI_03028	K01523	hisE; phosphoribosyl-ATP pyrophosphohydrolase [EC:3.6.1.31]
AVI_03029	K07074	K07074; uncharacterized protein
AVI_03030	K02500	hisF; imidazole glycerol-phosphate synthase subunit HisF [EC:4.3.2.10]
AVI_03036	K06996	K06996; uncharacterized protein
AVI_03040	K09986	K09986; uncharacterized protein
AVI_03041	K03924	moxR; MoxR-like ATPase [EC:3.6.3.-]
AVI_03047	K01755	argH, ASL; argininosuccinate lyase [EC:4.3.2.1]
AVI_03051	K01687	ilvD; dihydroxy-acid dehydratase [EC:4.2.1.9]
AVI_03057	K01963	accD; acetyl-CoA carboxylase carboxyl transferase subunit beta [EC:6.4.1.2 2.1.3.15]
AVI_03058	K11754	folC; dihydrofolate synthase / folylpolyglutamate synthase [EC:6.3.2.12 6.3.2.17]
AVI_03061	K06916	zapE; cell division protein ZapE
AVI_03065	K07025	K07025; putative hydrolase of the HAD superfamily
AVI_03066	K13936	mdcF; malonate transporter and related proteins
AVI_03072	K00121	frmA, ADH5, adhC; S-(hydroxymethyl)glutathione dehydrogenase / alcohol dehydrogenase [EC:1.1.1.284 1.1.1.1]
AVI_03075	K05782	benE; benzoate membrane transport protein
AVI_03080	K01255	CARP, pepA; leucyl aminopeptidase [EC:3.4.11.1]
AVI_03081	K05835	rhtC; threonine efflux protein
AVI_03083	K01673	cynT, can; carbonic anhydrase [EC:4.2.1.1]
AVI_03084	K07086	K07086; uncharacterized protein
AVI_03088	K00133	asd; aspartate-semialdehyde dehydrogenase [EC:1.2.1.11]
AVI_03090	K01533	copB; P-type Cu2+ transporter [EC:7.2.2.9]
AVI_03093	K00406	ccoP; cytochrome c oxidase cbb3-type subunit III
AVI_03094	K00407	ccoQ; cytochrome c oxidase cbb3-type subunit IV
AVI_03095	K00405	ccoO; cytochrome c oxidase cbb3-type subunit II
AVI_03096	K00404	ccoN; cytochrome c oxidase cbb3-type subunit I [EC:7.1.1.9]
AVI_03097	K01420	fnr; CRP/FNR family transcriptional regulator, anaerobic regulatory protein
AVI_03098	K02495	hemN, hemZ; oxygen-independent coproporphyrinogen III oxidase [EC:1.3.98.3]
AVI_03101	K00045	E1.1.1.67, mtlK; mannitol 2-dehydrogenase [EC:1.1.1.67]
AVI_03102	K21620	sorbD; galactitol 2-dehydrogenase [EC:1.1.1.16]
AVI_03103	K10111	malK, mtlK, thuK; multiple sugar transport system ATP-binding protein [EC:7.5.2.-]
AVI_03104	K10229	smoG, mtlG; polyol transport system permease protein
AVI_03105	K10228	smoF, mtlF; polyol transport system permease protein
AVI_03106	K10227	smoE, mtlE; polyol transport system substrate-binding protein
AVI_03110	K00219	fadH; 2,4-dienoyl-CoA reductase (NADPH2) [EC:1.3.1.34]
AVI_03112	K15977	K15977; putative oxidoreductase
AVI_03114	K00001	E1.1.1.1, adh; alcohol dehydrogenase [EC:1.1.1.1]
AVI_03115	K08151	tetA; MFS transporter, DHA1 family, tetracycline resistance protein
AVI_03116	K01857	pcaB; 3-carboxy-cis,cis-muconate cycloisomerase [EC:5.5.1.2]
AVI_03117	K01055	pcaD; 3-oxoadipate enol-lactonase [EC:3.1.1.24]

AVI_03119	K01754	E4.3.1.19, ilvA, tdcB; threonine dehydratase [EC:4.3.1.19]
AVI_03121	K01560	E3.8.1.2; 2-haloacid dehalogenase [EC:3.8.1.2]
AVI_03123	K07303	iorB; isoquinoline 1-oxidoreductase subunit beta [EC:1.3.99.16]
AVI_03124	K07302	iorA; isoquinoline 1-oxidoreductase subunit alpha [EC:1.3.99.16]
AVI_03125	K01187	malZ; alpha-glucosidase [EC:3.2.1.20]
AVI_03133	K02358	tuf, TUFM; elongation factor Tu
AVI_03134	K02946	RP-S10, MRPS10, rpsJ; small subunit ribosomal protein S10
AVI_03135	K02906	RP-L3, MRPL3, rplC; large subunit ribosomal protein L3
AVI_03136	K02926	RP-L4, MRPL4, rplD; large subunit ribosomal protein L4
AVI_03137	K02892	RP-L23, MRPL23, rplW; large subunit ribosomal protein L23
AVI_03138	K02886	RP-L2, MRPL2, RML2, rplB; large subunit ribosomal protein L2
AVI_03139	K02965	RP-S19, RSM19, rpsS; small subunit ribosomal protein S19
AVI_03140	K02890	RP-L22, MRPL22, rplV; large subunit ribosomal protein L22
AVI_03141	K02982	RP-S3, rpsC; small subunit ribosomal protein S3
AVI_03142	K02878	RP-L16, MRPL16, rplP; large subunit ribosomal protein L16
AVI_03145	K03559	exbD; biopolymer transport protein ExbD
AVI_03146	K03561	exbB; biopolymer transport protein ExbB
AVI_03152	K11907	vasG, clpV; type VI secretion system protein VasG
AVI_03153	K11895	impH, vasB; type VI secretion system protein ImpH
AVI_03154	K11896	impG, vasA; type VI secretion system protein ImpG
AVI_03155	K11897	impF; type VI secretion system protein ImpF
AVI_03156	K11903	hcp; type VI secretion system secreted protein Hcp
AVI_03157	K11900	impC; type VI secretion system protein ImpC
AVI_03158	K11901	impB; type VI secretion system protein ImpB
AVI_03159	K11902	impA; type VI secretion system protein ImpA
AVI_03161	K01945	purD; phosphoribosylamine---glycine ligase [EC:6.3.4.13]
AVI_03162	K03601	xseA; exodeoxyribonuclease VII large subunit [EC:3.1.11.6]
AVI_03167	K06168	miaB; tRNA-2-methylthio-N6-dimethylallyladenosine synthase [EC:2.8.4.3]
AVI_03170	K06217	phoH, phoL; phosphate starvation-inducible protein PhoH and related proteins
AVI_03172	K07042	ybeY, yqfG; probable rRNA maturation factor
AVI_03173	K06189	corC, tlyC; hemolysin (HlyC) family protein
AVI_03174	K03820	lnt; apolipoprotein N-acyltransferase [EC:2.3.1.269]
AVI_03175	K00789	metK, MAT; S-adenosylmethionine synthetase [EC:2.5.1.6]
AVI_03176	K03439	trmB, METTL1, TRM8; tRNA (guanine-N7-)-methyltransferase [EC:2.1.1.33]
AVI_03177	K00800	aroA; 3-phosphoshikimate 1-carboxyvinyltransferase [EC:2.5.1.19]
AVI_03180	K00945	cmk; CMP/dCMP kinase [EC:2.7.4.25]
AVI_03181	K19271	catA; chloramphenicol O-acetyltransferase type A [EC:2.3.1.28]
AVI_03182	K02945	RP-S1, rpsA; small subunit ribosomal protein S1
AVI_03183	K05788	ihfB, himD; integration host factor subunit beta
AVI_03185	K01817	trpF; phosphoribosylanthranilate isomerase [EC:5.3.1.24]
AVI_03189	K17722	preT; dihydropyrimidine dehydrogenase (NAD+) subunit PreT [EC:1.3.1.1]
AVI_03191	K10563	mutM, fpg; formamidopyrimidine-DNA glycosylase [EC:3.2.2.23 4.2.99.18]

AVI_03193	K17723	preA; dihydropyrimidine dehydrogenase (NAD+) subunit PreA [EC:1.3.1.1]
AVI_03194	K06016	pydC; beta-ureidopropionase / N-carbamoyl-L-amino-acid hydrolase [EC:3.5.1.6 3.5.1.87]
AVI_03196	K01464	DPYS, dht, hydA; dihydropyrimidinase [EC:3.5.2.2]
AVI_03197	K02049	ABC.SN.A; NitT/TauT family transport system ATP-binding protein
AVI_03198	K02050	ABC.SN.P; NitT/TauT family transport system permease protein
AVI_03199	K02050	ABC.SN.P; NitT/TauT family transport system permease protein
AVI_03200	K02051	ABC.SN.S; NitT/TauT family transport system substrate-binding protein
AVI_03203	K00790	murA; UDP-N-acetylglucosamine 1-carboxyvinyltransferase [EC:2.5.1.7]
AVI_03206	K00013	hisD; histidinol dehydrogenase [EC:1.1.1.23]
AVI_03212	K07010	K07010; putative glutamine amidotransferase
AVI_03214	K02518	infA; translation initiation factor IF-1
AVI_03215	K06287	yhdE; nucleoside triphosphate pyrophosphatase [EC:3.6.1.-]
AVI_03218	K09862	K09862; uncharacterized protein
AVI_03223	K03380	E1.14.13.7; phenol 2-monooxygenase (NADPH) [EC:1.14.13.7]
AVI_03224	K01630	garL; 2-dehydro-3-deoxyglucarate aldolase [EC:4.1.2.20]
AVI_03226	K11381	bkdA; 2-oxoisovalerate dehydrogenase E1 component [EC:1.2.4.4]
AVI_03230	K18013	kce; 3-keto-5-aminohexanoate cleavage enzyme [EC:2.3.1.247]
AVI_03233	K10112	msmX, msmK, malK, sugC, ggtA, msiK; multiple sugar transport system ATP-binding protein [EC:7.5.2.-]
AVI_03234	K02025	ABC.MS.P; multiple sugar transport system permease protein
AVI_03235	K02026	ABC.MS.P1; multiple sugar transport system permease protein
AVI_03236	K02027	ABC.MS.S; multiple sugar transport system substrate-binding protein
AVI_03237	K03651	cpdA; 3',5'-cyclic-AMP phosphodiesterase [EC:3.1.4.53]
AVI_03239	K10027	crtI; phytoene desaturase [EC:1.3.99.26 1.3.99.28 1.3.99.29 1.3.99.31]
AVI_03240	K02291	crtB; 15-cis-phytoene synthase [EC:2.5.1.32]
AVI_03241	K05770	TSPO, BZRP; translocator protein
AVI_03243	K09844	crtC; carotenoid 1,2-hydratase [EC:4.2.1.131]
AVI_03244	K09845	crtD; 1-hydroxycarotenoid 3,4-desaturase [EC:1.3.99.27]
AVI_03245	K13789	GGPS; geranylgeranyl diphosphate synthase, type II [EC:2.5.1.1 2.5.1.10 2.5.1.29]
AVI_03246	K09846	crtF; demethylspheroidene O-methyltransferase [EC:2.1.1.210]
AVI_03247	K11337	bchC; bacteriochlorophyllide a dehydrogenase [EC:1.1.1.396]
AVI_03248	K11333	bchX; 3,8-divinyl chlorophyllide a/chlorophyllide a reductase subunit X [EC:1.3.7.14 1.3.7.15]
AVI_03249	K11334	bchY; 3,8-divinyl chlorophyllide a/chlorophyllide a reductase subunit Y [EC:1.3.7.14 1.3.7.15]
AVI_03250	K11335	bchZ; 3,8-divinyl chlorophyllide a/chlorophyllide a reductase subunit Z [EC:1.3.7.14 1.3.7.15]
AVI_03253	K08927	pufB; light-harvesting complex 1 beta chain
AVI_03254	K08926	pufA; light-harvesting complex 1 alpha chain
AVI_03255	K08928	pufL; photosynthetic reaction center L subunit
AVI_03256	K08929	pufM; photosynthetic reaction center M subunit
AVI_03259	K02078	acpP; acyl carrier protein
AVI_03262	K15509	hpsN; sulfopropanediol 3-dehydrogenase [EC:1.1.1.308]
AVI_03263	K02001	proW; glycine betaine/proline transport system permease protein
AVI_03264	K02000	proV; glycine betaine/proline transport system ATP-binding protein [EC:7.6.2.9]
AVI_03265	K02002	proX; glycine betaine/proline transport system substrate-binding protein

AVI_03266	K01779	racD; aspartate racemase [EC:5.1.1.13]
AVI_03267	K17950	cuyA; L-cysteate sulfo-lyase [EC:4.4.1.25]
AVI_03268	K21645	hypT, qseD; LysR family transcriptional regulator, hypochlorite-specific transcription factor HypT
AVI_03269	K01811	xylS, yicI; alpha-D-xyloside xylohydrolase [EC:3.2.1.177]
AVI_03270	K10439	rbsB; ribose transport system substrate-binding protein
AVI_03271	K10441	rbsA; ribose transport system ATP-binding protein [EC:7.5.2.7]
AVI_03272	K10440	rbsC; ribose transport system permease protein
AVI_03273	K17206	xltB; putative xylitol transport system permease protein
AVI_03274	K01053	gnl, RGN; gluconolactonase [EC:3.1.1.17]
AVI_03275	K10112	msmX, msmK, malK, sugC, ggtA, msiK; multiple sugar transport system ATP-binding protein [EC:7.5.2.-]
AVI_03276	K02026	ABC.MS.P1; multiple sugar transport system permease protein
AVI_03277	K02025	ABC.MS.P; multiple sugar transport system permease protein
AVI_03278	K02027	ABC.MS.S; multiple sugar transport system substrate-binding protein
AVI_03279	K01811	xylS, yicI; alpha-D-xyloside xylohydrolase [EC:3.2.1.177]
AVI_03282	K02529	galR; LacI family transcriptional regulator, galactose operon repressor
AVI_03283	K16079	omp31; outer membrane immunogenic protein
AVI_03287	K03088	rpoE; RNA polymerase sigma-70 factor, ECF subfamily
AVI_03290	K01470	E3.5.2.10; creatinine amidohydrolase [EC:3.5.2.10]
AVI_03295	K01485	codA; cytosine/creatinine deaminase [EC:3.5.4.1 3.5.4.21]
AVI_03296	K02050	ABC.SN.P; NitT/TauT family transport system permease protein
AVI_03297	K02049	ABC.SN.A; NitT/TauT family transport system ATP-binding protein
AVI_03298	K02051	ABC.SN.S; NitT/TauT family transport system substrate-binding protein
AVI_03300	K06929	K06929; uncharacterized protein
AVI_03303	K03218	rlmB; 23S rRNA (guanosine2251-2'-O)-methyltransferase [EC:2.1.1.185]
AVI_03305	K06891	clpS; ATP-dependent Clp protease adaptor protein ClpS
AVI_03306	K01814	hisA; phosphoribosylformimino-5-aminoimidazole carboxamide ribotide isomerase [EC:5.3.1.16]
AVI_03308	K02501	hisH; imidazole glycerol-phosphate synthase subunit HisH [EC:4.3.2.10]
AVI_03309	K01693	hisB; imidazoleglycerol-phosphate dehydratase [EC:4.2.1.19]
AVI_03311	K11688	dctP; C4-dicarboxylate-binding protein DctP
AVI_03312	K02040	pstS; phosphate transport system substrate-binding protein
AVI_03315	K01958	PC, pyc; pyruvate carboxylase [EC:6.4.1.1]
AVI_03317	K00101	lldD; L-lactate dehydrogenase (cytochrome) [EC:1.1.2.3]
AVI_03318	K03657	uvrD, pcrA; ATP-dependent DNA helicase UvrD/PcrA [EC:5.6.2.4]
AVI_03324	K03593	mrp, NUBPL; ATP-binding protein involved in chromosome partitioning
AVI_03325	K03925	mraZ; transcriptional regulator MraZ
AVI_03326	K03438	mraW, rsmH; 16S rRNA (cytosine1402-N4)-methyltransferase [EC:2.1.1.199]
AVI_03327	K03586	ftsL; cell division protein FtsL
AVI_03328	K03587	ftsI; cell division protein FtsI (penicillin-binding protein 3) [EC:3.4.16.4]
AVI_03329	K01928	murE; UDP-N-acetylmuramoyl-L-alanyl-D-glutamate--2,6-diaminopimelate ligase [EC:6.3.2.13]
AVI_03330	K01929	murF; UDP-N-acetylmuramoyl-tripeptide--D-alanyl-D-alanine ligase [EC:6.3.2.10]
AVI_03331	K01000	mraY; phospho-N-acetylmuramoyl-pentapeptide-transferase [EC:2.7.8.13]
AVI_03332	K09964	K09964; uncharacterized protein

AVI_03333	K01925	murD; UDP-N-acetylmuramoylalanine--D-glutamate ligase [EC:6.3.2.9]
AVI_03334	K13821	putA; RHH-type transcriptional regulator, proline utilization regulon repressor / proline dehydrogenase / delta 1-pyrroline-5-carboxylate dehydrogenase [EC:1.5.5.2 1.2.1.88]
AVI_03337	K20074	prpC, phpP; PPM family protein phosphatase [EC:3.1.3.16]
AVI_03343	K02040	pstS; phosphate transport system substrate-binding protein
AVI_03346	K18661	matB; malonyl-CoA/methylmalonyl-CoA synthetase [EC:6.2.1.76 6.2.1.-]
AVI_03347	K00830	AGXT; alanine-glyoxylate transaminase / serine-glyoxylate transaminase / serine-pyruvate transaminase [EC:2.6.1.44 2.6.1.45 2.6.1.51]
AVI_03349	K01873	VARS, valS; valyl-tRNA synthetase [EC:6.1.1.9]
AVI_03352	K03520	coxL, cutL; aerobic carbon-monoxide dehydrogenase large subunit [EC:1.2.5.3]
AVI_03355	K00297	metF, MTHFR; methylenetetrahydrofolate reductase (NADH) [EC:1.5.1.54]
AVI_03356	K03576	metR; LysR family transcriptional regulator, regulator for metE and metH
AVI_03357	K21990	yfdC; formate-nitrite transporter family protein
AVI_03359	K03168	topA; DNA topoisomerase I [EC:5.6.2.1]
AVI_03360	K26937	dinF, mepA, vmrA; MATE family, multidrug efflux pump
AVI_03365	K03088	rpoE; RNA polymerase sigma-70 factor, ECF subfamily
AVI_03370	K07560	dtd, DTD; D-aminoacyl-tRNA deacylase [EC:3.1.1.96]
AVI_03371	K00847	E2.7.1.4, scrK; fructokinase [EC:2.7.1.4]
AVI_03375	K01969	MCCC2, accD1; 3-methylcrotonyl-CoA carboxylase beta subunit [EC:6.4.1.4]
AVI_03383	K00253	IVD, ivd; isovaleryl-CoA dehydrogenase [EC:1.3.8.4]
AVI_03386	K01091	gph; phosphoglycolate phosphatase [EC:3.1.3.18]
AVI_03387	K04042	glmU; bifunctional UDP-N-acetylglucosamine pyrophosphorylase / glucosamine-1-phosphate N-acetyltransferase [EC:2.7.7.23 2.3.1.157]
AVI_03388	K00820	glmS, GFPT; glutamine---fructose-6-phosphate transaminase (isomerizing) [EC:2.6.1.16]
AVI_03391	K03639	moaA, CNX2; GTP 3',8-cyclase [EC:4.1.99.22]
AVI_03394	K07182	K07182; CBS domain-containing protein
AVI_03395	K14393	actP; cation/acetate symporter
AVI_03397	K01875	SARS, serS; seryl-tRNA synthetase [EC:6.1.1.11]
AVI_03400	K03977	engA, der; GTPase
AVI_03403	K18990	vexE; membrane fusion protein, multidrug efflux system
AVI_03404	K18989	vexF; multidrug efflux pump
AVI_03405	K07552	bcr, tcaB; MFS transporter, DHA1 family, multidrug resistance protein
AVI_03406	K11752	ribD; diaminohydroxyphosphoribosylaminopyrimidine deaminase / 5-amino-6-(5-phosphoribosylamino)uracil reductase [EC:3.5.4.26 1.1.1.193]
AVI_03407	K07738	nrdR; transcriptional repressor NrdR
AVI_03409	K09165	K09165; dodecin
AVI_03413	K05801	djlA; DnaJ like chaperone protein
AVI_03414	K01847	MUT; methylmalonyl-CoA mutase [EC:5.4.99.2]
AVI_03417	K01965	PCCA, pccA; propionyl-CoA carboxylase alpha chain [EC:6.4.1.3]
AVI_03423	K01966	PCCB, pccB; propionyl-CoA carboxylase beta chain [EC:6.4.1.3 2.1.3.15]
AVI_03424	K07552	bcr, tcaB; MFS transporter, DHA1 family, multidrug resistance protein
AVI_03430	K09769	ymdB; 2',3'-cyclic-nucleotide 2'-phosphodiesterase [EC:3.1.4.16]
AVI_03431	K01934	MTHFS; 5-formyltetrahydrofolate cyclo-ligase [EC:6.3.3.2]
AVI_03432	K06213	mgtE; magnesium transporter
AVI_03433	K01487	guaD, GDA; guanine deaminase [EC:3.5.4.3]

AVI_03438	K00344	qor, CRYZ; NADPH:quinone reductase [EC:1.6.5.5]
AVI_03441	K06890	K06890; uncharacterized protein
AVI_03444	K02913	RP-L33, MRPL33, rpmG; large subunit ribosomal protein L33
AVI_03446	K01447	xlyAB; N-acetylmuramoyl-L-alanine amidase [EC:3.5.1.28]
AVI_03448	K02433	gatA, QRSL1; aspartyl-tRNA(Asn)/glutamyl-tRNA(Gln) amidotransferase subunit A [EC:6.3.5.6 6.3.5.7]
AVI_03449	K02435	gatC, GATC; aspartyl-tRNA(Asn)/glutamyl-tRNA(Gln) amidotransferase subunit C [EC:6.3.5.6 6.3.5.7]
AVI_03452	K03523	bioY; biotin transport system substrate-specific component
AVI_03454	K01756	purB, ADSL; adenylosuccinate lyase [EC:4.3.2.2]
AVI_03459	K02410	fliG; flagellar motor switch protein FliG
AVI_03462	K20760	scnA; thiocyanate hydrolase subunit alpha [EC:3.5.5.8]
AVI_03463	K01721	nthA; nitrile hydratase subunit alpha [EC:4.2.1.84]
AVI_03465	K02517	lpxL, htrB; Kdo2-lipid IVA lauroyltransferase/acyltransferase [EC:2.3.1.241 2.3.1.-]
AVI_03466	K01469	OPLAH, OXP1, oplAH; 5-oxoprolinase (ATP-hydrolysing) [EC:3.5.2.9]
AVI_03472	K00059	fabG, OAR1; 3-oxoacyl-[acyl-carrier protein] reductase [EC:1.1.1.100]
AVI_03473	K01681	ACO, acnA; aconitate hydratase [EC:4.2.1.3]
AVI_03475	K02199	ccmG, dsbE; cytochrome c biogenesis protein CcmG, thiol:disulfide interchange protein DsbE
AVI_03476	K02196	ccmD; heme exporter protein D
AVI_03477	K02195	ccmC; heme exporter protein C
AVI_03478	K02194	ccmB; heme exporter protein B
AVI_03479	K02193	ccmA; heme exporter protein A [EC:7.6.2.5]
AVI_03481	K07090	K07090; uncharacterized protein
AVI_03482	K07090	K07090; uncharacterized protein
AVI_03483	K03074	secF; preprotein translocase subunit SecF
AVI_03484	K03072	secD; preprotein translocase subunit SecD
AVI_03485	K03210	yajC; preprotein translocase subunit YajC
AVI_03487	K05835	rhtC; threonine efflux protein
AVI_03492	K03229	yscU, sctU, hrcU, ssaU; type III secretion protein U
AVI_03493	K03228	yscT, sctT, hrcT, ssaT; type III secretion protein T
AVI_03494	K03227	yscS, sctS, hrcS, ssaS; type III secretion protein S
AVI_03495	K03226	yscR, sctR, hrcR, ssaR; type III secretion protein R
AVI_03498	K03224	yscN, sctN, hrcN, ssaN; ATP synthase in type III secretion protein N [EC:7.4.2.8]
AVI_03499	K03223	yscL, sctL; type III secretion protein L
AVI_03501	K03222	yscJ, sctJ, hrcJ, ssaJ; type III secretion protein J
AVI_03507	K03220	yscD, sctD, ssaD; type III secretion protein D
AVI_03510	K03230	yscV, sctV, hrcV, ssaV, invA; type III secretion protein V
AVI_03527	K02622	parE; topoisomerase IV subunit B [EC:5.6.2.2]
AVI_03532	K07497	K07497; putative transposase
AVI_03538	K18989	vexF; multidrug efflux pump
AVI_03539	K18990	vexE; membrane fusion protein, multidrug efflux system
AVI_03541	K02483	K02483; two-component system, OmpR family, response regulator
AVI_03546	K00567	ogt, MGMT; methylated-DNA-[protein]-cysteine S-methyltransferase [EC:2.1.1.63]
AVI_03549	K00010	iolG; myo-inositol 2-dehydrogenase / D-chiro-inositol 1-dehydrogenase [EC:1.1.1.18 1.1.1.369]

AVI_03550	K01624	FBA, fbaA; fructose-bisphosphate aldolase, class II [EC:4.1.2.13]
AVI_03551	K03337	iolB; 5-deoxy-glucuronate isomerase [EC:5.3.1.30]
AVI_03552	K03338	iolC; 5-dehydro-2-deoxygluconokinase [EC:2.7.1.92]
AVI_03553	K03336	iolD; 3D-(3,5/4)-trihydroxycyclohexane-1,2-dione acylhydrolase (decyclizing) [EC:3.7.1.22]
AVI_03555	K03335	iolE; inosose dehydratase [EC:4.2.1.44]
AVI_03558	K02058	ABC.SS.S; simple sugar transport system substrate-binding protein
AVI_03559	K02057	ABC.SS.P; simple sugar transport system permease protein
AVI_03560	K02056	ABC.SS.A; simple sugar transport system ATP-binding protein [EC:7.5.2.-]
AVI_03561	K03337	iolB; 5-deoxy-glucuronate isomerase [EC:5.3.1.30]
AVI_03562	K07110	ramB; XRE family transcriptional regulator, fatty acid utilization regulator
AVI_03563	K01999	livK; branched-chain amino acid transport system substrate-binding protein
AVI_03564	K01997	livH; branched-chain amino acid transport system permease protein
AVI_03566	K01995	livG; branched-chain amino acid transport system ATP-binding protein
AVI_03567	K01996	livF; branched-chain amino acid transport system ATP-binding protein
AVI_03568	K01895	ACSS1_2, acs; acetyl-CoA synthetase [EC:6.2.1.1]
AVI_03571	K02160	accB, bccP; acetyl-CoA carboxylase biotin carboxyl carrier protein
AVI_03572	K01961	accC; acetyl-CoA carboxylase, biotin carboxylase subunit [EC:6.4.1.2 6.3.4.14]
AVI_03573	K00684	aat; leucyl/phenylalanyl-tRNA---protein transferase [EC:2.3.2.6]
AVI_03577	K03544	clpX, CLPX; ATP-dependent Clp protease ATP-binding subunit ClpX
AVI_03578	K01358	clpP, CLPP; ATP-dependent Clp protease, protease subunit [EC:3.4.21.92]
AVI_03582	K03088	rpoE; RNA polymerase sigma-70 factor, ECF subfamily
AVI_03584	K07058	K07058; membrane protein
AVI_03588	K06162	phnM; alpha-D-ribose 1-methylphosphonate 5-triphosphate diphosphatase [EC:3.6.1.63]
AVI_03591	K10554	frcA; fructose transport system ATP-binding protein
AVI_03592	K10553	frcC; fructose transport system permease protein
AVI_03593	K10552	frcB; fructose transport system substrate-binding protein
AVI_03598	K01652	E2.2.1.6L, ilvB, ilvG, ilvI; acetolactate synthase I/II/III large subunit [EC:2.2.1.6]
AVI_03602	K03670	mdoG; periplasmic glucans biosynthesis protein
AVI_03603	K03669	mdoH; membrane glycosyltransferase [EC:2.4.1.-]
AVI_03608	K06991	K06991; uncharacterized protein
AVI_03609	K01740	metY; O-acetylhomoserine (thiol)-lyase [EC:2.5.1.49]
AVI_03612	K00457	HPD, hppD; 4-hydroxyphenylpyruvate dioxygenase [EC:1.13.11.27]
AVI_03614	K00768	E2.4.2.21, cobU, cobT; nicotinate-nucleotide--dimethylbenzimidazole phosphoribosyltransferase [EC:2.4.2.21]
AVI_03615	K02233	E2.7.8.26, cobS, cobV; adenosylcobinamide-GDP ribazoletransferase [EC:2.7.8.26]
AVI_03617	K04755	fdx; ferredoxin, 2Fe-2S
AVI_03619	K04771	degP, htrA; serine protease Do [EC:3.4.21.107]
AVI_03620	K09937	K09937; uncharacterized protein
AVI_03621	K04087	hflC; modulator of FtsH protease HflC
AVI_03622	K04088	hflK; modulator of FtsH protease HflK
AVI_03623	K00383	GSR, gor; glutathione reductase (NADPH) [EC:1.8.1.7]
AVI_03624	K01807	rpiA; ribose 5-phosphate isomerase A [EC:5.3.1.6]
AVI_03626	K00949	thiN, TPK1, THI80; thiamine pyrophosphokinase [EC:2.7.6.2]

AVI_03629	K01939	purA, ADSS; adenylosuccinate synthase [EC:6.3.4.4]
AVI_03630	K03075	secG; preprotein translocase subunit SecG
AVI_03631	K01937	pyrG, CTPS; CTP synthase [EC:6.3.4.2]
AVI_03632	K06998	phzF; trans-2,3-dihydro-3-hydroxyanthranilate isomerase [EC:5.3.3.17]
AVI_03633	K07274	mipA, ompV; MipA family protein
AVI_03636	K02028	ABC.PA.A; polar amino acid transport system ATP-binding protein [EC:7.4.2.1]
AVI_03637	K02030	ABC.PA.S; polar amino acid transport system substrate-binding protein
AVI_03639	K02029	ABC.PA.P; polar amino acid transport system permease protein
AVI_03640	K02029	ABC.PA.P; polar amino acid transport system permease protein
AVI_03642	K05835	rhtC; threonine efflux protein
AVI_03644	K01915	glnA, GLUL; glutamine synthetase [EC:6.3.1.2]
AVI_03650	K03821	phaC, phbC; poly[(R)-3-hydroxyalkanoate] polymerase subunit PhaC [EC:2.3.1.304]
AVI_03651	K05973	phaZ; poly(3-hydroxybutyrate) depolymerase [EC:3.1.1.75]
AVI_03654	K00549	metE; 5-methyltetrahydropteroyltriglutamate--homocysteine methyltransferase [EC:2.1.1.14]
AVI_03659	K02034	ABC.PE.P1; peptide/nickel transport system permease protein
AVI_03660	K02033	ABC.PE.P; peptide/nickel transport system permease protein
AVI_03661	K02035	ABC.PE.S; peptide/nickel transport system substrate-binding protein
AVI_03662	K18954	pobR; AraC family transcriptional regulator, transcriptional activator of pobA
AVI_03663	K00791	miaA, TRIT1; tRNA dimethylallyltransferase [EC:2.5.1.75]
AVI_03664	K09903	pyrH; uridylate kinase [EC:2.7.4.22]
AVI_03666	K02838	frr, MRRF, RRF; ribosome recycling factor
AVI_03668	K00806	uppS; undecaprenyl diphosphate synthase [EC:2.5.1.31]
AVI_03669	K00981	E2.7.7.41, CDS1, CDS2, cdsA; phosphatidate cytidylyltransferase [EC:2.7.7.41]
AVI_03670	K00099	dxr; 1-deoxy-D-xylulose-5-phosphate reductoisomerase [EC:1.1.1.267]
AVI_03671	K11749	rseP; regulator of sigma E protease [EC:3.4.24.-]
AVI_03673	K07277	SAM50, TOB55, bamA; outer membrane protein insertion porin family
AVI_03675	K02372	fabZ; 3-hydroxyacyl-[acyl-carrier-protein] dehydratase [EC:4.2.1.59]
AVI_03676	K00677	lpxA; UDP-N-acetylglucosamine acyltransferase [EC:2.3.1.129]
AVI_03677	K09949	lpxI; UDP-2,3-diacylglucosamine hydrolase [EC:3.6.1.54]
AVI_03678	K00748	lpxB; lipid-A-disaccharide synthase [EC:2.4.1.182]
AVI_03683	K00566	mnmA, trmU; tRNA-uridine 2-sulfurtransferase [EC:2.8.1.13]
AVI_03685	K13584	ctrA; two-component system, cell cycle response regulator CtrA
AVI_03686	K01972	E6.5.1.2, ligA, ligB; DNA ligase (NAD+) [EC:6.5.1.2]
AVI_03687	K03655	recG; ATP-dependent DNA helicase RecG [EC:5.6.2.4]
AVI_03690	K01496	hisI; phosphoribosyl-AMP cyclohydrolase [EC:3.5.4.19]
AVI_03691	K01894	gluQ; glutamyl-Q tRNA(Asp) synthetase [EC:6.1.1.-]
AVI_03693	K04094	trmFO, gid; methylenetetrahydrofolate--tRNA-(uracil-5-)-methyltransferase [EC:2.1.1.74]
AVI_03694	K02469	gyrA; DNA gyrase subunit A [EC:5.6.2.2]
AVI_03698	K01750	E4.3.1.12, ocd; ornithine cyclodeaminase [EC:4.3.1.12]
AVI_03699	K01476	E3.5.3.1, rocF, arg; arginase [EC:3.5.3.1]
AVI_03702	K07054	K07054; uncharacterized protein
AVI_03703	K21395	yiaO; TRAP-type transport system periplasmic protein

AVI_03704	K20940	phzS; 5-methylphenazine-1-carboxylate 1-monooxygenase [EC:1.14.13.218]
AVI_03707	K09705	K09705; uncharacterized protein
AVI_03709	K01689	ENO1_2_3, eno; enolase 1/2/3 [EC:4.2.1.11]
AVI_03711	K03711	fur, zur, furB; Fur family transcriptional regulator, ferric uptake regulator
AVI_03713	K08997	SELENOO, selO; protein adenylyltransferase [EC:2.7.7.108]
AVI_03717	K09957	K09957; uncharacterized protein
AVI_03724	K05520	yhbO; deglycase [EC:3.5.1.124]
AVI_03726	K02427	rlmE, rrmJ, ftsJ; 23S rRNA (uridine2552-2'-O)-methyltransferase [EC:2.1.1.166]
AVI_03727	K00864	glpK, GK; glycerol kinase [EC:2.7.1.30]
AVI_03729	K00102	LDHD, dld; D-lactate dehydrogenase (cytochrome) [EC:1.1.2.4]
AVI_03735	K00123	fdoG, fdhF, fdwA; formate dehydrogenase major subunit [EC:1.17.1.9]
AVI_03736	K00124	fdoH, fdsB; formate dehydrogenase iron-sulfur subunit
AVI_03737	K00127	fdoI, fdsG; formate dehydrogenase subunit gamma
AVI_03739	K05773	tupB, vupB; tungstate transport system permease protein
AVI_03740	K06857	tupC, vupC; tungstate transport system ATP-binding protein [EC:7.3.2.6]
AVI_03741	K05772	tupA, vupA; tungstate transport system substrate-binding protein
AVI_03742	K07219	K07219; putative molybdopterin biosynthesis protein
AVI_03748	K02416	fliM; flagellar motor switch protein FliM
AVI_03751	K00249	ACADM, acd; acyl-CoA dehydrogenase [EC:1.3.8.7]
AVI_03755	K00688	PYG, glgP; glycogen phosphorylase [EC:2.4.1.1]
AVI_03756	K00700	GBE1, glgB; 1,4-alpha-glucan branching enzyme [EC:2.4.1.18]
AVI_03757	K00975	glgC; glucose-1-phosphate adenylyltransferase [EC:2.7.7.27]
AVI_03758	K00703	glgA; starch synthase [EC:2.4.1.21]
AVI_03759	K01214	ISA, treX; isoamylase [EC:3.2.1.68]
AVI_03760	K01835	pgm; phosphoglucomutase [EC:5.4.2.2]
AVI_03764	K07395	K07395; putative proteasome-type protease
AVI_03769	K00874	kdgK; 2-dehydro-3-deoxygluconokinase [EC:2.7.1.45]
AVI_03774	K03497	parB, spo0J; ParB family transcriptional regulator, chromosome partitioning protein
AVI_03776	K19092	parE1_3_4; toxin ParE1/3/4
AVI_03777	K07746	parD1_3_4; antitoxin ParD1/3/4
AVI_03784	K02914	RP-L34, MRPL34, rpmH; large subunit ribosomal protein L34
AVI_03785	K03536	rnpA; ribonuclease P protein component [EC:3.1.26.5]
AVI_03786	K08998	K08998; uncharacterized protein
AVI_03788	K14058	ttcA; tRNA 2-thiocytidine biosynthesis protein TtcA
AVI_03789	K03217	yidC, spoIIIJ, OXA1, ccfA; YidC/Oxa1 family membrane protein insertase
AVI_03790	K03978	engB; GTP-binding protein
AVI_03791	K00930	argB; acetylglutamate kinase [EC:2.7.2.8]
AVI_03792	K08296	sixA; phosphohistidine phosphatase [EC:3.1.3.-]
AVI_03793	K09972	aapP, bztD; general L-amino acid transport system ATP-binding protein [EC:7.4.2.1]
AVI_03794	K09971	aapM, bztC; general L-amino acid transport system permease protein
AVI_03795	K09970	aapQ, bztB; general L-amino acid transport system permease protein
AVI_03796	K09969	aapJ, bztA; general L-amino acid transport system substrate-binding protein

AVI_03798	K06179	rluC; 23S rRNA pseudouridine955/2504/2580 synthase [EC:5.4.99.24]
AVI_03799	K06199	crcB, FEX; fluoride exporter
AVI_03800	K07478	ycaJ; putative ATPase
AVI_03802	K07782	sdiA; LuxR family transcriptional regulator, quorum-sensing system regulator SdiA
AVI_03804	K02879	RP-L17, MRPL17, rplQ; large subunit ribosomal protein L17
AVI_03805	K03040	rpoA; DNA-directed RNA polymerase subunit alpha [EC:2.7.7.6]
AVI_03806	K02948	RP-S11, MRPS11, rpsK; small subunit ribosomal protein S11
AVI_03807	K02952	RP-S13, rpsM; small subunit ribosomal protein S13
AVI_03808	K00939	adk, AK; adenylate kinase [EC:2.7.4.3]
AVI_03809	K03076	secY; preprotein translocase subunit SecY
AVI_03810	K02876	RP-L15, MRPL15, rplO; large subunit ribosomal protein L15
AVI_03812	K02907	RP-L30, MRPL30, rpmD; large subunit ribosomal protein L30
AVI_03813	K02988	RP-S5, MRPS5, rpsE; small subunit ribosomal protein S5
AVI_03814	K02881	RP-L18, MRPL18, rplR; large subunit ribosomal protein L18
AVI_03815	K02933	RP-L6, MRPL6, rplF; large subunit ribosomal protein L6
AVI_03816	K02994	RP-S8, MRPS8, rpsH; small subunit ribosomal protein S8
AVI_03817	K02954	RP-S14, MRPS14, rpsN; small subunit ribosomal protein S14
AVI_03818	K02931	RP-L5, MRPL5, rplE; large subunit ribosomal protein L5
AVI_03819	K02895	RP-L24, MRPL24, rplX; large subunit ribosomal protein L24
AVI_03820	K02874	RP-L14, MRPL14, rplN; large subunit ribosomal protein L14
AVI_03821	K02961	RP-S17, MRPS17, rpsQ; small subunit ribosomal protein S17
AVI_03822	K02904	RP-L29, rpmC; large subunit ribosomal protein L29
AVI_03823	K22468	ppk2; polyphosphate kinase [EC:2.7.4.34]
AVI_03827	K11890	impM; type VI secretion system protein ImpM
AVI_03828	K11891	impL, vasK, icmF; type VI secretion system protein ImpL
AVI_03829	K11892	impK, ompA, vasF, dotU; type VI secretion system protein ImpK
AVI_03830	K11893	impJ, vasE; type VI secretion system protein ImpJ
AVI_03833	K05779	ynjD; putative thiamine transport system ATP-binding protein
AVI_03834	K05778	ynjC; putative thiamine transport system permease protein
AVI_03835	K05777	ynjB; putative thiamine transport system substrate-binding protein
AVI_03838	K01087	otsB; trehalose 6-phosphate phosphatase [EC:3.1.3.12]
AVI_03839	K00697	otsA; trehalose 6-phosphate synthase [EC:2.4.1.15 2.4.1.347]
AVI_03843	K04080	ibpA; molecular chaperone IbpA
AVI_03846	K11473	glcF; glycolate dehydrogenase iron-sulfur subunit [EC:1.1.99.14]
AVI_03848	K11472	glcE; glycolate dehydrogenase FAD-binding subunit [EC:1.1.99.14]
AVI_03849	K00104	glcD; glycolate dehydrogenase FAD-linked subunit [EC:1.1.99.14]
AVI_03851	K00799	GST, gst; glutathione S-transferase [EC:2.5.1.18]
AVI_03852	K16137	nemR; TetR/AcrR family transcriptional regulator, transcriptional repressor for nem operon
AVI_03855	K00799	GST, gst; glutathione S-transferase [EC:2.5.1.18]
AVI_03863	K00344	qor, CRYZ; NADPH:quinone reductase [EC:1.6.5.5]
AVI_03888	K03746	hns; DNA-binding protein H-NS
AVI_03889	K10778	ada; AraC family transcriptional regulator, regulatory protein of adaptative response / methylated-DNA-[protein]-cysteine methyltransferase [EC:2.1.1.63]

AVI_03891	K01259	pip; proline iminopeptidase [EC:3.4.11.5]
AVI_03896	K06894	yfhM; alpha-2-macroglobulin
AVI_03897	K05367	pbpC; penicillin-binding protein 1C [EC:2.4.99.28]
AVI_03898	K03746	hns; DNA-binding protein H-NS
AVI_03900	K07133	K07133; uncharacterized protein
AVI_03904	K02483	K02483; two-component system, OmpR family, response regulator
AVI_03911	K03296	TC.HAE1; hydrophobic/amphiphilic exporter-1 (mainly G- bacteria), HAE1 family
AVI_03914	K01630	garL; 2-dehydro-3-deoxyglucarate aldolase [EC:4.1.2.20]
AVI_03917	K21395	yiaO; TRAP-type transport system periplasmic protein
AVI_03918	K11206	NIT1, ybeM; deaminated glutathione amidase [EC:3.5.1.128]
AVI_03921	K07669	mprA; two-component system, OmpR family, response regulator MprA
AVI_03926	K06147	ABCB-BAC; ATP-binding cassette, subfamily B, bacterial
AVI_03929	K06893	K06893; uncharacterized protein
AVI_03936	K07486	K07486; transposase
AVI_03937	K00573	E2.1.1.77, pcm; protein-L-isoaspartate(D-aspartate) O-methyltransferase [EC:2.1.1.77]
AVI_03938	K03787	surE; 5'/3'-nucleotidase [EC:3.1.3.5 3.1.3.6]
AVI_03943	K00764	purF, PPAT; amidophosphoribosyltransferase [EC:2.4.2.14]
AVI_03944	K03558	cvpA; membrane protein required for colicin V production
AVI_03945	K04485	radA, sms; DNA repair protein RadA/Sms
AVI_03948	K01775	alr; alanine racemase [EC:5.1.1.1]
AVI_03950	K07305	msrB; peptide-methionine (R)-S-oxide reductase [EC:1.8.4.12]
AVI_03952	K15738	uup; ABC transport system ATP-binding/permease protein
AVI_03953	K01476	E3.5.3.1, rocF, arg; arginase [EC:3.5.3.1]
AVI_03955	K07093	K07093; uncharacterized protein
AVI_03957	K07341	doc; death on curing protein
AVI_03958	K18923	stbD; antitoxin StbD
AVI_03959	K03596	lepA; GTP-binding protein LepA
AVI_03969	K01175	ybfF; esterase [EC:3.1.-.-]
AVI_03971	K03498	trkH, trkG, ktrB, ktrD; trk/ktr system potassium uptake protein
AVI_03973	K01878	glyQ; glycyl-tRNA synthetase alpha chain [EC:6.1.1.14]
AVI_03975	K01879	glyS; glycyl-tRNA synthetase beta chain [EC:6.1.1.14]
AVI_03976	K01006	ppdK; pyruvate, orthophosphate dikinase [EC:2.7.9.1]
AVI_03978	K01633	folB; 7,8-dihydroneopterin aldolase/epimerase/oxygenase [EC:4.1.2.25 5.1.99.8 1.13.11.81]
AVI_03979	K00796	folP; dihydropteroate synthase [EC:2.5.1.15]
AVI_03980	K03431	glmM; phosphoglucosamine mutase [EC:5.4.2.10]
AVI_03983	K00053	ilvC; ketol-acid reductoisomerase [EC:1.1.1.86]
AVI_03985	K03185	ubiH; 2-octaprenyl-6-methoxyphenol hydroxylase [EC:1.14.13.-]
AVI_03986	K07025	K07025; putative hydrolase of the HAD superfamily
AVI_03989	K01956	carA, CPA1; carbamoyl-phosphate synthase small subunit [EC:6.3.5.5]
AVI_03990	K09117	K09117; uncharacterized protein
AVI_03993	K02274	coxA, ctaD; cytochrome c oxidase subunit I [EC:7.1.1.9]
AVI_04004	K01048	pldB; lysophospholipase [EC:3.1.1.5]

AVI_04007	K17675	SUPV3L1, SUV3; ATP-dependent RNA helicase SUPV3L1/SUV3 [EC:5.6.2.6]
AVI_04008	K04762	hslR; ribosome-associated heat shock protein Hsp15
AVI_04009	K05524	fdxA; ferredoxin
AVI_04010	K07736	carD; CarD family transcriptional regulator, regulator of rRNA transcription
AVI_04012	K11177	yagR; xanthine dehydrogenase YagR molybdenum-binding subunit [EC:1.17.1.4]
AVI_04013	K11178	yagS; xanthine dehydrogenase YagS FAD-binding subunit [EC:1.17.1.4]
AVI_04014	K13483	yagT; xanthine dehydrogenase YagT iron-sulfur-binding subunit
AVI_04015	K06893	K06893; uncharacterized protein
AVI_04016	K07506	K07506; AraC family transcriptional regulator
AVI_04024	K02032	ddpF; peptide/nickel transport system ATP-binding protein
AVI_04025	K02031	ddpD; peptide/nickel transport system ATP-binding protein
AVI_04026	K12369	dppB; dipeptide transport system permease protein
AVI_04027	K02034	ABC.PE.P1; peptide/nickel transport system permease protein
AVI_04028	K02035	ABC.PE.S; peptide/nickel transport system substrate-binding protein
AVI_04030	K22601	hpxY; oxamate carbamoyltransferase [EC:2.1.3.5]
AVI_04032	K00681	ggt; gamma-glutamyltranspeptidase / glutathione hydrolase [EC:2.3.2.2 3.4.19.13]
AVI_04033	K18335	E1.1.1.434; 2-dehydro-3-deoxy-L-fuconate 4-dehydrogenase [EC:1.1.1.434]
AVI_04034	K21417	acoB; acetoin:2,6-dichlorophenolindophenol oxidoreductase subunit beta [EC:1.1.1.-]
AVI_04035	K21416	acoA; acetoin:2,6-dichlorophenolindophenol oxidoreductase subunit alpha [EC:1.1.1.-]
AVI_04041	K03593	mrp, NUBPL; ATP-binding protein involved in chromosome partitioning
AVI_04043	K03089	rpoH; RNA polymerase sigma-32 factor
AVI_04045	K01552	ecfA; energy-coupling factor transport system ATP-binding protein [EC:7.-.-.-]
AVI_04046	K16925	ykoE; energy-coupling factor transport system permease protein
AVI_04047	K24620	ykoF; energy-coupling factor transport system substrate-specific component
AVI_04048	K05835	rhtC; threonine efflux protein
AVI_04053	K00663	aacA; aminoglycoside 6'-N-acetyltransferase [EC:2.3.1.82]
AVI_04056	K09022	ridA, tdcF, RIDA; 2-iminobutanoate/2-iminopropanoate deaminase [EC:3.5.99.10]
AVI_04062	K07090	K07090; uncharacterized protein
AVI_04065	K05835	rhtC; threonine efflux protein
AVI_04066	K05834	rhtB; homoserine/homoserine lactone efflux protein
AVI_04069	K05834	rhtB; homoserine/homoserine lactone efflux protein
AVI_04073	K00799	GST, gst; glutathione S-transferase [EC:2.5.1.18]
AVI_04083	K06173	truA, PUS1; tRNA pseudouridine38-40 synthase [EC:5.4.99.12]
AVI_04084	K03733	xerC; integrase/recombinase XerC
AVI_04085	K09921	K09921; uncharacterized protein
AVI_04086	K00432	gpx, btuE, bsaA; glutathione peroxidase [EC:1.11.1.9]
AVI_04087	K01992	ABC-2.P; ABC-2 type transport system permease protein
AVI_04088	K13583	gcrA; GcrA cell cycle regulator
AVI_04089	K07238	TC.ZIP, zupT, ZRT3, ZIP2; zinc transporter, ZIP family
AVI_04091	K14441	rimO; ribosomal protein S12 methylthiotransferase [EC:2.8.4.4]
AVI_04094	K00821	argD; acetylornithine/N-succinyldiaminopimelate aminotransferase [EC:2.6.1.11 2.6.1.17]
AVI_04098	K00611	OTC, argF, argI; ornithine carbamoyltransferase [EC:2.1.3.3]

AVI_04099	K13936	mdcF; malonate transporter and related proteins
AVI_04100	K06167	phnP; phosphoribosyl 1,2-cyclic phosphate phosphodiesterase [EC:3.1.4.55]
AVI_04101	K03424	tatD; TatD DNase family protein [EC:3.1.21.-]
AVI_04102	K02341	holB; DNA polymerase III subunit delta' [EC:2.7.7.7]
AVI_04104	K00943	tmk, DTYMK; dTMP kinase [EC:2.7.4.9]
AVI_04106	K07258	dacC, dacA, dacD; serine-type D-Ala-D-Ala carboxypeptidase (penicillin-binding protein 5/6) [EC:3.4.16.4]
AVI_04114	K02055	ABC.SP.S; putative spermidine/putrescine transport system substrate-binding protein
AVI_04115	K02052	ABC.SP.A; putative spermidine/putrescine transport system ATP-binding protein
AVI_04116	K02054	ABC.SP.P1; putative spermidine/putrescine transport system permease protein
AVI_04117	K02053	ABC.SP.P; putative spermidine/putrescine transport system permease protein
AVI_04118	K00137	prr; aminobutyraldehyde dehydrogenase [EC:1.2.1.19]
AVI_04125	K01465	URA4, pyrC; dihydroorotase [EC:3.5.2.3]
AVI_04129	K15509	hpsN; sulfopropanediol 3-dehydrogenase [EC:1.1.1.308]
AVI_04130	K03292	TC.GPH; glycoside/pentoside/hexuronide:cation symporter, GPH family
AVI_04136	K01868	TARS, thrS; threonyl-tRNA synthetase [EC:6.1.1.3]
AVI_04138	K09930	K09930; uncharacterized protein
AVI_04140	K15977	K15977; putative oxidoreductase
AVI_04141	K03704	cspA; cold shock protein
AVI_04142	K03465	thyX, thy1; thymidylate synthase (FAD) [EC:2.1.1.148]
AVI_04143	K01759	GLO1, gloA; lactoylglutathione lyase [EC:4.4.1.5]
AVI_04146	K09159	cptB; antitoxin CptB
AVI_04148	K00812	aspB; aspartate aminotransferase [EC:2.6.1.1]
AVI_04150	K19335	bdcR; TetR/AcrR family transcriptional regulator, repressor for divergent bdcA
AVI_04152	K11209	yghU, yfcG; GSH-dependent disulfide-bond oxidoreductase [EC:1.8.4.-]
AVI_04157	K05841	E2.4.1.173; sterol 3beta-glucosyltransferase [EC:2.4.1.173]
AVI_04158	K03832	tonB; periplasmic protein TonB
AVI_04159	K03560	tolR; biopolymer transport protein TolR
AVI_04160	K03559	exbD; biopolymer transport protein ExbD
AVI_04161	K03561	exbB; biopolymer transport protein ExbB
AVI_04163	K16087	TC.FEV.OM3, tbpA, hemR, lbpA, hpuB, bhuR, hugA, hmbR; hemoglobin/transferrin/lactoferrin receptor protein
AVI_04164	K07225	hmuS; putative hemin transport protein
AVI_04165	K25132	hmuT, phuT, bhuT, hemT; heme transport system substrate-binding protein
AVI_04166	K25133	hmuU, phuU, bhuU, hemU; heme transport system permease protein
AVI_04167	K10834	hmuV, phuV, bhuV, hemV; heme transport system ATP-binding protein [EC:7.6.2.5]
AVI_04178	K13472	raxST; sulfotransferase
AVI_04185	K08482	kaiC; circadian clock protein KaiC
AVI_04186	K08481	kaiB; circadian clock protein KaiB
AVI_04191	K00817	hisC; histidinol-phosphate aminotransferase [EC:2.6.1.9]
AVI_04192	K21282	ind7; N-demethylindolmycin N-methyltransferase [EC:2.1.1.328]
AVI_04194	K00018	hprA; glycerate dehydrogenase [EC:1.1.1.29]
AVI_04199	K18098	bjaR1, rpaR, rhiR; LuxR family transcriptional regulator, quorum-sensing system regulator BjaR1
AVI_04200	K01867	WARS, trpS; tryptophanyl-tRNA synthetase [EC:6.1.1.2]

AVI_04202	K07483	K07483; transposase
AVI_04204	K03733	xerC; integrase/recombinase XerC
AVI_04208	K03424	tatD; TatD DNase family protein [EC:3.1.21.-]
AVI_04216	K02911	RP-L32, MRPL32, rpmF; large subunit ribosomal protein L32
AVI_04217	K03621	plsX; phosphate acyltransferase [EC:2.3.1.274]
AVI_04218	K00648	fabH; 3-oxoacyl-[acyl-carrier-protein] synthase III [EC:2.3.1.180]
AVI_04220	K04764	ihfA, himA; integration host factor subunit alpha
AVI_04223	K01494	dcd; dCTP deaminase [EC:3.5.4.13]
AVI_04225	K06024	scpB; segregation and condensation protein B
AVI_04226	K05896	scpA; segregation and condensation protein A
AVI_04227	K01207	nagZ; beta-N-acetylhexosaminidase [EC:3.2.1.52]
AVI_04229	K01887	RARS, argS; arginyl-tRNA synthetase [EC:6.1.1.19]
AVI_04230	K01129	dgt; dGTPase [EC:3.1.5.1]
AVI_04233	K01142	E3.1.11.2, xthA; exodeoxyribonuclease III [EC:3.1.11.2]
AVI_04235	K06204	dksA; RNA polymerase-binding transcription factor
AVI_04241	K09989	K09989; uncharacterized protein
AVI_04245	K01262	pepP; Xaa-Pro aminopeptidase [EC:3.4.11.9]
AVI_04250	K07027	K07027; glycosyltransferase 2 family protein
AVI_04254	K03669	mdoH; membrane glycosyltransferase [EC:2.4.1.-]
AVI_04255	K03670	mdoG; periplasmic glucans biosynthesis protein
AVI_04260	K01497	ribA, RIB1; GTP cyclohydrolase II [EC:3.5.4.25]
AVI_04262	K01092	E3.1.3.25, IMPA, suhB; myo-inositol-1(or 4)-monophosphatase [EC:3.1.3.25]
AVI_04265	K11959	urtA; urea transport system substrate-binding protein
AVI_04266	K11960	urtB; urea transport system permease protein
AVI_04267	K11961	urtC; urea transport system permease protein
AVI_04268	K11962	urtD; urea transport system ATP-binding protein
AVI_04269	K11963	urtE; urea transport system ATP-binding protein
AVI_04271	K02042	phnE; phosphonate transport system permease protein
AVI_04272	K02042	phnE; phosphonate transport system permease protein
AVI_04273	K02044	phnD; phosphonate transport system substrate-binding protein
AVI_04274	K02041	phnC; phosphonate transport system ATP-binding protein [EC:7.3.2.2]
AVI_04277	K01822	E5.3.3.1; steroid Delta-isomerase [EC:5.3.3.1]
AVI_04278	K03631	recN; DNA repair protein RecN (Recombination protein N)
AVI_04279	K05807	bamD; outer membrane protein assembly factor BamD
AVI_04280	K02535	lpxC; UDP-3-O-[3-hydroxymyristoyl] N-acetylglucosamine deacetylase [EC:3.5.1.108]
AVI_04281	K03531	ftsZ; cell division protein FtsZ
AVI_04283	K03590	ftsA; cell division protein FtsA
AVI_04284	K03589	ftsQ; cell division protein FtsQ
AVI_04285	K01921	ddl; D-alanine-D-alanine ligase [EC:6.3.2.4]
AVI_04286	K00075	murB; UDP-N-acetylmuramate dehydrogenase [EC:1.3.1.98]
AVI_04289	K01924	murC; UDP-N-acetylmuramate--alanine ligase [EC:6.3.2.8]
AVI_04290	K02563	murG; UDP-N-acetylglucosamine--N-acetylmuramyl-(pentapeptide) pyrophosphoryl-undecaprenol N-acetylglucosamine transferase [EC:2.4.1.227]

AVI_04291	K03588	ftsW, spoVE; cell division protein FtsW
AVI_04293	K11991	tadA; tRNA(adenine34) deaminase [EC:3.5.4.33]
AVI_04294	K06178	rluB; 23S rRNA pseudouridine2605 synthase [EC:5.4.99.22]
AVI_04303	K10823	oppF; oligopeptide transport system ATP-binding protein
AVI_04306	K03111	ssb; single-strand DNA-binding protein
AVI_04307	K01735	aroB; 3-dehydroquinate synthase [EC:4.2.3.4]
AVI_04308	K00891	aroK, aroL; shikimate kinase [EC:2.7.1.71]
AVI_04311	K04763	xerD; integrase/recombinase XerD
AVI_04312	K03699	tlyC; magnesium and cobalt exporter, CNNM family
AVI_04313	K00681	ggt; gamma-glutamyltranspeptidase / glutathione hydrolase [EC:2.3.2.2 3.4.19.13]
AVI_04314	K06915	herA; DNA double-strand break repair helicase HerA and related ATPase
AVI_04316	K14660	nodE; nodulation protein E [EC:2.3.1.-]
AVI_04317	K02078	acpP; acyl carrier protein
AVI_04318	K02536	lpxD; UDP-3-O-[3-hydroxymyristoyl] glucosamine N-acyltransferase [EC:2.3.1.191]
AVI_04319	K21470	ycbB; L,D-transpeptidase YcbB
AVI_04322	K02051	ABC.SN.S; NitT/TauT family transport system substrate-binding protein
AVI_04323	K02049	ABC.SN.A; NitT/TauT family transport system ATP-binding protein
AVI_04324	K02050	ABC.SN.P; NitT/TauT family transport system permease protein
AVI_04325	K01968	MCCC1, accA1; 3-methylcrotonyl-CoA carboxylase alpha subunit [EC:6.4.1.4]
AVI_04326	K02355	fusA, GFM, EFG; elongation factor G
AVI_04327	K02992	RP-S7, MRPS7, rpsG; small subunit ribosomal protein S7
AVI_04328	K02950	RP-S12, MRPS12, rpsL; small subunit ribosomal protein S12
AVI_04331	K03046	rpoC; DNA-directed RNA polymerase subunit beta' [EC:2.7.7.6]
AVI_04332	K03043	rpoB; DNA-directed RNA polymerase subunit beta [EC:2.7.7.6]
AVI_04333	K02935	RP-L7, MRPL12, rplL; large subunit ribosomal protein L7/L12
AVI_04334	K02864	RP-L10, MRPL10, rplJ; large subunit ribosomal protein L10
AVI_04336	K02863	RP-L1, MRPL1, rplA; large subunit ribosomal protein L1
AVI_04337	K02867	RP-L11, MRPL11, rplK; large subunit ribosomal protein L11
AVI_04338	K02601	nusG; transcription termination/antitermination protein NusG
AVI_04339	K03073	secE; preprotein translocase subunit SecE
AVI_04340	K07305	msrB; peptide-methionine (R)-S-oxide reductase [EC:1.8.4.12]
AVI_04343	K19746	dauA; D-arginine dehydrogenase [EC:1.4.99.6]
AVI_04346	K02033	ABC.PE.P; peptide/nickel transport system permease protein
AVI_04347	K02034	ABC.PE.P1; peptide/nickel transport system permease protein
AVI_04348	K02031	ddpD; peptide/nickel transport system ATP-binding protein
AVI_04349	K02032	ddpF; peptide/nickel transport system ATP-binding protein
AVI_04365	K01915	glnA, GLUL; glutamine synthetase [EC:6.3.1.2]
AVI_04373	K07483	K07483; transposase
AVI_04374	K06918	K06918; uncharacterized protein
AVI_04375	K08990	ycjF; putative membrane protein
AVI_04376	K00384	trxB, TRR; thioredoxin reductase (NADPH) [EC:1.8.1.9]
AVI_04386	K07303	iorB; isoquinoline 1-oxidoreductase subunit beta [EC:1.3.99.16]

AVI_04387	K08738	CYC; cytochrome c
AVI_04391	K06996	K06996; uncharacterized protein
AVI_04392	K06949	rsgA, engC; ribosome biogenesis GTPase / thiamine phosphate phosphatase [EC:3.6.1.- 3.1.3.100]
AVI_04395	K11741	sugE; quaternary ammonium compound-resistance protein SugE
AVI_04429	K07486	K07486; transposase
AVI_04432	K07497	K07497; putative transposase
AVI_04433	K07497	K07497; putative transposase
AVI_04434	K07497	K07497; putative transposase
AVI_04435	K07497	K07497; putative transposase
AVI_04441	K07487	K07487; transposase

Table S5. KEGG analysis results for the genome of strain KACC 19432^{T}.

AIN_00001	K02500	hisF; imidazole glycerol-phosphate synthase subunit HisF [EC:4.3.2.10]
AIN_00002	K01523	hisE; phosphoribosyl-ATP pyrophosphohydrolase [EC:3.6.1.31]
AIN_00003	K06929	K06929; uncharacterized protein
AIN_00006	K03218	rlmB; 23S rRNA (guanosine2251-2'-O)-methyltransferase [EC:2.1.1.185]
AIN_00008	K00010	iolG; myo-inositol 2-dehydrogenase / D-chiro-inositol 1-dehydrogenase [EC:1.1.1.18 1.1.1.369]
AIN_00009	K03710	K03710; GntR family transcriptional regulator
AIN_00010	K26058	smoC, squD; sulfoquinovose monooxygenase [EC:1.14.14.181]
AIN_00011	K26057	smoB, squF; 6-dehydroglucose reductase [EC:1.1.1.432]
AIN_00015	K21395	yiaO; TRAP-type transport system periplasmic protein
AIN_00017	K00428	E1.11.1.5; cytochrome c peroxidase [EC:1.11.1.5]
AIN_00019	K01805	xylA; xylose isomerase [EC:5.3.1.5]
AIN_00020	K00854	xylB, XYLB; xylulokinase [EC:2.7.1.17]
AIN_00021	K10545	xylG; D-xylose transport system ATP-binding protein [EC:7.5.2.10]
AIN_00022	K10544	xylH; D-xylose transport system permease protein
AIN_00023	K10543	xylF; D-xylose transport system substrate-binding protein
AIN_00026	K00428	E1.11.1.5; cytochrome c peroxidase [EC:1.11.1.5]
AIN_00029	K14160	imuA; protein ImuA
AIN_00030	K14161	imuB; protein ImuB
AIN_00031	K14162	dnaE2; error-prone DNA polymerase [EC:2.7.7.7]
AIN_00036	K07497	K07497; putative transposase
AIN_00037	K07497	K07497; putative transposase
AIN_00046	K21898	orr; ornithine racemase [EC:5.1.1.12]
AIN_00057	K06919	K06919; putative DNA primase/helicase
AIN_00071	K14414	rtcR; transcriptional regulatory protein RtcR
AIN_00072	K11089	TROVE2, SSA2; 60 kDa SS-A/Ro ribonucleoprotein
AIN_00074	K07559	kptA; putative RNA 2'-phosphotransferase [EC:2.7.1.-]
AIN_00075	K00428	E1.11.1.5; cytochrome c peroxidase [EC:1.11.1.5]
AIN_00079	K03310	TC.AGCS; alanine or glycine:cation symporter, AGCS family
AIN_00080	K06145	gntR; LacI family transcriptional regulator, gluconate utilization system Gnt-I transcriptional repressor
AIN_00084	K02030	ABC.PA.S; polar amino acid transport system substrate-binding protein
AIN_00085	K22186	xylD; xylonate dehydratase [EC:4.2.1.82]
AIN_00087	K00874	kdgK; 2-dehydro-3-deoxygluconokinase [EC:2.7.1.45]
AIN_00088	K07395	K07395; putative proteasome-type protease
AIN_00092	K01835	pgm; phosphoglucomutase [EC:5.4.2.2]
AIN_00093	K01214	ISA, treX; isoamylase [EC:3.2.1.68]
AIN_00094	K00703	glgA; starch synthase [EC:2.4.1.21]
AIN_00095	K00975	glgC; glucose-1-phosphate adenylyltransferase [EC:2.7.7.27]
AIN_00096	K00700	GBE1, glgB; 1,4-alpha-glucan branching enzyme [EC:2.4.1.18]
AIN_00097	K00688	PYG, glgP; glycogen phosphorylase [EC:2.4.1.1]
AIN_00101	K00249	ACADM, acd; acyl-CoA dehydrogenase [EC:1.3.8.7]
AIN_00104	K02416	fliM; flagellar motor switch protein FliM

AIN_00108	K07110	ramB; XRE family transcriptional regulator, fatty acid utilization regulator
AIN_00109	K01999	livK; branched-chain amino acid transport system substrate-binding protein
AIN_00110	K01997	livH; branched-chain amino acid transport system permease protein
AIN_00112	K01995	livG; branched-chain amino acid transport system ATP-binding protein
AIN_00113	K01996	livF; branched-chain amino acid transport system ATP-binding protein
AIN_00114	K01895	ACSS1_2, acs; acetyl-CoA synthetase [EC:6.2.1.1]
AIN_00117	K02160	accB, bccP; acetyl-CoA carboxylase biotin carboxyl carrier protein
AIN_00118	K01961	accC; acetyl-CoA carboxylase, biotin carboxylase subunit [EC:6.4.1.2 6.3.4.14]
AIN_00119	K00684	aat; leucyl/phenylalanyl-tRNA---protein transferase [EC:2.3.2.6]
AIN_00123	K03544	clpX, CLPX; ATP-dependent Clp protease ATP-binding subunit ClpX
AIN_00124	K01358	clpP, CLPP; ATP-dependent Clp protease, protease subunit [EC:3.4.21.92]
AIN_00128	K03088	rpoE; RNA polymerase sigma-70 factor, ECF subfamily
AIN_00131	K07058	K07058; membrane protein
AIN_00143	K01652	E2.2.1.6L, ilvB, ilvG, ilvI; acetolactate synthase I/II/III large subunit [EC:2.2.1.6]
AIN_00147	K03670	mdoG; periplasmic glucans biosynthesis protein
AIN_00148	K03669	mdoH; membrane glycosyltransferase [EC:2.4.1.-]
AIN_00149	K03932	lpqC; polyhydroxybutyrate depolymerase
AIN_00153	K26939	norM, mdtK; MATE family, multidrug efflux pump
AIN_00155	K02622	parE; topoisomerase IV subunit B [EC:5.6.2.2]
AIN_00156	K09803	K09803; uncharacterized protein
AIN_00158	K09936	TC.BAT2; bacterial/archaeal transporter family-2 protein
AIN_00160	K00525	E1.17.4.1A, nrdA, nrdE; ribonucleoside-diphosphate reductase alpha chain [EC:1.17.4.1]
AIN_00162	K00765	hisG; ATP phosphoribosyltransferase [EC:2.4.2.17]
AIN_00163	K02502	hisZ; ATP phosphoribosyltransferase regulatory subunit
AIN_00164	K01892	HARS, hisS; histidyl-tRNA synthetase [EC:6.1.1.21]
AIN_00165	K03745	slyX; SlyX protein
AIN_00169	K02337	dnaE; DNA polymerase III subunit alpha [EC:2.7.7.7]
AIN_00172	K13481	xdhA; xanthine dehydrogenase small subunit [EC:1.17.1.4]
AIN_00173	K13482	xdhB; xanthine dehydrogenase large subunit [EC:1.17.1.4]
AIN_00174	K07402	xdhC; xanthine dehydrogenase accessory factor
AIN_00175	K23537	nupA; general nucleoside transport system ATP-binding protein
AIN_00176	K23535	nupB; general nucleoside transport system permease protein
AIN_00177	K23536	nupC; general nucleoside transport system permease protein
AIN_00178	K07335	bmpA, bmpB, tmpC; basic membrane protein A and related proteins
AIN_00180	K00315	DMGDH; dimethylglycine dehydrogenase [EC:1.5.8.4]
AIN_00181	K01918	panC; pantoate--beta-alanine ligase [EC:6.3.2.1]
AIN_00182	K00606	panB; 3-methyl-2-oxobutanoate hydroxymethyltransferase [EC:2.1.2.11]
AIN_00183	K00858	ppnK, NADK; NAD+ kinase [EC:2.7.1.23]
AIN_00184	K00600	glyA, SHMT; glycine hydroxymethyltransferase [EC:2.1.2.1]
AIN_00186	K01175	ybfF; esterase [EC:3.1.-.-]
AIN_00192	K03596	lepA; GTP-binding protein LepA
AIN_00193	K18923	stbD; antitoxin StbD

AIN_00194	K07341	doc; death on curing protein
AIN_00195	K06048	gshA, ybdK; glutamate---cysteine ligase / carboxylate-amine ligase [EC:6.3.2.2 6.3.-.-]
AIN_00197	K05564	phaG; multicomponent K+:H+ antiporter subunit G
AIN_00198	K05563	phaF; multicomponent K+:H+ antiporter subunit F
AIN_00199	K05562	phaE; multicomponent K+:H+ antiporter subunit E
AIN_00200	K05561	phaD; multicomponent K+:H+ antiporter subunit D
AIN_00201	K05560	phaC; multicomponent K+:H+ antiporter subunit C
AIN_00202	K05559	phaA; multicomponent K+:H+ antiporter subunit A
AIN_00203	K07093	K07093; uncharacterized protein
AIN_00205	K09982	K09982; uncharacterized protein
AIN_00212	K02002	proX; glycine betaine/proline transport system substrate-binding protein
AIN_00213	K02001	proW; glycine betaine/proline transport system permease protein
AIN_00214	K02000	proV; glycine betaine/proline transport system ATP-binding protein [EC:7.6.2.9]
AIN_00215	K01990	ABC-2.A; ABC-2 type transport system ATP-binding protein
AIN_00218	K02051	ABC.SN.S; NitT/TauT family transport system substrate-binding protein
AIN_00219	K02049	ABC.SN.A; NitT/TauT family transport system ATP-binding protein
AIN_00220	K02050	ABC.SN.P; NitT/TauT family transport system permease protein
AIN_00222	K05712	mhpA; 3-(3-hydroxy-phenyl)propionate hydroxylase [EC:1.14.13.127]
AIN_00228	K01912	paaK; phenylacetate-CoA ligase [EC:6.2.1.30]
AIN_00229	K02614	paaI; acyl-CoA thioesterase [EC:3.1.2.-]
AIN_00230	K02618	paaZ; oxepin-CoA hydrolase / 3-oxo-5,6-dehydrosuberyl-CoA semialdehyde dehydrogenase [EC:3.3.2.12 1.2.1.91]
AIN_00231	K02616	paaX; phenylacetic acid degradation operon negative regulatory protein
AIN_00232	K02613	paaE; ring-1,2-phenylacetyl-CoA epoxidase subunit PaaE
AIN_00233	K02612	paaD; ring-1,2-phenylacetyl-CoA epoxidase subunit PaaD
AIN_00234	K02611	paaC; ring-1,2-phenylacetyl-CoA epoxidase subunit PaaC [EC:1.14.13.149]
AIN_00235	K02609	paaA; ring-1,2-phenylacetyl-CoA epoxidase subunit PaaA [EC:1.14.13.149]
AIN_00236	K09947	K09947; uncharacterized protein
AIN_00237	K07338	K07338; uncharacterized protein
AIN_00240	K04047	dps; starvation-inducible DNA-binding protein
AIN_00241	K06938	K06938; uncharacterized protein
AIN_00242	K07231	K07231; putative iron-regulated protein
AIN_00246	K09862	K09862; uncharacterized protein
AIN_00248	K06287	yhdE; nucleoside triphosphate pyrophosphatase [EC:3.6.1.-]
AIN_00249	K02518	infA; translation initiation factor IF-1
AIN_00251	K07010	K07010; putative glutamine amidotransferase
AIN_00255	K00013	hisD; histidinol dehydrogenase [EC:1.1.1.23]
AIN_00258	K00790	murA; UDP-N-acetylglucosamine 1-carboxyvinyltransferase [EC:2.5.1.7]
AIN_00260	K09810	lolD; lipoprotein-releasing system ATP-binding protein [EC:7.6.2.-]
AIN_00261	K09808	lolC_E; lipoprotein-releasing system permease protein
AIN_00262	K02035	ABC.PE.S; peptide/nickel transport system substrate-binding protein
AIN_00263	K01881	PARS, proS; prolyl-tRNA synthetase [EC:6.1.1.15]
AIN_00266	K00937	ppk1; polyphosphate kinase [EC:2.7.4.1]

AIN_00267	K01524	ppx-gppA; exopolyphosphatase / guanosine-5'-triphosphate,3'-diphosphate pyrophosphatase [EC:3.6.1.11 3.6.1.40]
AIN_00270	K05801	djlA; DnaJ like chaperone protein
AIN_00271	K01847	MUT; methylmalonyl-CoA mutase [EC:5.4.99.2]
AIN_00274	K01965	PCCA, pccA; propionyl-CoA carboxylase alpha chain [EC:6.4.1.3]
AIN_00279	K01966	PCCB, pccB; propionyl-CoA carboxylase beta chain [EC:6.4.1.3 2.1.3.15]
AIN_00280	K07552	bcr, tcaB; MFS transporter, DHA1 family, multidrug resistance protein
AIN_00281	K21686	prpR; XRE family transcriptional regulator, fatty acid utilization regulator
AIN_00287	K09769	ymdB; 2',3'-cyclic-nucleotide 2'-phosphodiesterase [EC:3.1.4.16]
AIN_00288	K01934	MTHFS; 5-formyltetrahydrofolate cyclo-ligase [EC:6.3.3.2]
AIN_00289	K06213	mgtE; magnesium transporter
AIN_00290	K01487	guaD, GDA; guanine deaminase [EC:3.5.4.3]
AIN_00294	K00344	qor, CRYZ; NADPH:quinone reductase [EC:1.6.5.5]
AIN_00297	K06890	K06890; uncharacterized protein
AIN_00299	K02913	RP-L33, MRPL33, rpmG; large subunit ribosomal protein L33
AIN_00301	K01447	xlyAB; N-acetylmuramoyl-L-alanine amidase [EC:3.5.1.28]
AIN_00303	K02433	gatA, QRSL1; aspartyl-tRNA(Asn)/glutamyl-tRNA(Gln) amidotransferase subunit A [EC:6.3.5.6 6.3.5.7]
AIN_00304	K02435	gatC, GATC; aspartyl-tRNA(Asn)/glutamyl-tRNA(Gln) amidotransferase subunit C [EC:6.3.5.6 6.3.5.7]
AIN_00306	K11991	tadA; tRNA(adenine34) deaminase [EC:3.5.4.33]
AIN_00307	K06178	rluB; 23S rRNA pseudouridine2605 synthase [EC:5.4.99.22]
AIN_00314	K00847	E2.7.1.4, scrK; fructokinase [EC:2.7.1.4]
AIN_00315	K07560	dtd, DTD; D-aminoacyl-tRNA deacylase [EC:3.1.1.96]
AIN_00320	K03088	rpoE; RNA polymerase sigma-70 factor, ECF subfamily
AIN_00325	K26937	dinF, mepA, vmrA; MATE family, multidrug efflux pump
AIN_00326	K03168	topA; DNA topoisomerase I [EC:5.6.2.1]
AIN_00329	K21990	yfdC; formate-nitrite transporter family protein
AIN_00330	K03576	metR; LysR family transcriptional regulator, regulator for metE and metH
AIN_00331	K00297	metF, MTHFR; methylenetetrahydrofolate reductase (NADH) [EC:1.5.1.54]
AIN_00334	K03520	coxL, cutL; aerobic carbon-monoxide dehydrogenase large subunit [EC:1.2.5.3]
AIN_00336	K01873	VARS, valS; valyl-tRNA synthetase [EC:6.1.1.9]
AIN_00338	K00830	AGXT; alanine-glyoxylate transaminase / serine-glyoxylate transaminase / serine-pyruvate transaminase [EC:2.6.1.44 2.6.1.45 2.6.1.51]
AIN_00339	K11529	gck, gckA, GLYCTK; glycerate 2-kinase [EC:2.7.1.165]
AIN_00340	K15580	oppA, mppA; oligopeptide transport system substrate-binding protein
AIN_00341	K15581	oppB; oligopeptide transport system permease protein
AIN_00342	K15582	oppC; oligopeptide transport system permease protein
AIN_00343	K15583	oppD; oligopeptide transport system ATP-binding protein
AIN_00344	K10823	oppF; oligopeptide transport system ATP-binding protein
AIN_00345	K07114	yfbK; Ca-activated chloride channel homolog
AIN_00346	K03088	rpoE; RNA polymerase sigma-70 factor, ECF subfamily
AIN_00347	K06118	SQD1, sqdB; UDP-sulfoquinovose synthase [EC:3.13.1.1]
AIN_00352	K00135	gabD; succinate-semialdehyde dehydrogenase / glutarate-semialdehyde dehydrogenase [EC:1.2.1.16 1.2.1.79 1.2.1.20]
AIN_00354	K09935	ybiA; N-glycosidase YbiA [EC:3.2.2.-]
AIN_00355	K01246	tag; DNA-3-methyladenine glycosylase I [EC:3.2.2.20]

AIN_00356	K06147	ABCB-BAC; ATP-binding cassette, subfamily B, bacterial
AIN_00360	K11751	ushA; 5'-nucleotidase / UDP-sugar diphosphatase [EC:3.1.3.5 3.6.1.45]
AIN_00362	K00254	DHODH, pyrD; dihydroorotate dehydrogenase [EC:1.3.5.2]
AIN_00363	K00537	arsC; arsenate reductase (glutaredoxin) [EC:1.20.4.1]
AIN_00364	K26937	dinF, mepA, vmrA; MATE family, multidrug efflux pump
AIN_00370	K20035	dmdC; 3-(methylsulfanyl)propanoyl-CoA dehydrogenase [EC:1.3.99.41]
AIN_00372	K00799	GST, gst; glutathione S-transferase [EC:2.5.1.18]
AIN_00373	K00626	ACAT, atoB; acetyl-CoA C-acetyltransferase [EC:2.3.1.9]
AIN_00375	K01782	fadJ; 3-hydroxyacyl-CoA dehydrogenase / enoyl-CoA hydratase / 3-hydroxybutyryl-CoA epimerase [EC:1.1.1.35 4.2.1.17 5.1.2.3]
AIN_00376	K20034	dmdB; 3-(methylthio)propionyl---CoA ligase [EC:6.2.1.44]
AIN_00378	K00761	upp, UPRT; uracil phosphoribosyltransferase [EC:2.4.2.9]
AIN_00379	K01488	add, ADA; adenosine deaminase [EC:3.5.4.4]
AIN_00380	K01839	deoB; phosphopentomutase [EC:5.4.2.7]
AIN_00381	K00758	deoA, TYMP; thymidine phosphorylase [EC:2.4.2.4]
AIN_00382	K01489	cdd, CDA; cytidine deaminase [EC:3.5.4.5]
AIN_00385	K00029	maeB; malate dehydrogenase (oxaloacetate-decarboxylating)(NADP+) [EC:1.1.1.40]
AIN_00386	K01908	ACSS3, prpE; propionyl-CoA synthetase [EC:6.2.1.17]
AIN_00390	K00015	gyaR, GOR1; glyoxylate reductase [EC:1.1.1.26]
AIN_00393	K00568	ubiG; 2-polyprenyl-6-hydroxyphenyl methylase / 3-demethylubiquinone-9 3-methyltransferase [EC:2.1.1.222 2.1.1.64]
AIN_00397	K14393	actP; cation/acetate symporter
AIN_00398	K07182	K07182; CBS domain-containing protein
AIN_00401	K01284	dcp; peptidyl-dipeptidase Dcp [EC:3.4.15.5]
AIN_00402	K02029	ABC.PA.P; polar amino acid transport system permease protein
AIN_00403	K02030	ABC.PA.S; polar amino acid transport system substrate-binding protein
AIN_00405	K01520	dut, DUT; dUTP diphosphatase [EC:3.6.1.23]
AIN_00406	K13038	coaBC, dfp; phosphopantothenoylcysteine decarboxylase / phosphopantothenate---cysteine ligase [EC:4.1.1.36 6.3.2.5]
AIN_00408	K03089	rpoH; RNA polymerase sigma-32 factor
AIN_00409	K02231	cobP, cobU; adenosylcobinamide kinase / adenosylcobinamide-phosphate guanylyltransferase [EC:2.7.1.156 2.7.7.62]
AIN_00411	K00799	GST, gst; glutathione S-transferase [EC:2.5.1.18]
AIN_00412	K07391	comM; magnesium chelatase family protein
AIN_00413	K01920	gshB; glutathione synthase [EC:6.3.2.3]
AIN_00414	K07460	yraN; putative endonuclease
AIN_00415	K07056	rsmI; 16S rRNA (cytidine1402-2'-O)-methyltransferase [EC:2.1.1.198]
AIN_00417	K00990	glnD; [protein-PII] uridylyltransferase [EC:2.7.7.59]
AIN_00418	K03980	murJ, mviN; putative peptidoglycan lipid II flippase
AIN_00420	K01867	WARS, trpS; tryptophanyl-tRNA synthetase [EC:6.1.1.2]
AIN_00426	K14742	tsaB; tRNA threonylcarbamoyladenosine biosynthesis protein TsaB
AIN_00427	K03789	rimI; [ribosomal protein S18]-alanine N-acetyltransferase [EC:2.3.1.266]
AIN_00428	K07335	bmpA, bmpB, tmpC; basic membrane protein A and related proteins
AIN_00429	K23537	nupA; general nucleoside transport system ATP-binding protein
AIN_00431	K23535	nupB; general nucleoside transport system permease protein
AIN_00432	K23536	nupC; general nucleoside transport system permease protein

AIN_00433	K07090	K07090; uncharacterized protein
AIN_00436	K20249	raiI; acyl homoserine lactone synthase [EC:2.3.1.184]
AIN_00437	K25873	raiR; LuxR family transcriptional regulator, quorum-sensing system regulator RaiR
AIN_00438	K14446	ccr; crotonyl-CoA carboxylase/reductase [EC:1.3.1.85]
AIN_00440	K14447	ecm; ethylmalonyl-CoA mutase [EC:5.4.99.63]
AIN_00441	K03746	hns; DNA-binding protein H-NS
AIN_00442	K03784	deoD; purine-nucleoside phosphorylase [EC:2.4.2.1]
AIN_00445	K09967	K09967; uncharacterized protein
AIN_00446	K06143	creD; inner membrane protein
AIN_00447	K00240	sdhB, frdB; succinate dehydrogenase iron-sulfur subunit [EC:1.3.5.1]
AIN_00451	K00239	sdhA, frdA; succinate dehydrogenase flavoprotein subunit [EC:1.3.5.1]
AIN_00452	K00242	sdhD, frdD; succinate dehydrogenase membrane anchor subunit
AIN_00453	K00241	sdhC, frdC; succinate dehydrogenase cytochrome b subunit
AIN_00455	K14449	mch, mcd; 2-methylfumaryl-CoA hydratase [EC:4.2.1.148]
AIN_00458	K14451	mcl2; (3S)-malyl-CoA thioesterase [EC:3.1.2.30]
AIN_00460	K00024	mdh; malate dehydrogenase [EC:1.1.1.37]
AIN_00462	K01903	sucC; succinyl-CoA synthetase beta subunit [EC:6.2.1.5]
AIN_00467	K01902	sucD; succinyl-CoA synthetase alpha subunit [EC:6.2.1.5]
AIN_00471	K00164	OGDH, sucA; 2-oxoglutarate dehydrogenase E1 component [EC:1.2.4.2]
AIN_00472	K00658	DLST, sucB; 2-oxoglutarate dehydrogenase E2 component (dihydrolipoamide succinyltransferase) [EC:2.3.1.61]
AIN_00477	K00382	DLD, lpd, pdhD; dihydrolipoyl dehydrogenase [EC:1.8.1.4]
AIN_00481	K11894	impI, vasC; type VI secretion system protein ImpI
AIN_00482	K11902	impA; type VI secretion system protein ImpA
AIN_00483	K11901	impB; type VI secretion system protein ImpB
AIN_00484	K11900	impC; type VI secretion system protein ImpC
AIN_00485	K11903	hcp; type VI secretion system secreted protein Hcp
AIN_00486	K11897	impF; type VI secretion system protein ImpF
AIN_00487	K11896	impG, vasA; type VI secretion system protein ImpG
AIN_00488	K11895	impH, vasB; type VI secretion system protein ImpH
AIN_00489	K11907	vasG, clpV; type VI secretion system protein VasG
AIN_00495	K03561	exbB; biopolymer transport protein ExbB
AIN_00496	K03559	exbD; biopolymer transport protein ExbD
AIN_00499	K08884	K08884; serine/threonine protein kinase, bacterial [EC:2.7.11.1]
AIN_00501	K11890	impM; type VI secretion system protein ImpM
AIN_00502	K11891	impL, vasK, icmF; type VI secretion system protein ImpL
AIN_00503	K11892	impK, ompA, vasF, dotU; type VI secretion system protein ImpK
AIN_00504	K11893	impJ, vasE; type VI secretion system protein ImpJ
AIN_00506	K05779	ynjD; putative thiamine transport system ATP-binding protein
AIN_00507	K05778	ynjC; putative thiamine transport system permease protein
AIN_00508	K05777	ynjB; putative thiamine transport system substrate-binding protein
AIN_00510	K00383	GSR, gor; glutathione reductase (NADPH) [EC:1.8.1.7]
AIN_00511	K01187	malZ; alpha-glucosidase [EC:3.2.1.20]

AIN_00512	K17324	glpS; glycerol transport system ATP-binding protein
AIN_00513	K17325	glpT; glycerol transport system ATP-binding protein
AIN_00514	K17322	glpP; glycerol transport system permease protein
AIN_00515	K17323	glpQ; glycerol transport system permease protein
AIN_00517	K17321	glpV; glycerol transport system substrate-binding protein
AIN_00519	K01087	otsB; trehalose 6-phosphate phosphatase [EC:3.1.3.12]
AIN_00520	K00697	otsA; trehalose 6-phosphate synthase [EC:2.4.1.15 2.4.1.347]
AIN_00524	K04080	ibpA; molecular chaperone IbpA
AIN_00526	K11473	glcF; glycolate dehydrogenase iron-sulfur subunit [EC:1.1.99.14]
AIN_00527	K11472	glcE; glycolate dehydrogenase FAD-binding subunit [EC:1.1.99.14]
AIN_00528	K00104	glcD; glycolate dehydrogenase FAD-linked subunit [EC:1.1.99.14]
AIN_00533	K03924	moxR; MoxR-like ATPase [EC:3.6.3.-]
AIN_00534	K09986	K09986; uncharacterized protein
AIN_00536	K06996	K06996; uncharacterized protein
AIN_00539	K02335	polA; DNA polymerase I [EC:2.7.7.7]
AIN_00542	K11606	sitD; manganese/iron transport system permease protein
AIN_00543	K11605	sitC; manganese/iron transport system permease protein
AIN_00544	K11607	sitB; manganese/iron transport system ATP-binding protein
AIN_00545	K11604	sitA; manganese/iron transport system substrate-binding protein
AIN_00546	K11747	kefB; glutathione-regulated potassium-efflux system protein KefB
AIN_00547	K11748	kefG; glutathione-regulated potassium-efflux system ancillary protein KefG
AIN_00548	K16171	faaH; fumarylacetoacetate (FAA) hydrolase [EC:3.7.1.2]
AIN_00550	K00451	HGD, hmgA; homogentisate 1,2-dioxygenase [EC:1.13.11.5]
AIN_00551	K15552	tauC; taurine transport system permease protein
AIN_00552	K10831	tauB; taurine transport system ATP-binding protein [EC:7.6.2.7]
AIN_00553	K15551	tauA; taurine transport system substrate-binding protein
AIN_00555	K03851	tpa; taurine-pyruvate aminotransferase [EC:2.6.1.77]
AIN_00559	K21307	soeA; sulfite dehydrogenase (quinone) subunit SoeA [EC:1.8.5.6]
AIN_00560	K20608	tet; tetrahedral aminopeptidase [EC:3.4.11.-]
AIN_00561	K21308	soeB; sulfite dehydrogenase (quinone) subunit SoeB
AIN_00562	K21309	soeC; sulfite dehydrogenase (quinone) subunit SoeC
AIN_00565	K10191	lacK; lactose/L-arabinose transport system ATP-binding protein
AIN_00567	K02027	ABC.MS.S; multiple sugar transport system substrate-binding protein
AIN_00568	K02025	ABC.MS.P; multiple sugar transport system permease protein
AIN_00570	K02026	ABC.MS.P1; multiple sugar transport system permease protein
AIN_00571	K07406	melA; alpha-galactosidase [EC:3.2.1.22]
AIN_00572	K22215	galD; galactose dehydrogenase [EC:1.1.1.48 1.1.1.120]
AIN_00573	K00883	dgoK; 2-dehydro-3-deoxygalactonokinase [EC:2.7.1.58]
AIN_00574	K01631	dgoA; 2-dehydro-3-deoxyphosphogalactonate aldolase [EC:4.1.2.21]
AIN_00576	K12308	bgaB, lacA; beta-galactosidase [EC:3.2.1.23]
AIN_00577	K01785	galM, GALM; aldose 1-epimerase [EC:5.1.3.3]
AIN_00579	K09781	K09781; uncharacterized protein

AIN_00580	K07127	uraH, pucM, hiuH; 5-hydroxyisourate hydrolase [EC:3.5.2.17]
AIN_00581	K16842	hpxB; allantoinase [EC:3.5.2.5]
AIN_00582	K14977	ylbA, UGHY; (S)-ureidoglycine aminohydrolase [EC:3.5.3.26]
AIN_00583	K01483	allA; ureidoglycolate lyase [EC:4.3.2.3]
AIN_00586	K03426	NUDT12_13, nudC; NAD+ diphosphatase [EC:3.6.1.22]
AIN_00587	K01119	cpdB; 2',3'-cyclic-nucleotide 2'-phosphodiesterase / 3'-nucleotidase [EC:3.1.4.16 3.1.3.6]
AIN_00590	K02343	dnaX; DNA polymerase III subunit gamma/tau [EC:2.7.7.7]
AIN_00591	K09747	ebfC; nucleoid-associated protein EbfC
AIN_00592	K06187	recR; recombination protein RecR
AIN_00593	K09987	K09987; uncharacterized protein
AIN_00594	K01166	RNASET2; ribonuclease T2 [EC:4.6.1.19]
AIN_00596	K03088	rpoE; RNA polymerase sigma-70 factor, ECF subfamily
AIN_00600	K18587	COQ9; ubiquinone biosynthesis protein COQ9
AIN_00603	K03282	mscL; large conductance mechanosensitive channel
AIN_00604	K11209	yghU, yfcG; GSH-dependent disulfide-bond oxidoreductase [EC:1.8.4.-]
AIN_00605	K01890	FARSB, pheT; phenylalanyl-tRNA synthetase beta chain [EC:6.1.1.20]
AIN_00607	K01889	FARSA, pheS; phenylalanyl-tRNA synthetase alpha chain [EC:6.1.1.20]
AIN_00609	K02887	RP-L20, MRPL20, rplT; large subunit ribosomal protein L20
AIN_00610	K02916	RP-L35, MRPL35, rpmI; large subunit ribosomal protein L35
AIN_00612	K00873	PK, pyk; pyruvate kinase [EC:2.7.1.40]
AIN_00614	K09948	K09948; uncharacterized protein
AIN_00617	K00824	dat; D-alanine transaminase [EC:2.6.1.21]
AIN_00618	K19802	ycjG, ykfB, dgcA; L-Ala-D/L-Glu epimerase / N-acetyl-D-glutamate racemase [EC:5.1.1.20 5.1.1.25]
AIN_00619	K26272	dgcN; D-glutamate N-acetyltransferase [EC:2.3.1.312]
AIN_00620	K08691	mcl; malyl-CoA/(S)-citramalyl-CoA lyase [EC:4.1.3.24 4.1.3.25]
AIN_00621	K13652	K13652; AraC family transcriptional regulator
AIN_00622	K01962	accA; acetyl-CoA carboxylase carboxyl transferase subunit alpha [EC:6.4.1.2 2.1.3.15]
AIN_00623	K00655	plsC; 1-acyl-sn-glycerol-3-phosphate acyltransferase [EC:2.3.1.51]
AIN_00624	K09811	ftsX; cell division transport system permease protein
AIN_00625	K09812	ftsE; cell division transport system ATP-binding protein
AIN_00628	K01586	lysA; diaminopimelate decarboxylase [EC:4.1.1.20]
AIN_00630	K18661	matB; malonyl-CoA/methylmalonyl-CoA synthetase [EC:6.2.1.76 6.2.1.-]
AIN_00631	K01578	MLYCD; malonyl-CoA decarboxylase [EC:4.1.1.9]
AIN_00638	K07080	K07080; uncharacterized protein
AIN_00642	K01755	argH, ASL; argininosuccinate lyase [EC:4.3.2.1]
AIN_00645	K01246	tag; DNA-3-methyladenine glycosylase I [EC:3.2.2.20]
AIN_00647	K00626	ACAT, atoB; acetyl-CoA C-acetyltransferase [EC:2.3.1.9]
AIN_00649	K00023	phbB; acetoacetyl-CoA reductase [EC:1.1.1.36]
AIN_00651	K03566	gcvA; LysR family transcriptional regulator, glycine cleavage system transcriptional activator
AIN_00655	K02523	ispB; octaprenyl-diphosphate synthase [EC:2.5.1.90]
AIN_00656	K00919	ispE; 4-diphosphocytidyl-2-C-methyl-D-erythritol kinase [EC:2.7.1.148]
AIN_00658	K00311	ETFDH; electron-transferring-flavoprotein dehydrogenase [EC:1.5.5.1]

AIN_00659	K03624	greA; transcription elongation factor GreA
AIN_00662	K03750	moeA; molybdopterin molybdotransferase [EC:2.10.1.1]
AIN_00663	K03753	mobB; molybdopterin-guanine dinucleotide biosynthesis adapter protein
AIN_00664	K03752	mobA; molybdenum cofactor guanylyltransferase [EC:2.7.7.77]
AIN_00665	K02379	fdhD; FdhD protein
AIN_00666	K26605	azlC, brnF; branched chain amino acid efflux pump
AIN_00667	K26606	azlD, brnE; branched chain amino acid efflux pump
AIN_00672	K20035	dmdC; 3-(methylsulfanyl)propanoyl-CoA dehydrogenase [EC:1.3.99.41]
AIN_00673	K07566	tsaC, rimN, SUA5, YRDC; L-threonylcarbamoyladenylate synthase [EC:2.7.7.87]
AIN_00683	K07735	algH; putative transcriptional regulator
AIN_00687	K03638	moaB; molybdopterin adenylyltransferase [EC:2.7.7.75]
AIN_00688	K21929	udg; uracil-DNA glycosylase [EC:3.2.2.27]
AIN_00689	K00609	pyrB, PYR2; aspartate carbamoyltransferase catalytic subunit [EC:2.1.3.2]
AIN_00691	K01465	URA4, pyrC; dihydroorotase [EC:3.5.2.3]
AIN_00692	K08591	plsY; acyl phosphate:glycerol-3-phosphate acyltransferase [EC:2.3.1.275]
AIN_00696	K01919	gshA; glutamate--cysteine ligase [EC:6.3.2.2]
AIN_00698	K09761	rsmE; 16S rRNA (uracil1498-N3)-methyltransferase [EC:2.1.1.193]
AIN_00699	K03179	ubiA; 4-hydroxybenzoate polyprenyltransferase [EC:2.5.1.39]
AIN_00700	K03286	TC.OOP; OmpA-OmpF porin, OOP family
AIN_00702	K03635	MOCS2B, moaE; molybdopterin synthase catalytic subunit [EC:2.8.1.12]
AIN_00703	K03636	moaD, cysO; sulfur-carrier protein
AIN_00704	K00995	pgsA, PGS1; CDP-diacylglycerol---glycerol-3-phosphate 3-phosphatidyltransferase [EC:2.7.8.5]
AIN_00705	K03703	uvrC; excinuclease ABC subunit C
AIN_00707	K07301	yrbG; cation:H+ antiporter
AIN_00708	K04774	sohB; serine protease SohB [EC:3.4.21.-]
AIN_00709	K01992	ABC-2.P; ABC-2 type transport system permease protein
AIN_00710	K01990	ABC-2.A; ABC-2 type transport system ATP-binding protein
AIN_00712	K10773	NTHL1, nth; endonuclease III [EC:3.2.2.- 4.2.99.18]
AIN_00718	K03852	xsc; sulfoacetaldehyde acetyltransferase [EC:2.3.3.15]
AIN_00719	K00108	betA, CHDH; choline dehydrogenase [EC:1.1.99.1]
AIN_00720	K00130	betB, gbsA; betaine-aldehyde dehydrogenase [EC:1.2.1.8]
AIN_00721	K02167	betI; TetR/AcrR family transcriptional regulator, transcriptional repressor of bet genes
AIN_00729	K17247	msrQ; methionine sulfoxide reductase heme-binding subunit
AIN_00730	K07147	msrP; methionine sulfoxide reductase catalytic subunit [EC:1.8.-.-]
AIN_00733	K09983	K09983; uncharacterized protein
AIN_00734	K03695	clpB; ATP-dependent Clp protease ATP-binding subunit ClpB
AIN_00736	K01591	pyrF; orotidine-5'-phosphate decarboxylase [EC:4.1.1.23]
AIN_00744	K03574	mutT, NUDT15, MTH2; 8-oxo-dGTP diphosphatase [EC:3.6.1.55]
AIN_00745	K02346	dinB; DNA polymerase IV [EC:2.7.7.7]
AIN_00748	K02919	RP-L36, MRPL36, rpmJ; large subunit ribosomal protein L36
AIN_00749	K07302	iorA; isoquinoline 1-oxidoreductase subunit alpha [EC:1.3.99.16]
AIN_00750	K07303	iorB; isoquinoline 1-oxidoreductase subunit beta [EC:1.3.99.16]

AIN_00753	K01996	livF; branched-chain amino acid transport system ATP-binding protein
AIN_00754	K01999	livK; branched-chain amino acid transport system substrate-binding protein
AIN_00755	K01998	livM; branched-chain amino acid transport system permease protein
AIN_00757	K01997	livH; branched-chain amino acid transport system permease protein
AIN_00758	K01995	livG; branched-chain amino acid transport system ATP-binding protein
AIN_00759	K01897	ACSL, fadD; long-chain acyl-CoA synthetase [EC:6.2.1.3]
AIN_00760	K00123	fdoG, fdhF, fdwA; formate dehydrogenase major subunit [EC:1.17.1.9]
AIN_00761	K00290	LYS1; saccharopine dehydrogenase (NAD+, L-lysine forming) [EC:1.5.1.7]
AIN_00763	K03292	TC.GPH; glycoside/pentoside/hexuronide:cation symporter, GPH family
AIN_00764	K09701	K09701; uncharacterized protein
AIN_00765	K06954	K06954; uncharacterized protein
AIN_00766	K03088	rpoE; RNA polymerase sigma-70 factor, ECF subfamily
AIN_00767	K07167	chrR; putative transcriptional regulator
AIN_00769	K09760	rmuC; DNA recombination protein RmuC
AIN_00770	K03572	mutL; DNA mismatch repair protein MutL
AIN_00771	K07263	pqqL; zinc protease [EC:3.4.24.-]
AIN_00772	K07263	pqqL; zinc protease [EC:3.4.24.-]
AIN_00774	K03101	lspA; signal peptidase II [EC:3.4.23.36]
AIN_00775	K00602	purH; phosphoribosylaminoimidazolecarboxamide formyltransferase / IMP cyclohydrolase [EC:2.1.2.3 3.5.4.10]
AIN_00777	K03500	rsmB, sun; 16S rRNA (cytosine967-C5)-methyltransferase [EC:2.1.1.176]
AIN_00780	K00215	dapB; 4-hydroxy-tetrahydrodipicolinate reductase [EC:1.17.1.8]
AIN_00781	K02834	rbfA; ribosome-binding factor A
AIN_00783	K03177	truB, PUS4, TRUB1; tRNA pseudouridine55 synthase [EC:5.4.99.25]
AIN_00785	K01561	dehH; haloacetate dehalogenase [EC:3.8.1.3]
AIN_00787	K02956	RP-S15, MRPS15, rpsO; small subunit ribosomal protein S15
AIN_00790	K07086	K07086; uncharacterized protein
AIN_00792	K00962	pnp, PNPT1; polyribonucleotide nucleotidyltransferase [EC:2.7.7.8]
AIN_00795	K24158	prx; thioredoxin-dependent peroxiredoxin [EC:1.11.1.24]
AIN_00797	K06177	rluA; tRNA pseudouridine32 synthase / 23S rRNA pseudouridine746 synthase [EC:5.4.99.28 5.4.99.29]
AIN_00799	K08968	msrC; L-methionine (R)-S-oxide reductase [EC:1.8.4.14]
AIN_00800	K00626	ACAT, atoB; acetyl-CoA C-acetyltransferase [EC:2.3.1.9]
AIN_00801	K04749	rsbV; anti-sigma B factor antagonist
AIN_00802	K04757	rsbW; serine/threonine-protein kinase RsbW [EC:2.7.11.1]
AIN_00803	K00681	ggt; gamma-glutamyltranspeptidase / glutathione hydrolase [EC:2.3.2.2 3.4.19.13]
AIN_00804	K11940	hspQ; heat shock protein HspQ
AIN_00808	K03466	ftsK, spoIIIE; DNA segregation ATPase FtsK/SpoIIIE, S-DNA-T family
AIN_00810	K02433	gatA, QRSL1; aspartyl-tRNA(Asn)/glutamyl-tRNA(Gln) amidotransferase subunit A [EC:6.3.5.6 6.3.5.7]
AIN_00811	K03185	ubiH; 2-octaprenyl-6-methoxyphenol hydroxylase [EC:1.14.13.-]
AIN_00812	K09791	K09791; uncharacterized protein
AIN_00813	K07157	K07157; uncharacterized protein
AIN_00814	K05838	ybbN; putative thioredoxin
AIN_00815	K01142	E3.1.11.2, xthA; exodeoxyribonuclease III [EC:3.1.11.2]

AIN_00817	K01533	copB; P-type Cu2+ transporter [EC:7.2.2.9]
AIN_00820	K00406	ccoP; cytochrome c oxidase cbb3-type subunit III
AIN_00821	K00407	ccoQ; cytochrome c oxidase cbb3-type subunit IV
AIN_00822	K00405	ccoO; cytochrome c oxidase cbb3-type subunit II
AIN_00823	K00404	ccoN; cytochrome c oxidase cbb3-type subunit I [EC:7.1.1.9]
AIN_00825	K01420	fnr; CRP/FNR family transcriptional regulator, anaerobic regulatory protein
AIN_00828	K01497	ribA, RIB1; GTP cyclohydrolase II [EC:3.5.4.25]
AIN_00829	K02040	pstS; phosphate transport system substrate-binding protein
AIN_00830	K07077	K07077; uncharacterized protein
AIN_00832	K04518	pheA2; prephenate dehydratase [EC:4.2.1.51]
AIN_00833	K08738	CYC; cytochrome c
AIN_00834	K13893	yejA; microcin C transport system substrate-binding protein
AIN_00835	K13894	yejB; microcin C transport system permease protein
AIN_00836	K13895	yejE; microcin C transport system permease protein
AIN_00837	K13896	yejF; microcin C transport system ATP-binding protein
AIN_00838	K01286	E3.4.16.4; D-alanyl-D-alanine carboxypeptidase [EC:3.4.16.4]
AIN_00839	K07025	K07025; putative hydrolase of the HAD superfamily
AIN_00840	K06891	clpS; ATP-dependent Clp protease adaptor protein ClpS
AIN_00841	K00564	rsmC; 16S rRNA (guanine1207-N2)-methyltransferase [EC:2.1.1.172]
AIN_00842	K00076	hdhA; 7-alpha-hydroxysteroid dehydrogenase [EC:1.1.1.159]
AIN_00844	K00228	CPOX, hemF; coproporphyrinogen III oxidase [EC:1.3.3.3]
AIN_00845	K01599	hemE, UROD; uroporphyrinogen decarboxylase [EC:4.1.1.37]
AIN_00846	K01749	hemC, HMBS; hydroxymethylbilane synthase [EC:2.5.1.61]
AIN_00849	K01823	idi, IDI; isopentenyl-diphosphate Delta-isomerase [EC:5.3.3.2]
AIN_00850	K10960	chlP, bchP; geranylgeranyl diphosphate/geranylgeranyl-bacteriochlorophyllide a reductase [EC:1.3.1.83 1.3.1.111]
AIN_00851	K08226	pucC; MFS transporter, BCD family, chlorophyll transporter
AIN_00852	K04040	chlG, bchG; chlorophyll/bacteriochlorophyll a synthase [EC:2.5.1.62 2.5.1.133]
AIN_00856	K11336	bchF; 3-vinyl bacteriochlorophyllide hydratase [EC:4.2.1.165]
AIN_00857	K04038	chlN; light-independent protochlorophyllide reductase subunit N [EC:1.3.7.7]
AIN_00858	K04039	chlB; light-independent protochlorophyllide reductase subunit B [EC:1.3.7.7]
AIN_00859	K03403	chlH, bchH; magnesium chelatase subunit H [EC:6.6.1.1]
AIN_00860	K04037	chlL; light-independent protochlorophyllide reductase subunit L [EC:1.3.7.7]
AIN_00861	K03428	bchM, chlM; magnesium-protoporphyrin O-methyltransferase [EC:2.1.1.11]
AIN_00862	K08226	pucC; MFS transporter, BCD family, chlorophyll transporter
AIN_00863	K13991	puhA; photosynthetic reaction center H subunit
AIN_00867	K04035	E1.14.13.81, acsF, chlE; magnesium-protoporphyrin IX monomethyl ester (oxidative) cyclase [EC:1.14.13.81]
AIN_00869	K00643	E2.3.1.37, ALAS; 5-aminolevulinate synthase [EC:2.3.1.37]
AIN_00870	K08738	CYC; cytochrome c
AIN_00871	K02470	gyrB; DNA gyrase subunit B [EC:5.6.2.2]
AIN_00872	K03629	recF; DNA replication and repair protein RecF
AIN_00873	K02338	dnaN; DNA polymerase III subunit beta [EC:2.7.7.7]
AIN_00874	K02313	dnaA; chromosomal replication initiator protein

AIN_00875	K02968	RP-S20, rpsT; small subunit ribosomal protein S20
AIN_00876	K01715	crt; enoyl-CoA hydratase [EC:4.2.1.17]
AIN_00877	K10563	mutM, fpg; formamidopyrimidine-DNA glycosylase [EC:3.2.2.23 4.2.99.18]
AIN_00878	K03183	ubiE; demethylmenaquinone methyltransferase / 2-methoxy-6-polyprenyl-1,4-benzoquinol methylase [EC:2.1.1.163 2.1.1.201]
AIN_00879	K03688	ubiB, aarF; ubiquinone biosynthesis protein
AIN_00880	K02389	flgD; flagellar basal-body rod modification protein FlgD
AIN_00882	K02395	flgJ; peptidoglycan hydrolase FlgJ
AIN_00886	K06601	flbT; flagellar biosynthesis repressor protein FlbT
AIN_00893	K02483	K02483; two-component system, OmpR family, response regulator
AIN_00906	K03657	uvrD, pcrA; ATP-dependent DNA helicase UvrD/PcrA [EC:5.6.2.4]
AIN_00909	K01778	dapF; diaminopimelate epimerase [EC:5.1.1.7]
AIN_00910	K18707	mtaB; threonylcarbamoyladenosine tRNA methylthiotransferase MtaB [EC:2.8.4.5]
AIN_00911	K07734	paiB; transcriptional regulator
AIN_00913	K12980	lpxQ; lipid A oxidase
AIN_00914	K00411	UQCRFS1, RIP1, petA; ubiquinol-cytochrome c reductase iron-sulfur subunit [EC:7.1.1.8]
AIN_00915	K00412	CYTB, petB; ubiquinol-cytochrome c reductase cytochrome b subunit
AIN_00916	K00413	CYC1, CYT1, petC; ubiquinol-cytochrome c reductase cytochrome c1 subunit
AIN_00919	K06153	bacA; undecaprenyl-diphosphatase [EC:3.6.1.27]
AIN_00920	K00266	gltD; glutamate synthase (NADPH) small chain [EC:1.4.1.13]
AIN_00924	K00265	gltB; glutamate synthase (NADPH) large chain [EC:1.4.1.13]
AIN_00925	K03814	mtgA; monofunctional glycosyltransferase [EC:2.4.99.28]
AIN_00926	K00799	GST, gst; glutathione S-transferase [EC:2.5.1.18]
AIN_00927	K18979	queG; epoxyqueuosine reductase [EC:1.17.99.6]
AIN_00928	K21430	yliI; aldose sugar dehydrogenase [EC:1.1.5.-]
AIN_00932	K00826	E2.6.1.42, ilvE; branched-chain amino acid aminotransferase [EC:2.6.1.42]
AIN_00934	K07659	ompR; two-component system, OmpR family, phosphate regulon response regulator OmpR
AIN_00936	K03602	xseB; exodeoxyribonuclease VII small subunit [EC:3.1.11.6]
AIN_00937	K00795	ispA; farnesyl diphosphate synthase [EC:2.5.1.1 2.5.1.10]
AIN_00938	K01662	dxs; 1-deoxy-D-xylulose-5-phosphate synthase [EC:2.2.1.7]
AIN_00939	K08714	VGSC; voltage-gated sodium channel
AIN_00943	K01486	ade; adenine deaminase [EC:3.5.4.2]
AIN_00944	K01241	amn; AMP nucleosidase [EC:3.2.2.4]
AIN_00945	K03530	hupB; DNA-binding protein HU-beta
AIN_00947	K01736	aroC; chorismate synthase [EC:4.2.3.5]
AIN_00948	K02064	thiB, tbpA; thiamine transport system substrate-binding protein
AIN_00949	K02063	thiP; thiamine transport system permease protein
AIN_00950	K02062	thiQ; thiamine transport system ATP-binding protein [EC:7.6.2.15]
AIN_00951	K04047	dps; starvation-inducible DNA-binding protein
AIN_00952	K07516	fadN; 3-hydroxyacyl-CoA dehydrogenase [EC:1.1.1.35]
AIN_00953	K00626	ACAT, atoB; acetyl-CoA C-acetyltransferase [EC:2.3.1.9]
AIN_00962	K03230	yscV, sctV, hrcV, ssaV, invA; type III secretion protein V
AIN_00963	K03229	yscU, sctU, hrcU, ssaU; type III secretion protein U

AIN_00964	K03228	yscT, sctT, hrcT, ssaT; type III secretion protein T
AIN_00965	K22508	spaQ; type III secretion system export apparatus protein
AIN_00966	K03226	yscR, sctR, hrcR, ssaR; type III secretion protein R
AIN_00969	K03224	yscN, sctN, hrcN, ssaN; ATP synthase in type III secretion protein N [EC:7.4.2.8]
AIN_00970	K03223	yscL, sctL; type III secretion protein L
AIN_00974	K11618	liaR; two-component system, NarL family, response regulator LiaR
AIN_00976	K10227	smoE, mtlE; polyol transport system substrate-binding protein
AIN_00977	K10228	smoF, mtlF; polyol transport system permease protein
AIN_00978	K10229	smoG, mtlG; polyol transport system permease protein
AIN_00979	K10111	malK, mtlK, thuK; multiple sugar transport system ATP-binding protein [EC:7.5.2.-]
AIN_00980	K21620	sorbD; galactitol 2-dehydrogenase [EC:1.1.1.16]
AIN_00981	K00045	E1.1.1.67, mtlK; mannitol 2-dehydrogenase [EC:1.1.1.67]
AIN_00982	K00820	glmS, GFPT; glutamine---fructose-6-phosphate transaminase (isomerizing) [EC:2.6.1.16]
AIN_00983	K01443	nagA, AMDHD2; N-acetylglucosamine-6-phosphate deacetylase [EC:3.5.1.25]
AIN_00984	K00281	GLDC, gcvP; glycine cleavage system P protein (glycine dehydrogenase) [EC:1.4.4.2]
AIN_00985	K02437	gcvH, GCSH; glycine cleavage system H protein
AIN_00986	K00605	gcvT, AMT; glycine cleavage system T protein (aminomethyltransferase) [EC:2.1.2.10]
AIN_00993	K01915	glnA, GLUL; glutamine synthetase [EC:6.3.1.2]
AIN_00997	K00981	E2.7.7.41, CDS1, CDS2, cdsA; phosphatidate cytidylyltransferase [EC:2.7.7.41]
AIN_00999	K01611	speD, AMD1; S-adenosylmethionine decarboxylase [EC:4.1.1.50]
AIN_01002	K00797	speE, SRM, SPE3; spermidine synthase [EC:2.5.1.16]
AIN_01003	K03684	rnd; ribonuclease D [EC:3.1.13.5]
AIN_01004	K11719	lptC; lipopolysaccharide export system protein LptC
AIN_01005	K09774	lptA; lipopolysaccharide export system protein LptA
AIN_01006	K06861	lptB; lipopolysaccharide export system ATP-binding protein [EC:7.5.2.5]
AIN_01007	K05808	hpf; ribosome hibernation promoting factor
AIN_01008	K02806	ptsN; nitrogen PTS system EIIA component [EC:2.7.1.-]
AIN_01013	K01784	galE, GALE; UDP-glucose 4-epimerase [EC:5.1.3.2]
AIN_01014	K00963	UGP2, galU, galF; UTP--glucose-1-phosphate uridylyltransferase [EC:2.7.7.9]
AIN_01016	K00979	kdsB; 3-deoxy-manno-octulosonate cytidylyltransferase (CMP-KDO synthetase) [EC:2.7.7.38]
AIN_01017	K01082	cysQ, MET22, BPNT1; 3'(2'), 5'-bisphosphate nucleotidase [EC:3.1.3.7]
AIN_01021	K07793	tctA; putative tricarboxylic transport membrane protein
AIN_01023	K07774	tctD; two-component system, OmpR family, response regulator TctD
AIN_01024	K07649	tctE; two-component system, OmpR family, sensor histidine kinase TctE [EC:2.7.13.3]
AIN_01025	K07120	K07120; uncharacterized protein
AIN_01027	K13796	cobZ, tcuA; tricarballylate dehydrogenase
AIN_01028	K13795	citB, tcuB; citrate/tricarballylate utilization protein
AIN_01029	K04043	dnaK, HSPA9; molecular chaperone DnaK
AIN_01030	K03686	dnaJ; molecular chaperone DnaJ
AIN_01031	K03630	radC; DNA repair protein RadC
AIN_01033	K03070	secA; preprotein translocase subunit SecA [EC:7.4.2.8]
AIN_01034	K03769	ppiC; peptidyl-prolyl cis-trans isomerase C [EC:5.2.1.8]

AIN_01035	K00620	argJ; glutamate N-acetyltransferase / amino-acid N-acetyltransferase [EC:2.3.1.35 2.3.1.1]
AIN_01036	K03574	mutT, NUDT15, MTH2; 8-oxo-dGTP diphosphatase [EC:3.6.1.55]
AIN_01038	K02519	infB, MTIF2; translation initiation factor IF-2
AIN_01039	K07742	ylxR; uncharacterized protein
AIN_01040	K02600	nusA; transcription termination/antitermination protein NusA
AIN_01041	K09748	rimP; ribosome maturation factor RimP
AIN_01043	K01259	pip; proline iminopeptidase [EC:3.4.11.5]
AIN_01044	K00568	ubiG; 2-polyprenyl-6-hydroxyphenyl methylase / 3-demethylubiquinone-9 3-methyltransferase [EC:2.1.1.222 2.1.1.64]
AIN_01046	K11206	NIT1, ybeM; deaminated glutathione amidase [EC:3.5.1.128]
AIN_01047	K03676	grxC, GLRX, GLRX2; glutaredoxin 3
AIN_01050	K01772	hemH, FECH; protoporphyrin/coproporphyrin ferrochelatase [EC:4.98.1.1 4.99.1.9]
AIN_01055	K03215	rumA; 23S rRNA (uracil1939-C5)-methyltransferase [EC:2.1.1.190]
AIN_01056	K06147	ABCB-BAC; ATP-binding cassette, subfamily B, bacterial
AIN_01057	K18893	vcaM; ATP-binding cassette, subfamily B, multidrug efflux pump
AIN_01058	K00970	pcnB; poly(A) polymerase [EC:2.7.7.19]
AIN_01060	K04083	hslO; molecular chaperone Hsp33
AIN_01061	K03574	mutT, NUDT15, MTH2; 8-oxo-dGTP diphosphatase [EC:3.6.1.55]
AIN_01062	K07315	rsbU_P; phosphoserine phosphatase RsbU/P [EC:3.1.3.3]
AIN_01063	K01754	E4.3.1.19, ilvA, tdcB; threonine dehydratase [EC:4.3.1.19]
AIN_01065	K01940	argG, ASS1; argininosuccinate synthase [EC:6.3.4.5]
AIN_01066	K07304	msrA; peptide-methionine (S)-S-oxide reductase [EC:1.8.4.11]
AIN_01067	K00852	rbsK, RBKS; ribokinase [EC:2.7.1.15]
AIN_01068	K03555	mutS; DNA mismatch repair protein MutS
AIN_01069	K18904	nodT, ameC; outer membrane protein, multidrug efflux system
AIN_01070	K03328	TC.PST; polysaccharide transporter, PST family
AIN_01075	K16554	exoP, vpsO; polysaccharide biosynthesis transport protein [EC:2.7.10.3]
AIN_01076	K16567	exoQ; exopolysaccharide production protein ExoQ
AIN_01086	K00111	glpA, glpD; glycerol-3-phosphate dehydrogenase [EC:1.1.5.3]
AIN_01096	K16558	exoL; succinoglycan biosynthesis protein ExoL [EC:2.-.-.-]
AIN_01097	K16557	exoA; succinoglycan biosynthesis protein ExoA [EC:2.4.-.-]
AIN_01099	K01991	wza, gfcE; polysaccharide biosynthesis/export protein
AIN_01101	K16555	exoO; succinoglycan biosynthesis protein ExoO [EC:2.4.-.-]
AIN_01102	K16564	exoU; succinoglycan biosynthesis protein ExoU [EC:2.4.-.-]
AIN_01104	K16566	exoY; exopolysaccharide production protein ExoY
AIN_01106	K16556	exoM; succinoglycan biosynthesis protein ExoM [EC:2.4.-.-]
AIN_01108	K03687	GRPE; molecular chaperone GrpE
AIN_01109	K03705	hrcA; heat-inducible transcriptional repressor
AIN_01110	K00989	rph; ribonuclease PH [EC:2.7.7.56]
AIN_01111	K01519	rdgB, ITPA; XTP/dITP diphosphohydrolase [EC:3.6.1.66]
AIN_01113	K03497	parB, spo0J; ParB family transcriptional regulator, chromosome partitioning protein
AIN_01114	K03496	parA, soj; chromosome partitioning protein
AIN_01116	K03495	gidA, mnmG, MTO1; tRNA uridine 5-carboxymethylaminomethyl modification enzyme

AIN_01117	K03650	mnmE, trmE, MSS1; tRNA modification GTPase [EC:3.6.-.-]
AIN_01118	K03628	rho; transcription termination factor Rho
AIN_01119	K08973	hemJ; protoporphyrinogen IX oxidase [EC:1.3.99.-]
AIN_01120	K06287	yhdE; nucleoside triphosphate pyrophosphatase [EC:3.6.1.-]
AIN_01121	K00014	aroE; shikimate dehydrogenase [EC:1.1.1.25]
AIN_01122	K00859	coaE; dephospho-CoA kinase [EC:2.7.1.24]
AIN_01123	K02342	dnaQ; DNA polymerase III subunit epsilon [EC:2.7.7.7]
AIN_01124	K03071	secB; preprotein translocase subunit SecB
AIN_01125	K07113	fxsA; UPF0716 protein FxsA
AIN_01127	K08304	mltA; peptidoglycan lytic transglycosylase A [EC:4.2.2.29]
AIN_01129	K06049	bchO; magnesium chelatase accessory protein
AIN_01130	K03404	chlD, bchD; magnesium chelatase subunit D [EC:6.6.1.1]
AIN_01131	K03405	chlI, bchI; magnesium chelatase subunit I [EC:6.6.1.1]
AIN_01132	K09847	crtA; spheroidene monooxygenase [EC:1.14.15.9]
AIN_01133	K10027	crtI; phytoene desaturase [EC:1.3.99.26 1.3.99.28 1.3.99.29 1.3.99.31]
AIN_01134	K02291	crtB; 15-cis-phytoene synthase [EC:2.5.1.32]
AIN_01135	K05770	TSPO, BZRP; translocator protein
AIN_01137	K09844	crtC; carotenoid 1,2-hydratase [EC:4.2.1.131]
AIN_01138	K09845	crtD; 1-hydroxycarotenoid 3,4-desaturase [EC:1.3.99.27]
AIN_01139	K13789	GGPS; geranylgeranyl diphosphate synthase, type II [EC:2.5.1.1 2.5.1.10 2.5.1.29]
AIN_01140	K09846	crtF; demethylspheroidene O-methyltransferase [EC:2.1.1.210]
AIN_01141	K11337	bchC; bacteriochlorophyllide a dehydrogenase [EC:1.1.1.396]
AIN_01142	K11333	bchX; 3,8-divinyl chlorophyllide a/chlorophyllide a reductase subunit X [EC:1.3.7.14 1.3.7.15]
AIN_01144	K11334	bchY; 3,8-divinyl chlorophyllide a/chlorophyllide a reductase subunit Y [EC:1.3.7.14 1.3.7.15]
AIN_01145	K11335	bchZ; 3,8-divinyl chlorophyllide a/chlorophyllide a reductase subunit Z [EC:1.3.7.14 1.3.7.15]
AIN_01147	K08927	pufB; light-harvesting complex 1 beta chain
AIN_01148	K08926	pufA; light-harvesting complex 1 alpha chain
AIN_01149	K08928	pufL; photosynthetic reaction center L subunit
AIN_01150	K08929	pufM; photosynthetic reaction center M subunit
AIN_01156	K03470	rnhB; ribonuclease HII [EC:3.1.26.4]
AIN_01157	K13581	ccrM; modification methylase [EC:2.1.1.72]
AIN_01160	K02419	fliP; flagellar biosynthesis protein FliP
AIN_01161	K02417	fliN; flagellar motor switch protein FliN
AIN_01163	K02409	fliF; flagellar M-ring protein FliF
AIN_01164	K02415	fliL; flagellar protein FliL
AIN_01167	K02556	motA; chemotaxis protein MotA
AIN_01170	K02400	flhA; flagellar biosynthesis protein FlhA
AIN_01171	K02421	fliR; flagellar biosynthesis protein FliR
AIN_01172	K02401	flhB; flagellar biosynthesis protein FlhB
AIN_01174	K02415	fliL; flagellar protein FliL
AIN_01175	K02393	flgH; flagellar L-ring protein FlgH
AIN_01176	K02386	flgA; flagellar basal body P-ring formation protein FlgA

AIN_01177	K02392	flgG; flagellar basal-body rod protein FlgG
AIN_01178	K02391	flgF; flagellar basal-body rod protein FlgF
AIN_01179	K02420	fliQ; flagellar biosynthesis protein FliQ
AIN_01180	K02408	fliE; flagellar hook-basal body complex protein FliE
AIN_01181	K02388	flgC; flagellar basal-body rod protein FlgC
AIN_01182	K02387	flgB; flagellar basal-body rod protein FlgB
AIN_01183	K02412	fliI; flagellum-specific ATP synthase [EC:7.4.2.8]
AIN_01184	K02035	ABC.PE.S; peptide/nickel transport system substrate-binding protein
AIN_01185	K02557	motB; chemotaxis protein MotB
AIN_01186	K02390	flgE; flagellar hook protein FlgE
AIN_01187	K02396	flgK; flagellar hook-associated protein 1
AIN_01188	K02397	flgL; flagellar hook-associated protein 3 FlgL
AIN_01189	K02394	flgI; flagellar P-ring protein FlgI
AIN_01190	K03498	trkH, trkG, ktrB, ktrD; trk/ktr system potassium uptake protein
AIN_01191	K03499	trkA, ktrA, ktrC; trk/ktr system potassium uptake protein
AIN_01192	K02035	ABC.PE.S; peptide/nickel transport system substrate-binding protein
AIN_01195	K02033	ABC.PE.P; peptide/nickel transport system permease protein
AIN_01196	K02034	ABC.PE.P1; peptide/nickel transport system permease protein
AIN_01197	K02031	ddpD; peptide/nickel transport system ATP-binding protein
AIN_01198	K02032	ddpF; peptide/nickel transport system ATP-binding protein
AIN_01199	K03808	pqiA; paraquat-inducible protein A
AIN_01200	K03808	pqiA; paraquat-inducible protein A
AIN_01201	K06192	pqiB; paraquat-inducible protein B
AIN_01202	K09857	K09857; uncharacterized protein
AIN_01205	K07793	tctA; putative tricarboxylic transport membrane protein
AIN_01209	K13039	comE; sulfopyruvate decarboxylase subunit beta [EC:4.1.1.79]
AIN_01210	K06034	comD; sulfopyruvate decarboxylase subunit alpha [EC:4.1.1.79]
AIN_01212	K15509	hpsN; sulfopropanediol 3-dehydrogenase [EC:1.1.1.308]
AIN_01215	K07793	tctA; putative tricarboxylic transport membrane protein
AIN_01216	K07794	tctB; putative tricarboxylic transport membrane protein
AIN_01217	K07795	tctC; putative tricarboxylic transport membrane protein
AIN_01218	K06145	gntR; LacI family transcriptional regulator, gluconate utilization system Gnt-I transcriptional repressor
AIN_01220	K23256	curA; NADPH-dependent curcumin reductase [EC:1.3.1.-]
AIN_01221	K00496	alkB1_2, alkM; alkane 1-monooxygenase [EC:1.14.15.3]
AIN_01222	K03575	mutY; A/G-specific adenine glycosylase [EC:3.2.2.31]
AIN_01225	K00912	lpxK; tetraacyldisaccharide 4'-kinase [EC:2.7.1.130]
AIN_01226	K02527	kdtA, waaA; 3-deoxy-D-manno-octulosonic-acid transferase [EC:2.4.99.12 2.4.99.13 2.4.99.14 2.4.99.15]
AIN_01229	K01092	E3.1.3.25, IMPA, suhB; myo-inositol-1(or 4)-monophosphatase [EC:3.1.3.25]
AIN_01230	K03592	pmbA; PmbA protein
AIN_01231	K03654	recQ; ATP-dependent DNA helicase RecQ [EC:5.6.2.4]
AIN_01233	K02221	yggT; YggT family protein
AIN_01235	K06902	UMF1; MFS transporter, UMF1 family

AIN_01236	K07261	mepA; penicillin-insensitive murein DD-endopeptidase [EC:3.4.24.-]
AIN_01238	K09125	yhhQ; queuosine precursor transporter
AIN_01240	K15034	yaeJ; ribosome-associated protein
AIN_01244	K00763	pncB, NAPRT1; nicotinate phosphoribosyltransferase [EC:6.3.4.21]
AIN_01247	K00943	tmk, DTYMK; dTMP kinase [EC:2.7.4.9]
AIN_01249	K01885	EARS, gltX; glutamyl-tRNA synthetase [EC:6.1.1.17]
AIN_01251	K02004	ABC.CD.P; putative ABC transport system permease protein
AIN_01252	K02003	ABC.CD.A; putative ABC transport system ATP-binding protein
AIN_01253	K10804	tesA; acyl-CoA thioesterase I [EC:3.1.2.- 3.1.2.2 3.1.1.2 3.1.1.5]
AIN_01256	K03704	cspA; cold shock protein
AIN_01257	K21014	stf0; trehalose 2-sulfotransferase [EC:2.8.2.37]
AIN_01259	K03704	cspA; cold shock protein
AIN_01264	K12410	cobB, srtN, npdA; NAD-dependent protein deacetylase/lipoamidase [EC:2.3.1.286 2.3.1.313]
AIN_01265	K02902	RP-L28, MRPL28, rpmB; large subunit ribosomal protein L28
AIN_01266	K07588	MMAA, argK; GTPase [EC:3.6.5.-]
AIN_01267	K03579	hrpB; ATP-dependent RNA helicase HrpB [EC:5.6.2.6]
AIN_01268	K00033	PGD, gnd, gntZ; 6-phosphogluconate dehydrogenase [EC:1.1.1.44 1.1.1.343]
AIN_01269	K00611	OTC, argF, argI; ornithine carbamoyltransferase [EC:2.1.3.3]
AIN_01270	K00821	argD; acetylornithine/N-succinyldiaminopimelate aminotransferase [EC:2.6.1.11 2.6.1.17]
AIN_01272	K00432	gpx, btuE, bsaA; glutathione peroxidase [EC:1.11.1.9]
AIN_01273	K01992	ABC-2.P; ABC-2 type transport system permease protein
AIN_01274	K13583	gcrA; GcrA cell cycle regulator
AIN_01275	K02275	coxB, ctaC; cytochrome c oxidase subunit II [EC:7.1.1.9]
AIN_01276	K02257	COX10, ctaB, cyoE; heme o synthase [EC:2.5.1.141]
AIN_01278	K02258	COX11, ctaG; cytochrome c oxidase assembly protein subunit 11
AIN_01279	K02276	coxC, ctaE; cytochrome c oxidase subunit III [EC:7.1.1.9]
AIN_01280	K14998	SURF1, SHY1; surfeit locus 1 family protein
AIN_01281	K01733	thrC; threonine synthase [EC:4.2.3.1]
AIN_01283	K03790	rimJ; [ribosomal protein S5]-alanine N-acetyltransferase [EC:2.3.1.267]
AIN_01287	K00759	APRT, apt; adenine phosphoribosyltransferase [EC:2.4.2.7]
AIN_01288	K05834	rhtB; homoserine/homoserine lactone efflux protein
AIN_01289	K00772	mtaP, MTAP; 5'-methylthioadenosine phosphorylase [EC:2.4.2.28]
AIN_01290	K03321	TC.SULP; sulfate permease, SulP family
AIN_01291	K18234	vat; virginiamycin A acetyltransferase [EC:2.3.1.-]
AIN_01298	K00114	exaA; alcohol dehydrogenase (cytochrome c) [EC:1.1.2.8]
AIN_01299	K02051	ABC.SN.S; NitT/TauT family transport system substrate-binding protein
AIN_01300	K02049	ABC.SN.A; NitT/TauT family transport system ATP-binding protein
AIN_01302	K01992	ABC-2.P; ABC-2 type transport system permease protein
AIN_01303	K01990	ABC-2.A; ABC-2 type transport system ATP-binding protein
AIN_01310	K09386	K09386; uncharacterized protein
AIN_01311	K03518	coxS; aerobic carbon-monoxide dehydrogenase small subunit [EC:1.2.5.3]
AIN_01312	K03520	coxL, cutL; aerobic carbon-monoxide dehydrogenase large subunit [EC:1.2.5.3]

AIN_01313	K03519	coxM, cutM; aerobic carbon-monoxide dehydrogenase medium subunit [EC:1.2.5.3]
AIN_01314	K03734	apbE; FAD:protein FMN transferase [EC:2.7.1.180]
AIN_01315	K19339	nosR; NosR/NirI family transcriptional regulator, nitrous oxide reductase regulator
AIN_01316	K02050	ABC.SN.P; NitT/TauT family transport system permease protein
AIN_01317	K01011	TST, MPST, sseA; thiosulfate/3-mercaptopyruvate sulfurtransferase [EC:2.8.1.1 2.8.1.2]
AIN_01318	K00928	lysC; aspartate kinase [EC:2.7.2.4]
AIN_01322	K04768	acuC; acetoin utilization protein AcuC
AIN_01323	K02527	kdtA, waaA; 3-deoxy-D-manno-octulosonic-acid transferase [EC:2.4.99.12 2.4.99.13 2.4.99.14 2.4.99.15]
AIN_01324	K02020	modA; molybdate transport system substrate-binding protein
AIN_01325	K02018	modB; molybdate transport system permease protein
AIN_01326	K02017	modC; molybdate transport system ATP-binding protein [EC:7.3.2.5]
AIN_01330	K18302	mexJ; membrane fusion protein, multidrug efflux system
AIN_01332	K11904	vgrG; type VI secretion system secreted protein VgrG
AIN_01341	K07506	K07506; AraC family transcriptional regulator
AIN_01345	K06889	K06889; uncharacterized protein
AIN_01356	K23244	apsD; D-apiose dehydrogenase [EC:1.1.1.420]
AIN_01357	K00052	leuB, IMDH; 3-isopropylmalate dehydrogenase [EC:1.1.1.85]
AIN_01358	K07795	tctC; putative tricarboxylic transport membrane protein
AIN_01360	K07793	tctA; putative tricarboxylic transport membrane protein
AIN_01366	K10107	kpsE; capsular polysaccharide transport system permease protein
AIN_01367	K09689	kpsT; capsular polysaccharide transport system ATP-binding protein [EC:7.6.2.12]
AIN_01372	K12972	ghrA; glyoxylate/hydroxypyruvate reductase [EC:1.1.1.79 1.1.1.81]
AIN_01373	K05837	rodA, mrdB; rod shape determining protein RodA
AIN_01374	K05515	mrdA; penicillin-binding protein 2 [EC:3.4.16.4]
AIN_01375	K03571	mreD; rod shape-determining protein MreD
AIN_01376	K03570	mreC; rod shape-determining protein MreC
AIN_01377	K03569	mreB; rod shape-determining protein MreB and related proteins
AIN_01378	K01649	leuA, IMS; 2-isopropylmalate synthase [EC:2.3.3.13]
AIN_01380	K06876	phrB; (6-4)DNA photolyase [EC:4.1.99.13]
AIN_01382	K01916	nadE; NAD+ synthase [EC:6.3.1.5]
AIN_01388	K18911	egtD; L-histidine Nalpha-methyltransferase [EC:2.1.1.44]
AIN_01391	K11688	dctP; C4-dicarboxylate-binding protein DctP
AIN_01392	K02040	pstS; phosphate transport system substrate-binding protein
AIN_01395	K00812	aspB; aspartate aminotransferase [EC:2.6.1.1]
AIN_01397	K09159	cptB; antitoxin CptB
AIN_01400	K01759	GLO1, gloA; lactoylglutathione lyase [EC:4.4.1.5]
AIN_01401	K03465	thyX, thy1; thymidylate synthase (FAD) [EC:2.1.1.148]
AIN_01402	K03704	cspA; cold shock protein
AIN_01403	K15977	K15977; putative oxidoreductase
AIN_01405	K09930	K09930; uncharacterized protein
AIN_01407	K01868	TARS, thrS; threonyl-tRNA synthetase [EC:6.1.1.3]
AIN_01409	K03292	TC.GPH; glycoside/pentoside/hexuronide:cation symporter, GPH family

AIN_01410	K15509	hpsN; sulfopropanediol 3-dehydrogenase [EC:1.1.1.308]
AIN_01416	K00549	metE; 5-methyltetrahydropteroyltriglutamate--homocysteine methyltransferase [EC:2.1.1.14]
AIN_01419	K05973	phaZ; poly(3-hydroxybutyrate) depolymerase [EC:3.1.1.75]
AIN_01420	K03821	phaC, phbC; poly[(R)-3-hydroxyalkanoate] polymerase subunit PhaC [EC:2.3.1.304]
AIN_01426	K01915	glnA, GLUL; glutamine synthetase [EC:6.3.1.2]
AIN_01428	K02029	ABC.PA.P; polar amino acid transport system permease protein
AIN_01429	K02029	ABC.PA.P; polar amino acid transport system permease protein
AIN_01431	K02030	ABC.PA.S; polar amino acid transport system substrate-binding protein
AIN_01432	K02028	ABC.PA.A; polar amino acid transport system ATP-binding protein [EC:7.4.2.1]
AIN_01435	K07274	mipA, ompV; MipA family protein
AIN_01436	K06998	phzF; trans-2,3-dihydro-3-hydroxyanthranilate isomerase [EC:5.3.3.17]
AIN_01437	K01937	pyrG, CTPS; CTP synthase [EC:6.3.4.2]
AIN_01438	K03075	secG; preprotein translocase subunit SecG
AIN_01439	K01939	purA, ADSS; adenylosuccinate synthase [EC:6.3.4.4]
AIN_01442	K00949	thiN, TPK1, THI80; thiamine pyrophosphokinase [EC:2.7.6.2]
AIN_01443	K01807	rpiA; ribose 5-phosphate isomerase A [EC:5.3.1.6]
AIN_01444	K00383	GSR, gor; glutathione reductase (NADPH) [EC:1.8.1.7]
AIN_01445	K04088	hflK; modulator of FtsH protease HflK
AIN_01446	K04087	hflC; modulator of FtsH protease HflC
AIN_01447	K09937	K09937; uncharacterized protein
AIN_01448	K04771	degP, htrA; serine protease Do [EC:3.4.21.107]
AIN_01449	K04755	fdx; ferredoxin, 2Fe-2S
AIN_01451	K02233	E2.7.8.26, cobS, cobV; adenosylcobinamide-GDP ribazoletransferase [EC:2.7.8.26]
AIN_01452	K00768	E2.4.2.21, cobU, cobT; nicotinate-nucleotide--dimethylbenzimidazole phosphoribosyltransferase [EC:2.4.2.21]
AIN_01454	K00457	HPD, hppD; 4-hydroxyphenylpyruvate dioxygenase [EC:1.13.11.27]
AIN_01456	K01740	metY; O-acetylhomoserine (thiol)-lyase [EC:2.5.1.49]
AIN_01457	K06991	K06991; uncharacterized protein
AIN_01459	K22084	mgdA; methylglutamate dehydrogenase subunit A [EC:1.5.99.5]
AIN_01462	K22085	mgdB; methylglutamate dehydrogenase subunit B [EC:1.5.99.5]
AIN_01463	K22086	mgdC; methylglutamate dehydrogenase subunit C [EC:1.5.99.5]
AIN_01464	K22087	mgdD; methylglutamate dehydrogenase subunit D [EC:1.5.99.5]
AIN_01465	K02316	dnaG; DNA primase [EC:2.7.7.101]
AIN_01466	K03086	rpoD; RNA polymerase primary sigma factor
AIN_01469	K09165	K09165; dodecin
AIN_01471	K07738	nrdR; transcriptional repressor NrdR
AIN_01472	K11752	ribD; diaminohydroxyphosphoribosylaminopyrimidine deaminase / 5-amino-6-(5-phosphoribosylamino)uracil reductase [EC:3.5.4.26 1.1.1.193]
AIN_01473	K07266	kpsC, lipA; capsular polysaccharide export protein
AIN_01474	K01991	wza, gfcE; polysaccharide biosynthesis/export protein
AIN_01475	K07265	kpsS, lipB; capsular polysaccharide export protein
AIN_01476	K00793	ribE, RIB5; riboflavin synthase [EC:2.5.1.9]
AIN_01481	K03625	nusB; transcription antitermination protein NusB
AIN_01482	K00794	ribH, RIB4; 6,7-dimethyl-8-ribityllumazine synthase [EC:2.5.1.78]

AIN_01483	K14652	ribBA; 3,4-dihydroxy 2-butanone 4-phosphate synthase / GTP cyclohydrolase II [EC:4.1.99.12 3.5.4.25]
AIN_01488	K06180	rluD; 23S rRNA pseudouridine1911/1915/1917 synthase [EC:5.4.99.23]
AIN_01489	K03089	rpoH; RNA polymerase sigma-32 factor
AIN_01490	K07577	K07577; putative mRNA 3-end processing factor
AIN_01493	K08602	pepF, pepB; oligoendopeptidase F [EC:3.4.24.-]
AIN_01496	K01048	pldB; lysophospholipase [EC:3.1.1.5]
AIN_01499	K17675	SUPV3L1, SUV3; ATP-dependent RNA helicase SUPV3L1/SUV3 [EC:5.6.2.6]
AIN_01500	K04762	hslR; ribosome-associated heat shock protein Hsp15
AIN_01501	K05524	fdxA; ferredoxin
AIN_01502	K07736	carD; CarD family transcriptional regulator, regulator of rRNA transcription
AIN_01503	K01945	purD; phosphoribosylamine---glycine ligase [EC:6.3.4.13]
AIN_01504	K03601	xseA; exodeoxyribonuclease VII large subunit [EC:3.1.11.6]
AIN_01506	K22515	fdwB; formate dehydrogenase beta subunit [EC:1.17.1.9]
AIN_01507	K03110	ftsY; fused signal recognition particle receptor
AIN_01509	K06190	ispZ; intracellular septation protein
AIN_01511	K11209	yghU, yfcG; GSH-dependent disulfide-bond oxidoreductase [EC:1.8.4.-]
AIN_01513	K10764	metZ; O-succinylhomoserine sulfhydrylase [EC:2.5.1.-]
AIN_01514	K09007	folE2; GTP cyclohydrolase IB [EC:3.5.4.16]
AIN_01515	K08484	ptsP; phosphotransferase system, enzyme I, PtsP [EC:2.7.3.9]
AIN_01517	K18372	acmB; methyl acetate hydrolase [EC:3.1.1.114]
AIN_01519	K00567	ogt, MGMT; methylated-DNA-[protein]-cysteine S-methyltransferase [EC:2.1.1.63]
AIN_01520	K03588	ftsW, spoVE; cell division protein FtsW
AIN_01521	K02563	murG; UDP-N-acetylglucosamine--N-acetylmuramyl-(pentapeptide) pyrophosphoryl-undecaprenol N-acetylglucosamine transferase [EC:2.4.1.227]
AIN_01522	K01924	murC; UDP-N-acetylmuramate--alanine ligase [EC:6.3.2.8]
AIN_01525	K00075	murB; UDP-N-acetylmuramate dehydrogenase [EC:1.3.1.98]
AIN_01526	K01921	ddl; D-alanine-D-alanine ligase [EC:6.3.2.4]
AIN_01527	K03589	ftsQ; cell division protein FtsQ
AIN_01528	K03590	ftsA; cell division protein FtsA
AIN_01529	K03531	ftsZ; cell division protein FtsZ
AIN_01530	K02535	lpxC; UDP-3-O-[3-hydroxymyristoyl] N-acetylglucosamine deacetylase [EC:3.5.1.108]
AIN_01531	K05807	bamD; outer membrane protein assembly factor BamD
AIN_01532	K03631	recN; DNA repair protein RecN (Recombination protein N)
AIN_01533	K03281	clcA, clcB, CLC-E, CLC-F; chloride channel protein, CIC family
AIN_01535	K01262	pepP; Xaa-Pro aminopeptidase [EC:3.4.11.9]
AIN_01536	K09883	cobT; cobaltochelatase CobT [EC:6.6.1.2]
AIN_01540	K01256	pepN; aminopeptidase N [EC:3.4.11.2]
AIN_01542	K01638	aceB, glcB; malate synthase [EC:2.3.3.9]
AIN_01546	K07010	K07010; putative glutamine amidotransferase
AIN_01547	K09988	lyxA; D-lyxose ketol-isomerase [EC:5.3.1.15]
AIN_01548	K03830	yafP; putative acetyltransferase [EC:2.3.1.-]
AIN_01549	K01556	KYNU, kynU; kynureninase [EC:3.7.1.3]
AIN_01551	K00324	pntA; proton-translocating NAD(P)+ transhydrogenase subunit alpha [EC:7.1.1.1]

AIN_01552	K00325	pntB; proton-translocating NAD(P)+ transhydrogenase subunit beta [EC:7.1.1.1]
AIN_01553	K06181	rluE; 23S rRNA pseudouridine2457 synthase [EC:5.4.99.20]
AIN_01556	K00655	plsC; 1-acyl-sn-glycerol-3-phosphate acyltransferase [EC:2.3.1.51]
AIN_01557	K02034	ABC.PE.P1; peptide/nickel transport system permease protein
AIN_01558	K02033	ABC.PE.P; peptide/nickel transport system permease protein
AIN_01559	K02035	ABC.PE.S; peptide/nickel transport system substrate-binding protein
AIN_01560	K02032	ddpF; peptide/nickel transport system ATP-binding protein
AIN_01561	K15583	oppD; oligopeptide transport system ATP-binding protein
AIN_01562	K01438	argE; acetylornithine deacetylase [EC:3.5.1.16]
AIN_01566	K03639	moaA, CNX2; GTP 3',8-cyclase [EC:4.1.99.22]
AIN_01568	K00820	glmS, GFPT; glutamine---fructose-6-phosphate transaminase (isomerizing) [EC:2.6.1.16]
AIN_01569	K04042	glmU; bifunctional UDP-N-acetylglucosamine pyrophosphorylase / glucosamine-1-phosphate N-acetyltransferase [EC:2.7.7.23 2.3.1.157]
AIN_01570	K01091	gph; phosphoglycolate phosphatase [EC:3.1.3.18]
AIN_01573	K00253	IVD, ivd; isovaleryl-CoA dehydrogenase [EC:1.3.8.4]
AIN_01575	K01969	MCCC2, accD1; 3-methylcrotonyl-CoA carboxylase beta subunit [EC:6.4.1.4]
AIN_01578	K01968	MCCC1, accA1; 3-methylcrotonyl-CoA carboxylase alpha subunit [EC:6.4.1.4]
AIN_01579	K00799	GST, gst; glutathione S-transferase [EC:2.5.1.18]
AIN_01580	K01640	HMGCL, hmgL; hydroxymethylglutaryl-CoA lyase [EC:4.1.3.4]
AIN_01581	K13766	liuC; methylglutaconyl-CoA hydratase [EC:4.2.1.18]
AIN_01582	K00330	nuoA; NADH-quinone oxidoreductase subunit A [EC:7.1.1.2]
AIN_01585	K06904	GP4; Escherichia/Staphylococcus phage prohead protease
AIN_01599	K00640	cysE; serine O-acetyltransferase [EC:2.3.1.30]
AIN_01600	K00627	DLAT, aceF, pdhC; pyruvate dehydrogenase E2 component (dihydrolipoyllysine-residue acetyltransferase) [EC:2.3.1.12]
AIN_01602	K00162	PDHB, pdhB; pyruvate dehydrogenase E1 component subunit beta [EC:1.2.4.1]
AIN_01604	K00161	PDHA, pdhA; pyruvate dehydrogenase E1 component subunit alpha [EC:1.2.4.1]
AIN_01606	K11645	fbaB; fructose-bisphosphate aldolase, class I [EC:4.1.2.13]
AIN_01608	K00927	PGK, pgk; phosphoglycerate kinase [EC:2.7.2.3]
AIN_01610	K01802	E5.2.1.8; peptidylprolyl isomerase [EC:5.2.1.8]
AIN_01611	K01866	YARS, tyrS; tyrosyl-tRNA synthetase [EC:6.1.1.1]
AIN_01612	K09001	anmK; anhydro-N-acetylmuramic acid kinase [EC:2.7.1.170]
AIN_01614	K01653	E2.2.1.6S, ilvH, ilvN; acetolactate synthase I/III small subunit [EC:2.2.1.6]
AIN_01617	K01652	E2.2.1.6L, ilvB, ilvG, ilvI; acetolactate synthase I/II/III large subunit [EC:2.2.1.6]
AIN_01622	K21420	bpt; leucyl-tRNA---protein transferase [EC:2.3.2.29]
AIN_01625	K00982	glnE; [glutamine synthetase] adenylyltransferase / [glutamine synthetase]-adenylyl-L-tyrosine phosphorylase [EC:2.7.7.42 2.7.7.89]
AIN_01627	K01625	eda; 2-dehydro-3-deoxyphosphogluconate aldolase / (4S)-4-hydroxy-2-oxoglutarate aldolase [EC:4.1.2.14 4.1.3.42]
AIN_01628	K07006	K07006; uncharacterized protein
AIN_01630	K01690	edd; phosphogluconate dehydratase [EC:4.2.1.12]
AIN_01632	K00500	phhA, PAH; phenylalanine-4-hydroxylase [EC:1.14.16.1]
AIN_01639	K00382	DLD, lpd, pdhD; dihydrolipoyl dehydrogenase [EC:1.8.1.4]
AIN_01643	K03701	uvrA; excinuclease ABC subunit A
AIN_01645	K00020	HIBADH, mmsB; 3-hydroxyisobutyrate dehydrogenase [EC:1.1.1.31]

AIN_01649	K00140	mmsA, iolA, ALDH6A1; malonate-semialdehyde dehydrogenase (acetylating) / methylmalonate-semialdehyde dehydrogenase [EC:1.2.1.18 1.2.1.27]
AIN_01651	K00954	E2.7.7.3A, coaD, kdtB; pantetheine-phosphate adenylyltransferase [EC:2.7.7.3]
AIN_01655	K00134	GAPDH, gapA; glyceraldehyde 3-phosphate dehydrogenase (phosphorylating) [EC:1.2.1.12]
AIN_01657	K00615	E2.2.1.1, tktA, tktB; transketolase [EC:2.2.1.1]
AIN_01660	K07390	grxD, GLRX5; monothiol glutaredoxin
AIN_01665	K09882	cobS; cobaltochelatase CobS [EC:6.6.1.2]
AIN_01669	K05527	bolA; BolA family transcriptional regulator, general stress-responsive regulator
AIN_01672	K02434	gatB, PET112; aspartyl-tRNA(Asn)/glutamyl-tRNA(Gln) amidotransferase subunit B [EC:6.3.5.6 6.3.5.7]
AIN_01680	K05520	yhbO; deglycase [EC:3.5.1.124]
AIN_01682	K23269	purL; phosphoribosylformylglycinamidine synthase subunit PurL [EC:6.3.5.3]
AIN_01685	K04090	E1.2.7.8; indolepyruvate ferredoxin oxidoreductase [EC:1.2.7.8]
AIN_01687	K01776	murI; glutamate racemase [EC:5.1.1.3]
AIN_01688	K00145	argC; N-acetyl-gamma-glutamyl-phosphate reductase [EC:1.2.1.38]
AIN_01689	K02197	ccmE; cytochrome c-type biogenesis protein CcmE
AIN_01692	K02198	ccmF; cytochrome c-type biogenesis protein CcmF
AIN_01693	K02200	ccmH; cytochrome c-type biogenesis protein CcmH
AIN_01694	K15866	paaG; 2-(1,2-epoxy-1,2-dihydrophenyl)acetyl-CoA isomerase [EC:5.3.3.18]
AIN_01695	K01647	CS, gltA; citrate synthase [EC:2.3.3.1]
AIN_01696	K01885	EARS, gltX; glutamyl-tRNA synthetase [EC:6.1.1.17]
AIN_01697	K02238	comEC; competence protein ComEC
AIN_01698	K01356	lexA; repressor LexA [EC:3.4.21.88]
AIN_01699	K03750	moeA; molybdopterin molybdotransferase [EC:2.10.1.1]
AIN_01700	K03637	moaC, CNX3; cyclic pyranopterin monophosphate synthase [EC:4.6.1.17]
AIN_01701	K01609	trpC; indole-3-glycerol phosphate synthase [EC:4.1.1.48]
AIN_01702	K00766	trpD; anthranilate phosphoribosyltransferase [EC:2.4.2.18]
AIN_01703	K01658	trpG; anthranilate synthase component II [EC:4.1.3.27]
AIN_01706	K01657	trpE; anthranilate synthase component I [EC:4.1.3.27]
AIN_01707	K03770	ppiD; peptidyl-prolyl cis-trans isomerase D [EC:5.2.1.8]
AIN_01709	K00769	gpt; xanthine phosphoribosyltransferase [EC:2.4.2.22]
AIN_01710	K05835	rhtC; threonine efflux protein
AIN_01712	K00208	fabI; enoyl-[acyl-carrier protein] reductase I [EC:1.3.1.9 1.3.1.10]
AIN_01713	K00275	pdxH, PNPO; pyridoxamine 5'-phosphate oxidase [EC:1.4.3.5]
AIN_01714	K03704	cspA; cold shock protein
AIN_01715	K09005	K09005; uncharacterized protein
AIN_01719	K00525	E1.17.4.1A, nrdA, nrdE; ribonucleoside-diphosphate reductase alpha chain [EC:1.17.4.1]
AIN_01722	K21470	ycbB; L,D-transpeptidase YcbB
AIN_01723	K02536	lpxD; UDP-3-O-[3-hydroxymyristoyl] glucosamine N-acyltransferase [EC:2.3.1.191]
AIN_01724	K02078	acpP; acyl carrier protein
AIN_01725	K14660	nodE; nodulation protein E [EC:2.3.1.-]
AIN_01727	K06915	herA; DNA double-strand break repair helicase HerA and related ATPase
AIN_01728	K00681	ggt; gamma-glutamyltranspeptidase / glutathione hydrolase [EC:2.3.2.2 3.4.19.13]
AIN_01729	K03699	tlyC; magnesium and cobalt exporter, CNNM family

AIN_01730	K04763	xerD; integrase/recombinase XerD
AIN_01733	K00891	aroK, aroL; shikimate kinase [EC:2.7.1.71]
AIN_01734	K01735	aroB; 3-dehydroquinate synthase [EC:4.2.3.4]
AIN_01735	K03111	ssb; single-strand DNA-binding protein
AIN_01739	K02034	ABC.PE.P1; peptide/nickel transport system permease protein
AIN_01740	K02033	ABC.PE.P; peptide/nickel transport system permease protein
AIN_01741	K02035	ABC.PE.S; peptide/nickel transport system substrate-binding protein
AIN_01742	K00791	miaA, TRIT1; tRNA dimethylallyltransferase [EC:2.5.1.75]
AIN_01743	K09903	pyrH; uridylate kinase [EC:2.7.4.22]
AIN_01744	K02838	frr, MRRF, RRF; ribosome recycling factor
AIN_01745	K00806	uppS; undecaprenyl diphosphate synthase [EC:2.5.1.31]
AIN_01746	K00981	E2.7.7.41, CDS1, CDS2, cdsA; phosphatidate cytidylyltransferase [EC:2.7.7.41]
AIN_01747	K00099	dxr; 1-deoxy-D-xylulose-5-phosphate reductoisomerase [EC:1.1.1.267]
AIN_01748	K11749	rseP; regulator of sigma E protease [EC:3.4.24.-]
AIN_01750	K07277	SAM50, TOB55, bamA; outer membrane protein insertion porin family
AIN_01752	K02372	fabZ; 3-hydroxyacyl-[acyl-carrier-protein] dehydratase [EC:4.2.1.59]
AIN_01753	K00677	lpxA; UDP-N-acetylglucosamine acyltransferase [EC:2.3.1.129]
AIN_01754	K09949	lpxI; UDP-2,3-diacylglucosamine hydrolase [EC:3.6.1.54]
AIN_01755	K00748	lpxB; lipid-A-disaccharide synthase [EC:2.4.1.182]
AIN_01756	K00507	SCD, desC; stearoyl-CoA desaturase (Delta-9 desaturase) [EC:1.14.19.1]
AIN_01758	K00566	mnmA, trmU; tRNA-uridine 2-sulfurtransferase [EC:2.8.1.13]
AIN_01760	K13584	ctrA; two-component system, cell cycle response regulator CtrA
AIN_01761	K01972	E6.5.1.2, ligA, ligB; DNA ligase (NAD+) [EC:6.5.1.2]
AIN_01762	K03655	recG; ATP-dependent DNA helicase RecG [EC:5.6.2.4]
AIN_01765	K01496	hisI; phosphoribosyl-AMP cyclohydrolase [EC:3.5.4.19]
AIN_01766	K01894	gluQ; glutamyl-Q tRNA(Asp) synthetase [EC:6.1.1.-]
AIN_01768	K04094	trmFO, gid; methylenetetrahydrofolate--tRNA-(uracil-5-)-methyltransferase [EC:2.1.1.74]
AIN_01770	K02469	gyrA; DNA gyrase subunit A [EC:5.6.2.2]
AIN_01771	K01750	E4.3.1.12, ocd; ornithine cyclodeaminase [EC:4.3.1.12]
AIN_01772	K01476	E3.5.3.1, rocF, arg; arginase [EC:3.5.3.1]
AIN_01776	K07054	K07054; uncharacterized protein
AIN_01779	K09705	K09705; uncharacterized protein
AIN_01782	K01689	ENO1_2_3, eno; enolase 1/2/3 [EC:4.2.1.11]
AIN_01784	K03711	fur, zur, furB; Fur family transcriptional regulator, ferric uptake regulator
AIN_01785	K11927	rhlE; ATP-dependent RNA helicase RhlE [EC:5.6.2.7]
AIN_01786	K07058	K07058; membrane protein
AIN_01788	K08997	SELENOO, selO; protein adenylyltransferase [EC:2.7.7.108]
AIN_01791	K04765	mazG; nucleoside triphosphate diphosphatase [EC:3.6.1.9]
AIN_01792	K01480	speB; agmatinase [EC:3.5.3.11]
AIN_01795	K02835	prfA, MTRF1, MRF1; peptide chain release factor 1
AIN_01796	K02493	hemK, prmC, HEMK; release factor glutamine methyltransferase [EC:2.1.1.297]
AIN_01798	K02528	ksgA; 16S rRNA (adenine1518-N6/adenine1519-N6)-dimethyltransferase [EC:2.1.1.182]

AIN_01799	K00097	pdxA; 4-hydroxythreonine-4-phosphate dehydrogenase [EC:1.1.1.262]
AIN_01800	K03771	surA; peptidyl-prolyl cis-trans isomerase SurA [EC:5.2.1.8]
AIN_01801	K04744	lptD, imp, ostA; LPS-assembly protein
AIN_01802	K11720	lptG; lipopolysaccharide export system permease protein
AIN_01803	K07091	lptF; lipopolysaccharide export system permease protein
AIN_01804	K01255	CARP, pepA; leucyl aminopeptidase [EC:3.4.11.1]
AIN_01805	K02339	holC; DNA polymerase III subunit chi [EC:2.7.7.7]
AIN_01806	K06985	K06985; aspartyl protease family protein
AIN_01807	K05595	marC; multiple antibiotic resistance protein
AIN_01808	K06158	ABCF3; ATP-binding cassette, subfamily F, member 3
AIN_01809	K00940	ndk, NME; nucleoside-diphosphate kinase [EC:2.7.4.6]
AIN_01811	K07343	tfoX; DNA transformation protein and related proteins
AIN_01815	K07638	envZ; two-component system, OmpR family, osmolarity sensor histidine kinase EnvZ [EC:2.7.13.3]
AIN_01820	K01933	purM; phosphoribosylformylglycinamidine cyclo-ligase [EC:6.3.3.1]
AIN_01821	K11175	purN; phosphoribosylglycinamide formyltransferase 1 [EC:2.1.2.2]
AIN_01822	K03684	rnd; ribonuclease D [EC:3.1.13.5]
AIN_01825	K02426	sufE; cysteine desulfuration protein SufE
AIN_01831	K00548	metH, MTR; 5-methyltetrahydrofolate--homocysteine methyltransferase [EC:2.1.1.13]
AIN_01833	K01923	purC; phosphoribosylaminoimidazole-succinocarboxamide synthase [EC:6.3.2.6]
AIN_01834	K23264	purS; phosphoribosylformylglycinamidine synthase subunit PurS [EC:6.3.5.3]
AIN_01835	K00001	E1.1.1.1, adh; alcohol dehydrogenase [EC:1.1.1.1]
AIN_01836	K23265	purQ; phosphoribosylformylglycinamidine synthase subunit PurQ / glutaminase [EC:6.3.5.3 3.5.1.2]
AIN_01837	K10125	dctB; two-component system, NtrC family, C4-dicarboxylate transport sensor histidine kinase DctB [EC:2.7.13.3]
AIN_01840	K08300	rne; ribonuclease E [EC:3.1.26.12]
AIN_01841	K04085	tusA, sirA; tRNA 2-thiouridine synthesizing protein A [EC:2.8.1.-]
AIN_01842	K06196	ccdA; cytochrome c-type biogenesis protein
AIN_01847	K01679	E4.2.1.2B, fumC, FH; fumarate hydratase, class II [EC:4.2.1.2]
AIN_01848	K09985	K09985; uncharacterized protein
AIN_01849	K07240	chrA; chromate transporter
AIN_01856	K17486	dmdA; dimethylsulfoniopropionate demethylase [EC:2.1.1.269]
AIN_01857	K19745	acuI; acrylyl-CoA reductase (NADPH) [EC:1.3.1.-]
AIN_01858	K03451	TC.BCT; betaine/carnitine transporter, BCCT family
AIN_01859	K01669	phr, PHR1; deoxyribodipyrimidine photo-lyase [EC:4.1.99.3]
AIN_01860	K00574	cfa; cyclopropane-fatty-acyl-phospholipid synthase [EC:2.1.1.79]
AIN_01863	K01515	nudF; ADP-ribose diphosphatase [EC:3.6.1.13 3.6.1.-]
AIN_01864	K01738	cysK; cysteine synthase [EC:2.5.1.47]
AIN_01865	K05802	mscK, kefA, aefA; potassium-dependent mechanosensitive channel
AIN_01869	K00088	IMPDH, guaB; IMP dehydrogenase [EC:1.1.1.205]
AIN_01870	K03500	rsmB, sun; 16S rRNA (cytosine967-C5)-methyltransferase [EC:2.1.1.176]
AIN_01871	K13587	cckA; two-component system, cell cycle sensor histidine kinase and response regulator CckA [EC:2.7.13.3]
AIN_01873	K03553	recA; recombination protein RecA
AIN_01874	K03453	TC.BASS; bile acid:Na+ symporter, BASS family

AIN_01875	K01872	AARS, alaS; alanyl-tRNA synthetase [EC:6.1.1.7]
AIN_01878	K06207	typA, bipA; GTP-binding protein
AIN_01881	K03545	tig; trigger factor
AIN_01882	K02939	RP-L9, MRPL9, rplI; large subunit ribosomal protein L9
AIN_01883	K02963	RP-S18, MRPS18, rpsR; small subunit ribosomal protein S18
AIN_01884	K02990	RP-S6, MRPS6, rpsF; small subunit ribosomal protein S6
AIN_01889	K09816	znuB; zinc transport system permease protein
AIN_01890	K09817	znuC; zinc transport system ATP-binding protein [EC:7.2.2.20]
AIN_01891	K09815	znuA; zinc transport system substrate-binding protein
AIN_01893	K00031	IDH1, IDH2, icd; isocitrate dehydrogenase [EC:1.1.1.42]
AIN_01894	K03809	wrbA; NAD(P)H dehydrogenase (quinone) [EC:1.6.5.2]
AIN_01896	K22479	argA; N-acetyltransferase
AIN_01897	K07018	K07018; uncharacterized protein
AIN_01898	K13643	iscR; Rrf2 family transcriptional regulator, iron-sulfur cluster assembly transcription factor
AIN_01899	K04487	iscS, NFS1; cysteine desulfurase [EC:2.8.1.7]
AIN_01900	K09014	sufB; Fe-S cluster assembly protein SufB
AIN_01904	K09013	sufC; Fe-S cluster assembly ATP-binding protein
AIN_01905	K09015	sufD; Fe-S cluster assembly protein SufD
AIN_01908	K11717	sufS; cysteine desulfurase / selenocysteine lyase [EC:2.8.1.7 4.4.1.16]
AIN_01910	K09950	K09950; uncharacterized protein
AIN_01911	K02004	ABC.CD.P; putative ABC transport system permease protein
AIN_01912	K02003	ABC.CD.A; putative ABC transport system ATP-binding protein
AIN_01914	K02302	cysG; uroporphyrin-III C-methyltransferase / precorrin-2 dehydrogenase / sirohydrochlorin ferrochelatase [EC:2.1.1.107 1.3.1.76 4.99.1.4]
AIN_01915	K00372	nasC, nasA; assimilatory nitrate reductase catalytic subunit [EC:1.7.99.-]
AIN_01916	K26138	nasE; nitrite reductase [NAD(P)H] small subunit [EC:1.7.1.4]
AIN_01917	K26139	nasD, nasB; nitrite reductase [NAD(P)H] large subunit [EC:1.7.1.4]
AIN_01920	K15577	nrtB, nasE, cynB; nitrate/nitrite transport system permease protein
AIN_01921	K15576	nrtA, nasF, cynA; nitrate/nitrite transport system substrate-binding protein
AIN_01922	K22067	nasS; two-component system, oxyanion-binding sensor
AIN_01923	K07183	nasT; two-component system, response regulator / RNA-binding antiterminator
AIN_01924	K05367	pbpC; penicillin-binding protein 1C [EC:2.4.99.28]
AIN_01925	K06894	yfhM; alpha-2-macroglobulin
AIN_01926	K01951	guaA, GMPS; GMP synthase (glutamine-hydrolysing) [EC:6.3.5.2]
AIN_01927	K14083	mttB; trimethylamine---corrinoid protein Co-methyltransferase [EC:2.1.1.250]
AIN_01930	K03781	katE, CAT, catB, srpA; catalase [EC:1.11.1.6]
AIN_01931	K04761	oxyR; LysR family transcriptional regulator, hydrogen peroxide-inducible genes activator
AIN_01932	K03644	lipA, LIAS, LIP1, LIP5; lipoyl synthase [EC:2.8.1.8]
AIN_01934	K00760	hprT, hpt, HPRT1; hypoxanthine phosphoribosyltransferase [EC:2.4.2.8]
AIN_01935	K18588	COQ10; coenzyme Q-binding protein COQ10
AIN_01937	K03743	pncC; nicotinamide-nucleotide amidase [EC:3.5.1.42]
AIN_01938	K01095	pgpA; phosphatidylglycerophosphatase A [EC:3.1.3.27]
AIN_01939	K12506	ispDF; 2-C-methyl-D-erythritol 4-phosphate cytidylyltransferase / 2-C-methyl-D-erythritol 2,4-cyclodiphosphate synthase [EC:2.7.7.60 4.6.1.12]

AIN_01940	K05540	dusB; tRNA-dihydrouridine synthase B [EC:1.-.-.-]
AIN_01941	K07708	glnL, ntrB; two-component system, NtrC family, nitrogen regulation sensor histidine kinase GlnL [EC:2.7.13.3]
AIN_01942	K07712	glnG, ntrC; two-component system, NtrC family, nitrogen regulation response regulator GlnG
AIN_01943	K13598	ntrY; two-component system, NtrC family, nitrogen regulation sensor histidine kinase NtrY [EC:2.7.13.3]
AIN_01944	K13599	ntrX; two-component system, NtrC family, nitrogen regulation response regulator NtrX
AIN_01946	K03499	trkA, ktrA, ktrC; trk/ktr system potassium uptake protein
AIN_01947	K03498	trkH, trkG, ktrB, ktrD; trk/ktr system potassium uptake protein
AIN_01948	K03666	hfq; host factor-I protein
AIN_01949	K03665	hflX; GTPase
AIN_01951	K01434	pac; penicillin G amidase [EC:3.5.1.11]
AIN_01952	K00020	HIBADH, mmsB; 3-hydroxyisobutyrate dehydrogenase [EC:1.1.1.31]
AIN_01953	K01998	livM; branched-chain amino acid transport system permease protein
AIN_01954	K01997	livH; branched-chain amino acid transport system permease protein
AIN_01956	K01996	livF; branched-chain amino acid transport system ATP-binding protein
AIN_01957	K01995	livG; branched-chain amino acid transport system ATP-binding protein
AIN_01958	K01999	livK; branched-chain amino acid transport system substrate-binding protein
AIN_01959	K21826	gbdR; AraC family transcriptional regulator, glycine betaine-responsive activator
AIN_01960	K01626	E2.5.1.54, aroF, aroG, aroH; 3-deoxy-7-phosphoheptulonate synthase [EC:2.5.1.54]
AIN_01963	K00942	gmk, GUK1; guanylate kinase [EC:2.7.4.8]
AIN_01965	K01739	metB; cystathionine gamma-synthase [EC:2.5.1.48]
AIN_01967	K07636	phoR; two-component system, OmpR family, phosphate regulon sensor histidine kinase PhoR [EC:2.7.13.3]
AIN_01968	K02040	pstS; phosphate transport system substrate-binding protein
AIN_01969	K02037	pstC; phosphate transport system permease protein
AIN_01970	K02038	pstA; phosphate transport system permease protein
AIN_01971	K02036	pstB; phosphate transport system ATP-binding protein [EC:7.3.2.1]
AIN_01972	K02039	phoU; phosphate transport system protein
AIN_01973	K07657	phoB; two-component system, OmpR family, phosphate regulon response regulator PhoB
AIN_01974	K24662	rgtD; lipid A galacturonosyltransferase RgtD [EC:2.4.1.-]
AIN_01977	K00925	ackA; acetate kinase [EC:2.7.2.1]
AIN_01978	K00634	ptb; phosphate butyryltransferase [EC:2.3.1.19]
AIN_01980	K03786	aroQ, qutE; 3-dehydroquinate dehydratase II [EC:4.2.1.10]
AIN_01982	K03719	lrp; Lrp/AsnC family transcriptional regulator, leucine-responsive regulatory protein
AIN_01983	K13821	putA; RHH-type transcriptional regulator, proline utilization regulon repressor / proline dehydrogenase / delta 1-pyrroline-5-carboxylate dehydrogenase [EC:1.5.5.2 1.2.1.88]
AIN_01987	K09927	K09927; uncharacterized protein
AIN_01993	K09118	K09118; uncharacterized protein
AIN_01999	K06162	phnM; alpha-D-ribose 1-methylphosphonate 5-triphosphate diphosphatase [EC:3.6.1.63]
AIN_02001	K05774	phnN; ribose 1,5-bisphosphokinase [EC:2.7.4.23]
AIN_02002	K02043	phnF; GntR family transcriptional regulator, phosphonate transport system regulatory protein
AIN_02005	K06076	fadL; long-chain fatty acid transport protein
AIN_02009	K00651	metA; homoserine O-succinyltransferase/O-acetyltransferase [EC:2.3.1.46 2.3.1.31]
AIN_02011	K22468	ppk2; polyphosphate kinase [EC:2.7.4.34]
AIN_02015	K02996	RP-S9, MRPS9, rpsI; small subunit ribosomal protein S9

AIN_02016	K02871	RP-L13, MRPL13, rplM; large subunit ribosomal protein L13
AIN_02019	K01299	E3.4.17.19; carboxypeptidase Taq [EC:3.4.17.19]
AIN_02020	K02259	COX15, ctaA; heme a synthase [EC:1.17.99.9]
AIN_02021	K02533	lasT; tRNA/rRNA methyltransferase [EC:2.1.1.-]
AIN_02022	K00788	thiE; thiamine-phosphate pyrophosphorylase [EC:2.5.1.3]
AIN_02023	K22617	olsA; lyso-ornithine lipid O-acyltransferase [EC:2.3.1.270]
AIN_02026	K13588	chpT; histidine phosphotransferase ChpT
AIN_02027	K00147	proA; glutamate-5-semialdehyde dehydrogenase [EC:1.2.1.41]
AIN_02028	K00931	proB; glutamate 5-kinase [EC:2.7.2.11]
AIN_02029	K03979	obgE, cgtA, MTG2; GTPase [EC:3.6.5.-]
AIN_02033	K14415	RTCB, rtcB; tRNA-splicing ligase RtcB (3'-phosphate/5'-hydroxy nucleic acid ligase) [EC:6.5.1.8]
AIN_02034	K02899	RP-L27, MRPL27, rpmA; large subunit ribosomal protein L27
AIN_02035	K02888	RP-L21, MRPL21, rplU; large subunit ribosomal protein L21
AIN_02040	K02026	ABC.MS.P1; multiple sugar transport system permease protein
AIN_02041	K02025	ABC.MS.P; multiple sugar transport system permease protein
AIN_02042	K02027	ABC.MS.S; multiple sugar transport system substrate-binding protein
AIN_02043	K10112	msmX, msmK, malK, sugC, ggtA, msiK; multiple sugar transport system ATP-binding protein [EC:7.5.2.-]
AIN_02048	K22935	XK1, psk; D-ribulokinase [EC:2.7.1.47]
AIN_02050	K00864	glpK, GK; glycerol kinase [EC:2.7.1.30]
AIN_02051	K02427	rlmE, rrmJ, ftsJ; 23S rRNA (uridine2552-2'-O)-methyltransferase [EC:2.1.1.166]
AIN_02056	K01524	ppx-gppA; exopolyphosphatase / guanosine-5'-triphosphate,3'-diphosphate pyrophosphatase [EC:3.6.1.11 3.6.1.40]
AIN_02058	K00297	metF, MTHFR; methylenetetrahydrofolate reductase (NADH) [EC:1.5.1.54]
AIN_02059	K00548	metH, MTR; 5-methyltetrahydrofolate--homocysteine methyltransferase [EC:2.1.1.13]
AIN_02061	K02510	hpaI, hpcH; 4-hydroxy-2-oxoheptanedioate aldolase [EC:4.1.2.52]
AIN_02062	K10235	aglK; alpha-glucoside transport system ATP-binding protein
AIN_02063	K01187	malZ; alpha-glucosidase [EC:3.2.1.20]
AIN_02064	K10234	aglG, ggtD; alpha-glucoside transport system permease protein
AIN_02065	K10233	aglF, ggtC; alpha-glucoside transport system permease protein
AIN_02066	K10232	aglE, ggtB; alpha-glucoside transport system substrate-binding protein
AIN_02067	K05350	bglB; beta-glucosidase [EC:3.2.1.21]
AIN_02068	K00845	glk; glucokinase [EC:2.7.1.2]
AIN_02069	K00111	glpA, glpD; glycerol-3-phosphate dehydrogenase [EC:1.1.5.3]
AIN_02073	K07727	K07727; putative transcriptional regulator
AIN_02074	K02357	tsf, TSFM; elongation factor Ts
AIN_02075	K02967	RP-S2, MRPS2, rpsB; small subunit ribosomal protein S2
AIN_02078	K02030	ABC.PA.S; polar amino acid transport system substrate-binding protein
AIN_02082	K16329	psuG; pseudouridylate synthase [EC:4.2.1.70]
AIN_02084	K06895	lysE, argO; L-lysine exporter family protein LysE/ArgO
AIN_02085	K16348	ecnB; entericidin B
AIN_02086	K07001	K07001; NTE family protein
AIN_02088	K00019	BDH1, bdhA; 3-hydroxybutyrate dehydrogenase [EC:1.1.1.30]
AIN_02092	K02035	ABC.PE.S; peptide/nickel transport system substrate-binding protein

AIN_02096	K03741	arsC; arsenate reductase (thioredoxin) [EC:1.20.4.4]
AIN_02103	K24160	NHA1, SOD2; sodium/hydrogen antiporter
AIN_02104	K03723	mfd; transcription-repair coupling factor (superfamily II helicase) [EC:5.6.2.4]
AIN_02106	K01698	hemB, ALAD; porphobilinogen synthase [EC:4.2.1.24]
AIN_02108	K04719	bluB; 5,6-dimethylbenzimidazole synthase [EC:1.13.11.79]
AIN_02111	K00315	DMGDH; dimethylglycine dehydrogenase [EC:1.5.8.4]
AIN_02114	K01119	cpdB; 2',3'-cyclic-nucleotide 2'-phosphodiesterase / 3'-nucleotidase [EC:3.1.4.16 3.1.3.6]
AIN_02115	K02044	phnD; phosphonate transport system substrate-binding protein
AIN_02116	K02041	phnC; phosphonate transport system ATP-binding protein [EC:7.3.2.2]
AIN_02117	K02042	phnE; phosphonate transport system permease protein
AIN_02118	K02042	phnE; phosphonate transport system permease protein
AIN_02120	K07107	ybgC; acyl-CoA thioester hydrolase [EC:3.1.2.-]
AIN_02121	K01810	GPI, pgi; glucose-6-phosphate isomerase [EC:5.3.1.9]
AIN_02122	K01057	PGLS, pgl, devB; 6-phosphogluconolactonase [EC:3.1.1.31]
AIN_02123	K00036	G6PD, zwf; glucose-6-phosphate 1-dehydrogenase [EC:1.1.1.49 1.1.1.363]
AIN_02125	K06999	K06999; phospholipase/carboxylesterase
AIN_02126	K01247	alkA; DNA-3-methyladenine glycosylase II [EC:3.2.2.21]
AIN_02127	K05895	cobK-cbiJ; precorrin-6A/cobalt-precorrin-6A reductase [EC:1.3.1.54 1.3.1.106]
AIN_02128	K02188	cbiD; cobalt-precorrin-5B (C1)-methyltransferase [EC:2.1.1.195]
AIN_02129	K02303	cobA; uroporphyrin-III C-methyltransferase [EC:2.1.1.107]
AIN_02130	K02224	cobB-cbiA; cobyrinic acid a,c-diamide synthase [EC:6.3.5.9 6.3.5.11]
AIN_02133	K01649	leuA, IMS; 2-isopropylmalate synthase [EC:2.3.3.13]
AIN_02134	K01883	CARS, cysS; cysteinyl-tRNA synthetase [EC:6.1.1.16]
AIN_02136	K13639	soxR; MerR family transcriptional regulator, redox-sensitive transcriptional activator SoxR
AIN_02139	K15738	uup; ABC transport system ATP-binding/permease protein
AIN_02141	K07305	msrB; peptide-methionine (R)-S-oxide reductase [EC:1.8.4.12]
AIN_02144	K02911	RP-L32, MRPL32, rpmF; large subunit ribosomal protein L32
AIN_02145	K03621	plsX; phosphate acyltransferase [EC:2.3.1.274]
AIN_02146	K00648	fabH; 3-oxoacyl-[acyl-carrier-protein] synthase III [EC:2.3.1.180]
AIN_02147	K04764	ihfA, himA; integration host factor subunit alpha
AIN_02150	K01494	dcd; dCTP deaminase [EC:3.5.4.13]
AIN_02152	K06024	scpB; segregation and condensation protein B
AIN_02153	K05896	scpA; segregation and condensation protein A
AIN_02154	K01207	nagZ; beta-N-acetylhexosaminidase [EC:3.2.1.52]
AIN_02156	K01887	RARS, argS; arginyl-tRNA synthetase [EC:6.1.1.19]
AIN_02157	K01129	dgt; dGTPase [EC:3.1.5.1]
AIN_02159	K01142	E3.1.11.2, xthA; exodeoxyribonuclease III [EC:3.1.11.2]
AIN_02161	K06204	dksA; RNA polymerase-binding transcription factor
AIN_02166	K09989	K09989; uncharacterized protein
AIN_02171	K06969	rlmI; 23S rRNA (cytosine1962-C5)-methyltransferase [EC:2.1.1.191]
AIN_02172	K02529	galR; LacI family transcriptional regulator, galactose operon repressor
AIN_02175	K02027	ABC.MS.S; multiple sugar transport system substrate-binding protein

AIN_02176	K02025	ABC.MS.P; multiple sugar transport system permease protein
AIN_02177	K02026	ABC.MS.P1; multiple sugar transport system permease protein
AIN_02179	K10112	msmX, msmK, malK, sugC, ggtA, msiK; multiple sugar transport system ATP-binding protein [EC:7.5.2.-]
AIN_02180	K10112	msmX, msmK, malK, sugC, ggtA, msiK; multiple sugar transport system ATP-binding protein [EC:7.5.2.-]
AIN_02181	K00863	DAK, TKFC; triose/dihydroxyacetone kinase / FAD-AMP lyase (cyclizing) [EC:2.7.1.28 2.7.1.29 4.6.1.15]
AIN_02183	K07568	queA; S-adenosylmethionine:tRNA ribosyltransferase-isomerase [EC:2.4.99.17]
AIN_02185	K03564	BCP, PRXQ, DOT5; thioredoxin-dependent peroxiredoxin [EC:1.11.1.24]
AIN_02193	K02836	prfB; peptide chain release factor 2
AIN_02194	K05366	mrcA; penicillin-binding protein 1A [EC:2.4.99.28 3.4.16.4]
AIN_02195	K01448	amiABC; N-acetylmuramoyl-L-alanine amidase [EC:3.5.1.28]
AIN_02200	K03526	gcpE, ispG; (E)-4-hydroxy-3-methylbut-2-enyl-diphosphate synthase [EC:1.17.7.1 1.17.7.3]
AIN_02202	K00643	E2.3.1.37, ALAS; 5-aminolevulinate synthase [EC:2.3.1.37]
AIN_02214	K00558	DNMT1, dcm; DNA (cytosine-5)-methyltransferase 1 [EC:2.1.1.37]
AIN_02215	K07458	vsr; DNA mismatch endonuclease, patch repair protein [EC:3.1.-.-]
AIN_02219	K06223	dam; DNA adenine methylase [EC:2.1.1.72]
AIN_02227	K01790	rfbC, rmlC; dTDP-4-dehydrorhamnose 3,5-epimerase [EC:5.1.3.13]
AIN_02228	K01710	rfbB, rmlB, rffG; dTDP-glucose 4,6-dehydratase [EC:4.2.1.46]
AIN_02229	K00067	rfbD, rmlD; dTDP-4-dehydrorhamnose reductase [EC:1.1.1.133]
AIN_02230	K00973	rfbA, rmlA, rffH; glucose-1-phosphate thymidylyltransferase [EC:2.7.7.24]
AIN_02233	K01406	prtC; serralysin [EC:3.4.24.40]
AIN_02234	K09689	kpsT; capsular polysaccharide transport system ATP-binding protein [EC:7.6.2.12]
AIN_02235	K10107	kpsE; capsular polysaccharide transport system permease protein
AIN_02236	K09688	kpsM; capsular polysaccharide transport system permease protein
AIN_02238	K02474	wbpO; UDP-N-acetyl-D-glucosamine/UDP-N-acetyl-D-galactosamine dehydrogenase [EC:1.1.1.136 1.1.1.-]
AIN_02242	K08281	pncA; nicotinamidase/pyrazinamidase [EC:3.5.1.19 3.5.1.-]
AIN_02243	K07146	K07146; UPF0176 protein
AIN_02247	K03667	hslU; ATP-dependent HslUV protease ATP-binding subunit HslU
AIN_02250	K01419	hslV, clpQ; ATP-dependent HslUV protease, peptidase subunit HslV [EC:3.4.25.2]
AIN_02251	K03671	TXN, trxA; thioredoxin
AIN_02252	K16898	addA; ATP-dependent helicase/nuclease subunit A [EC:5.6.2.4 3.1.-.-]
AIN_02254	K00992	murU; N-acetyl-alpha-D-muramate 1-phosphate uridylyltransferase [EC:2.7.7.99]
AIN_02255	K07102	amgK; N-acetylmuramate 1-kinase [EC:2.7.1.221]
AIN_02256	K06925	tsaE; tRNA threonylcarbamoyladenosine biosynthesis protein TsaE
AIN_02258	K15011	regB, regS, actS; two-component system, sensor histidine kinase RegB [EC:2.7.13.3]
AIN_02259	K07152	SCO1; protein SCO1
AIN_02260	K15012	regA, regR, actR; two-component system, response regulator RegA
AIN_02261	K10554	frcA; fructose transport system ATP-binding protein
AIN_02262	K10553	frcC; fructose transport system permease protein
AIN_02263	K10552	frcB; fructose transport system substrate-binding protein
AIN_02267	K00799	GST, gst; glutathione S-transferase [EC:2.5.1.18]
AIN_02274	K09861	K09861; uncharacterized protein
AIN_02275	K00626	ACAT, atoB; acetyl-CoA C-acetyltransferase [EC:2.3.1.9]

AIN_02276	K05805	creA; CreA protein
AIN_02277	K02003	ABC.CD.A; putative ABC transport system ATP-binding protein
AIN_02278	K02004	ABC.CD.P; putative ABC transport system permease protein
AIN_02280	K00275	pdxH, PNPO; pyridoxamine 5'-phosphate oxidase [EC:1.4.3.5]
AIN_02282	K06917	selU, mnmH; tRNA 2-selenouridine synthase [EC:2.9.1.3]
AIN_02285	K00058	serA, PHGDH; D-3-phosphoglycerate dehydrogenase / 2-oxoglutarate reductase [EC:1.1.1.95 1.1.1.399]
AIN_02286	K00831	serC, PSAT1; phosphoserine aminotransferase [EC:2.6.1.52]
AIN_02287	K01079	serB, PSPH; phosphoserine phosphatase [EC:3.1.3.3]
AIN_02290	K06941	rlmN; 23S rRNA (adenine2503-C2)-methyltransferase [EC:2.1.1.192]
AIN_02293	K00674	dapD; 2,3,4,5-tetrahydropyridine-2,6-dicarboxylate N-succinyltransferase [EC:2.3.1.117]
AIN_02297	K01439	dapE; succinyl-diaminopimelate desuccinylase [EC:3.5.1.18]
AIN_02298	K12573	rnr, vacB; ribonuclease R [EC:3.1.13.1]
AIN_02300	K08305	mltB; peptidoglycan lytic transglycosylase B [EC:4.2.2.29]
AIN_02303	K08311	nudH; putative (di)nucleoside polyphosphate hydrolase [EC:3.6.1.-]
AIN_02304	K03797	E3.4.21.102, prc, ctpA; carboxyl-terminal processing protease [EC:3.4.21.102]
AIN_02305	K22719	envC; murein hydrolase activator
AIN_02306	K15633	gpmI; 2,3-bisphosphoglycerate-independent phosphoglycerate mutase [EC:5.4.2.12]
AIN_02307	K00783	rlmH; 23S rRNA (pseudouridine1915-N3)-methyltransferase [EC:2.1.1.177]
AIN_02308	K09710	ybeB; ribosome-associated protein
AIN_02311	K01703	leuC, IPMI-L; 3-isopropylmalate/(R)-2-methylmalate dehydratase large subunit [EC:4.2.1.33 4.2.1.35]
AIN_02312	K01704	leuD, IPMI-S; 3-isopropylmalate/(R)-2-methylmalate dehydratase small subunit [EC:4.2.1.33 4.2.1.35]
AIN_02315	K00052	leuB, IMDH; 3-isopropylmalate dehydrogenase [EC:1.1.1.85]
AIN_02319	K01687	ilvD; dihydroxy-acid dehydratase [EC:4.2.1.9]
AIN_02321	K01963	accD; acetyl-CoA carboxylase carboxyl transferase subunit beta [EC:6.4.1.2 2.1.3.15]
AIN_02322	K11754	folC; dihydrofolate synthase / folylpolyglutamate synthase [EC:6.3.2.12 6.3.2.17]
AIN_02323	K06916	zapE; cell division protein ZapE
AIN_02327	K01560	E3.8.1.2; 2-haloacid dehalogenase [EC:3.8.1.2]
AIN_02328	K09768	K09768; uncharacterized protein
AIN_02329	K13936	mdcF; malonate transporter and related proteins
AIN_02334	K00121	frmA, ADH5, adhC; S-(hydroxymethyl)glutathione dehydrogenase / alcohol dehydrogenase [EC:1.1.1.284 1.1.1.1]
AIN_02339	K01255	CARP, pepA; leucyl aminopeptidase [EC:3.4.11.1]
AIN_02341	K01673	cynT, can; carbonic anhydrase [EC:4.2.1.1]
AIN_02343	K00133	asd; aspartate-semialdehyde dehydrogenase [EC:1.2.1.11]
AIN_02345	K02340	holA; DNA polymerase III subunit delta [EC:2.7.7.7]
AIN_02346	K03643	lptE, rlpB; LPS-assembly lipoprotein
AIN_02347	K01869	LARS, leuS; leucyl-tRNA synthetase [EC:6.1.1.4]
AIN_02349	K08720	ompU; outer membrane protein OmpU
AIN_02350	K06997	yggS, PROSC; PLP dependent protein
AIN_02351	K00425	cydA; cytochrome bd ubiquinol oxidase subunit I [EC:7.1.1.7]
AIN_02352	K00426	cydB; cytochrome bd ubiquinol oxidase subunit II [EC:7.1.1.7]
AIN_02354	K06952	yfdR; 5'-nucleotidase [EC:3.1.3.89]
AIN_02357	K01754	E4.3.1.19, ilvA, tdcB; threonine dehydratase [EC:4.3.1.19]

AIN_02358	K01251	AHCY, ahcY; adenosylhomocysteinase [EC:3.13.2.1]
AIN_02365	K00057	gpsA; glycerol-3-phosphate dehydrogenase (NAD(P)+) [EC:1.1.1.94]
AIN_02366	K00012	UGDH, ugd; UDPglucose 6-dehydrogenase [EC:1.1.1.22]
AIN_02367	K06041	kdsD, kpsF; arabinose-5-phosphate isomerase [EC:5.3.1.13]
AIN_02368	K01627	kdsA; 2-dehydro-3-deoxyphosphooctonate aldolase (KDO 8-P synthase) [EC:2.5.1.55]
AIN_02373	K01784	galE, GALE; UDP-glucose 4-epimerase [EC:5.1.3.2]
AIN_02374	K25706	tsaD; tRNA N6-adenosine threonylcarbamoyltransferase [EC:2.3.1.234]
AIN_02375	K01719	hemD, UROS; uroporphyrinogen-III synthase [EC:4.2.1.75]
AIN_02377	K02498	hemY; HemY protein
AIN_02379	K03824	yhbS; putative acetyltransferase [EC:2.3.1.-]
AIN_02383	K00219	fadH; 2,4-dienoyl-CoA reductase (NADPH2) [EC:1.3.1.34]
AIN_02386	K03286	TC.OOP; OmpA-OmpF porin, OOP family
AIN_02390	K06206	sfsA; sugar fermentation stimulation protein A
AIN_02391	K01265	map; methionyl aminopeptidase [EC:3.4.11.18]
AIN_02392	K21395	yiaO; TRAP-type transport system periplasmic protein
AIN_02394	K08316	rsmD; 16S rRNA (guanine966-N2)-methyltransferase [EC:2.1.1.171]
AIN_02395	K00529	hcaD; 3-phenylpropionate/trans-cinnamate dioxygenase ferredoxin reductase component [EC:1.18.1.3]
AIN_02396	K24138	prx3; glutaredoxin/glutathione-dependent peroxiredoxin [EC:1.11.1.25 1.11.1.27]
AIN_02397	K03442	mscS; small conductance mechanosensitive channel
AIN_02398	K01724	PCBD, phhB; 4a-hydroxytetrahydrobiopterin dehydratase [EC:4.2.1.96]
AIN_02399	K09919	K09919; uncharacterized protein
AIN_02402	K12537	hasE, prtE, rsaE, prsE, eexE; membrane fusion protein, type I secretion system
AIN_02403	K12536	hasD, prtD, aprD, rsaD, prsD, eexD; ATP-binding cassette, subfamily C, type I secretion system permease/ATPase
AIN_02406	K04751	glnB; nitrogen regulatory protein P-II 1
AIN_02407	K03320	amt, AMT, MEP; ammonium transporter, Amt family
AIN_02408	K00832	tyrB; aromatic-amino-acid transaminase [EC:2.6.1.57]
AIN_02409	K01011	TST, MPST, sseA; thiosulfate/3-mercaptopyruvate sulfurtransferase [EC:2.8.1.1 2.8.1.2]
AIN_02410	K03664	smpB; SsrA-binding protein
AIN_02411	K03566	gcvA; LysR family transcriptional regulator, glycine cleavage system transcriptional activator
AIN_02416	K08309	slt; peptidoglycan lytic transglycosylase [EC:4.2.2.29]
AIN_02417	K01714	dapA; 4-hydroxy-tetrahydrodipicolinate synthase [EC:4.3.3.7]
AIN_02419	K01652	E2.2.1.6L, ilvB, ilvG, ilvI; acetolactate synthase I/II/III large subunit [EC:2.2.1.6]
AIN_02421	K19221	cobA, btuR; cob(I)alamin adenosyltransferase [EC:2.5.1.17]
AIN_02425	K06518	cidA; holin-like protein
AIN_02428	K02230	cobN; cobaltochelatase CobN [EC:6.6.1.2]
AIN_02430	K03829	yedL; putative acetyltransferase [EC:2.3.1.-]
AIN_02431	K02234	cobW; cobalamin biosynthesis protein CobW
AIN_02432	K03192	ureJ; urease accessory protein
AIN_02433	K03529	smc; chromosome segregation protein
AIN_02437	K02227	cbiB, cobD; adenosylcobinamide-phosphate synthase [EC:6.3.1.10]
AIN_02438	K02225	cobC1, cobC; cobalamin biosynthesis protein CobC
AIN_02441	K07393	ECM4, yqjG; glutathionyl-hydroquinone reductase [EC:1.8.5.7]

AIN_02443	K03795	cbiX; sirohydrochlorin cobaltochelatase [EC:4.99.1.3]
AIN_02444	K06042	cobH-cbiC; precorrin-8X/cobalt-precorrin-8 methylmutase [EC:5.4.99.61 5.4.99.60]
AIN_02445	K00595	cobL-cbiET; precorrin-6B C5,15-methyltransferase / cobalt-precorrin-6B C5,C15-methyltransferase [EC:2.1.1.132 2.1.1.289 2.1.1.196]
AIN_02446	K03394	cobI-cbiL; precorrin-2/cobalt-factor-2 C20-methyltransferase [EC:2.1.1.130 2.1.1.151]
AIN_02447	K13541	cbiGH-cobJ; cobalt-precorrin 5A hydrolase / cobalt-factor III methyltransferase / precorrin-3B C17-methyltransferase [EC:3.7.1.12 2.1.1.272 2.1.1.131]
AIN_02448	K05936	cobM, cbiF; precorrin-4/cobalt-precorrin-4 C11-methyltransferase [EC:2.1.1.133 2.1.1.271]
AIN_02452	K14155	patB, malY; cysteine-S-conjugate beta-lyase [EC:4.4.1.13]
AIN_02453	K01462	PDF, def; peptide deformylase [EC:3.5.1.88]
AIN_02454	K01462	PDF, def; peptide deformylase [EC:3.5.1.88]
AIN_02455	K01462	PDF, def; peptide deformylase [EC:3.5.1.88]
AIN_02456	K00604	MTFMT, fmt; methionyl-tRNA formyltransferase [EC:2.1.2.9]
AIN_02457	K01561	dehH; haloacetate dehalogenase [EC:3.8.1.3]
AIN_02459	K03469	rnhA, RNASEH1; ribonuclease HI [EC:3.1.26.4]
AIN_02462	K03527	ispH, lytB; 4-hydroxy-3-methylbut-2-en-1-yl diphosphate reductase [EC:1.17.7.4]
AIN_02467	K02027	ABC.MS.S; multiple sugar transport system substrate-binding protein
AIN_02468	K02025	ABC.MS.P; multiple sugar transport system permease protein
AIN_02469	K02026	ABC.MS.P1; multiple sugar transport system permease protein
AIN_02471	K01755	argH, ASL; argininosuccinate lyase [EC:4.3.2.1]
AIN_02472	K10112	msmX, msmK, malK, sugC, ggtA, msiK; multiple sugar transport system ATP-binding protein [EC:7.5.2.-]
AIN_02476	K00950	folK; 2-amino-4-hydroxy-6-hydroxymethyldihydropteridine diphosphokinase [EC:2.7.6.3]
AIN_02477	K03060	rpoZ; DNA-directed RNA polymerase subunit omega [EC:2.7.7.6]
AIN_02478	K01139	spoT; GTP diphosphokinase / guanosine-3',5'-bis(diphosphate) 3'-diphosphatase [EC:2.7.6.5 3.1.7.2]
AIN_02479	K09928	K09928; uncharacterized protein
AIN_02480	K03474	pdxJ; pyridoxine 5-phosphate synthase [EC:2.6.99.2]
AIN_02482	K00997	acpS; holo-[acyl-carrier protein] synthase [EC:2.7.8.7]
AIN_02483	K03100	lepB; signal peptidase I [EC:3.4.21.89]
AIN_02484	K03100	lepB; signal peptidase I [EC:3.4.21.89]
AIN_02485	K03685	rnc, DROSHA, RNT1; ribonuclease III [EC:3.1.26.3]
AIN_02486	K03595	era, ERAL1; GTPase
AIN_02494	K03584	recO; DNA repair protein RecO (recombination protein O)
AIN_02498	K14448	mcd; (2S)-methylsuccinyl-CoA dehydrogenase [EC:1.3.8.12]
AIN_02499	K07090	K07090; uncharacterized protein
AIN_02501	K01610	pckA; phosphoenolpyruvate carboxykinase (ATP) [EC:4.1.1.49]
AIN_02502	K14981	chvI; two-component system, OmpR family, response regulator ChvI
AIN_02503	K14980	chvG; two-component system, OmpR family, sensor histidine kinase ChvG [EC:2.7.13.3]
AIN_02505	K06958	rapZ; RNase adapter protein RapZ
AIN_02506	K02793	manXa; mannose PTS system EIIA component [EC:2.7.1.191]
AIN_02507	K02784	ptsH; phosphocarrier protein HPr
AIN_02509	K00074	paaH, hbd, fadB, mmgB; 3-hydroxybutyryl-CoA dehydrogenase [EC:1.1.1.157]
AIN_02511	K03522	fixB, etfA; electron transfer flavoprotein alpha subunit
AIN_02512	K03521	fixA, etfB; electron transfer flavoprotein beta subunit
AIN_02513	K01999	livK; branched-chain amino acid transport system substrate-binding protein

AIN_02514	K00798	MMAB, pduO; cob(I)alamin adenosyltransferase [EC:2.5.1.17]
AIN_02518	K02621	parC; topoisomerase IV subunit A [EC:5.6.2.2]
AIN_02521	K02358	tuf, TUFM; elongation factor Tu
AIN_02522	K02946	RP-S10, MRPS10, rpsJ; small subunit ribosomal protein S10
AIN_02523	K02906	RP-L3, MRPL3, rplC; large subunit ribosomal protein L3
AIN_02524	K02926	RP-L4, MRPL4, rplD; large subunit ribosomal protein L4
AIN_02525	K02892	RP-L23, MRPL23, rplW; large subunit ribosomal protein L23
AIN_02526	K02886	RP-L2, MRPL2, RML2, rplB; large subunit ribosomal protein L2
AIN_02527	K02965	RP-S19, RSM19, rpsS; small subunit ribosomal protein S19
AIN_02528	K02890	RP-L22, MRPL22, rplV; large subunit ribosomal protein L22
AIN_02529	K02982	RP-S3, rpsC; small subunit ribosomal protein S3
AIN_02530	K02878	RP-L16, MRPL16, rplP; large subunit ribosomal protein L16
AIN_02532	K02904	RP-L29, rpmC; large subunit ribosomal protein L29
AIN_02533	K02961	RP-S17, MRPS17, rpsQ; small subunit ribosomal protein S17
AIN_02534	K02874	RP-L14, MRPL14, rplN; large subunit ribosomal protein L14
AIN_02535	K02895	RP-L24, MRPL24, rplX; large subunit ribosomal protein L24
AIN_02536	K02931	RP-L5, MRPL5, rplE; large subunit ribosomal protein L5
AIN_02537	K02954	RP-S14, MRPS14, rpsN; small subunit ribosomal protein S14
AIN_02538	K02994	RP-S8, MRPS8, rpsH; small subunit ribosomal protein S8
AIN_02539	K02933	RP-L6, MRPL6, rplF; large subunit ribosomal protein L6
AIN_02540	K02881	RP-L18, MRPL18, rplR; large subunit ribosomal protein L18
AIN_02541	K02988	RP-S5, MRPS5, rpsE; small subunit ribosomal protein S5
AIN_02542	K02907	RP-L30, MRPL30, rpmD; large subunit ribosomal protein L30
AIN_02544	K02876	RP-L15, MRPL15, rplO; large subunit ribosomal protein L15
AIN_02545	K03076	secY; preprotein translocase subunit SecY
AIN_02546	K00939	adk, AK; adenylate kinase [EC:2.7.4.3]
AIN_02547	K02952	RP-S13, rpsM; small subunit ribosomal protein S13
AIN_02548	K02948	RP-S11, MRPS11, rpsK; small subunit ribosomal protein S11
AIN_02549	K03040	rpoA; DNA-directed RNA polymerase subunit alpha [EC:2.7.7.6]
AIN_02550	K02879	RP-L17, MRPL17, rplQ; large subunit ribosomal protein L17
AIN_02551	K07782	sdiA; LuxR family transcriptional regulator, quorum-sensing system regulator SdiA
AIN_02553	K07478	ycaJ; putative ATPase
AIN_02554	K06199	crcB, FEX; fluoride exporter
AIN_02555	K06179	rluC; 23S rRNA pseudouridine955/2504/2580 synthase [EC:5.4.99.24]
AIN_02557	K09969	aapJ, bztA; general L-amino acid transport system substrate-binding protein
AIN_02558	K09970	aapQ, bztB; general L-amino acid transport system permease protein
AIN_02559	K09971	aapM, bztC; general L-amino acid transport system permease protein
AIN_02560	K09972	aapP, bztD; general L-amino acid transport system ATP-binding protein [EC:7.4.2.1]
AIN_02561	K08296	sixA; phosphohistidine phosphatase [EC:3.1.3.-]
AIN_02562	K00930	argB; acetylglutamate kinase [EC:2.7.2.8]
AIN_02563	K03978	engB; GTP-binding protein
AIN_02564	K03217	yidC, spoIIIJ, OXA1, ccfA; YidC/Oxa1 family membrane protein insertase

AIN_02565	K14058	ttcA; tRNA 2-thiocytidine biosynthesis protein TtcA
AIN_02567	K08998	K08998; uncharacterized protein
AIN_02568	K03536	rnpA; ribonuclease P protein component [EC:3.1.26.5]
AIN_02569	K02914	RP-L34, MRPL34, rpmH; large subunit ribosomal protein L34
AIN_02582	K01185	E3.2.1.17; lysozyme [EC:3.2.1.17]
AIN_02585	K06223	dam; DNA adenine methylase [EC:2.1.1.72]
AIN_02596	K03496	parA, soj; chromosome partitioning protein
AIN_02597	K02909	RP-L31, rpmE; large subunit ribosomal protein L31
AIN_02598	K02884	RP-L19, MRPL19, rplS; large subunit ribosomal protein L19
AIN_02599	K00554	trmD; tRNA (guanine37-N1)-methyltransferase [EC:2.1.1.228]
AIN_02601	K02860	rimM; 16S rRNA processing protein RimM
AIN_02602	K02959	RP-S16, MRPS16, rpsP; small subunit ribosomal protein S16
AIN_02603	K04092	tyrA1; chorismate mutase [EC:5.4.99.5]
AIN_02608	K03106	SRP54, ffh; signal recognition particle subunit SRP54 [EC:3.6.5.4]
AIN_02610	K01118	acpD, azoR; FMN-dependent NADH-azoreductase [EC:1.7.1.17]
AIN_02613	K02116	atpI; ATP synthase protein I
AIN_02614	K02108	ATPF0A, atpB; F-type H+-transporting ATPase subunit a
AIN_02615	K02110	ATPF0C, atpE; F-type H+-transporting ATPase subunit c
AIN_02616	K02109	ATPF0B, atpF; F-type H+-transporting ATPase subunit b
AIN_02617	K02109	ATPF0B, atpF; F-type H+-transporting ATPase subunit b
AIN_02619	K10778	ada; AraC family transcriptional regulator, regulatory protein of adaptative response / methylated-DNA-[protein]-cysteine methyltransferase [EC:2.1.1.63]
AIN_02620	K10773	NTHL1, nth; endonuclease III [EC:3.2.2.- 4.2.99.18]
AIN_02625	K02503	HINT1_2, hinT, hit; histidine triad (HIT) family protein [EC:3.9.1.-]
AIN_02626	K01187	malZ; alpha-glucosidase [EC:3.2.1.20]
AIN_02627	K07302	iorA; isoquinoline 1-oxidoreductase subunit alpha [EC:1.3.99.16]
AIN_02628	K07303	iorB; isoquinoline 1-oxidoreductase subunit beta [EC:1.3.99.16]
AIN_02631	K01473	hyuA; N-methylhydantoinase A [EC:3.5.2.14]
AIN_02632	K01474	hyuB; N-methylhydantoinase B [EC:3.5.2.14]
AIN_02634	K18981	udh; uronate dehydrogenase [EC:1.1.1.203]
AIN_02636	K02483	K02483; two-component system, OmpR family, response regulator
AIN_02638	K01783	rpe, RPE; ribulose-phosphate 3-epimerase [EC:5.1.3.1]
AIN_02639	K03490	chbR, celD; AraC family transcriptional regulator, dual regulator of chb operon
AIN_02640	K01619	deoC, DERA; deoxyribose-phosphate aldolase [EC:4.1.2.4]
AIN_02641	K00128	ALDH; aldehyde dehydrogenase (NAD+) [EC:1.2.1.3]
AIN_02653	K16137	nemR; TetR/AcrR family transcriptional regulator, transcriptional repressor for nem operon
AIN_02654	K01821	praC, xylH; 4-oxalocrotonate tautomerase [EC:5.3.2.6]
AIN_02656	K00666	K00666; fatty-acyl-CoA synthase [EC:6.2.1.-]
AIN_02661	K17722	preT; dihydropyrimidine dehydrogenase (NAD+) subunit PreT [EC:1.3.1.1]
AIN_02663	K17723	preA; dihydropyrimidine dehydrogenase (NAD+) subunit PreA [EC:1.3.1.1]
AIN_02664	K06016	pydC; beta-ureidopropionase / N-carbamoyl-L-amino-acid hydrolase [EC:3.5.1.6 3.5.1.87]
AIN_02666	K01464	DPYS, dht, hydA; dihydropyrimidinase [EC:3.5.2.2]
AIN_02668	K02049	ABC.SN.A; NitT/TauT family transport system ATP-binding protein

AIN_02671	K02050	ABC.SN.P; NitT/TauT family transport system permease protein
AIN_02672	K02050	ABC.SN.P; NitT/TauT family transport system permease protein
AIN_02673	K02051	ABC.SN.S; NitT/TauT family transport system substrate-binding protein
AIN_02675	K02986	RP-S4, NAM9, rpsD; small subunit ribosomal protein S4
AIN_02676	K00817	hisC; histidinol-phosphate aminotransferase [EC:2.6.1.9]
AIN_02677	K00220	tyrC; cyclohexadieny/prephenate dehydrogenase [EC:1.3.1.43 1.3.1.12]
AIN_02680	K07232	CHAC, chaC; glutathione-specific gamma-glutamylcyclotransferase [EC:4.3.2.7]
AIN_02682	K02557	motB; chemotaxis protein MotB
AIN_02683	K06867	K06867; uncharacterized protein
AIN_02684	K03694	clpA; ATP-dependent Clp protease ATP-binding subunit ClpA
AIN_02685	K01069	gloB, gloC, HAGH; hydroxyacylglutathione hydrolase [EC:3.1.2.6]
AIN_02688	K02113	ATPF1D, atpH; F-type H+-transporting ATPase subunit delta
AIN_02689	K02111	ATPF1A, atpA; F-type H+/Na+-transporting ATPase subunit alpha [EC:7.1.2.2 7.2.2.1]
AIN_02690	K02115	ATPF1G, atpG; F-type H+-transporting ATPase subunit gamma
AIN_02691	K02112	ATPF1B, atpD; F-type H+/Na+-transporting ATPase subunit beta [EC:7.1.2.2 7.2.2.1]
AIN_02692	K02114	ATPF1E, atpC; F-type H+-transporting ATPase subunit epsilon
AIN_02695	K00948	PRPS, prsA; ribose-phosphate pyrophosphokinase [EC:2.7.6.1]
AIN_02698	K01620	ltaE; threonine aldolase [EC:4.1.2.48]
AIN_02699	K09160	K09160; uncharacterized protein
AIN_02700	K11753	ribF; riboflavin kinase / FMN adenylyltransferase [EC:2.7.1.26 2.7.7.2]
AIN_02701	K17865	croR; 3-hydroxybutyryl-CoA dehydratase [EC:4.2.1.55]
AIN_02703	K15986	ppaC; manganese-dependent inorganic pyrophosphatase [EC:3.6.1.1]
AIN_02705	K09973	K09973; uncharacterized protein
AIN_02706	K04078	groES, HSPE1; chaperonin GroES
AIN_02707	K04077	groEL, HSPD1; chaperonin GroEL [EC:5.6.1.7]
AIN_02712	K01589	purK; 5-(carboxyamino)imidazole ribonucleotide synthase [EC:6.3.4.18]
AIN_02713	K01588	purE; 5-(carboxyamino)imidazole ribonucleotide mutase [EC:5.4.99.18]
AIN_02718	K00958	sat, met3; sulfate adenylyltransferase [EC:2.7.7.4]
AIN_02719	K00384	trxB, TRR; thioredoxin reductase (NADPH) [EC:1.8.1.9]
AIN_02723	K05810	LACC1, yfiH; purine-nucleoside/S-methyl-5'-thioadenosine phosphorylase / adenosine deaminase [EC:2.4.2.1 2.4.2.28 3.5.4.4]
AIN_02725	K13292	lgt, umpA; phosphatidylglycerol---prolipoprotein diacylglyceryl transferase [EC:2.5.1.145]
AIN_02728	K00286	proC; pyrroline-5-carboxylate reductase [EC:1.5.1.2]
AIN_02729	K06878	K06878; tRNA-binding protein
AIN_02731	K00857	tdk, TK; thymidine kinase [EC:2.7.1.21]
AIN_02732	K11811	arsH; arsenical resistance protein ArsH
AIN_02733	K16872	E2.3.1.207; beta-ketodecanoyl-[acyl-carrier-protein] synthase [EC:2.3.1.207]
AIN_02734	K01955	carB, CPA2; carbamoyl-phosphate synthase large subunit [EC:6.3.5.5]
AIN_02736	K00574	cfa; cyclopropane-fatty-acyl-phospholipid synthase [EC:2.1.1.79]
AIN_02737	K01876	DARS2, aspS; aspartyl-tRNA synthetase [EC:6.1.1.12]
AIN_02741	K05606	MCEE, epi; methylmalonyl-CoA/ethylmalonyl-CoA epimerase [EC:5.1.99.1]
AIN_02743	K06203	cysZ; CysZ protein
AIN_02747	K11068	hlyIII; hemolysin III

AIN_02749	K14441	rimO; ribosomal protein S12 methylthiotransferase [EC:2.8.4.4]
AIN_02750	K06999	K06999; phospholipase/carboxylesterase
AIN_02751	K15975	K15975; glyoxalase family protein
AIN_02754	K10238	thuG, sugB; trehalose/maltose transport system permease protein
AIN_02757	K10112	msmX, msmK, malK, sugC, ggtA, msiK; multiple sugar transport system ATP-binding protein [EC:7.5.2.-]
AIN_02762	K00127	fdoI, fdsG; formate dehydrogenase subunit gamma
AIN_02763	K00124	fdoH, fdsB; formate dehydrogenase iron-sulfur subunit
AIN_02764	K00123	fdoG, fdhF, fdwA; formate dehydrogenase major subunit [EC:1.17.1.9]
AIN_02771	K03593	mrp, NUBPL; ATP-binding protein involved in chromosome partitioning
AIN_02774	K05772	tupA, vupA; tungstate transport system substrate-binding protein
AIN_02775	K06857	tupC, vupC; tungstate transport system ATP-binding protein [EC:7.3.2.6]
AIN_02776	K05773	tupB, vupB; tungstate transport system permease protein
AIN_02778	K01457	atzF; allophanate hydrolase [EC:3.5.1.54]
AIN_02779	K07160	pxpA; 5-oxoprolinase (ATP-hydrolysing) subunit A [EC:3.5.2.9]
AIN_02781	K21929	udg; uracil-DNA glycosylase [EC:3.2.2.27]
AIN_02782	K13924	cheBR; two-component system, chemotaxis family, CheB/CheR fusion protein [EC:2.1.1.80 3.1.1.61]
AIN_02784	K07278	tamA; translocation and assembly module TamA
AIN_02785	K06173	truA, PUS1; tRNA pseudouridine38-40 synthase [EC:5.4.99.12]
AIN_02786	K06918	K06918; uncharacterized protein
AIN_02787	K08990	ycjF; putative membrane protein
AIN_02788	K00384	trxB, TRR; thioredoxin reductase (NADPH) [EC:1.8.1.9]
AIN_02790	K01870	IARS, ileS; isoleucyl-tRNA synthetase [EC:6.1.1.5]
AIN_02792	K01004	pcs; phosphatidylcholine synthase [EC:2.7.8.24]
AIN_02793	K03733	xerC; integrase/recombinase XerC
AIN_02794	K09921	K09921; uncharacterized protein
AIN_02795	K00616	TALDO1, talB, talA; transaldolase [EC:2.2.1.2]
AIN_02796	K04066	priA; primosomal protein N' (replication factor Y) (superfamily II helicase) [EC:5.6.2.4]
AIN_02798	K07304	msrA; peptide-methionine (S)-S-oxide reductase [EC:1.8.4.11]
AIN_02799	K02687	prmA; ribosomal protein L11 methyltransferase [EC:2.1.1.-]
AIN_02802	K01159	ruvC; crossover junction endodeoxyribonuclease RuvC [EC:3.1.21.10]
AIN_02803	K03550	ruvA; holliday junction DNA helicase RuvA
AIN_02804	K03551	ruvB; holliday junction DNA helicase RuvB [EC:5.6.2.4]
AIN_02806	K07107	ybgC; acyl-CoA thioester hydrolase [EC:3.1.2.-]
AIN_02807	K03562	tolQ; biopolymer transport protein TolQ
AIN_02808	K03560	tolR; biopolymer transport protein TolR
AIN_02810	K03641	tolB; TolB protein
AIN_02811	K03640	pal; peptidoglycan-associated lipoprotein
AIN_02813	K04075	tilS, mesJ; tRNA(Ile)-lysidine synthase [EC:6.3.4.19]
AIN_02814	K03798	ftsH, hflB; cell division protease FtsH [EC:3.4.24.-]
AIN_02816	K01938	fhs; formate--tetrahydrofolate ligase [EC:6.3.4.3]
AIN_02817	K01491	folD; methylenetetrahydrofolate dehydrogenase (NADP+) / methenyltetrahydrofolate cyclohydrolase [EC:1.5.1.5 3.5.4.9]
AIN_02819	K06953	K06953; uncharacterized protein

AIN_02820	K03724	lhr; ATP-dependent helicase Lhr and Lhr-like helicase [EC:5.6.2.6 5.6.2.4]
AIN_02824	K02278	cpaA, tadV; prepilin peptidase CpaA [EC:3.4.23.43]
AIN_02827	K12511	tadC; tight adherence protein C
AIN_02828	K12510	tadB; tight adherence protein B
AIN_02829	K02283	cpaF, tadA; pilus assembly protein CpaF [EC:7.4.2.8]
AIN_02830	K02282	cpaE, tadZ; pilus assembly protein CpaE
AIN_02832	K02280	cpaC, rcpA; pilus assembly protein CpaC
AIN_02833	K02279	cpaB, rcpC; pilus assembly protein CpaB
AIN_02834	K02651	flp, pilA; pilus assembly protein Flp/PilA
AIN_02839	K16653	dprE1; decaprenylphospho-beta-D-ribofuranose 2-oxidase [EC:1.1.98.3]
AIN_02840	K06975	K06975; uncharacterized protein
AIN_02841	K01029	scoB; 3-oxoacid CoA-transferase subunit B [EC:2.8.3.5]
AIN_02843	K01028	scoA; 3-oxoacid CoA-transferase subunit A [EC:2.8.3.5]
AIN_02845	K03168	topA; DNA topoisomerase I [EC:5.6.2.1]
AIN_02846	K04096	smf; DNA processing protein
AIN_02849	K03568	tldD; TldD protein
AIN_02852	K00969	nadD; nicotinate-nucleotide adenylyltransferase [EC:2.7.7.18]
AIN_02853	K07259	dacB; serine-type D-Ala-D-Ala carboxypeptidase/endopeptidase (penicillin-binding protein 4) [EC:3.4.16.4 3.4.21.-]
AIN_02857	K04566	lysK; lysyl-tRNA synthetase, class I [EC:6.1.1.6]
AIN_02860	K03190	ureD, ureH; urease accessory protein
AIN_02861	K01430	ureA; urease subunit gamma [EC:3.5.1.5]
AIN_02864	K01429	ureB; urease subunit beta [EC:3.5.1.5]
AIN_02866	K01428	ureC; urease subunit alpha [EC:3.5.1.5]
AIN_02867	K03187	ureE; urease accessory protein
AIN_02868	K03188	ureF; urease accessory protein
AIN_02869	K03189	ureG; urease accessory protein
AIN_02873	K22922	vexD; Vi polysaccharide transport system permease protein
AIN_02874	K09689	kpsT; capsular polysaccharide transport system ATP-binding protein [EC:7.6.2.12]
AIN_02875	K22921	vexB; Vi polysaccharide transport system permease protein
AIN_02876	K08679	GAE, cap1J; UDP-glucuronate 4-epimerase [EC:5.1.3.6]
AIN_02879	K02474	wbpO; UDP-N-acetyl-D-glucosamine/UDP-N-acetyl-D-galactosamine dehydrogenase [EC:1.1.1.136 1.1.1.-]
AIN_02882	K09981	K09981; uncharacterized protein
AIN_02892	K16137	nemR; TetR/AcrR family transcriptional regulator, transcriptional repressor for nem operon
AIN_02894	K22318	oleB; cis-3-alkyl-4-acyloxetan-2-one decarboxylase [EC:4.1.1.114]
AIN_02897	K01563	dhaA; haloalkane dehalogenase [EC:3.8.1.5]
AIN_02907	K01821	praC, xylH; 4-oxalocrotonate tautomerase [EC:5.3.2.6]
AIN_02912	K07497	K07497; putative transposase
AIN_02913	K07497	K07497; putative transposase
AIN_02915	K21394	yiaM; TRAP-type transport system small permease protein
AIN_02918	K24955	ooxB; octopine oxidase subunit B
AIN_02920	K24954	ooxA; octopine oxidase subunit A
AIN_02922	K01752	E4.3.1.17, sdaA, sdaB, tdcG; L-serine dehydratase [EC:4.3.1.17]

AIN_02925	K20533	trbI; type IV secretion system protein TrbI
AIN_02926	K20532	trbG; type IV secretion system protein TrbG
AIN_02927	K20531	trbF; type IV secretion system protein TrbF
AIN_02928	K07344	trbL; type IV secretion system protein TrbL
AIN_02930	K20266	trbJ; type IV secretion system protein TrbJ
AIN_02931	K20530	trbE; type IV secretion system protein TrbE [EC:7.4.2.8]
AIN_02932	K20529	trbD; type IV secretion system protein TrbD
AIN_02933	K20528	trbC; type IV secretion system protein TrbC
AIN_02934	K20527	trbB; type IV secretion system protein TrbB [EC:7.4.2.8]
AIN_02936	K07116	pvdQ, quiP; acyl-homoserine-lactone acylase [EC:3.5.1.97]
AIN_02940	K16264	czcD, zitB; cobalt-zinc-cadmium efflux system protein
AIN_02946	K07156	copC, pcoC; copper resistance protein C
AIN_02950	K21600	csoR, ricR; CsoR family transcriptional regulator, copper-sensing transcriptional repressor
AIN_02954	K19156	prlF, sohA; antitoxin PrlF
AIN_02955	K19155	yhaV; toxin YhaV [EC:3.1.-.-]
AIN_02957	K03205	virD4, lvhD4; type IV secretion system protein VirD4 [EC:7.4.2.8]
AIN_02972	K03497	parB, spo0J; ParB family transcriptional regulator, chromosome partitioning protein
AIN_02976	K07462	recJ; single-stranded-DNA-specific exonuclease [EC:3.1.-.-]
AIN_02977	K11532	glpX-SEBP; fructose-1,6-bisphosphatase II / sedoheptulose-1,7-bisphosphatase [EC:3.1.3.11 3.1.3.37]
AIN_02978	K00003	hom; homoserine dehydrogenase [EC:1.1.1.3]
AIN_02985	K00261	GLUD1_2, gdhA; glutamate dehydrogenase (NAD(P)+) [EC:1.4.1.3]
AIN_02988	K05539	dusA; tRNA-dihydrouridine synthase A [EC:1.-.-.-]
AIN_02990	K06938	K06938; uncharacterized protein
AIN_02991	K07447	ruvX; putative pre-16S rRNA nuclease [EC:3.1.-.-]
AIN_02992	K02200	ccmH; cytochrome c-type biogenesis protein CcmH
AIN_02993	K00303	soxB; sarcosine oxidase, subunit beta [EC:1.5.3.24 1.5.3.1]
AIN_02996	K00304	soxD; sarcosine oxidase, subunit delta [EC:1.5.3.24 1.5.3.1]
AIN_02997	K00302	soxA; sarcosine oxidase, subunit alpha [EC:1.5.3.24 1.5.3.1]
AIN_02998	K00305	soxG; sarcosine oxidase, subunit gamma [EC:1.5.3.24 1.5.3.1]
AIN_02999	K04564	SOD2; superoxide dismutase, Fe-Mn family [EC:1.15.1.1]
AIN_03003	K24821	atm1, pexA; ATP-binding cassette, subfamily B, heavy metal transporter
AIN_03004	K18989	vexF; multidrug efflux pump
AIN_03005	K18990	vexE; membrane fusion protein, multidrug efflux system
AIN_03008	K03977	engA, der; GTPase
AIN_03011	K01875	SARS, serS; seryl-tRNA synthetase [EC:6.1.1.11]
AIN_03012	K03210	yajC; preprotein translocase subunit YajC
AIN_03013	K03072	secD; preprotein translocase subunit SecD
AIN_03014	K03074	secF; preprotein translocase subunit SecF
AIN_03015	K07090	K07090; uncharacterized protein
AIN_03017	K02193	ccmA; heme exporter protein A [EC:7.6.2.5]
AIN_03018	K02194	ccmB; heme exporter protein B
AIN_03019	K02195	ccmC; heme exporter protein C

AIN_03020	K02196	ccmD; heme exporter protein D
AIN_03021	K02199	ccmG, dsbE; cytochrome c biogenesis protein CcmG, thiol:disulfide interchange protein DsbE
AIN_03024	K01681	ACO, acnA; aconitate hydratase [EC:4.2.1.3]
AIN_03026	K02517	lpxL, htrB; Kdo2-lipid IVA lauroyltransferase/acyltransferase [EC:2.3.1.241 2.3.1.-]
AIN_03028	K01721	nthA; nitrile hydratase subunit alpha [EC:4.2.1.84]
AIN_03031	K02410	fliG; flagellar motor switch protein FliG
AIN_03034	K01756	purB, ADSL; adenylosuccinate lyase [EC:4.3.2.2]
AIN_03036	K03523	bioY; biotin transport system substrate-specific component
AIN_03041	K01915	glnA, GLUL; glutamine synthetase [EC:6.3.1.2]
AIN_03042	K04751	glnB; nitrogen regulatory protein P-II 1
AIN_03043	K23997	nnr; ADP-dependent NAD(P)H-hydrate dehydratase / NAD(P)H-hydrate epimerase [EC:4.2.1.136 5.1.99.6]
AIN_03044	K00645	fabD, MCAT, MCT1; [acyl-carrier-protein] S-malonyltransferase [EC:2.3.1.39]
AIN_03045	K00059	fabG, OAR1; 3-oxoacyl-[acyl-carrier protein] reductase [EC:1.1.1.100]
AIN_03046	K02078	acpP; acyl carrier protein
AIN_03051	K21029	moeB; molybdopterin-synthase adenylyltransferase [EC:2.7.7.80]
AIN_03054	K09458	fabF, OXSM, CEM1; 3-oxoacyl-[acyl-carrier-protein] synthase II [EC:2.3.1.179]
AIN_03055	K07082	mltG; peptidoglycan lytic transglycosylase G [EC:4.2.2.29]
AIN_03058	K00331	nuoB; NADH-quinone oxidoreductase subunit B [EC:7.1.1.2]
AIN_03059	K00332	nuoC; NADH-quinone oxidoreductase subunit C [EC:7.1.1.2]
AIN_03061	K00333	nuoD; NADH-quinone oxidoreductase subunit D [EC:7.1.1.2]
AIN_03063	K00334	nuoE; NADH-quinone oxidoreductase subunit E [EC:7.1.1.2]
AIN_03066	K00335	nuoF; NADH-quinone oxidoreductase subunit F [EC:7.1.1.2]
AIN_03071	K00336	nuoG; NADH-quinone oxidoreductase subunit G [EC:7.1.1.2]
AIN_03072	K00337	nuoH; NADH-quinone oxidoreductase subunit H [EC:7.1.1.2]
AIN_03073	K00338	nuoI; NADH-quinone oxidoreductase subunit I [EC:7.1.1.2]
AIN_03074	K01607	pcaC; 4-carboxymuconolactone decarboxylase [EC:4.1.1.44]
AIN_03075	K00339	nuoJ; NADH-quinone oxidoreductase subunit J [EC:7.1.1.2]
AIN_03077	K00340	nuoK; NADH-quinone oxidoreductase subunit K [EC:7.1.1.2]
AIN_03078	K00341	nuoL; NADH-quinone oxidoreductase subunit L [EC:7.1.1.2]
AIN_03079	K00342	nuoM; NADH-quinone oxidoreductase subunit M [EC:7.1.1.2]
AIN_03080	K00343	nuoN; NADH-quinone oxidoreductase subunit N [EC:7.1.1.2]
AIN_03081	K03524	birA; BirA family transcriptional regulator, biotin operon repressor / biotin---[acetyl-CoA-carboxylase] ligase [EC:6.3.4.15]
AIN_03082	K03525	coaX; type III pantothenate kinase [EC:2.7.1.33]
AIN_03083	K12574	rnj; ribonuclease J [EC:3.1.-.-]
AIN_03086	K02837	prfC; peptide chain release factor 3
AIN_03088	K00059	fabG, OAR1; 3-oxoacyl-[acyl-carrier protein] reductase [EC:1.1.1.100]
AIN_03089	K02010	afuC, fbpC; iron(III) transport system ATP-binding protein [EC:7.2.2.7]
AIN_03090	K03116	tatA; sec-independent protein translocase protein TatA
AIN_03091	K03117	tatB; sec-independent protein translocase protein TatB
AIN_03092	K03118	tatC; sec-independent protein translocase protein TatC
AIN_03093	K06923	K06923; uncharacterized protein
AIN_03095	K00573	E2.1.1.77, pcm; protein-L-isoaspartate(D-aspartate) O-methyltransferase [EC:2.1.1.77]

AIN_03096	K03787	surE; 5'/3'-nucleotidase [EC:3.1.3.5 3.1.3.6]
AIN_03099	K07448	mrr; restriction system protein
AIN_03100	K00764	purF, PPAT; amidophosphoribosyltransferase [EC:2.4.2.14]
AIN_03102	K03558	cvpA; membrane protein required for colicin V production
AIN_03103	K04485	radA, sms; DNA repair protein RadA/Sms
AIN_03107	K01775	alr; alanine racemase [EC:5.1.1.1]
AIN_03109	K08987	K08987; putative membrane protein
AIN_03110	K02314	dnaB; replicative DNA helicase [EC:5.6.2.3]
AIN_03111	K00762	pyrE; orotate phosphoribosyltransferase [EC:2.4.2.10]
AIN_03112	K01465	URA4, pyrC; dihydroorotase [EC:3.5.2.3]
AIN_03115	K00252	GCDH, gcdH; glutaryl-CoA dehydrogenase [EC:1.3.8.6]
AIN_03119	K07161	K07161; uncharacterized protein
AIN_03121	K07402	xdhC; xanthine dehydrogenase accessory factor
AIN_03122	K07141	mocA; molybdenum cofactor cytidylyltransferase [EC:2.7.7.76]
AIN_03124	K02520	infC, MTIF3; translation initiation factor IF-3
AIN_03125	K00528	fpr; ferredoxin/flavodoxin---NADP+ reductase [EC:1.18.1.2 1.19.1.1]
AIN_03127	K00390	cysH; phosphoadenosine phosphosulfate reductase [EC:1.8.4.8 1.8.4.10]
AIN_03128	K00381	cysI; sulfite reductase (NADPH) hemoprotein beta-component [EC:1.8.1.2]
AIN_03130	K02302	cysG; uroporphyrin-III C-methyltransferase / precorrin-2 dehydrogenase / sirohydrochlorin ferrochelatase [EC:2.1.1.107 1.3.1.76 4.99.1.4]
AIN_03131	K05800	decR, cyuR; Lrp/AsnC family transcriptional regulator, cysteine-sensing transcriptional activator
AIN_03133	K10125	dctB; two-component system, NtrC family, C4-dicarboxylate transport sensor histidine kinase DctB [EC:2.7.13.3]
AIN_03134	K10126	dctD; two-component system, NtrC family, C4-dicarboxylate transport response regulator DctD
AIN_03135	K11688	dctP; C4-dicarboxylate-binding protein DctP
AIN_03136	K11689	dctQ; C4-dicarboxylate transporter, DctQ subunit
AIN_03137	K11690	dctM; C4-dicarboxylate transporter, DctM subunit
AIN_03138	K01803	TPI, tpiA; triosephosphate isomerase (TIM) [EC:5.3.1.1]
AIN_03139	K07343	tfoX; DNA transformation protein and related proteins
AIN_03140	K13628	iscA; iron-sulfur cluster assembly protein
AIN_03141	K00661	maa; maltose O-acetyltransferase [EC:2.3.1.79]
AIN_03142	K01126	E3.1.4.46, glpQ, ugpQ; glycerophosphoryl diester phosphodiesterase [EC:3.1.4.46]
AIN_03148	K05844	rimK; ribosomal protein S6--L-glutamate ligase [EC:6.3.2.-]
AIN_03150	K00773	tgt; queuine tRNA-ribosyltransferase [EC:2.4.2.29]
AIN_03152	K01338	lon; ATP-dependent Lon protease [EC:3.4.21.53]
AIN_03153	K01243	mtnN, mtn, pfs; adenosylhomocysteine nucleosidase [EC:3.2.2.9]
AIN_03168	K06919	K06919; putative DNA primase/helicase
AIN_03192	K03543	emrA; membrane fusion protein, multidrug efflux system
AIN_03193	K11936	pgaC, icaA; poly-beta-1,6-N-acetyl-D-glucosamine synthase [EC:2.4.1.-]
AIN_03196	K01784	galE, GALE; UDP-glucose 4-epimerase [EC:5.1.3.2]
AIN_03207	K03630	radC; DNA repair protein RadC
AIN_03213	K07301	yrbG; cation:H+ antiporter
AIN_03220	K03724	lhr; ATP-dependent helicase Lhr and Lhr-like helicase [EC:5.6.2.6 5.6.2.4]
AIN_03238	K07059	K07059; rhomboid family protein

AIN_03241	K01092	E3.1.3.25, IMPA, suhB; myo-inositol-1(or 4)-monophosphatase [EC:3.1.3.25]
AIN_03243	K11085	msbA; ATP-binding cassette, subfamily B, bacterial MsbA [EC:7.5.2.6]
AIN_03244	K06980	ygfZ; tRNA-modifying protein YgfZ
AIN_03246	K02356	efp; elongation factor P
AIN_03249	K02232	cobQ, cbiP; adenosylcobyric acid synthase [EC:6.3.5.10]
AIN_03251	K12340	tolC, bepC, cyaE, raxC, sapF, rsaF, hasF; outer membrane protein
AIN_03252	K00573	E2.1.1.77, pcm; protein-L-isoaspartate(D-aspartate) O-methyltransferase [EC:2.1.1.77]
AIN_03258	K08688	E3.5.3.3; creatinase [EC:3.5.3.3]
AIN_03262	K00696	E2.4.1.14; sucrose-phosphate synthase [EC:2.4.1.14]
AIN_03264	K02012	afuA, fbpA; iron(III) transport system substrate-binding protein
AIN_03265	K02011	afuB, fbpB; iron(III) transport system permease protein
AIN_03269	K01874	MARS, metG; methionyl-tRNA synthetase [EC:6.1.1.10]
AIN_03272	K03098	APOD; apolipoprotein D and lipocalin family protein
AIN_03279	K02488	pleD; two-component system, cell cycle response regulator [EC:2.7.7.65]
AIN_03281	K01091	gph; phosphoglycolate phosphatase [EC:3.1.3.18]
AIN_03282	K14083	mttB; trimethylamine---corrinoid protein Co-methyltransferase [EC:2.1.1.250]
AIN_03287	K03707	tenA; thiaminase (transcriptional activator TenA) [EC:3.5.99.2]
AIN_03288	K00941	thiD; hydroxymethylpyrimidine/phosphomethylpyrimidine kinase [EC:2.7.1.49 2.7.4.7]
AIN_03289	K00788	thiE; thiamine-phosphate pyrophosphorylase [EC:2.5.1.3]
AIN_03290	K00878	thiM; hydroxyethylthiazole kinase [EC:2.7.1.50]
AIN_03298	K02014	TC.FEV.OM; iron complex outermembrane recepter protein
AIN_03300	K02013	ABC.FEV.A; iron complex transport system ATP-binding protein [EC:7.2.2.-]
AIN_03301	K02016	ABC.FEV.S; iron complex transport system substrate-binding protein
AIN_03302	K02015	ABC.FEV.P; iron complex transport system permease protein
AIN_03309	K06893	K06893; uncharacterized protein
AIN_03310	K06988	fno; 8-hydroxy-5-deazaflavin:NADPH oxidoreductase [EC:1.5.1.40]
AIN_03312	K11963	urtE; urea transport system ATP-binding protein
AIN_03313	K11962	urtD; urea transport system ATP-binding protein
AIN_03314	K11961	urtC; urea transport system permease protein
AIN_03315	K11960	urtB; urea transport system permease protein
AIN_03316	K11959	urtA; urea transport system substrate-binding protein
AIN_03323	K02031	ddpD; peptide/nickel transport system ATP-binding protein
AIN_03324	K02032	ddpF; peptide/nickel transport system ATP-binding protein
AIN_03325	K19746	dauA; D-arginine dehydrogenase [EC:1.4.99.6]
AIN_03328	K02033	ABC.PE.P; peptide/nickel transport system permease protein
AIN_03329	K02034	ABC.PE.P1; peptide/nickel transport system permease protein
AIN_03330	K15583	oppD; oligopeptide transport system ATP-binding protein
AIN_03331	K02032	ddpF; peptide/nickel transport system ATP-binding protein
AIN_03339	K07258	dacC, dacA, dacD; serine-type D-Ala-D-Ala carboxypeptidase (penicillin-binding protein 5/6) [EC:3.4.16.4]
AIN_03340	K01925	murD; UDP-N-acetylmuramoylalanine--D-glutamate ligase [EC:6.3.2.9]
AIN_03341	K01000	mraY; phospho-N-acetylmuramoyl-pentapeptide-transferase [EC:2.7.8.13]
AIN_03342	K01929	murF; UDP-N-acetylmuramoyl-tripeptide--D-alanyl-D-alanine ligase [EC:6.3.2.10]

AIN_03343	K01928	murE; UDP-N-acetylmuramoyl-L-alanyl-D-glutamate--2,6-diaminopimelate ligase [EC:6.3.2.13]
AIN_03344	K03587	ftsI; cell division protein FtsI (penicillin-binding protein 3) [EC:3.4.16.4]
AIN_03345	K03586	ftsL; cell division protein FtsL
AIN_03346	K03438	mraW, rsmH; 16S rRNA (cytosine1402-N4)-methyltransferase [EC:2.1.1.199]
AIN_03347	K03925	mraZ; transcriptional regulator MraZ
AIN_03348	K03593	mrp, NUBPL; ATP-binding protein involved in chromosome partitioning
AIN_03354	K03657	uvrD, pcrA; ATP-dependent DNA helicase UvrD/PcrA [EC:5.6.2.4]
AIN_03355	K00661	maa; maltose O-acetyltransferase [EC:2.3.1.79]
AIN_03358	K00101	lldD; L-lactate dehydrogenase (cytochrome) [EC:1.1.2.3]
AIN_03359	K10112	msmX, msmK, malK, sugC, ggtA, msiK; multiple sugar transport system ATP-binding protein [EC:7.5.2.-]
AIN_03360	K10108	malE; maltose/maltodextrin transport system substrate-binding protein
AIN_03361	K10109	malF; maltose/maltodextrin transport system permease protein
AIN_03362	K10110	malG; maltose/maltodextrin transport system permease protein
AIN_03366	K01958	PC, pyc; pyruvate carboxylase [EC:6.4.1.1]
AIN_03372	K01693	hisB; imidazoleglycerol-phosphate dehydratase [EC:4.2.1.19]
AIN_03373	K02501	hisH; imidazole glycerol-phosphate synthase subunit HisH [EC:4.3.2.10]
AIN_03375	K01814	hisA; phosphoribosylformimino-5-aminoimidazole carboxamide ribotide isomerase [EC:5.3.1.16]
AIN_03378	K03702	uvrB; excinuclease ABC subunit B
AIN_03389	K04565	SOD1; superoxide dismutase, Cu-Zn family [EC:1.15.1.1]
AIN_03392	K07246	ttuC, dmlA; tartrate dehydrogenase/decarboxylase / D-malate dehydrogenase [EC:1.1.1.93 4.1.1.73 1.1.1.83]
AIN_03393	K02058	ABC.SS.S; simple sugar transport system substrate-binding protein
AIN_03394	K02056	ABC.SS.A; simple sugar transport system ATP-binding protein [EC:7.5.2.-]
AIN_03395	K02057	ABC.SS.P; simple sugar transport system permease protein
AIN_03396	K02057	ABC.SS.P; simple sugar transport system permease protein
AIN_03397	K18910	dpe, lre; D-psicose/D-tagatose/L-ribulose 3-epimerase [EC:5.1.3.30 5.1.3.31]
AIN_03399	K00875	rbtK, FGGY; D-ribulokinase [EC:2.7.1.47]
AIN_03400	K03435	fruR1, fruR; LacI family transcriptional regulator, fructose operon transcriptional repressor
AIN_03402	K01840	manB; phosphomannomutase [EC:5.4.2.8]
AIN_03403	K16011	algA, xanB, rfbA, wbpW, pslB; mannose-1-phosphate guanylyltransferase / mannose-6-phosphate isomerase [EC:2.7.7.13 5.3.1.8]
AIN_03404	K00847	E2.7.1.4, scrK; fructokinase [EC:2.7.1.4]
AIN_03405	K01915	glnA, GLUL; glutamine synthetase [EC:6.3.1.2]
AIN_03406	K11074	potI, spuH; putrescine transport system permease protein
AIN_03407	K11075	potH, spuG; putrescine transport system permease protein
AIN_03408	K11076	potG, spuF; putrescine transport system ATP-binding protein [EC:7.6.2.16]
AIN_03409	K11073	potF, spuD, spuE; putrescine transport system substrate-binding protein
AIN_03410	K12256	spuC; putrescine---pyruvate transaminase [EC:2.6.1.113]
AIN_03411	K09471	puuB, ordL; gamma-glutamylputrescine oxidase [EC:1.4.3.-]
AIN_03412	K08151	tetA; MFS transporter, DHA1 family, tetracycline resistance protein
AIN_03414	K01754	E4.3.1.19, ilvA, tdcB; threonine dehydratase [EC:4.3.1.19]
AIN_03416	K01560	E3.8.1.2; 2-haloacid dehalogenase [EC:3.8.1.2]
AIN_03418	K00208	fabI; enoyl-[acyl-carrier protein] reductase I [EC:1.3.1.9 1.3.1.10]
AIN_03419	K00647	fabB; 3-oxoacyl-[acyl-carrier-protein] synthase I [EC:2.3.1.41]

AIN_03420	K01716	fabA; 3-hydroxyacyl-[acyl-carrier protein] dehydratase / trans-2-decenoyl-[acyl-carrier protein] isomerase [EC:4.2.1.59 5.3.3.14]
AIN_03421	K09826	irr; Fur family transcriptional regulator, iron response regulator
AIN_03423	K09966	K09966; uncharacterized protein
AIN_03427	K01696	trpB; tryptophan synthase beta chain [EC:4.2.1.20]
AIN_03428	K01817	trpF; phosphoribosylanthranilate isomerase [EC:5.3.1.24]
AIN_03430	K05788	ihfB, himD; integration host factor subunit beta
AIN_03431	K02945	RP-S1, rpsA; small subunit ribosomal protein S1
AIN_03432	K00945	cmk; CMP/dCMP kinase [EC:2.7.4.25]
AIN_03434	K00800	aroA; 3-phosphoshikimate 1-carboxyvinyltransferase [EC:2.5.1.19]
AIN_03435	K03439	trmB, METTL1, TRM8; tRNA (guanine-N7-)-methyltransferase [EC:2.1.1.33]
AIN_03436	K00789	metK, MAT; S-adenosylmethionine synthetase [EC:2.5.1.6]
AIN_03437	K03820	lnt; apolipoprotein N-acyltransferase [EC:2.3.1.269]
AIN_03438	K06189	corC, tlyC; hemolysin (HlyC) family protein
AIN_03439	K07042	ybeY, yqfG; probable rRNA maturation factor
AIN_03440	K06217	phoH, phoL; phosphate starvation-inducible protein PhoH and related proteins
AIN_03441	K06168	miaB; tRNA-2-methylthio-N6-dimethylallyladenosine synthase [EC:2.8.4.3]
AIN_03444	K01056	PTH1, PTRH1, pth, spoVC; peptidyl-tRNA hydrolase, PTH1 family [EC:3.1.1.29]
AIN_03447	K02897	RP-L25, rplY; large subunit ribosomal protein L25
AIN_03449	K00101	lldD; L-lactate dehydrogenase (cytochrome) [EC:1.1.2.3]
AIN_03458	K01695	trpA; tryptophan synthase alpha chain [EC:4.2.1.20]
AIN_03460	K06942	ychF; ribosome-binding ATPase
AIN_03466	K22131	otnI; 2-dehydrotetronate isomerase [EC:5.3.1.35]
AIN_03467	K22130	otnC; 3-dehydro-4-phosphotetronate decarboxylase [EC:4.1.1.104]
AIN_03468	K21948	otnK; 3-dehydrotetronate 4-kinase [EC:2.7.1.217]
AIN_03469	K08319	ltnD; L-threonate 2-dehydrogenase [EC:1.1.1.411]
AIN_03473	K22025	denD; D-erythronate 2-dehydrogenase [EC:1.1.1.410]
AIN_03474	K23246	apnL; D-apionolactonase [EC:3.1.1.115]
AIN_03475	K10440	rbsC; ribose transport system permease protein
AIN_03476	K17215	K17215; inositol transport system ATP-binding protein
AIN_03477	K02058	ABC.SS.S; simple sugar transport system substrate-binding protein
AIN_03479	K00641	metX; homoserine O-acetyltransferase/O-succinyltransferase [EC:2.3.1.31 2.3.1.46]
AIN_03482	K10834	hmuV, phuV, bhuV, hemV; heme transport system ATP-binding protein [EC:7.6.2.5]
AIN_03483	K25133	hmuU, phuU, bhuU, hemU; heme transport system permease protein
AIN_03484	K25132	hmuT, phuT, bhuT, hemT; heme transport system substrate-binding protein
AIN_03485	K07225	hmuS; putative hemin transport protein
AIN_03486	K16087	TC.FEV.OM3, tbpA, hemR, lbpA, hpuB, bhuR, hugA, hmbR; hemoglobin/transferrin/lactoferrin receptor protein
AIN_03487	K03832	tonB; periplasmic protein TonB
AIN_03488	K03559	exbD; biopolymer transport protein ExbD
AIN_03489	K03559	exbD; biopolymer transport protein ExbD
AIN_03490	K03561	exbB; biopolymer transport protein ExbB
AIN_03493	K05373	ybtX, irp8; MFS transporter, putative signal transducer
AIN_03494	K02014	TC.FEV.OM; iron complex outermembrane recepter protein

AIN_03503	K01118	acpD, azoR; FMN-dependent NADH-azoreductase [EC:1.7.1.17]
AIN_03504	K07104	catE; catechol 2,3-dioxygenase [EC:1.13.11.2]
AIN_03509	K09022	ridA, tdcF, RIDA; 2-iminobutanoate/2-iminopropanoate deaminase [EC:3.5.99.10]
AIN_03511	K23775	ohrR; MarR family transcriptional regulator, organic hydroperoxide resistance regulator
AIN_03513	K03498	trkH, trkG, ktrB, ktrD; trk/ktr system potassium uptake protein
AIN_03515	K01878	glyQ; glycyl-tRNA synthetase alpha chain [EC:6.1.1.14]
AIN_03517	K01879	glyS; glycyl-tRNA synthetase beta chain [EC:6.1.1.14]
AIN_03518	K01006	ppdK; pyruvate, orthophosphate dikinase [EC:2.7.9.1]
AIN_03520	K01633	folB; 7,8-dihydroneopterin aldolase/epimerase/oxygenase [EC:4.1.2.25 5.1.99.8 1.13.11.81]
AIN_03521	K00796	folP; dihydropteroate synthase [EC:2.5.1.15]
AIN_03522	K03431	glmM; phosphoglucosamine mutase [EC:5.4.2.10]
AIN_03525	K00053	ilvC; ketol-acid reductoisomerase [EC:1.1.1.86]
AIN_03527	K03185	ubiH; 2-octaprenyl-6-methoxyphenol hydroxylase [EC:1.14.13.-]
AIN_03528	K07025	K07025; putative hydrolase of the HAD superfamily
AIN_03531	K01956	carA, CPA1; carbamoyl-phosphate synthase small subunit [EC:6.3.5.5]
AIN_03532	K09117	K09117; uncharacterized protein
AIN_03535	K02274	coxA, ctaD; cytochrome c oxidase subunit I [EC:7.1.1.9]
AIN_03539	K03490	chbR, celD; AraC family transcriptional regulator, dual regulator of chb operon
AIN_03541	K02035	ABC.PE.S; peptide/nickel transport system substrate-binding protein
AIN_03543	K02033	ABC.PE.P; peptide/nickel transport system permease protein
AIN_03544	K02034	ABC.PE.P1; peptide/nickel transport system permease protein
AIN_03546	K01191	MAN2C1; alpha-mannosidase [EC:3.2.1.24]
AIN_03550	K03801	lipB; lipoyl(octanoyl) transferase [EC:2.3.1.181]
AIN_03552	K02051	ABC.SN.S; NitT/TauT family transport system substrate-binding protein
AIN_03553	K02049	ABC.SN.A; NitT/TauT family transport system ATP-binding protein
AIN_03554	K02050	ABC.SN.P; NitT/TauT family transport system permease protein
AIN_03556	K02052	ABC.SP.A; putative spermidine/putrescine transport system ATP-binding protein
AIN_03557	K02055	ABC.SP.S; putative spermidine/putrescine transport system substrate-binding protein
AIN_03559	K02054	ABC.SP.P1; putative spermidine/putrescine transport system permease protein
AIN_03560	K02053	ABC.SP.P; putative spermidine/putrescine transport system permease protein
AIN_03562	K13936	mdcF; malonate transporter and related proteins
AIN_03563	K06167	phnP; phosphoribosyl 1,2-cyclic phosphate phosphodiesterase [EC:3.1.4.55]
AIN_03564	K03424	tatD; TatD DNase family protein [EC:3.1.21.-]
AIN_03565	K02341	holB; DNA polymerase III subunit delta' [EC:2.7.7.7]
AIN_03588	K06919	K06919; putative DNA primase/helicase
AIN_03597	K02355	fusA, GFM, EFG; elongation factor G
AIN_03598	K02992	RP-S7, MRPS7, rpsG; small subunit ribosomal protein S7
AIN_03599	K02950	RP-S12, MRPS12, rpsL; small subunit ribosomal protein S12
AIN_03601	K01512	acyP; acylphosphatase [EC:3.6.1.7]
AIN_03602	K03046	rpoC; DNA-directed RNA polymerase subunit beta' [EC:2.7.7.6]
AIN_03603	K03043	rpoB; DNA-directed RNA polymerase subunit beta [EC:2.7.7.6]
AIN_03604	K02935	RP-L7, MRPL12, rplL; large subunit ribosomal protein L7/L12

AIN_03605	K02864	RP-L10, MRPL10, rplJ; large subunit ribosomal protein L10
AIN_03606	K02863	RP-L1, MRPL1, rplA; large subunit ribosomal protein L1
AIN_03607	K02867	RP-L11, MRPL11, rplK; large subunit ribosomal protein L11
AIN_03608	K02601	nusG; transcription termination/antitermination protein NusG
AIN_03609	K03073	secE; preprotein translocase subunit SecE
AIN_03611	K07305	msrB; peptide-methionine (R)-S-oxide reductase [EC:1.8.4.12]
AIN_03620	K11177	yagR; xanthine dehydrogenase YagR molybdenum-binding subunit [EC:1.17.1.4]
AIN_03621	K11178	yagS; xanthine dehydrogenase YagS FAD-binding subunit [EC:1.17.1.4]
AIN_03622	K13483	yagT; xanthine dehydrogenase YagT iron-sulfur-binding subunit
AIN_03627	K01469	OPLAH, OXP1, oplAH; 5-oxoprolinase (ATP-hydrolysing) [EC:3.5.2.9]
AIN_03634	K11904	vgrG; type VI secretion system secreted protein VgrG
